# Engineered calprotectin-sensing probiotics for IBD surveillance in humans

**DOI:** 10.1073/pnas.2221121120

**Published:** 2023-07-31

**Authors:** Jonathan Y. Xia, Chelsea Hepler, Peter Tran, Nathan J. Waldeck, Joseph Bass, Arthur Prindle

**Affiliations:** ^a^Division of Gastroenterology and Hepatology, Department of Medicine, Feinberg School of Medicine, Northwestern University, Chicago, IL 60611; ^b^Department of Biochemistry and Molecular Genetics, Feinberg School of Medicine, Northwestern University, Chicago, IL 60611; ^c^Division of Endocrinology, Metabolism and Molecular Medicine, Department of Medicine, Feinberg School of Medicine, Northwestern University, Chicago, IL 60611; ^d^Department of Chemical and Biological Engineering, Northwestern University, Evanston, IL 60208; ^e^Center for Synthetic Biology, Northwestern University, Evanston, IL 60208

**Keywords:** inflammatory bowel disease, synthetic biology, probiotics, microbiome

## Abstract

Inflammatory bowel disease (IBD) is a spectrum of autoimmune diseases affecting the gastrointestinal tract characterized by a relapsing and remitting course of gut mucosal inflammation. Disease flares can be difficult to predict, and the current practice of IBD disease activity surveillance through endoscopy is invasive and requires medical expertise. Recent advancements in synthetic biology raise the possibility that symbiotic microbes can be engineered to selectively detect disease biomarkers used in current clinical practice. Here, we introduce an engineered probiotic capable of detecting the clinical gold standard IBD biomarker, calprotectin, with sensitivity and specificity in IBD patients. Specifically, we identified a bacterial promoter in the probiotic strain *Escherichia coli* Nissle 1917 (EcN) which exhibits a specific expression increase in the presence of calprotectin. Using murine models of colitis, we show that the reporter signal is activated in vivo during transit of the GI tract following oral delivery. Furthermore, our engineered probiotic can successfully discriminate human patients with active IBD from those in remission and without IBD using patient stool samples, where the intensity of reporter signal quantitatively tracks with clinical laboratory–measured levels of calprotectin. Our pilot study sets the stage for probiotics that can be engineered to detect fecal calprotectin for precise noninvasive disease activity monitoring in IBD patients.

## Significance

Integration of synthetic biology into clinical practice promises to be a transformative approach to modernizing disease diagnosis and monitoring. In particular, inflammatory bowel disease (IBD) is a spectrum of chronic inflammatory gastrointestinal diseases that is difficult to monitor due to the relapsing and remitting nature of disease flares often resulting in downstream complications. To bridge this gap, our pilot study sets the stage for engineered calprotectin sensing probiotics that can be used as a precise and noninvasive method of disease activity monitoring in IBD patients.

Bacteria in the gut microbiome have evolved complex mechanisms of sensing and responding to external stimuli from the host. Over the past decade, advancements in molecular biology and nucleic acid sequencing have led to the emergence of synthetic biology as a potential tool to develop novel non-cell-based and cell-based clinical diagnostics (1–3). Advancements in synthetic biology have demonstrated that cell-based biosensors can be engineered by placing a reporter gene under the control of a promoter that is sensitive to the presence of specific environmental stimuli or biomarker (4, 5). Several microbes have been designed previously to serve as potential diagnostics to human infectious diseases and cancers (6–8). Furthermore, past studies have demonstrated that recombinant probiotics can be used to for successful detection of gut inflammation both in vitro and in murine models of IBD (9–12). However, these models either lacked specificity to gut inflammation or detected molecules that have not been demonstrated to be correlated with inflammatory bowel disease (IBD) activity in humans.

IBD is a spectrum of chronic autoimmune inflammatory disorders of the gastrointestinal tract ranging from ulcerative colitis to Crohn’s disease. Globally, the prevalence of IBD has been steadily increasing from 3.7 million in 1990 to 6.8 million in 2017, particularly in regions with historically low rates and more limited health care resources (13, 14). IBD is characterized by a progressive chronic relapsing and remitting course of diarrhea, rectal bleeding, and abdominal pain that can lead to irreversible bowel damage and colorectal cancer if relapses are not identified early (15). Since the clinical course of IBD varies from patient to patient, the optimal method for monitoring disease activity is difficult. Previous studies have shown that patient-reported symptoms are poor predictors of disease activity (16, 17). The current gold standard of disease surveillance through colonoscopy is invasive, costly, and requires medical expertise. C-reactive protein is a systemic biomarker that indirectly quantifies disease activity, but it is nonspecific to IBD as it is elevated in other infectious and autoimmune illnesses. Calprotectin, a stool biomarker that is both sensitive and specific to gut inflammation, is currently the clinical laboratory gold standard for IBD disease activity surveillance, but results usually take 1 to 2 weeks to obtain in the clinical setting (18, 19). Thus, a clinical gap remains in methods of precise, efficient noninvasive monitoring of disease activity.

To fill this gap, we set out to engineer the probiotic *Escherichia coli* Nissle 1917 (EcN) to detect calprotectin, the clinical gold standard biomarker of gut inflammation, for the goal of being used as a potential noninvasive diagnostic of gut inflammation. EcN is a known human gut colonizing probiotic with a long track record of safety in humans and its compatibility with the canonical genetic engineering techniques for bacteria (20). We first identified calprotectin-responsive EcN genes through RNA sequencing (RNA-seq) and coupled their respective promoters to a fluorescent reporter prior to transforming into EcN. Next, we screened these engineered EcN sensors for sensitivity and specificity to calprotectin and optimized their performance. Finally, we demonstrated that our engineered EcN sensor is sensitive and specific to gut inflammation in murine models of IBD and stool samples from human patients with active IBD. Our goal is to improve disease monitoring and identify at-risk patients earlier with the hope of maintaining remission and avoid irreversible bowel damage in this growing patient population.

## Results

### Calprotectin Treatment Results in Robust Transcriptional Changes in *E. coli* Nissle 1917.

**Fig. 1.**
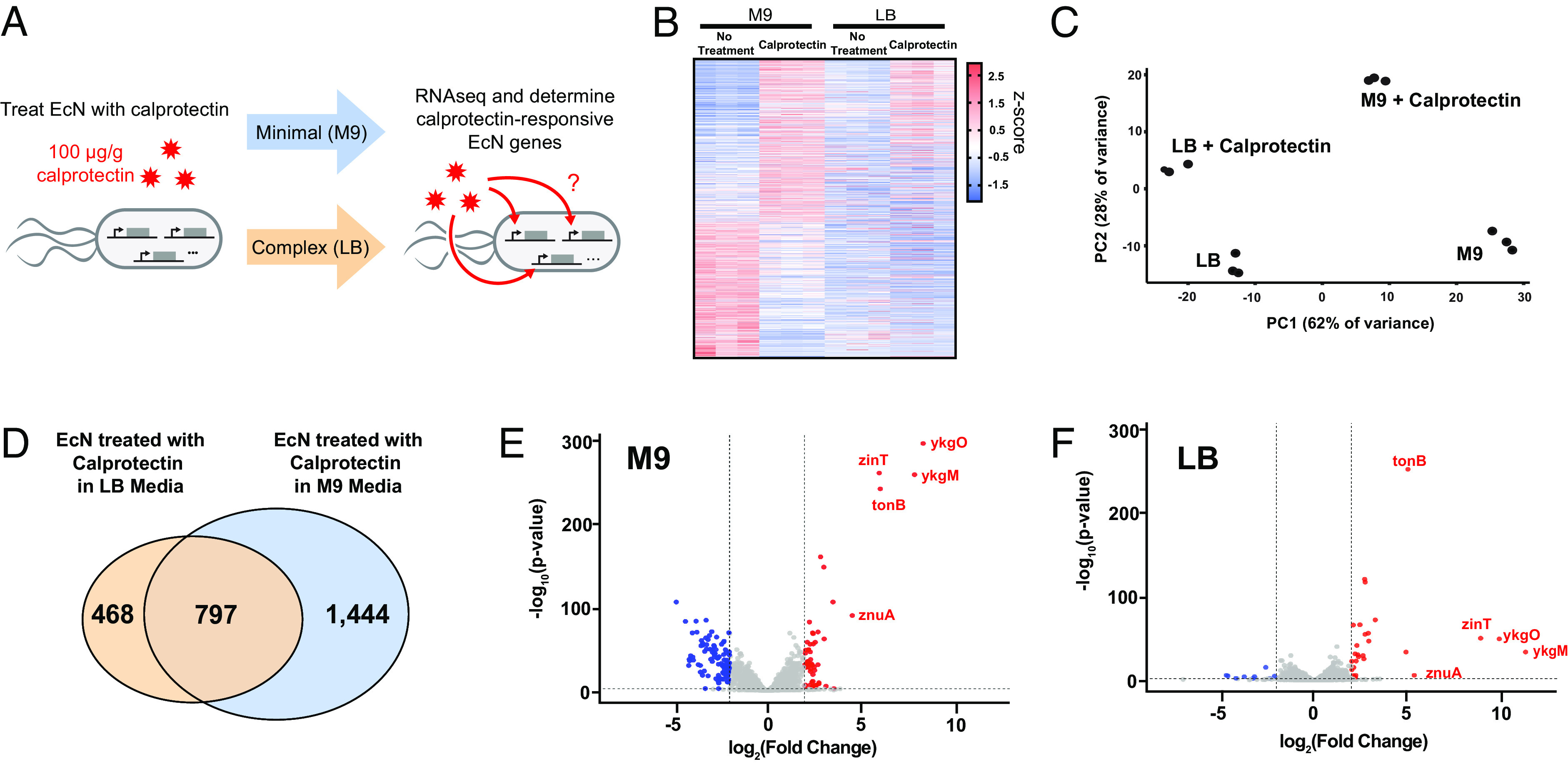
EcN show distinct gene expression changes in response to calprotectin. (*A*) RNA-seq was performed on EcN that were treated with calprotectin in either minimal (M9) or complex (LB) media. (*B*) Heat map showing all the significant (padj < 0.05) differentially expressed genes induced by calprotectin in EcN in both M9 and LB media (n = 3). (*C*) Principal component analysis performed on RNA-seq data (n = 3). (*D*) Venn diagram depicting overlap in statistically significant differentially expressed genes in EcN treated with calprotectin in LB and M9 media. (*E* and *F*) Volcano plot of all the significant (padj < 0.05) differentially expressed genes induced by calprotectin in EcN in both M9 and LB media.

Calprotectin, also known as S100A8/A9, is a heterodimer protein produced by neutrophils and monocytes that exerts antimicrobial activity through chelation of essential nutrients such as zinc and manganese, resulting in bacterial metal starvation (21). Whether engineered bacterial sensors can be used to detect calprotectin is currently unknown as it is unclear whether calprotectin elicits any transcriptional changes in bacterial species. Previous work has demonstrated that bacteria species exhibited unique transcriptomes when cultured in nutrient-rich and limited conditions (22). Although the optimal cutoff values for fecal calprotectin that indicate active inflammation can be variable across patients, values greater than 100 µg/g indicated the presence of inflammation with high levels of specificity without reducing sensitivity (23). Thus, to test whether the human probiotic strain EcN is transcriptionally responsive to calprotectin, we treated EcN with a clinically significant dose of 100 µg/g of human calprotectin and performed RNA-seq in the environments of complex (LB) and minimal media (M9) (Fig. 1*A*). Heatmaps of the statistically significant (padj < 0.05) differentially expressed genes that overlapped between calprotectin-treated EcN in M9 and LB media were generated (Fig. 1*B*). We observed a more robust transcriptional response pattern in calprotectin-treated EcN in minimal media (M9) as compared to complex media (LB). Principle component analysis reveals that the global gene expression profile of EcN treated with calprotectin is distinct from untreated in both complex (LB) and minimal (M9) media conditions (Fig. 1*C*). Overall, there were 2,241 differentially regulated transcripts from calprotectin-treated EcN in M9 media compared to 1,265 in LB media, with 797 overlapping genes (Fig. 1*D*). These results show that calprotectin elicits a robust transcriptional response in EcN that could be used for the construction of the synthetic sensor gene circuit in our probiotic based whole-cell biosensor.

Functional classification of differentially regulated genes by gene ontology analysis indicated enrichment of genes in pathways involved in ion transport, cellular metabolic processes, and cell motility (*SI Appendix*, Fig. S1 *A* and *B*). Interestingly, the up-regulated genes with the highest fold-change were involved in cellular regulation of zinc ion (Zn). This includes the expression of genes *ykgM* and *ykgO,* both under the control of the same promoter *ykgMO*, encoding the zinc-responsive 50S ribosomal subunit proteins L31 and L36, which are increased by approximately 2,500-fold and 1,000-fold respectively in the presence of calprotectin (*SI Appendix*, Table S1). Fold changes in gene expression after calprotectin treatment appeared to be more robust in EcN grown in minimal media (M9) compared to complex media (LB). We identified five genes with both the highest fold-change and statistical significance after calprotectin treatment that were consistent between the 2 groups (Fig. 1 *E* and *F*). The promoters of these genes were then screened as potential targets to be used for construction of the biosensor of calprotectin.

### The Zinc-Responsive EcN Promoter *ykgMO* Can Reliably Sense Elevations in Calprotectin In Vitro.

**Fig. 2.**
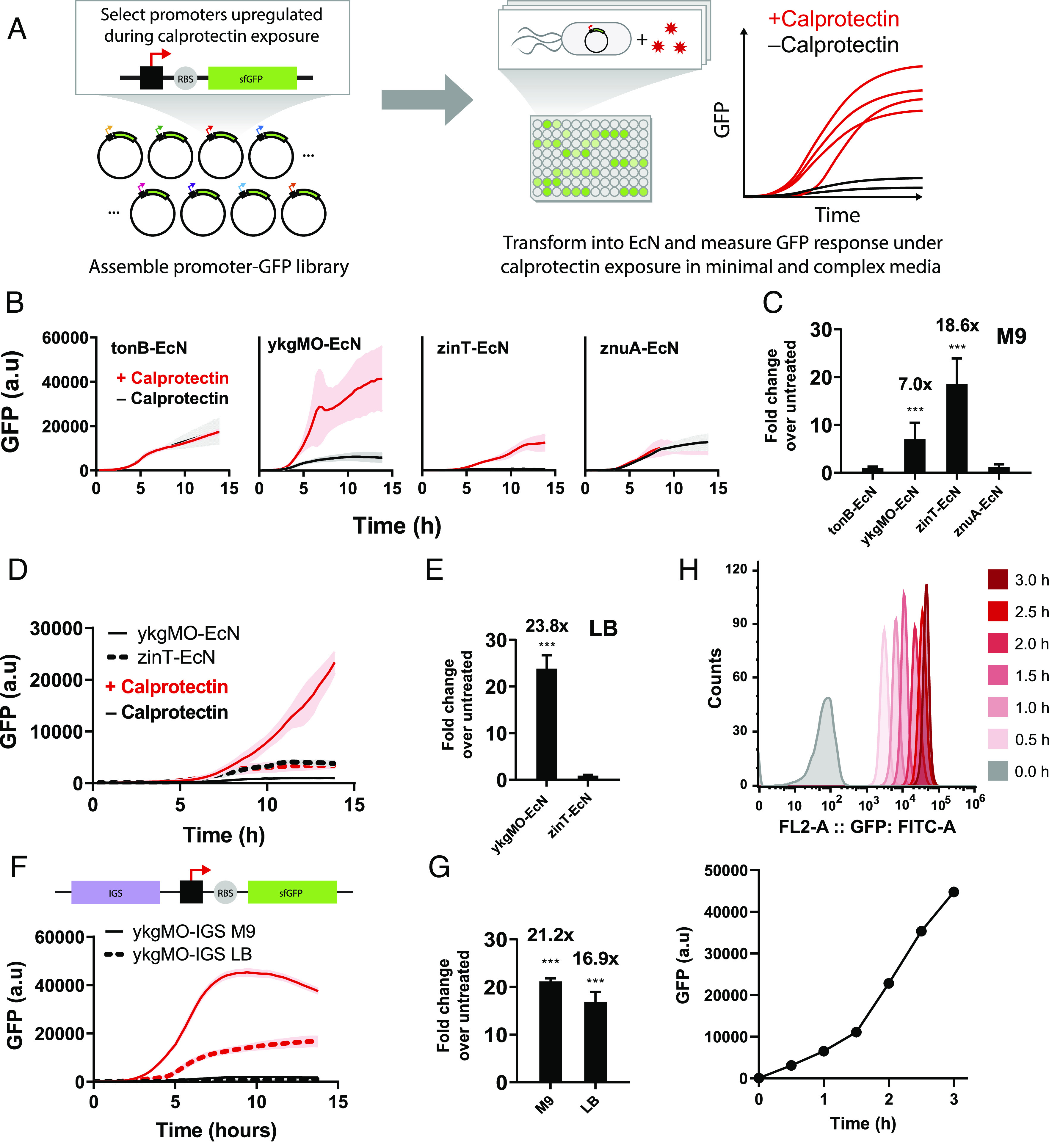
Identification of the EcN promoter ykgMO as a reliable sensor of calprotectin in both LB and M9 media in vitro. (*A*) Schematic of the screening process used to test the sensitivity of calprotectin-responsive promoters identified from the RNA-seq data. (*B*) Four strains of recombinant EcN were generated using the promoters of the top four calprotectin-sensitive genes cloned into GFP expression vectors. GFP expression was quantified by the TECAN infinite plate reader after each strain (n = 3) was treated with 100 μg/g of calprotectin for 13.8 h in M9 media. (*C*) Fold-change of GFP expression of calprotectin-treated strains over untreated. (*D*) GFP expression was quantified by the TECAN infinite plate reader after recombinant EcN with GFP expression vectors containing ykgMO (n = 3) and zinT (n = 3) promoters were treated with 100 μg/g of calprotectin for 13.8 h in LB media. (*E*) Fold-change of GFP expression of calprotectin-treated strains over untreated. (*F*) GFP expression was quantified by the TECAN infinite plate reader after recombinant EcN with GFP expression vectors containing ykgMO-IGS promoter (n = 3) were treated with 100 μg/g of calprotectin for 13.8 h in M9 and LB media. (*G*) Fold-change of GFP expression of calprotectin-treated strains over untreated. (*H*) A representative flow cytometry image of ykgMO-IGS EcN from 1 to 3 h of treatment with 100 μg/g of calprotectin with correcting graph of GFP intensities after calprotectin treatment. **P < 0.01 and ***P < 0.001 for Student’s unpaired *t* test for indicated comparisons. Data are represented as mean ± SEM.

To identify potential biosensors for calprotectin, the respective promoters of the five most significantly up-regulated genes were coupled to super folder green-fluorescent reporter protein (sfGFP) prior to transformation into EcN. These recombinant strains of EcN were then treated with 100 µg/g calprotectin to assess for the sensitivity of these promoters to calprotectin using sfGFP fluorescence intensity as a surrogate marker (Fig. 2*A*). We initially screened promoters of the top up-regulated calprotectin-responsive genes *ykgM*, *ykgO*, *zinT*, *znuA*, and *tonB* which are all induced in the setting of zinc starvation (24). Interestingly, the expression of these genes is regulated by transcription factors from the ferric uptake regulator family that are responsible for cellular homeostasis of metal ions including manganese, nickel, zinc, and iron (25). The promoters of *ykgMO*, *zinT*, and *znuA* are all under the control of the transcriptional repressor zinc uptake regulator (Zur), while the promoter of *tonB* is regulated by the transcription factor ferric uptake regulator (Fur) (24, 25). These results are consistent with an expected mode of action for calprotectin sensing involving zinc chelation leading to metal starvation.

We first evaluated the responses of biosensors constructed from these promoters in minimal media (M9) with the goal of identifying promoters with high sensitivity to calprotectin and low background induction during the growth phase. EcN transformed with plasmid-containing promoters ykgMO (ykgMO-EcN) and zinT (zinT-EcN) demonstrated calprotectin-dependent activation of sfGFP fluorescence compared to untreated controls with similar growth patterns (Fig. 2*B* and *SI Appendix*, Fig. S2*A*). ZinT-EcN displayed an 18-fold fluorescence change above untreated controls after 13.8 h of calprotectin induction, while ykgMO-EcN exhibited a sevenfold change compared to untreated controls (Fig. 2*C*). The total sfGFP fluorescence of calprotectin-treated ykgMO-EcN at the end of the 13.8 h growth curve was approximately fourfold of zinT-EcN (Fig. 2*B*). However, untreated ykgMO-EcN exhibited 10-fold increase sfGFP fluorescence compared to its zinT-EcN counterpart, suggesting the presence of gene induction during normal cellular growth (Fig. 2*B*). EcN transformed with plasmid-containing promoters for znuA and tonB did not show significant increases in fluorescence compared to untreated controls upon calprotectin treatment. (Fig. 2*B*). In addition, total sfGFP fluorescence of znuA-EcN and tonB-EcN during the growth curve study was elevated to a comparable degree as zinT-EcN, suggesting significant induction of these promoters during normal cellular growth (Fig. 2*B*). Thus, only ykgMO-EcN and zinT-EcN remained as viable candidates for our sensor.

Next, we evaluated whether calprotectin sensitivity in ykgMO-EcN and zinT-EcN was also observed in complex media conditions (LB). When treated with calprotectin in LB, zinT-EcN no longer exhibited its robust calprotectin-dependent activation of sfGFP (Fig. 2*D*), while ykgMO-EcN maintained its response with 23-fold increase sfGFP fluorescence compared to untreated control (Fig. 2*E*). Growth conditions were similar for all groups in this study (*SI Appendix*, Fig. S2*B*). Interestingly, untreated ykgMO-EcN did not exhibit a similar increase in sfGFP-induction compared to when grown in minimal media after 13.8 h. This effect is likely due to the lack of metal ion starvation–induced gene expression of ykgMO in complex media compared to minimal media conditions.

The intergenic region upstream of genes can contain a variety of functional and regulatory elements, including binding sites for transcription factors (26–28). In addition, the zinc-specific regulator, Zur, has been shown to bind to sites in the intergenic region upstream of known promoter sites of other zinc-responsive ribosomal proteins, like L31/L36 encoded by the ykgMO promoter (29). Thus, to improve the stability and reduce background expression levels of the ykgMO promoter, the entire intergenic region upstream of the *ykgMO* gene was identified and coupled with sfGFP to produce a new strain of calprotectin sensing EcN (ykgMO-IGS EcN). This new strain exhibited a 68% reduction in background sfGFP expression level when untreated in minimal media, while maintaining similar induction when treated with calprotectin (Fig. 2*F* and *SI Appendix*, Fig. S2*C*). Sensitivity to calprotectin in complex media for ykgMO-IGS-EcN was comparable to calprotectin-treated ykgMO-ECN (Fig. 2*G*). Growth conditions were similar for all groups in these experiments (*SI Appendix*, Fig. S2*D*). Changes in fluorescence can be detected as early as 1 h posttreatment of calprotectin in ykgMO-IGS EcN compared to untreated (Fig. 2*H*). We also performed a dose–response curve using ykgMO-IGS-EcN strain with varying doses of calprotectin. We found that the lower limit of detection was 25 µg/g of calprotectin and maximum sfGFP signal increased with higher doses of calprotectin (*SI Appendix*, Fig. S2*E*). Taken together, our results show that ykgMO-IGS-EcN is a viable candidate for in vivo sensing of calprotectin.

### Calprotectin-Sensing EcN Can Reliably Detect Gut Mucosal Inflammation In Vivo.

To test whether our calprotectin sensor detects gut inflammation in vivo, the ykgMO-IGS promoter was coupled with luxCDABE cassette and transformed to EcN to generate a new inducible bioluminescent calprotectin-sensing EcN (CS EcN-Lux). This strain of EcN is capable of endogenous production of bacterial luciferin and luciferase to generate a luminescent signal on whole-animal imaging when the ykgMO-IGS promoter is induced (6). CS EcN-Lux demonstrated robust calprotectin-dependent activation of luminescence compared to untreated controls with similar growth patterns (*SI Appendix*, Fig. S3 *A* and *B*). The maximum luminescence of calprotectin-treated CS EcN-Lux was 1,830 fold over untreated controls (*SI Appendix*, Fig. S3*C*). The lower limit of detection of CS EcN-Lux is the same as previous experiments from ykgMO-IGS-EcN strain at 25 µg/g of calprotectin (*SI Appendix*, Fig. S3 *D* and *E*).

**Fig. 3.**
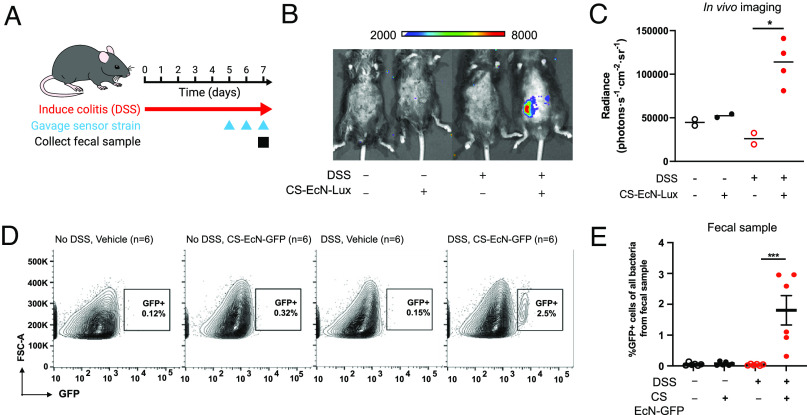
Calprotectin-sensing EcN can reliably detect gut mucosal inflammation in vivo in the DSS-induced colitis mouse model. (*A*) Mice were gavaged daily with 3% DSS for 7 d to induce colitis. CS EcN-Lux or CS EcN-GFP was gavaged daily for 3 d prior to IVIS imaging or fecal sample collection. (*B*) Representative live animal luminescence imaging was performed using IVIS Spectrum Instrument on mice (n = 4) that were gavaged CS EcN-Lux compared to controls that were not treated with DSS or not gavaged CS EcN-Lux (n = 2). (*C*) Luminescence was quantified for live animal imaging of mice gavaged with CS EcN-Lux with colitis induced by DSS (n = 4) and controls (n = 2). (*D*) Representative flow cytometry images quantifying GFP positive cell populations in mice that were treated with DSS and gavaged CS EcN-GFP and controls. Carbenicillin was given 2 d prior to bacterial gavage. (*E*) GFP expression was quantified by flow cytometry from stool of mice gavaged with CS EcN-GFP with colitis induced by DSS (n = 6) and controls (n = 6). **P < 0.01 and ***P < 0.001 for Student’s unpaired *t* test for indicated comparisons. Data are represented as mean ± SEM.

To model IBD in mice, we utilized the dextran sulfate solution (DSS)-induced colitis model where mice were given an oral gavage of 3% DSS daily for 7 d (Fig. 3*A*) (30). We chose the DSS-induced colitis model mainly due its ability to rapidly induce colitis phenotype and also extensive evidence demonstrating that DSS treatment resulted in elevation of fecal calprotectin levels (31–33). Starting on day 5, we orally gavaged the control and DSS-treated groups with either 1 × 10^9^ CS EcN-Lux or EcN without sensor daily (Fig. 3*A*). In vivo luminescence imaging was performed using the in vivo imaging system (IVIS) on day 7. Luminescent signals were detected in DSS-treated mice that were gavaged with CS EcN-Lux with signals reaching 1.13 × 10^5^ radiance (photons s^−1^ cm^−2^ sr^−1^) that was significantly increased compared to those measured from mice not treated with DSS and not gavaged CS EcN-Lux (Fig. 3*B* and *SI Appendix*, Fig. S3*F*). The mouse colon collected after 7 d of DSS treatment had altered colonic architecture, goblet cell loss, and immune cell infiltration compared to controls, confirming the presence of gut inflammation (*SI Appendix*, Fig. S3*G*). Serum and stool calprotectin levels were significantly elevated in DSS-treated mice as well (*SI Appendix*, Fig. S3 *H* and *I*), further confirming that luminescent signals are corresponding elevated calprotectin and gut inflammation caused by DSS treatment.

To further demonstrate that our calprotectin sensor is capable of functioning in the complex environment of the mammalian gut, we utilized the previously created ykgMO-IGS-sfGFP reporter strain transformed into EcN, termed CS EcN-GFP, for further in vivo studies. Mice were treated with 3% DSS daily and were gavaged with either 1 × 10^9^ CS EcN-GFP or EcN without sensor at days 5, 6, and 7 of DSS treatment. Stool samples and mouse colons were collected on day 7 (Fig. 3*A*). Although EcN is a known colonizer of the human gastrointestinal tract, it is a poor colonizer of the mouse gut. Past studies have shown that EcN can persist transiently in mice pretreated with antibiotics to reduce local competition (34). Therefore, carbenicillin was given 2 d prior to bacterial gavage to allow CS EcN-GFP to reach high density. Flow cytometry analysis of stool samples of mice treated with DSS and gavaged with CS EcN-GFP revealed the presence of a distinct sfGFP-positive population of bacteria (Fig. 3*D*). Importantly, this sfGFP-positive population was not seen in the absence of DSS or in mice that were not given CS EcN-GFP (Fig. 3 *E* and *F*). Furthermore, DSS-treated mice that were fed CS EcN-GFP had ~26-fold increase in this sfGFP-positive bacterial population in stool seen on flow cytometry compared to non-DSS-treated mice that were also fed CS EcN-GFP (*SI Appendix*, Fig. S3*J*). This demonstrates that CS EcN-GFP is highly specific for calprotectin even in a highly heterogeneous environment such as the mouse colon. Thus, both sensor strains are activated in response to gut inflammation in the mouse colon, and this signal can be detected in stool samples.

### Calprotectin-Sensing EcN Can Differentiate Stool Samples from Patients with Active IBD and Those in Remission.

**Fig. 4.**
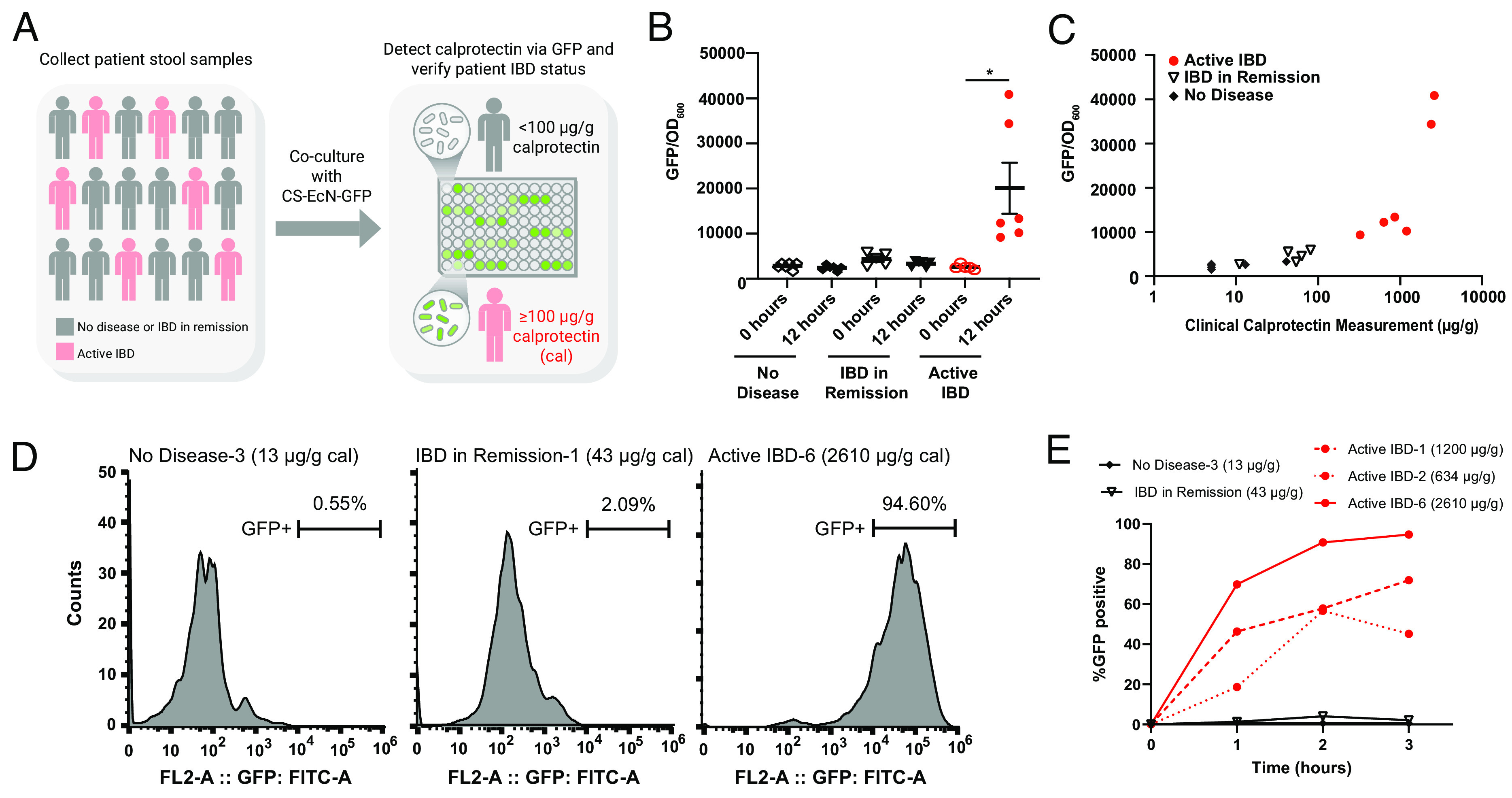
Calprotectin-sensing EcN can differentiate stool samples from patients with active IBD and those in remission. (*A*) Stool samples were collected from patients with active IBD (n = 6), IBD in remission (n = 5), and no IBD (n = 6). CS EcN-GFP was cocultured with stool samples and GFP was quantified using the TECAN infinite plate reader after 12 h. (*B*) GFP/OD_600_ of CS EcN-GFP cocultured with fecal samples of patients with active IBD (n = 6), IBD in remission (n = 5), and no IBD (n = 6). GFP/OD_600_ was quantified by the TECAN infinite plate reader immediately after coculturing and 12 h after coculturing. (*C*) Laboratory-measured calprotectin values obtained from electronic medical records retrospectively of patients with active IBD (n = 6), IBD in remission (n = 5), and no IBD (n = 6) plotted with their respective GFP/OD_600_ intensities. (*D*) Representative flow cytometry images quantifying GFP positive cell populations in CS EcN-GFP that were cocultured with fecal samples of patients with no IBD, IBD in remission, and active IBD after 3 h. (*E*) %GFP-positive cells from flow cytometry time course of CS EcN-GFP after coculturing with patient samples. *P < 0.05 for Student’s unpaired *t* test for indicated comparisons. Data are represented as mean ± SEM.

To evaluate whether our CS EcN-GFP can identify patients with active IBD, we collected fecal samples from patients undergoing stool studies either at the emergency department, in the hospital, and in outpatient clinic settings (Fig. 4*A*). Stool specimens were collected from 17 patients that consisted of 11 patients with a history of IBD and 6 with no history of IBD (Table 1). Of those 11 patients with IBD, four had a history of Crohn’s disease, and seven had a history of ulcerative colitis; fecal calprotectin levels were ordered for these patients for the indication of disease surveillance, therapeutic drug monitoring, or suspicion of acute IBD flare. For IBD medications, patients in the active IBD and IBD in remission groups were on a mix of therapy ranging from oral 5-aminosalicyclic acid to intravenous biologics. Of note, many of the patients also were taking oral multivitamin at the time of stool sample collection which includes 5 to 10 mg of zinc supplementation. Clinical laboratory–quantified calprotectin levels obtained retrospectively from electronic medical records showed that six patients had active IBD with calprotectin ranging from 325 to 2610 µg/g (Table 1), while those with inactive disease had calprotectin levels ranging from 11 to 81 µg/g. Of the patients with no IBD history, fecal calprotectin levels were evaluated for indications of diarrhea, rectal bleeding, or abdominal pain, with laboratory results of calprotectin levels being <50 µg/g for all specimens. This small cohort will therefore allow us to gather proof-of-principle data for using CS EcN-GFP to discriminate stool samples from patients with active IBD, IBD in remission, and healthy controls.

**Table 1.** Patient demographics

	Sex	Age	Laboratory measured calprotectin	IBD type	IBD medication	Multi-vitamin on medication list
No disease-1	M	54	<5	NA	NA	yes
No disease-2	M	41	41	NA	NA	yes
No disease-3	F	27	13	NA	NA	no
No disease-4	F	30	<5	NA	NA	yes
No disease-5	F	42	<5	NA	NA	yes
No disease-6	F	37	<5	NA	NA	no
IBD in remission-1	M	67	43	Ulcerative pancolitis	Mesalamine	no
IBD in remission-2	F	53	81	Ileocolonic Crohn’s disease	Certolizumab	no
IBD in remission-3	F	46	63	UC with left sided colitis	Vedolizumab	yes
IBD in remission-4	M	23	54	Ileal Crohn’s disease	Ustekinumab	no
IBD in remission-5	M	48	11	Unspecified UC	6-MP, Mesalamine	no
Active IBD-1	M	33	1,200	Ulcerative proctitis	Mesalamine	no
Active IBD-2	F	58	634	UC with left sided colitis	Vedolizumab	no
Active IBD-3	F	52	325	Ulcerative proctosigmoiditis	Mesalamine, Infliximab	no
Active IBD-4	F	44	858	Ileocolonic CD	Ustekinumab	yes
Active IBD-5	F	48	2,390	Crohn’s Colitis	Mesalamine	no
Active IBD-6	F	33	2,610	Ulcerative pancolitis	Infliximab	yes

To determine whether CS EcN-GFP is activated in response to stool with elevated levels of calprotectin, we cocultured CS EcN-GFP with stool samples from patients with no IBD, IBD in remission, and active IBD (Fig. 4*A*). Immediately after coculture, sfGFP fluorescence normalized to optical density at 600 mm (OD_600_) was comparable for the samples from patients with no IBD, IBD in remission, and active IBD (Fig. 4*B*). After 12 h of coculturing, CS EcN-GFP cocultured with fecal samples from patients with active IBD had significantly increased sfGFP/OD_600_ ratios compared to those that were cultured with samples from patients without IBD or IBD in remission (Fig. 4*B*). Of the patients with active disease, those with the highest levels of calprotectin also had higher sfGFP/OD_600_ ratios when cocultured with EcN-lux exhibiting an overall positive correlation with fold changes ranging from approximately 5 to 15 when compared to healthy controls (Fig. 4*C*). There was no significant difference between sfGFP/OD_600_ ratios when comparing patients with no IBD history and those with disease in remission (Fig. 4*B*). Flow cytometry of CS EcN-GFP cocultured with fecal samples from patients with active disease confirmed the presence of a distinct sfGFP-positive population of bacteria as early as 1 h of coculturing and saturation of sfGFP-positive populations can be seen with stool sample from patients with higher levels of laboratory-measured calprotectin by 3 h of coculturing (Fig. 4 *D* and *E* and *SI Appendix*, Fig. S4 *A*–*C*). In addition, there was a faster rate of activation of sfGFP-positive populations for stool samples from patients with active IBD that had higher laboratory-measured calprotectin levels (Fig. 4*E* and *SI Appendix*, Fig. S4 *E* and *F* and Table S1). Overall, this validates that CS EcN-GFP can effectively sense elevated calprotectin in human stool samples, and it has the potential to be used as a reliable and efficient method of disease activity surveillance in IBD patients.

## Discussion

**Fig. 5.**
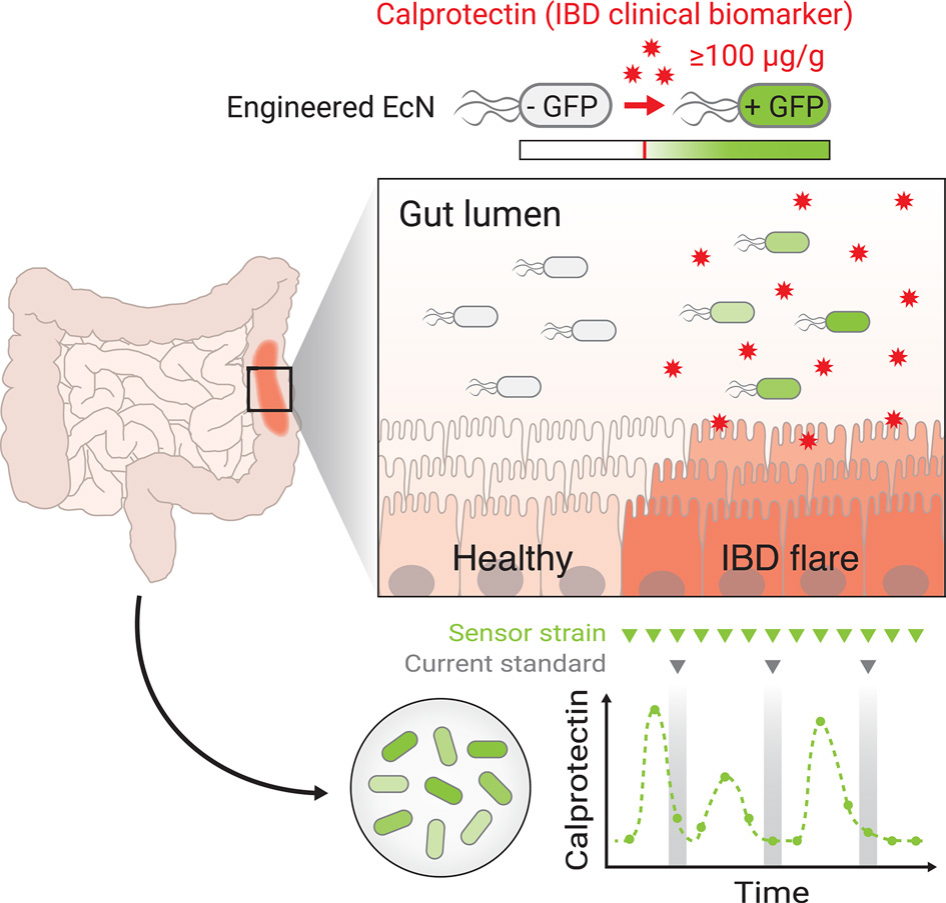
A schematic of calprotectin sensing EcN that can be used as a timely, reliable, and noninvasive method of disease activity monitoring in IBD.

We have developed an engineered probiotic that can reliably detect calprotectin, the clinical gold standard biomarker of noninvasive gut inflammation, with sensitivity and specificity for the purpose of disease activity monitoring in IBD. Oral delivery of CS EcN-GFP resulted in the presence of a distinct sfGFP+ bacterial population in stool of mice with DSS-induced colitis. These sfGFP+ bacteria were not present in mice that were either not treated with DSS or not given an oral gavage of CS EcN-GFP, suggesting that the activation of our sensor is specific to gut inflammation with low basal activity. Furthermore, oral delivery of CS EcN-Lux resulted in a significantly increased luminescence signal in mice treated with DSS that can be seen in live imaging with IVIS. Consistent with our GFP-reporter model, there was minimal leakage of luminescence signal seen in mice not treated with DSS or not gavaged with sensor. Our data from clinical stool samples showed that sensor activation was only present when cocultured with stool from patients with active IBD. Importantly, sfGFP intensity of our sensor can quantitively track with the laboratory-measured levels of fecal calprotectin. Minimal nonspecific sensor activation was observed in this heterogeneous population of IBD patients where diet and medications were not controlled. In addition, many of the patients both with and without IBD were taking daily multivitamin tablets which usually contain 5 to 10 mg of zinc supplementation. Interestingly, this did not appear to affect the readout of our sensor as zinc is absorbed in the small bowel. Overall, our data suggest the feasibility of our engineered calprotectin sensing probiotic as a method of long-term, noninvasive monitoring of disease activity in IBD that would improve early detection of IBD relapses and clinical outcomes by reducing hospitalizations (Fig. 5).

Several gut inflammation whole-cell biosensors have been developed previously to detect nitrous oxide and tetrathionate as surrogate markers of gut inflammation. However, these targets were nonspecific to IBD and have not been rigorously studied in the clinical setting as potential biomarkers for disease activity monitoring in IBD (9–11). Our model is geared to detect the current clinical gold standard of noninvasive disease activity monitoring for IBD, calprotectin, per the most recent clinical guidelines from the American College of Gastroenterology and quantitatively tracks with the clinical laboratory results obtained from Northwestern Memorial Hospital Clinical Pathology Laboratory (35, 36). In addition, the major advantages of our platform compared to conventional enzyme-based ELISA assays of calprotectin include simpler data analysis and on-demand assessment of samples as it avoids the extraction step that is required for all ELISA-based assays. Specialized lab equipment is not required to develop our sensors or run the reactions as it is culture and plate reader based.

The incidence and prevalence of IBD have been increasing at a global level, particularly in developing and newly industrialized nations with historically low rates (14, 37). In addition, accurate detection of relapsing disease is often delayed in these nations due to lack of reliable diagnostic facilities, particularly endoscopy and pathology (38, 39). Our engineered calprotectin probiotic could play a key role in these patient populations as it offers a noninvasive, easy-to-use method of semiquantitative determination of IBD disease activity that requires a simple fluorescent or luminescent readout. Offering accurate disease monitoring will result in appropriate risk stratification of patients and optimization of medical management as we strive to bridge the gap in health care disparity in these patient populations.

## Methods

### RNA Isolation.

EcN single colonies were grown overnight in either M9 or LB media. Cells were then diluted 1:100 into 5 mL of M9 or LB media and grown to log phase. Cell cultures were then either incubated with 100 μg/mL of calprotectin or remained untreated for 30 min. RNA was then isolated using the QIAGEN RNeasy kit (QIAGEN) according to the manufacturer’s instructions.

### RNA-seq.

RNA quality was checked using the Bioanalyzer (Agilent) prior to RNA-seq library preparation. RNAs with an RNA integrity number >8 were used for library preparation, which was constructed from 100 ng of RNA with the Illumina Stranded Total RNA Prep, Ligation with Ribo-Zero Plus kit (Illumina). RNA-seq was then performed on the NovaSeq 6000 sequencer and analyzed as previously described. Reads were aligned to the GCF_003546975.1 assembly of the *E. coli Nissle* 1917 reference genome with STAR (v2.7.5) using the GeneCounts option. Gene counts were obtained from EcN annotations (Refseq) using rsem-calculate-expression (v.1.3.0). Differential expression between treatments and media conditions using DESeq2 (v1.32.0) was analyzed after removal of genes with <10 assigned counts. FDR-adjusted *P*-value cutoff for significance was set to 0.05. Plots were made in R (v4.0.3) using ggplot2 (v3.3.0), pheatmap (v1.0.12). Annotations were converted to gene IDs using EcoCyc (www.ecocyc.org). Pathway and biological function assignments of differentially expressed genes were completed using DAVID bioinformatics tools (https://david.ncifcrf.gov) on all statistically significant differentially expressed genes (*P* < 0.05) in EcN treated with calprotectin and untreated controls in both LB and M9 media.

### DNA Cloning.

Custom promoter sequences were ordered from Integrated DNA Technologies and cloned upstream of sfGFP reporter or luxCDABE cassette in a P15A backbone with chloramphenicol resistance. All plasmid assembly was performed using the Gibson Assembly using the Gibson Assembly Master Mix (NEB). The assembled plasmid was transformed into chemically competent EcN as previously described and plated onto LB agar with appropriate antibiotics (6).

### Calprotectin Induction.

Recombinant human calprotectin was supplied by Walter Chazin of Vanderbilt University. EcN-containing sensor plasmids were grown overnight, diluted 1:100 in either M9 or LB, and then treated with 100 μg/mL of calprotectin. SfGFP fluorescence and OD_600_ were recorded for 12 h in the TECAN infinite plate reader. Luminescence imaging was quantified using a BioTek Synergy Neo2 Multi-Mode Plate Reader.

### Dextran Sodium Sulfate Mouse Experiments.

Colitis was induced in 6- to 8-wk-old male C57BL/6 mice by administration of 3% (W/V) DSS (MW ~ 40,000, Sigma) in drinking water for 7 d. On day 3 of DSS, mice were given an oral gavage of carbenicillin to promote engraftment of the probiotic in the mouse gut. On days 5, 6, and 7 of DSS, mice were given an oral gavage of 1 × 10^9^ CFUs of CS EcN-Lux/CS EcN-GFP or EcN without sensor. Six hours after probiotic sensor gavage on day 7 of DSS, mice were euthanized, and stool from the proximal and distal colon was collected for sfGFP analysis.

### Histology.

Colon tissue of DSS-treated and untreated mice was fixed in 4% paraformaldehyde for 24 h before transfer to 70% ethanol. Paraffin‐embedding, sectioning, and hematoxylin and eosin staining were performed by Northwestern University Research Histology and Phenotyping Laboratory Core.

### Mouse Fecal Sample Preparation.

Mouse stool collected from the colon was homogenized in 1.5 mL of sterile PBS. Samples were then vortexed and filtered through a 5-μm syringe filter (Millipore) to remove solid components and host cells. Filtered samples were incubated for 1 h in 37 Celsius to allow for maturation of fluorophores and then analyzed by flow cytometry.

### Human Stool Sample Collection and Preparation.

0.5 to 1 g of the stool sample was collected from stool samples of patients in the emergency department, outpatient clinic, and inpatient unit where fecal calprotectin was ordered as part of stool studies testing from 2021 to 2022 at Northwestern Memorial Hospital under the Institutional Review Board approved study STU00216870. Samples were collected only when excess stool samples were available after all necessary clinical laboratory testing was completed. No extra samples were requested from patients. All biospecimens were deidentified and labeled with a unique subject code that did not contain any personal identifiers.

For coculturing experiments, we homogenized ~0.1 g of the human stool sample with sterile PBS to make a final concentration of 1 mg/μL. If the stool was liquid, homogenization was not done, and 100 μL of the liquid stool sample was used instead. This mixture was then cocultured with 2 mL of 1:100 diluted CS EcN in the log phase in M9 media for 12 h. A 200 μL aliquot of this mixture was then quantified in the TECAN infinite plate reader for OD600 and sfGFP with excitation/emission wavelength set to 485/530 nm and gain set to 100.

### Calprotectin Quantification.

Measured calprotectin levels for the patient samples were performed by the Northwestern Clinical Pathology Laboratory. During 2021 to 2022 when the samples were collected, calprotectin was a send out lab to Quest Diagnostics and quantification was performed at Quest Nichols Institute in Chantilly, VA. Quest Diagnostics utilizes an FDA-approved chemiluminescence method for calprotectin detection requiring at least 0.3 g of the stool sample that has been in storage at room temperature for less than 6 d. No preservatives were used in storing of samples after collection. Calprotectin levels were obtained retrospectively through electronic medical records.

Mouse Calprotectin levels were quantified using ELISA (Immundiagnostik) from serum and stool collected from mice after 7 d of treatment with 3% DSS.

### Flow Cytometry.

Flow cytometry was performed using a Sony SH800 cytometer using a standard 408 nm laser configuration and a 100-μm sorting chip. Cells were gated on FSC-A and SSC-A to exclude debris followed by FSC-H and FSC-W to isolate single cells. For pure EcN culture experiments, cells were treated with calprotectin for 1 h prior to analysis. For human stool experiments, 1 mL of CS EcN-GFP grown to the log phase was cocultured with human stool samples for 1 to 3 h prior to analysis. Flow cytometry plots were generated with FlowJo (Becton, Dickinson & Company).

### Fluorescence and Luminescence Imaging.

SfGFP fluorescence in all our studies was measured using a TECAN infinite MPLEX plate reader with excitation/emission wavelength set to 485/530 nm and gain set to 100. Luminescence imaging was quantified using a BioTek Synergy Neo2 Multi-Mode Plate Reader with gain set to 135.

### Mouse Experiments.

All mice were maintained on a C57BL/6J background. Wild-type C57BL/6J mice were obtained from Jackson Laboratories. Mice were maintained at room temperature with a 12-h light/dark cycle and free access to water and food. All animal experiments were performed according to procedures approved by the Northwestern University Institutional Animal Care and Use Committee.

### IVIS Imaging.

To image in vivo bacterial luminescence, mice were shaved and anesthetized with isoflurane and imaged using IVIS Spectrum Instrument (Perkin Elmer). The bacteria LuxCDABE cassette produced a luminescent signal without provision of an exogenous substrate. Quantification of luminescence was done using the Living Image Analysis Software (Perkin Elmer).

### Statistical Analyses.

Statistical tests were calculated in either Microsoft Excel (Student’s *t* test) or GraphPad Prism 9.0 (ANOVA, Student’s *t* test). Differences between the two groups over time were determined by a two-way repeated measures ANOVA. For comparisons between two independent groups, a Student’s *t* test was used. Significance was accepted at *P* < 0.05. The details of the statistical tests carried out are indicated in respective figure legends.

## Supplementary Material

Appendix 01 (PDF)

Dataset S01 (PDF)

## Data Availability

RNA-seq raw data have been deposited in GEO repository (GSE211418) (40). All other data are included in the article and/or SI Appendix.
