# Supplementary material for: Engineered calprotectin-sensing probiotics for IBD surveillance in humans
Source: Proc Natl Acad Sci U S A. 2023 Jul 31;120(32):e2221121120. doi: 10.1073/pnas.2221121120 (PMC10410751; doi:10.1073/pnas.2221121120)
Supplement: Supplementary file 1 — Appendix 01 (PDF) [file pnas.2221121120.sapp.pdf]

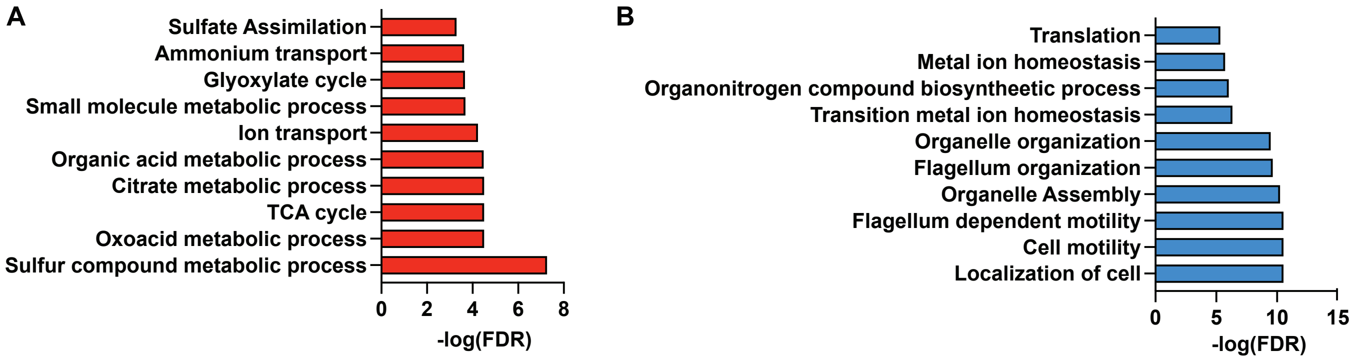

Supplemental Fig. 1. Gene ontology (GO) analysis indicated enrichment of EcN genes in pathways involved in ion transport, cellular metabolic processes, and cell motility after calprotectin treatment.
(A) Gene ontology analysis of statistically significant upregulated EcN genes with at least 2 times fold change after calprotectin treatment compared to controls. **(B)** Gene ontology analysis of statistically significant downregulated EcN genes with at least 2 times fold change after calprotectin treatment

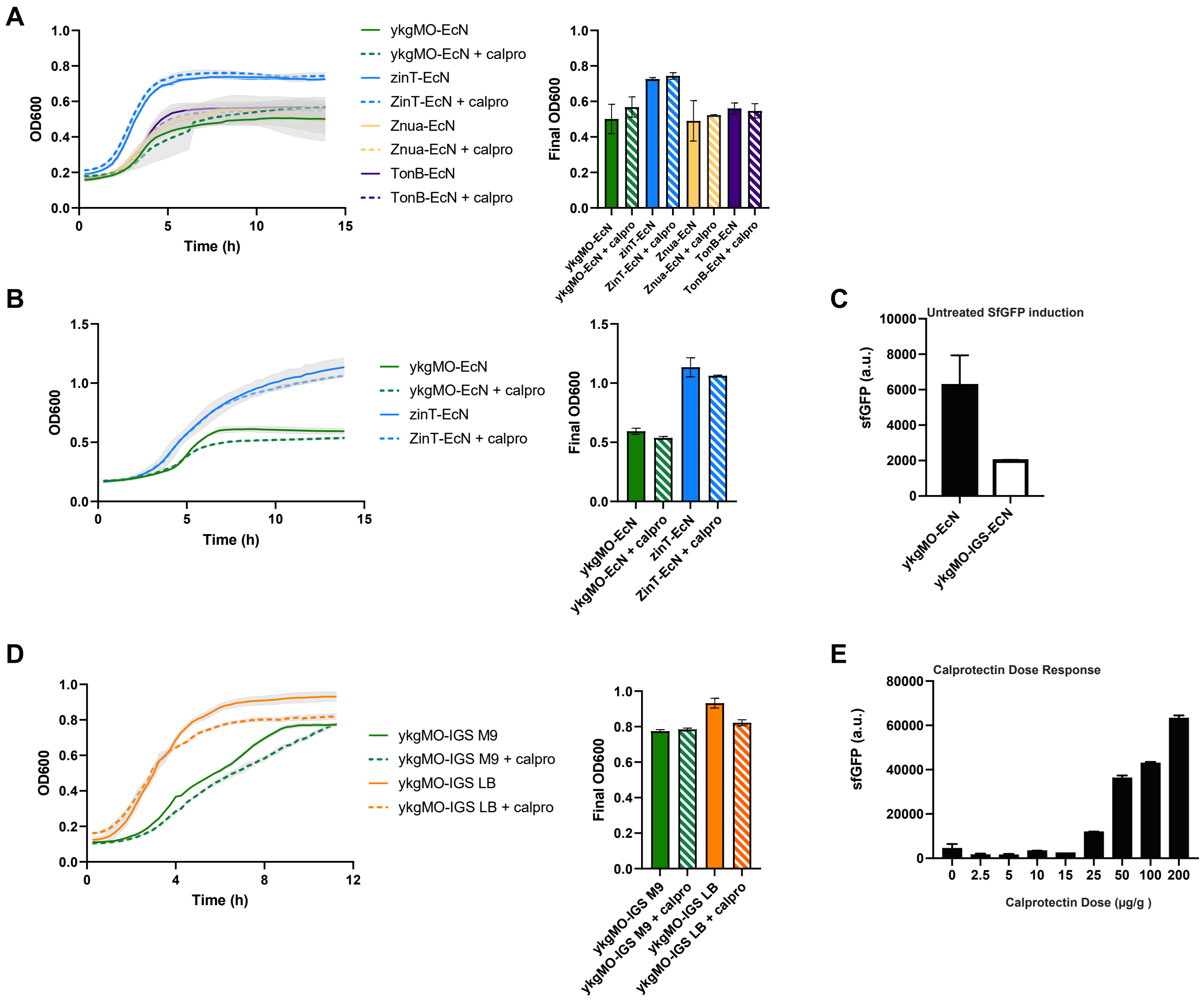

Supplemental Fig. 2. EcN transformed with plasmids containing ykgMO, zinT, tonB, znuA, and ykgMO-IGS displayed similar growth patterns in the setting of calprotectin treatment in both M9 and LB media.

(A) OD600 measurements during 13.8 hour growth curve and final OD600 at the end of growth curve for EcN transformed with plasmids containing ykgMO, zinT, tonB, znuA promoters with and without calprotectin in M9 media. **(B)** OD600 measurements during 13.8 hour growth curve and final OD600 at the end of growth curve for EcN transformed with plasmids containing ykgMO and zinT promoters with and without calprotectin in LB media. **(C)** Maximum sfGFP induction from ykgMO-EcN and ykgMO-IGS-EcN when untreated grown in M9 media. **(D)** OD600 measurements during 13.8 hour growth curve and final OD600 at the end of growth curve for EcN transformed with plasmids containing ykgMO-IGS promoter with and without calprotectin in both M9 and LB media. **(E)** Calprotectin dose response curve of ykgMO-IGS-EcN strain with varying doses of calprotectin grown in M9 media.

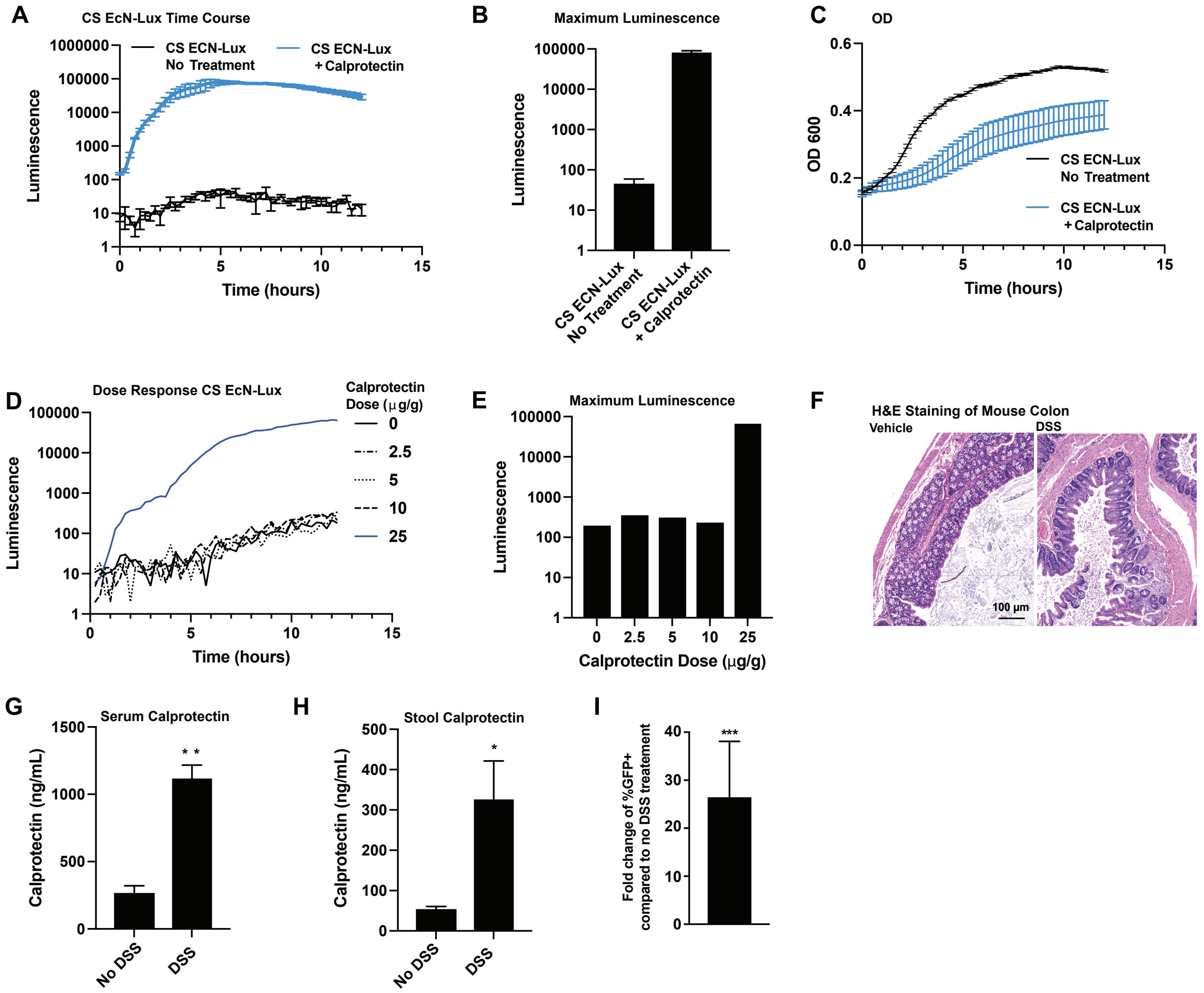

Supplemental Fig. 3. Construction and validation of CS EcN-Lux for in vivo inflammation detection in DSS treated mice.

(A) Luminescence was quantified by a BioTek Synergy Neo2 Multi-mode plate reader after CS EcN-Lux (n=3) was treated with 100 µg/g of calprotectin for 12.25 hours in M9 media. **(B)** Maximum Luminescence achieved by CS EcN-Lux after 100 µg/g of calprotectin compared to untreated controls during 12.25 hour growth curve in M9 media. **(C)** OD600 measurements during 12.25 hour growth curve for CS EcN-Lux (n=3) and CS EcN-Lux (n=3) treated with 100 µg/g of calprotectin in M9 media. **(D)** Calprotectin dose response curve of CS EcN-Lux strain with varying doses of calprotectin grown in M9 media. **(E)** Maximum Luminescence achieved by CS EcN-Lux during varying doses of calprotectin grown in M9 media. **(F)** Representative H&E staining of colons of mice treated with DSS and controls. **(G-H)** Serum and stool calprotectin levels of mice treated with DSS (n=5) and untreated controls (n=5). **(I)** Fold change of GFP positive cells present in stool of DSS-treated and control mice that were both gavaged with CS EcN-GFP.

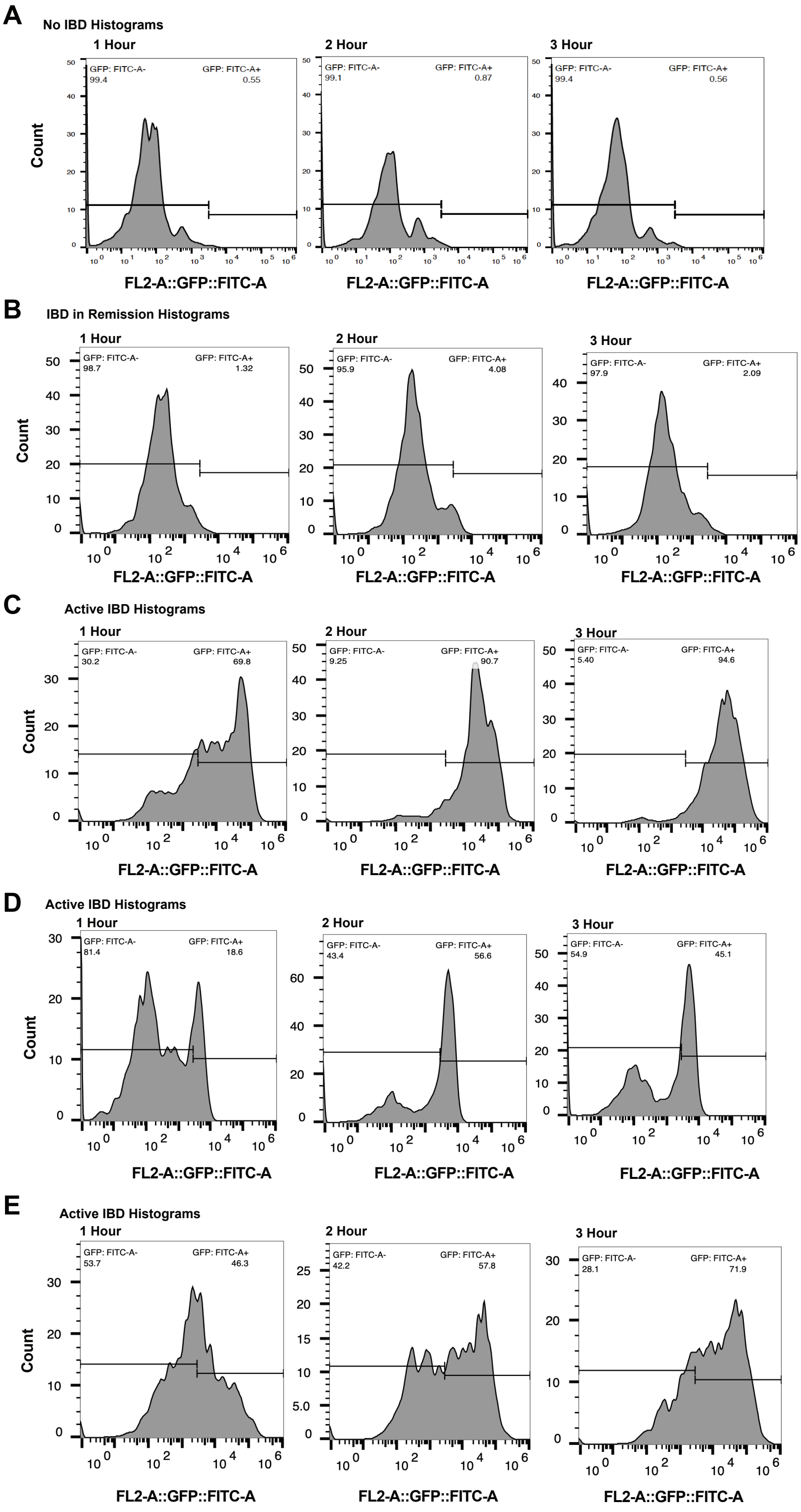

Supplemental Fig. 4. Time course analysis of flow cytometry of human stool samples co-cultured with EcN-GFP.

(A-E) Flow cytometry images quantifying GFP positive cell populations in CS EcN-GFP that were co-cultured with fecal samples of patients with no IBD, IBD in remission, and active IBD after 1, 2, and 3 hours.

Supplemental Table 1: Statistically Differentially Expressed Genes M9

Gene Description	baseMean	log2FoldChar	lfcSE	stat	padj
LSU ribosomal protein L31p @ LSU ribosomal protein L31p%2C zinc-independent	4159.9521	8.3312552	0.22599637	36.8645538	2.02E-294
LSU ribosomal protein L36p	1554.82149	7.8740522	0.22860914	34.4432955	4.47E-257
FIG00638682: hypothetical protein	25967.6907	6.29642581	0.11411961	55.1739159	0
Putative metal chaperone%2C involved in Zn homeostasis%2C GTPase of COG0523 family	9273.1339	6.27532017	0.12902424	48.6367542	0
membrane%3B Transport of small molecules: Cations	22974.0827	6.02893123	0.11835208	50.9406463	0
Candidate zinc-binding lipoprotein ZinT	13069.6575	5.97716781	0.17287756	34.5745736	5.76E-259
Zinc ABC transporter%2C periplasmic-binding protein ZnuA	12598.5021	4.53923021	0.22522956	20.1537946	1.07E-87
FIG00638093: hypothetical protein	2.89087556	3.55481229	1.37776426	2.580131	0.02321148
Alpha-ketoglutarate permease	657.254932	3.50966298	0.15970969	21.9752658	2.53E-104
Ornithine carbamoyltransferase	48486.8631	3.1397282	0.71084397	4.41690207	4.41E-05
Enoyl-CoA hydratase	354.42919	3.03978973	0.18249888	16.6564842	4.43E-60
FIG01045439: hypothetical protein	5903.77371	3.02131138	0.11626351	25.9867547	4.13E-146
Cell wall endopeptidase%2C family M23/M37	4553.34112	2.84757025	0.10523714	27.0586049	2.04E-158
Acetylglutamate kinase	17147.9007	2.75949496	0.48893753	5.64386001	1.01E-07
Argininosuccinate lyase	25827.4056	2.75849349	0.44733493	6.16650588	5.00E-09
L-2-hydroxyglutarate oxidase	670.483158	2.70879712	0.1524794	17.7650039	3.67E-68
gamma-aminobutyrate (GABA) permease	360.740592	2.70530055	0.23211585	11.6549581	7.76E-30
Uncharacterized GST-like protein yncG	18.8817548	2.61331432	0.54611683	4.78526604	8.38E-06
N-acetyl-gamma-glutamyl-phosphate reductase	14889.1258	2.57166721	0.51282599	5.01469746	2.76E-06
3-ketoacyl-CoA thiolase	359.070086	2.56377676	0.15924776	16.0992951	3.49E-56
Glycerol-3-phosphate ABC transporter%2C permease protein UgpA (TC 3.A.1.1.3)	83.0643008	2.5256736	0.24621442	10.2580246	2.48E-23
Biotin synthesis protein BioC	1289.76354	2.5035288	0.22607521	11.073876	4.79E-27
Zinc ABC transporter%2C inner membrane permease protein ZnuB	1749.13293	2.46077146	0.15580722	15.7936934	4.40E-54
Gamma-aminobutyrate:alpha-ketoglutarate aminotransferase	2265.85659	2.45062623	0.14005402	17.497722	2.94E-66
hypothetical protein	12.9929209	2.44502431	0.56971201	4.29168468	7.45E-05
hypothetical protein	60.5205562	2.4398318	0.26871669	9.07956928	1.81E-18
FIG00642236: hypothetical protein	39.3546132	2.43325895	0.3594961	6.76852677	1.13E-10
Succinate-semialdehyde dehydrogenase [NADP+]	2125.23673	2.42537769	0.13749907	17.63923	2.91E-67
Arginine ABC transporter%2C periplasmic arginine-binding protein ArtJ	18931.5814	2.42457143	0.56726009	4.27417944	8.02E-05
FIG00638909: hypothetical protein	204.639028	2.41927867	0.20973467	11.5349488	2.99E-29
Adenosylmethionine-8-amino-7-oxononanoate aminotransferase	1853.29017	2.38824604	0.37357288	6.39298551	1.26E-09
Glycerol-3-phosphate ABC transporter%2C permease protein UgpE (TC 3.A.1.1.3)	128.939216	2.36525379	0.19913166	11.8778387	5.94E-31
Phosphate starvation-inducible protein PhoH%2Cpredicted ATPase	61570.2327	2.31849404	0.21920568	10.5767972	9.31E-25
Lactate-responsive regulator LldR in Enterobacteria%2C GntR family	322.357714	2.31743476	0.1989355	11.6491764	8.24E-30
Arginine ABC transporter%2C permease protein ArtM	2227.75402	2.31736913	0.18052094	12.8371209	5.28E-36
Biotin synthase	1339.7991	2.3076781	0.23161401	9.96346498	4.65E-22
Arginine ABC transporter%2C permease protein ArtQ	3671.57437	2.29785426	0.16005795	14.3563896	7.79E-45
Acetyl-coenzyme A synthetase	1194.6021	2.2879276	0.14524013	15.7527234	8.19E-54
N-acetylglutamate synthase	6238.6552	2.27788742	0.45685253	4.98604534	3.18E-06
Dethiobiotin synthetase	718.414487	2.26007124	0.1928098	11.7217653	3.62E-30
aconitate hydratase	3934.90816	2.25488876	0.11717414	19.2439105	5.01E-80
Zinc ABC transporter%2C ATP-binding protein ZnuC	3765.81775	2.21225687	0.14948652	14.7990389	1.41E-47
Carbon starvation induced protein CsiD	299.49305	2.20914532	0.14958523	14.7684724	2.05E-47
putative protein PaaI%2C possibly involved in aromatic compounds catabolism	301.064824	2.18357964	0.17675121	12.3539728	2.17E-33
Nitrate/nitrite transporter	1028.59898	2.17547684	0.19574188	11.1140081	3.11E-27
Arginine N-succinyltransferase	177.240431	2.15628012	0.17364571	12.4176986	1.00E-33
Succinylglutamic semialdehyde dehydrogenase	368.976354	2.14284619	0.13304314	16.1064012	3.20E-56
Respiratory nitrate reductase beta chain	1141.26917	2.09270382	0.17792273	11.7618685	2.27E-30
L-lactate permease	412.007405	2.07994456	0.17857911	11.6471888	8.37E-30
Arginine ABC transporter%2C ATP-binding protein ArtP	3371.88701	2.06368388	0.18060039	11.4267964	9.96E-29
Respiratory nitrate reductase alpha chain	3188.60171	2.05736744	0.18716753	10.9921171	1.17E-26
Stage V sporulation protein involved in spore cortex synthesis (SpoVR)	7392.3733	2.05591422	0.13989267	14.6963688	5.85E-47
Argininosuccinate synthase	24521.9554	2.04264795	0.40166557	5.08544446	1.95E-06
Glycerol-3-phosphate ABC transporter%2C ATP-binding protein UgpC (TC 3.A.1.1.3)	1062.91148	2.03803907	0.14458576	14.095711	2.94E-43
Acetylornithine aminotransferase	3047.65923	2.02668277	0.46953003	4.31640717	6.69E-05
Respiratory nitrate reductase delta chain	451.462866	2.01650704	0.17316205	11.6452017	8.51E-30
UPF0229 protein YeaH	5606.61024	2.01597121	0.18256319	11.0425943	6.74E-27
FIG00638803: hypothetical protein	30.8688754	1.95284866	0.40544939	4.81650409	7.20E-06
Glycolate dehydrogenase	518.888175	1.9433716	0.14750735	13.1747444	6.99E-38
Succinylglutamate desuccinylase	204.099759	1.9386221	0.17483733	11.0881476	4.11E-27
Malate:quinone oxidoreductase	1933.89006	1.90891269	0.18082156	10.5568863	1.15E-24
Serine protein kinase (prkA protein)%2C P-loop containing	25603.7954	1.90382375	0.17891425	10.6409846	4.86E-25
Arginine ABC transporter%2C periplasmic arginine-binding protein ArtI	8730.75211	1.90332833	0.14880242	12.7909766	9.47E-36
Cold shock protein CspG	1632.47579	1.87577392	0.14127639	13.2773345	1.83E-38
8-amino-7-oxononanoate synthase	2315.43297	1.87036375	0.2045578	9.14344878	1.01E-18
Protein ygiW precursor	577.797212	1.85821061	0.18179045	10.2217172	3.54E-23
L-proline glycine betaine binding ABC transporter protein ProX (TC 3.A.1.12.1)	1589.38934	1.7859385	0.21262462	8.3994905	6.11E-16
Succinylarginine dihydrolase	392.851807	1.78382712	0.12432514	14.3480801	8.64E-45
Acetolactate synthase small subunit	409.102322	1.77355483	0.18166639	9.76270186	3.16E-21
LysR family transcriptional regulator YdcI	558.785885	1.76641842	0.15754107	11.2124315	1.06E-27
hypothetical protein	64.5176293	1.74921143	0.26407358	6.62395476	2.90E-10
Glycerol-3-phosphate ABC transporter%2C periplasmic glycerol-3-phosphate-binding protein (TC 3.A.1.1.3)	640.094984	1.73470655	0.12292486	14.1119258	2.37E-43
Curli production assembly/transport component CsgF	262.188683	1.73138803	0.14452542	11.9798166	1.88E-31
Alanine racemase	6420.7976	1.72108186	0.11927149	14.4299518	2.78E-45
Citrate synthase (si)	34824.5074	1.70310613	0.14184524	12.0067908	1.40E-31
Acetolactate synthase large subunit	2333.06692	1.69324772	0.23809106	7.11176526	1.09E-11
Osmotically inducible protein OsmY	30903.101	1.67223355	0.15786338	10.5929162	7.92E-25
Glycolate utilization operon transcriptional activator GlcC	369.171499	1.66962128	0.15665839	10.6577202	4.13E-25
FIG01220641: hypothetical protein	1440.35321	1.66167965	0.15052279	11.0393888	6.95E-27
Lysine-arginine-ornithine-binding periplasmic protein precursor (TC 3.A.1.3.1)	90.0433688	1.6591965	0.22087604	7.51188981	6.15E-13
HTH-type transcriptional regulator gadW	737.09523	1.6538028	0.18846372	8.77517836	2.56E-17
Lysine-arginine-ornithine-binding periplasmic protein precursor (TC 3.A.1.3.1)	23.816311	1.65163231	0.40618903	4.06616668	0.00018749
Succinate dehydrogenase iron-sulfur protein	3179.61141	1.64126201	0.12044408	13.6267557	1.78E-40
UPF0410 protein YmgE	74.6591253	1.64074541	0.28984438	5.66078051	9.23E-08
FIG00638507: hypothetical protein	9.3773631	1.63980538	0.57133538	2.87012749	0.01062485
L-lactate dehydrogenase	1384.85743	1.63879718	0.10848368	15.1063931	1.51E-49
Osmoprotectant ABC transporter permease protein YehY	414.760017	1.62717031	0.15302089	10.6336484	5.23E-25
Succinate dehydrogenase flavoprotein subunit	4475.24257	1.60789453	0.1343579	11.9672499	2.17E-31
Hypothetical protein ycgF	475.646157	1.60696297	0.15052287	10.6758725	3.45E-25
FIG00639204: hypothetical protein	85.7799927	1.59572256	0.23995498	6.65009152	2.45E-10
Putative cytoplasmic protein	467.832544	1.59545865	0.15424342	10.3437711	1.03E-23
Sodium-dependent phosphate transporter	1825.26224	1.56527849	0.13442449	11.6442951	8.53E-30
Glycolate dehydrogenase	316.21172	1.56111611	0.13078079	11.9368916	3.07E-31
Di/tripeptide permease YbgH	5458.50101	1.55810339	0.22010999	7.07874893	1.37E-11
Arginine/ornithine antiporter ArcD	428.803072	1.55502683	0.17738436	8.76642583	2.76E-17
Cell filamentation protein fic	1697.96776	1.54949436	0.13730503	11.2850514	4.78E-28
Alkanesulfonate utilization operon LysR-family regulator Cbl	683.118156	1.53640082	0.09878491	15.5529911	1.71E-52
Acetylornithine deacetylase	6752.05556	1.5354763	0.1685994	9.10724665	1.41E-18
FIG00638355: hypothetical protein	2064.28686	1.53272782	0.13127301	11.6758795	6.11E-30
Curli production assembly/transport component CsgE	294.578471	1.5247585	0.16098944	9.47117064	5.02E-20
Trehalose-6-phosphate phosphatase	1161.2132	1.52074027	0.13313659	11.4224066	1.04E-28
Hypothetical ABC transporter ATP-binding protein yddA	413.312735	1.51451555	0.14251298	10.627211	5.57E-25
FIG00643276: hypothetical protein	115.267439	1.51311685	0.22109387	6.84377583	6.86E-11
Putrescine transport system permease protein PotH (TC 3.A.1.11.2)	2097.23737	1.50982166	0.14572669	10.3606394	8.73E-24
Cytochrome B561	172.726442	1.50980885	0.15269811	9.88754142	9.74E-22

D-amino acid dehydrogenase small subunit	7215.43682	1.49694753	0.16211022	9.23413394	4.45E-19
FIG01045643: hypothetical protein	40.3110779	1.49571975	0.34281058	4.36310845	5.50E-05
Putrescine transport ATP-binding protein PotA (TC 3.A.1.11.1)	287.160716	1.49419856	0.15043459	9.93254665	6.26E-22
L-proline glycine betaine ABC transport system permease protein ProW (TC 3.A.1.12.1)	1250.61763	1.48525295	0.18762505	7.91606966	2.94E-14
Glycolate permease	113.486833	1.48524678	0.20041436	7.41088016	1.27E-12
Glutamate Aspartate transport system permease protein GltJ (TC 3.A.1.3.4)	144.636946	1.47764579	0.22479431	6.57332383	3.98E-10
Starvation sensing protein RspA	76.6017634	1.47756747	0.23647422	6.24832358	3.03E-09
Putrescine transport ATP-binding protein PotG (TC 3.A.1.11.2)	4217.36034	1.47543614	0.17017627	8.67004652	6.33E-17
Putative exported protein	3900.09451	1.47395035	0.14410729	10.2281456	3.33E-23
Outer membrane lipoprotein Blc	2342.23673	1.47123107	0.16876283	8.71774339	4.18E-17
Glutamate Aspartate transport system permease protein GltK (TC 3.A.1.3.4)	873.639845	1.46603932	0.13770927	10.6459014	4.63E-25
Succinate dehydrogenase hydrophobic membrane anchor protein	577.867346	1.46565141	0.18916037	7.7481947	1.05E-13
Glycoprotein-polysaccharide metabolism	6906.81452	1.46338258	0.12940945	11.3081587	3.70E-28
FIG00639943: hypothetical protein	3931.58384	1.4508654	0.1073333	13.5173837	7.69E-40
Phage protein	14.9049411	1.44478783	0.45055389	3.20669258	0.00392119
Acyl-CoA dehydrogenases	1423.93951	1.44291728	0.1200042	12.02389	1.16E-31
Protein YcgL	897.180231	1.42710663	0.08443172	16.902493	7.56E-62
L-proline glycine betaine ABC transport system permease protein ProV (TC 3.A.1.12.1)	2026.02263	1.41290464	0.16690565	8.4652894	3.56E-16
FIG00638676: hypothetical protein	189.559569	1.40644563	0.27613353	5.09335326	1.88E-06
Succinate dehydrogenase cytochrome b-556 subunit	693.509918	1.4013063	0.17258917	8.11931786	5.85E-15
ID=gene:EBG00000313225	35.873071	1.39895816	0.3726309	3.75427311	0.00061469
Isocitrate lyase	1920.08317	1.39605339	0.13367774	10.4434245	3.73E-24
Probable secreted protein	5708.78007	1.3853089	0.19327193	7.16766731	7.28E-12
FIG005189: putative transferase clustered with tellurite resistance proteins TehA/TehB	405.301792	1.38435325	0.17983866	7.69775115	1.55E-13
probable beta-D-galactosidase	16.3543351	1.38194402	0.46475649	2.97347975	0.00790587
Putative carboxymethylenebutenolidase	4107.68282	1.38108398	0.12157808	11.3596467	2.07E-28
Putrescine ABC transporter putrescine-binding protein PotF (TC 3.A.1.11.2)	10915.3739	1.37657022	0.17405744	7.90871219	3.11E-14
ID=gene:EBG00000313252	13.8319692	1.37113974	0.52116544	2.63091072	0.02033695
FIG00638962: hypothetical protein	24.4875929	1.37057157	0.33194479	4.12891423	0.00014454
Transcriptional regulator CsgD for 2nd curli operon	800.609456	1.36304987	0.1440057	9.46524918	5.29E-20
Glutamate Aspartate transport system permease protein GltJ (TC 3.A.1.3.4)	1177.06183	1.35530507	0.11307496	11.9858987	1.76E-31
Acetolactate synthase small subunit	3558.38002	1.35378027	0.13748721	9.84659041	1.43E-21
Transcriptional regulator%2C GntR family	38.0702289	1.35156777	0.28564734	4.73159578	1.08E-05
Butyryl-CoA dehydrogenase	1034.52859	1.34937577	0.15139905	8.91270967	7.91E-18
Periplasmic protein YqjC	4663.72542	1.34815375	0.11293206	11.9377416	3.07E-31
ATP-dependent Clp protease ATP-binding subunit ClpA	94182.8776	1.34744501	0.14497376	9.29440651	2.60E-19
FIG00637934: hypothetical protein	112.229574	1.34662686	0.17693238	7.61096893	2.98E-13
Sulfite reductase [NADPH] flavoprotein alpha-component	5892.88834	1.34549301	0.12009611	11.2034683	1.16E-27
Succinylornithine transaminase	171.361996	1.33787806	0.1780604	7.51361915	6.09E-13
Putrescine transport system permease protein PotI (TC 3.A.1.11.2)	1604.10313	1.33342902	0.12689478	10.5081473	1.89E-24
Respiratory nitrate reductase gamma chain	380.18954	1.32751687	0.1850988	7.17193644	7.10E-12
Malate synthase	1424.61401	1.32712465	0.13588778	9.76632836	3.06E-21
Histidine ABC transporter%2C permease protein HisM (TC 3.A.1.3.1)	2070.31042	1.32346433	0.16736302	7.90774652	3.12E-14
HTH-type transcriptional regulator gadX	2462.3037	1.32239138	0.17338513	7.626902	2.65E-13
Curli production assembly/transport component CsgG	911.292567	1.32082531	0.14150884	9.33387163	1.82E-19
Spermidine Putrescine ABC transporter permease component potC (TC_3.A.1.11.1)	162.000192	1.32013653	0.19735668	6.68908964	1.91E-10
FIG00638205: hypothetical protein	20.1385186	1.315778	0.3629054	3.62567766	0.00097323
YcgN (Fragment)	705.891695	1.31428229	0.09345734	14.0629113	4.60E-43
type 1 fimbriae regulatory protein FimB	15.3718119	1.30906096	0.40096806	3.26475121	0.00326199
Alpha%2Calpha-trehalose-phosphate synthase [UDP-forming]	2813.45187	1.30717597	0.15866748	8.23846182	2.26E-15
Taurine-binding periplasmic protein TauA	455.779725	1.30380918	0.19292728	6.75803432	1.21E-10
Alkanesulfonates transport system permease protein	288.82546	1.30326789	0.21074289	6.18416074	4.50E-09
Blue copper oxidase CueO precursor	4279.56105	1.30289993	0.10932778	11.9173731	3.85E-31
Mobile element protein	92.5412217	1.29637317	0.25709891	5.04231296	2.42E-06
Cold shock protein CspA	21580.2587	1.29607563	0.15378701	8.42773141	4.85E-16
Sulfate and thiosulfate binding protein CysP	8429.56124	1.29433418	0.1328356	9.74388052	3.78E-21
Two-component system response regulator QseB	140.060131	1.29024498	0.21472332	6.00887213	1.27E-08
probable secreted protein STY4010	35.1837626	1.2899594	0.27573922	4.67818623	1.38E-05
Putative integral membrane protein	501.526734	1.28277684	0.11781229	10.888311	3.60E-26
Osmoprotectant ABC transporter ATP-binding subunit YehX	493.129345	1.27971978	0.16854874	7.59258002	3.42E-13
Entericidin B precursor	78.3067711	1.27604062	0.35374162	3.60726736	0.001039
Stationary phase inducible protein CsiE	550.666224	1.27521177	0.16981977	7.50920657	6.27E-13
FIG00641190: hypothetical protein	179.026192	1.27440096	0.2095595	6.08133238	8.33E-09
FIG00637999: hypothetical protein	3600.36341	1.26889329	0.13075501	9.70435658	5.51E-21
Osmoprotectant ABC transporter binding protein YehZ	755.753134	1.2608289	0.1286912	9.79732017	2.28E-21
Sulfate transport system permease protein CysT	3652.59024	1.25678839	0.09700828	12.955476	1.19E-36
NAD(P)H-flavin oxidoreductase	80.173648	1.25333433	0.26895758	4.65997023	1.50E-05
TsgA protein homolog	611.596422	1.24759749	0.14905716	8.36992645	7.79E-16
Protein rarD	321.594568	1.24369949	0.14640029	8.49519833	2.79E-16
Glutamate Aspartate transport ATP-binding protein GltL (TC 3.A.1.3.4)	1524.36584	1.2433861	0.09854679	12.617215	8.36E-35
prophage DLP12 integrase	24.2696031	1.23887163	0.33881367	3.65649839	0.00087402
Iron-sulfur cluster assembly protein SufB	5462.36463	1.23784742	0.12922359	9.57911324	1.79E-20
FIG00639538: hypothetical protein	1437.65365	1.23729205	0.15073669	8.20830069	2.87E-15
Putrescine aminotransferase	4480.12917	1.23271237	0.13971894	8.82280089	1.69E-17
Lead%2C cadmium%2C zinc and mercury transporting ATPase	55227.1043	1.23054577	0.11438139	10.7582689	1.46E-25
Zinc transporter ZitB	483.303353	1.22860771	0.13019733	9.43650475	6.91E-20
Endonuclease/Exonuclease/phosphatase family protein	1032.47372	1.22724867	0.2145576	5.71990297	6.64E-08
Selenoprotein O and cysteine-containing homologs	1577.93817	1.22508991	0.14134317	8.66748562	6.45E-17
Lipid A biosynthesis (KDO) 2-(lauroyl)-lipid IVA acyltransferase	848.062346	1.22174028	0.09996978	12.2210955	1.11E-32
Inner membrane protein YphA	1344.72815	1.22076027	0.14835622	8.22857512	2.44E-15
Iron binding protein SufA for iron-sulfur cluster assembly	1414.94436	1.21311064	0.12083103	10.0397274	2.20E-22
Sulfur acceptor protein SufE for iron-sulfur cluster assembly	1303.3253	1.21039156	0.13120826	9.22496469	4.83E-19
L%2CD-transpeptidase YcbB	12615.5579	1.20940043	0.06878363	17.5826772	7.19E-67
Cell division protein BolA	6060.4257	1.20853954	0.1812269	6.66865445	2.19E-10
Histidine ABC transporter%2C ATP-binding protein HisP (TC 3.A.1.3.1)	1201.20657	1.20818956	0.15962446	7.56895022	4.05E-13
hypothetical protein	56.6333403	1.20421195	0.23461327	5.13275298	1.55E-06
Potassium efflux system KefA protein / Small-conductance mechanosensitive channel	642.460302	1.20370158	0.12053908	9.98598611	3.74E-22
Putative transport protein	36.3232535	1.20141205	0.33505848	3.58567875	0.00112012
probable ribonuclease inhibitor YPO3690	97.9696432	1.20055075	0.25132875	4.77681415	8.70E-06
ID=gene:EBG00000313286	212.767355	1.20019157	0.18320794	6.55098022	4.56E-10
ID=gene:EBG00000313289	212.767355	1.20019157	0.18320794	6.55098022	4.56E-10
Osmoprotectant ABC transporter inner membrane protein YehW	296.185709	1.1977575	0.21646158	5.53334921	1.83E-07
Isocitrate dehydrogenase phosphatase	411.255081	1.19134138	0.12691559	9.38687955	1.10E-19
FIG00638313: hypothetical protein	36.6273069	1.18756783	0.42820925	2.7733353	0.0139183
FIG143828: Hypothetical protein YbgA	1097.87101	1.18657609	0.14485182	8.1916545	3.28E-15
Glycolate dehydrogenase	515.223131	1.1837964	0.09789022	12.0931021	5.08E-32
Alkanesulfonates-binding protein	437.0333	1.1834963	0.25000504	4.73388981	1.07E-05
Inner membrane protein YbhQ	430.859742	1.17345603	0.17619743	6.65989296	2.32E-10
Inner membrane protein YhjD	663.602518	1.17061217	0.10265609	11.403241	1.27E-28
Invasin	37.5013204	1.16965378	0.29081303	4.02201299	0.00022432
Putative membrane protein%2C clustering with ActP	73.0636633	1.16751026	0.25443648	4.58861198	2.07E-05
Putative transport protein	208.641741	1.14947173	0.17382625	6.61276276	3.10E-10
Deoxyribodipyrimidine photolyase	3283.44819	1.14896471	0.12494887	9.19547921	6.33E-19
Superoxide dismutase [Cu-Zn] precursor	1074.71181	1.14738098	0.16800976	6.82925212	7.55E-11
Probable glutathione S-transferase	250.711631	1.14484232	0.21926244	5.22133342	9.84E-07
Putative inner membrane protein	116.456038	1.14458065	0.18443492	6.20587826	3.93E-09
Acetolactate synthase large subunit	11164.8352	1.14144822	0.19045023	5.99341989	1.38E-08

Glucans biosynthesis protein C	130.056986	1.13804521	0.17850539	6.37541103	1.40E-09
UPF0319 protein YccT precursor	243.785649	1.13787671	0.16007722	7.10829873	1.11E-11
ID=gene:EBG00000313288	153.617788	1.13443822	0.45152086	2.5124824	0.02757299
L%2CD-transpeptidase YnhG	5259.74533	1.13050984	0.12241282	9.23522411	4.42E-19
Ren protein	141.460007	1.1267051	0.16410051	6.86594508	5.93E-11
Sialic acid-induced transmembrane protein YjhT(NanM)%2C possible mutarotase	6227.37332	1.12668604	0.13029278	8.64734076	7.60E-17
Putative Heme-regulated two-component response regulator	425.787979	1.12279314	0.19887492	5.64572529	1.00E-07
FIG00638107: hypothetical protein	1384.09237	1.11989909	0.17583857	6.36890482	1.46E-09
Malate dehydrogenase	14083.8596	1.11935875	0.09231464	12.1254741	3.49E-32
Spermidine Putrescine ABC transporter permease component PotB (TC 3.A.1.11.1)	157.471351	1.11473306	0.183928	6.06070356	9.39E-09
FIG00639812: hypothetical protein	46.6355368	1.11392812	0.3273927	3.40242196	0.00208437
FIG00638559: hypothetical protein	2179.16649	1.10278	0.17017454	6.48028793	7.23E-10
ID=gene:EBG00000313283	59.5437031	1.101558	0.28345462	3.88618818	0.00037667
ID=gene:EBG00000313290	59.5437031	1.101558	0.28345462	3.88618818	0.00037667
putative transport	338.028838	1.09882581	0.17459921	6.29341819	2.30E-09
multidrug resistance protein A	14.4490862	1.09657946	0.43568544	2.51690635	0.02728224
Iron-sulfur cluster assembly protein SufD	8512.39007	1.09321702	0.11441797	9.55459195	2.27E-20
ATP-dependent RNA helicase	1246.69568	1.09238269	0.13090253	8.34500838	9.49E-16
Cardiolipin synthetase	1507.28476	1.09008279	0.16910955	6.44601567	8.99E-10
Choline dehydrogenase	34272.1579	1.08675616	0.08454828	12.8536763	4.31E-36
Probable zinc protease pqqL	1415.93612	1.08247899	0.17067342	6.34239927	1.72E-09
Transaldolase	3969.27961	1.07947998	0.13805567	7.81916457	6.15E-14
Glutamate Aspartate periplasmic binding protein precursor GltI (TC 3.A.1.3.4)	548.82202	1.07845878	0.18625924	5.79009553	4.46E-08
MFS permease protein	59.8452248	1.0777071	0.25299801	4.25974531	8.51E-05
ABC transporter%2C periplasmic spermidine putrescine-binding protein PotD (TC 3.A.1.11.1)	797.100072	1.07655882	0.14951128	7.20051911	5.82E-12
Dihydroxy-acid dehydratase	24010.8344	1.07414467	0.1318594	8.14613646	4.74E-15
Sulfate transport system permease protein CysW	5532.30943	1.07359174	0.09022948	11.8984588	4.71E-31
Universal stress protein B	1456.99736	1.07107714	0.17674783	6.05991678	9.42E-09
UPF0098 protein ybhB	1476.25184	1.06882006	0.13607523	7.8546259	4.70E-14
Di/tripeptide permease DtpB	603.66592	1.06681371	0.10149511	10.5109864	1.85E-24
Sensory histidine kinase QseC	262.888166	1.06540915	0.15455671	6.89332179	4.92E-11
Iron-sulfur cluster assembly ATPase protein SufC	3408.4347	1.06297334	0.12787365	8.31268498	1.24E-15
D-galactonate transporter	476.514838	1.06293845	0.13697405	7.76014491	9.65E-14
Glucose dehydrogenase%2C PQQ-dependent	33003.5315	1.0626786	0.12362209	8.59618705	1.18E-16
FIG00638140: hypothetical protein	801.99003	1.05724713	0.19380281	5.45527257	2.82E-07
Inner membrane ABC transporter permease protein YcjO	18.7401854	1.05667499	0.37263221	2.8357049	0.01170581
Para-aminobenzoate synthase%2C amidotransferase component	357.397537	1.05401722	0.13136366	8.02365927	1.25E-14
ID=gene:EBG00000313239	20.0026609	1.05090934	0.367999	2.85573965	0.0110579
Sulfate-binding protein Sbp	583.233604	1.04886737	0.11736988	8.93642707	6.49E-18
PhnB protein%3B putative DNA binding 3-demethylubiquinone-9 3-methyltransferase domain protein	312.243371	1.04416734	0.15312724	6.81895226	8.03E-11
Acetate permease ActP (cation/acetate symporter)	340.982437	1.04014866	0.1261642	8.2444044	2.16E-15
Putative membrane protein	657.461967	1.03736923	0.15962715	6.49870151	6.43E-10
Osmotically inducible lipoprotein E precursor	2113.25436	1.03657373	0.17113363	6.05710112	9.54E-09
Inner membrane protein YqjF	116.82166	1.03287873	0.22594171	4.57143891	2.24E-05
Putative inner membrane protein	760.145979	1.03072186	0.10710317	9.62363544	1.17E-20
FIG00639422: hypothetical protein	1848.4743	1.02837794	0.16426505	6.26047922	2.81E-09
Ferrichrome-iron receptor	1627.18633	1.02464094	0.18324387	5.59167921	1.35E-07
Histidine ABC transporter%2C permease protein HisQ (TC 3.A.1.3.1)	3434.09414	1.02110208	0.17094009	5.97344993	1.55E-08
Sulfate adenylyltransferase subunit 2	10388.2195	1.01939649	0.10338766	9.8599431	1.27E-21
Cysteine desulfurase	5037.46928	1.01816726	0.13004909	7.82909926	5.71E-14
FIG004405: Putative cytoplasmic protein	476.462751	1.01485906	0.12716687	7.98053006	1.76E-14
FIG00638524: hypothetical protein	975.309396	1.01441858	0.11846578	8.56296743	1.57E-16
Transcription regulator [contains diacylglycerol kinase catalytic domain]	1297.28936	1.0074396	0.17114232	5.88656045	2.57E-08
FIG010773: NAD-dependent epimerase/dehydratase	28.4267675	1.00653883	0.33669159	2.98949795	0.00754143
Cytoplasmic trehalase	3875.87493	1.00453577	0.13569906	7.40267279	1.35E-12
PTS system%2C chitobiose-specific IIC component	155.867513	1.00288474	0.16788412	5.97367244	1.55E-08
FIG00638451: hypothetical protein	983.113107	1.00287689	0.18120815	5.5343919	1.82E-07
Putrescine importer	1196.40544	0.99873013	0.17419158	5.73351567	6.16E-08
N-acetylneuraminic acid outer membrane channel protein NanC	30.8141619	0.99800444	0.32936741	3.03006433	0.00672131
FIG00637885: hypothetical protein	156.098523	0.99746344	0.16618181	6.00224197	1.31E-08
Succinyl-CoA ligase [ADP-forming] alpha chain	23602.6953	0.99682101	0.10252104	9.72308683	4.62E-21
FIG01045396: hypothetical protein	77.3410312	0.99547975	0.26723871	3.72505824	0.00068229
metal-dependent phosphohydrolase	447.775581	0.98917572	0.13001857	7.60795742	3.04E-13
FMN reductase	220.245208	0.98409122	0.26401067	3.72746761	0.0006768
Mobile element protein	88.3373059	0.98306464	0.19591211	5.01788603	2.72E-06
FIG00639237: hypothetical protein	3304.50931	0.97359658	0.20952738	4.64663185	1.60E-05
2-oxoglutarate dehydrogenase E1 component	47226.9238	0.97280551	0.10084843	9.64621331	9.50E-21
Putative PerM family permease	427.557408	0.97059939	0.13128821	7.39289055	1.45E-12
Alcohol dehydrogenase	6716.73252	0.96904436	0.15325867	6.32293347	1.93E-09
Succinyl-CoA ligase [ADP-forming] beta chain	22335.0589	0.96865657	0.10015668	9.67141241	7.52E-21
hypothetical protein	35.1943941	0.96828663	0.28947949	3.34492306	0.0025203
Alcohol dehydrogenase	2074.22017	0.96481256	0.14473262	6.66617211	2.23E-10
Aconitate hydratase 2	64741.2459	0.96223806	0.09747336	9.87180544	1.13E-21
UPF0028 protein YchK	1119.06345	0.96192981	0.08952328	10.745024	1.68E-25
FIG00639826: hypothetical protein	1479.55302	0.96035869	0.11714508	8.19802824	3.12E-15
Putative transport protein	326.465187	0.95926248	0.12750636	7.52325176	5.68E-13
Transketolase	10991.5657	0.95897802	0.1405417	6.82344094	7.82E-11
Putative cytoplasmic protein %2Cprobably associated with Glutathione-regulated potassium-efflux	340.431781	0.95833633	0.15540888	6.16654807	5.00E-09
Serine transporter	155.031187	0.95457074	0.1514481	6.30295618	2.17E-09
3-isopropylmalate dehydratase small subunit	2117.65023	0.95346884	0.12059525	7.90635465	3.15E-14
Gamma-glutamyltranspeptidase	2454.83478	0.95322769	0.14998241	6.35559652	1.59E-09
Hnr protein	1834.52069	0.9520369	0.0991388	9.60307085	1.43E-20
Putative transport protein	100.87933	0.95001982	0.1828409	5.19588233	1.12E-06
FIG01045311: hypothetical protein	141.999043	0.94414988	0.22835873	4.13450309	0.00014166
Luciferase-like monooxygenase	376.039436	0.94325713	0.15773651	5.97995417	1.50E-08
PTS system%2C maltose and glucose-specific IIC component	36.147355	0.94120694	0.32815212	2.86820314	0.010678
Putative lipase	193.586995	0.93856334	0.15290078	6.13838156	5.94E-09
probable lipoprotein	481.379167	0.93289045	0.21576646	4.32361196	6.50E-05
Transposase	16.3610077	0.93190411	0.38681535	2.40917046	0.03559677
Inner membrane protein YqjE	7184.00992	0.9318878	0.13450853	6.92809461	3.87E-11
FIG00638099: hypothetical protein	94.3748779	0.92616551	0.2673746	3.46392483	0.00169145
Glutamate transport ATP-binding protein	9262.68558	0.92601559	0.1222372	7.57556252	3.86E-13
Alkanesulfonate monooxygenase	714.020372	0.92560711	0.25365994	3.64900779	0.00089731
Heme-regulated cyclic AMP phosphodiesterase	2505.2806	0.92500236	0.12279759	7.53274019	5.31E-13
Cytoplasmic protein YaiB	567.002172	0.92079048	0.38097369	2.41693985	0.03497767
Regulator of sigma D	1549.34401	0.91668866	0.10356931	8.85096845	1.33E-17
Branched-chain amino acid aminotransferase	20796.5863	0.91405814	0.10260867	8.90819631	8.21E-18
Glutamate synthase [NADPH] large chain	37140.7567	0.91369176	0.11327722	8.06597969	9.00E-15
High-affinity choline uptake protein BetT	3903.55408	0.91267433	0.08524039	10.707064	2.51E-25
L-cystine uptake protein TcyP	4590.47579	0.9121549	0.10277255	8.87547245	1.08E-17
ID=gene:EBG00000313298	36.7180009	0.90553701	0.36882624	2.45518601	0.03185941
Pyruvate oxidase [ubiquinone%2C cytochrome]	11746.3283	0.90071029	0.15000206	6.00465296	1.30E-08
Threonine dehydratase biosynthetic	11265.8702	0.89862528	0.12125821	7.41084048	1.27E-12
CsiR%2C transcriptional repressor of CsiD	445.14875	0.8973634	0.18406733	4.875191	5.44E-06
FIG00643552: hypothetical protein	43.2855252	0.8950487	0.29972473	2.98623572	0.00760068
Putative transport system permease protein	27.3115831	0.89371415	0.34143135	2.61755153	0.02105488
Probable tonB-dependent receptor yncD precursor	743.602826	0.88972124	0.11027748	8.06802324	8.87E-15

Putative lipase	1181.87359	0.886532	0.14564558	6.08691307	8.07E-09
UPF0379 protein yjfY precursor	42.0941742	0.88522754	0.33249844	2.66235098	0.01876088
Transcriptional regulator%2C GntR family	32.5568601	0.88467684	0.28531276	3.10072652	0.00544605
FIG00896318: hypothetical protein	261.759587	0.87842417	0.14899467	5.89567508	2.44E-08
Inner membrane protein YqjK	5651.91378	0.8733717	0.12090712	7.22349298	4.93E-12
Propionate catabolism operon regulatory protein PrpR	73.4675429	0.87330099	0.21635354	4.03645346	0.000212
FIG00638412: hypothetical protein	55.5446152	0.86843943	0.25194371	3.44695822	0.0017941
Glutathione-regulated potassium-efflux system protein KefB	1314.91023	0.86725953	0.09857202	8.79823212	2.10E-17
adherence and invasion outermembrane protein (Inv%2Cenhances Peyer's patches colonization)	260.791346	0.86478046	0.13647486	6.33655497	1.78E-09
Glutathione S-transferase%2C omega	781.583231	0.8639398	0.1299076	6.65041759	2.45E-10
Ribosomal RNA large subunit methyltransferase A	282.937344	0.86083567	0.16520698	5.21064943	1.04E-06
Putative transport protein	369.476461	0.86070556	0.11596976	7.42181014	1.18E-12
hypothetical protein YjdJ	87.1206476	0.85686359	0.23895084	3.58594095	0.00111979
Putative iron compound permease protein of ABC transporter family	1392.80373	0.85442492	0.11083309	7.70911427	1.42E-13
FIG00638997: hypothetical protein	495.579937	0.85347929	0.1276713	6.68497371	1.97E-10
type 1 fimbriae regulatory protein FimE	69.333196	0.85287137	0.22752623	3.74845292	0.00062679
adherence and invasion outermembrane protein (Inv%2Cenhances Peyer's patches colonization)	415.619984	0.84910349	0.10805077	7.85837548	4.58E-14
FIG00638941: hypothetical protein	3443.03809	0.84712209	0.13640732	6.21023927	3.83E-09
FIG00639659: hypothetical protein	27.7641841	0.84383127	0.31374825	2.68951706	0.01750409
Glutamate Aspartate transport system permease protein GltK (TC 3.A.1.3.4)	129.4046	0.84144664	0.16941091	4.96689756	3.50E-06
PTS system%2C sorbose-specific IIC component	30.702995	0.8408966	0.35276637	2.38372097	0.03776557
hypothetical protein	45.8183889	0.83767256	0.30977163	2.70416169	0.01685559
Hyphotheical protein	2995.51158	0.83476251	0.10895062	7.66184237	2.04E-13
3-oxoacyl-[acyl-carrier protein] reductase	61.3611064	0.83224214	0.25883106	3.21538746	0.00381847
Rhodanese-related sulfurtransferases	1037.56288	0.83210588	0.09829169	8.46567867	3.55E-16
Taurine transport ATP-binding protein TauB	644.137384	0.82990703	0.15395074	5.3907311	4.00E-07
Glutamate synthase [NADPH] small chain	13325.7724	0.82923628	0.08354057	9.92615041	6.65E-22
hypothetical protein	244.231086	0.82394148	0.12717662	6.47871796	7.29E-10
FIG00637864: hypothetical protein	502.177101	0.82076007	0.10444529	7.85827785	4.58E-14
Glutamate Aspartate periplasmic binding protein precursor GltI (TC 3.A.1.3.4)	11405.7139	0.81655048	0.0987493	8.26892386	1.77E-15
ID=gene:EBG00000313232	30.8539421	0.81611839	0.2976799	2.74159727	0.01519219
ID=gene:EBG00000313246	30.8539421	0.81611839	0.2976799	2.74159727	0.01519219
ID=gene:EBG00000313247	30.8539421	0.81611773	0.2976799	2.74159727	0.01519219
Dipeptide transport system permease protein DppB (TC 3.A.1.5.2)	642.67993	0.81578838	0.1611059	5.06367795	2.17E-06
Soluble pyridine nucleotide transhydrogenase	3813.21798	0.81541217	0.10689254	7.62833575	2.63E-13
FIG00638228: hypothetical protein	93.1890339	0.8150274	0.27890439	2.92224659	0.00916727
Putative cytochrome oxidase subunit	707.674735	0.81289253	0.17629856	4.61088588	1.88E-05
Glutamate transport membrane-spanning protein	5569.97827	0.8126757	0.11904395	6.82668599	7.67E-11
Fumarate hydratase class II	1435.95899	0.81151365	0.11244668	7.21687485	5.17E-12
Galactosamine-6-phosphate isomerase (galactosamine-6-phosphate deaminase)	28.9942327	0.8047719	0.34716559	2.31812118	0.04394402
Nucleoside diphosphate kinase	273.413814	0.80470633	0.15022284	5.35675081	4.80E-07
PTS system%2C galactosamine-specific IIC component	59.5920063	0.80416133	0.26681936	3.01387921	0.00704137
Protein yceI precursor	2121.53262	0.80277389	0.14095875	5.69509785	7.63E-08
Uncharacterized membrane protein YqjD	3658.42758	0.80224366	0.14384916	5.57697854	1.46E-07
Integrase	486.639274	0.8021123	0.12047602	6.65785864	2.34E-10
Permease of the drug/metabolite transporter (DMT) superfamily	419.759368	0.80191981	0.14812904	5.41365711	3.54E-07
Sulfite reductase [NADPH] hemoprotein beta-component	7021.28886	0.80039687	0.14368216	5.57060721	1.51E-07
Isocitrate dehydrogenase [NADP]	108220.546	0.7997833	0.08625804	9.27198517	3.19E-19
Transcriptional regulator%2C TetR family	180.659235	0.79861663	0.17344404	4.60446278	1.93E-05
Putative inner membrane protein	269.040779	0.79831682	0.15224353	5.2436829	8.74E-07
3-isopropylmalate dehydratase large subunit	4383.97036	0.79641453	0.08573924	9.28879844	2.73E-19
2-isopropylmalate synthase	3091.27408	0.79584082	0.15830135	5.02737854	2.60E-06
Dipeptide transport system permease protein DppC (TC 3.A.1.5.2)	749.895687	0.79281085	0.13987262	5.66809163	8.87E-08
Thioredoxin 2	421.152745	0.79262079	0.14091848	5.624676	1.12E-07
FIG00638146: hypothetical protein	45.2349271	0.79119689	0.27611821	2.86542815	0.01076027
FIG00639292: hypothetical protein	342.297551	0.79078164	0.17157573	4.60893644	1.89E-05
General secretion pathway protein C	118.077392	0.78846448	0.17039391	4.62730426	1.74E-05
Rtn protein	273.213586	0.78752469	0.1248686	6.30682745	2.13E-09
D-galactonate transporter	89.6025368	0.78603145	0.20467716	3.84034763	0.00044746
ID=gene:EBG00000313233	38.0841824	0.78582731	0.29661952	2.64927709	0.0194111
Allantoinase	974.480729	0.78230555	0.11180977	6.9967548	2.42E-11
Oxidoreductase	669.261068	0.78113805	0.13346373	5.85281154	3.13E-08
Maltose O-acetyltransferase	2459.01779	0.78022214	0.13393065	5.82556832	3.63E-08
3-isopropylmalate dehydrogenase	3635.21584	0.77992328	0.11080058	7.03898203	1.81E-11
Putative GTP-binding protein YdgA	12445.8944	0.77988835	0.09336153	8.35342337	8.89E-16
HTH-type transcriptional regulator mlrA	765.885489	0.77850768	0.17829269	4.36645875	5.43E-05
COG1399 protein%2C clustered with ribosomal protein L32p	6118.07203	0.77678807	0.13276681	5.85077024	3.16E-08
Isopentenyl-diphosphate delta-isomerase	526.733892	0.77652878	0.18447866	4.2093149	0.00010483
FIG00637968: hypothetical protein	214.881827	0.77608357	0.13898091	5.58410213	1.41E-07
FIG00639587: hypothetical protein	41.235708	0.7741973	0.31355477	2.46909752	0.03079454
chaperone FimC	236.781544	0.77403647	0.19717605	3.92561105	0.00032457
Methylglyoxal reductase%2C acetol producing	3480.99838	0.77392514	0.13770372	5.6202194	1.15E-07
putative exported protein	52.8902365	0.77301773	0.22891035	3.37694521	0.00226346
NgrB	97.5675486	0.77073663	0.25787584	2.98878963	0.00755032
Inner membrane component of tripartite multidrug resistance system	84.6792704	0.76935813	0.20323062	3.78564083	0.00054659
FIG005119: putative inner membrane protein	383.980361	0.76821564	0.12107839	6.34477898	1.70E-09
Putative inner membrane protein	231.693429	0.76633536	0.16116709	4.75491203	9.67E-06
Putative oxidoreductase	799.270774	0.76623098	0.23376208	3.27782414	0.00313038
expressed protein	57.4641677	0.76566109	0.28346836	2.70104602	0.0169878
LysR family transcriptional regulator YnfL	231.679853	0.76305069	0.133435	5.71851985	6.69E-08
hypothetical protein	102.997789	0.76217825	0.21338344	3.57187157	0.00117323
Ribonuclease E	19609.4793	0.75986631	0.11289175	6.73092884	1.45E-10
Permease of the drug/metabolite transporter (DMT) superfamily	50.9192055	0.75864587	0.2765402	2.74334755	0.01513529
FIG00638229: hypothetical protein	243.665559	0.75612213	0.13925628	5.42971642	3.24E-07
N-3-oxohexanoyl-L-homoserine lactone quorum-sensing transcriptional activator @ N-3-oxooctanoyl-L-homoserine lactone quorum-sensing transcriptional activator	1513.51985	0.75155442	0.10172584	7.3880386	1.50E-12
Taurine transport system permease protein TauC	499.762195	0.7511196	0.14405785	5.2140135	1.02E-06
Putative arylsulfatase regulatory protein	244.909149	0.74709887	0.17489567	4.27168314	8.09E-05
Tyrosine-specific transport protein	210.050041	0.74523006	0.15977407	4.66427413	1.47E-05
Aminomethyltransferase (glycine cleavage system T protein)	2236.434	0.74480022	0.09256109	8.04657966	1.05E-14
Permease of the drug/metabolite transporter (DMT) superfamily	1199.44667	0.73904717	0.14323325	5.15974594	1.35E-06
UPF0410 protein YeaQ	517.843308	0.73508252	0.2226401	3.30166267	0.00289626
Acetyl-CoA:acetoacetyl-CoA transferase%2C alpha subunit	391.602177	0.73437702	0.15440371	4.7562136	9.62E-06
Electron transport complex protein RnfB	471.074249	0.7296783	0.13805234	5.28551907	7.06E-07
4-aminobutyraldehyde dehydrogenase	783.107295	0.72856569	0.12058884	6.04173388	1.04E-08
Phosphoadenylyl-sulfate reductase [thioredoxin]	2468.8612	0.72791049	0.13823361	5.26579957	7.81E-07
Mobile element protein	323.273868	0.72753177	0.15577337	4.67045035	1.43E-05
Z5092 protein	36.3195792	0.72702669	0.27638574	2.63047828	0.0203423
L%2CD-transpeptidase ErfK	1058.30064	0.72692206	0.08586618	8.46575545	3.55E-16
COG1242: Predicted Fe-S oxidoreductase	257.063342	0.72568151	0.14494884	5.00646641	2.88E-06
Uncharacterized membrane lipoprotein clustered with tellurite resistance proteins TehA/TehB	1037.9224	0.72418529	0.11635641	6.22385384	3.53E-09
Alkanesulfonates ABC transporter ATP-binding protein / Sulfonate ABC transporter%2C ATP-binding subunit SsuB	416.4877	0.724094	0.18713095	3.86945083	0.0004016
Rod shape-determining protein MreD	404.671848	0.7234022	0.12434047	5.81791434	3.79E-08
Ribonuclease P protein component	1264.94081	0.71832344	0.11905534	6.03352547	1.09E-08
FIG00638351: hypothetical protein	197.956486	0.71666988	0.1909678	3.75283095	0.00061732
Phosphate starvation-inducible protein PsiF	751.249489	0.7147518	0.1285744	5.55905211	1.60E-07
orf%3B Unknown function	29.3152169	0.71326683	0.30249354	2.35795723	0.04006265
FIG094199: Fumarylacetoacetate hydrolase	4239.01864	0.71082097	0.1218477	5.83368415	3.47E-08

High-affinity branched-chain amino acid transport system permease protein LivH (TC 3.A.1.4.1)	227.10355	0.70813835	0.1543168	4.5888611	2.07E-05
Betaine aldehyde dehydrogenase	24154.1064	0.7074038	0.08339619	8.48244773	3.10E-16
Sodium-Choline Symporter	56.9712508	0.70579628	0.24010493	2.93953268	0.00872191
Lipoprotein releasing system transmembrane protein LolC	1286.0605	0.70578526	0.07939373	8.88968564	9.60E-18
Endonuclease VIII	2541.06218	0.70563553	0.12815537	5.50609408	2.13E-07
Haemolysin expression modulating protein	4019.30016	0.70540557	0.10767097	6.55149251	4.56E-10
D-xylose transport ATP-binding protein XylG	131.606275	0.70165068	0.18535952	3.78535019	0.00054682
3-hydroxydecanoyl-[acyl-carrier-protein] dehydratase	1551.91547	0.70145144	0.16987408	4.12924343	0.00014445
Malate synthase G	3282.30725	0.70096001	0.08501595	8.24504149	2.16E-15
Spermidine Putrescine ABC transporter permease component PotB (TC 3.A.1.11.1)	704.442477	0.70015367	0.13343548	5.24713255	8.61E-07
FIG00638765: hypothetical protein	60.9609672	0.69889494	0.26195603	2.66798568	0.01852287
Electron transport complex protein RnfA	293.377057	0.69840311	0.14876812	4.69457502	1.28E-05
Serine transporter	206.949672	0.69663536	0.1442563	4.82915047	6.78E-06
Putative transport protein/putative regulator	535.306758	0.69460699	0.10278127	6.75810861	1.21E-10
hypothetical protein	78.7269097	0.69411257	0.19828903	3.50050921	0.00149757
Maltodextrin phosphorylase	2207.42894	0.69343877	0.11855345	5.84916577	3.18E-08
Phage tail fiber assembly protein	32.9501609	0.68913302	0.28275461	2.4372123	0.03331278
Proposed peptidoglycan lipid II flippase MurJ	779.194793	0.6860763	0.12525082	5.47761912	2.50E-07
Predicted sugar ABC transport system%2C periplasmic binding protein YphF precursor	68.5310173	0.68543397	0.22545053	3.04028552	0.00653055
FIG00637898: hypothetical protein	46.8769696	0.68536757	0.28756985	2.38330815	0.03777276
FIG002337: predicted inner membrane protein	3196.90294	0.68508635	0.09838798	6.96311048	3.04E-11
Electron transport complex protein RnfC	1572.51387	0.68356539	0.1189339	5.74743966	5.71E-08
Putative molybdenum transport ATP-binding protein modF	10686.1545	0.6812973	0.07868724	8.65829494	6.95E-17
Soluble aldose sugar dehydrogenase%2CPQQ-dependent	438.762761	0.68069675	0.12209068	5.57533757	1.47E-07
Dihydrolipoamide succinyltransferase component (E2) of 2-oxoglutarate dehydrogenase complex	32472.193	0.67863994	0.08740003	7.76475643	9.33E-14
Xylulose kinase	766.714649	0.67545108	0.13964454	4.83693166	6.55E-06
Putative membrane protein	234.774746	0.67511922	0.13850419	4.87435943	5.46E-06
Glycine dehydrogenase [decarboxylating] (glycine cleavage system P protein)	6469.85647	0.66991025	0.10130692	6.61267977	3.10E-10
FIG00639135: hypothetical protein	36.8537912	0.66980436	0.27257594	2.45731283	0.03168655
Lactam utilization protein LamB	6317.83185	0.66901009	0.09948425	6.72478372	1.51E-10
Low-affinity inorganic phosphate transporter	3206.72521	0.66777461	0.12024138	5.55361744	1.65E-07
putative permease of ferrichrome ABC transporter	1080.28576	0.66776418	0.12720802	5.24938722	8.51E-07
Transcriptional regulatory protein YciT	2771.3394	0.66502589	0.10149687	6.55218145	4.55E-10
D-3-phosphoglycerate dehydrogenase	99.8427276	0.66469406	0.20094811	3.30778965	0.00284633
ID=gene:EBG00000313285	76.317398	0.66414671	0.19495165	3.40672534	0.00205721
ID=gene:EBG00000313287	76.317398	0.66414671	0.19495165	3.40672534	0.00205721
ABC-type polar amino acid transport system%2CATPase component	420.535413	0.66296559	0.12353203	5.36675041	4.56E-07
LysR family transcriptional regulator YcjZ	349.482073	0.66035554	0.11686383	5.65064113	9.76E-08
hypothetical protein	48.6002504	0.65897502	0.257389	2.56022997	0.02444454
4-alpha-glucanotransferase (amylomaltase)	1534.62934	0.65822696	0.10862279	6.05975017	9.42E-09
Glutamate-aspartate carrier protein	4662.5715	0.6580681	0.09033157	7.28502904	3.15E-12
Branched-chain amino acid transport ATP-binding protein LivG (TC 3.A.1.4.1)	308.759369	0.65805227	0.14958311	4.39924172	4.72E-05
Mobile element protein	172.550232	0.65679519	0.13560342	4.84350015	6.35E-06
tRNA dihydrouridine synthase B	3258.21306	0.6557862	0.14182859	4.62379404	1.77E-05
Putative purine permease ybbY	212.423574	0.6552354	0.14875208	4.40488229	4.62E-05
Cellulose synthase catalytic subunit [UDP-forming]	5063.04335	0.65506172	0.09555655	6.85522562	6.36E-11
Putative oxidoreductase	39.8524039	0.65449576	0.27052362	2.4193664	0.03481096
Hypothetical Zinc-finger containing protein	288.195776	0.65363306	0.16894003	3.86902422	0.00040199
FIG00637950: hypothetical protein	70.0278	0.65207485	0.23705634	2.75071678	0.01484586
Evolved beta-D-galactosidase%2C alpha subunit	111.139063	0.65190792	0.18715491	3.48325316	0.00158669
biofilm regulator BssR	5844.44077	0.65162159	0.15538102	4.19370137	0.00011154
FIG006303: protein yraQ	482.678892	0.65037883	0.13696584	4.74847491	9.97E-06
Outer-membrane protein yhbX precursor	121.775407	0.64996548	0.16550452	3.9271765	0.00032272
Glucose-1-phosphate thymidylyltransferase	76.4212502	0.64951415	0.21917815	2.96340741	0.00812775
Sulfate and thiosulfate import ATP-binding protein CysA	13770.9014	0.64779402	0.10697978	6.05529404	9.64E-09
Thiol:disulfide interchange protein DsbG precursor	652.103512	0.64731836	0.13548249	4.77787452	8.67E-06
Electron transport complex protein RnfD	374.604537	0.64573986	0.14910312	4.3308273	6.30E-05
Putative regulator	86.868497	0.64534724	0.20605351	3.13194003	0.00495216
Intergenic-region protein	59.7191576	0.64499142	0.24092735	2.67711992	0.01807175
FIG00638001: hypothetical protein	65.3117575	0.64375942	0.22355011	2.8797097	0.01034121
NADH pyrophosphatase	1220.19628	0.64214231	0.08946484	7.1775943	6.84E-12
Polymyxin resistance protein ArnC%2C glycosyl transferase	2075.06535	0.64160611	0.12540785	5.11615575	1.69E-06
FIG00643895: hypothetical protein	97.2996976	0.63922418	0.17760441	3.59914579	0.0010682
Uncharacterized protein ygiV	182.070604	0.63920826	0.18517056	3.45199726	0.00176328
FIG00638983: hypothetical protein	162.457711	0.63905873	0.16809097	3.80186243	0.00051659
Transporter	77.327231	0.63822392	0.2107921	3.02774111	0.00676285
FIG00640398: hypothetical protein	84.8234341	0.63601667	0.24185443	2.62974991	0.02036539
Gamma-D-Glutamyl-meso-Diaminopimelate Amidase	120.939177	0.63412697	0.16550706	3.83141945	0.00046259
Putative transport protein	75.7281716	0.6328793	0.24395081	2.5942906	0.02236249
FolM Alternative dihydrofolate reductase 1	489.169243	0.63175981	0.10254859	6.16059022	5.18E-09
Enoyl-CoA hydratase	674.273722	0.63138877	0.1104213	5.71799795	6.70E-08
Xanthine permease	941.949525	0.63137344	0.14957056	4.22124133	9.99E-05
4-hydroxybenzoyl-CoA thioesterase family active site	168.969497	0.63048155	0.15100208	4.17531715	0.00011992
Putative inner membrane protein	155.060961	0.62973377	0.1561312	4.03336277	0.00021445
Phage shock protein D	413.931387	0.6285401	0.17235552	3.64676507	0.00090387
putative integral membrane protein	674.873484	0.62846999	0.09519118	6.60218718	3.32E-10
Xylose ABC transporter%2C permease protein XylH	225.90315	0.62842411	0.16022069	3.92224082	0.00032836
Trans-aconitate 2-methyltransferase	476.329247	0.62773368	0.14257927	4.40269947	4.66E-05
Long-chain-fatty-acid--CoA ligase	67.8293344	0.62545628	0.21567472	2.89999812	0.00975895
FIG018329: 1-acyl-sn-glycerol-3-phosphate acyltransferase	2451.1907	0.62531938	0.12925982	4.83769336	6.53E-06
Ethanolamine permease	98.1464896	0.62348684	0.20246908	3.07941755	0.0058027
FIG00510289: hypothetical protein	171.52264	0.6221403	0.15757987	3.94809505	0.00029935
Transporter%2C putative	410.981311	0.62107586	0.18747228	3.31289431	0.00279847
Hypothetical protein GlcG in glycolate utilization operon	330.733387	0.62104356	0.11675939	5.31900306	5.90E-07
Mobile element protein	69.683755	0.62026801	0.20658008	3.00255471	0.00727932
Uncharacterized GST-like protein yibF	224.228639	0.61995761	0.15639088	3.9641546	0.00028148
FIG00638742: hypothetical protein	427.451223	0.61952205	0.17004758	3.64322778	0.00091573
Phosphate:acyl-ACP acyltransferase PlsX	2732.26971	0.61662181	0.10794917	5.71214959	6.92E-08
Sulfate adenylyltransferase subunit 1	17411.5435	0.6147731	0.10967162	5.60558041	1.25E-07
Catalase	1840.70745	0.6143797	0.14602761	4.20728463	0.00010568
L-serine dehydratase 1	2935.26397	0.61384293	0.14501307	4.23301792	9.50E-05
UDP-4-amino-4-deoxy-L-arabinose--oxoglutarate aminotransferase	3890.74207	0.61348535	0.10955063	5.60001682	1.29E-07
Hypothetical transcriptional regulator yidL	290.650077	0.61295765	0.14425356	4.24916823	8.90E-05
Macrolide-specific efflux protein MacA	1070.96914	0.61246161	0.10017146	6.11413302	6.86E-09
hypothetical protein	127.734585	0.61238131	0.1798741	3.40449961	0.00207131
FIG005274: hypothetical protein	7867.41759	0.61207764	0.06746633	9.0723427	1.93E-18
FIG01069516: hypothetical protein	1112.29418	0.61136722	0.08926727	6.84872781	6.64E-11
hypothetical protein	728.2644	0.60947039	0.10170426	5.99257492	1.39E-08
Ribosomal large subunit pseudouridine synthase C	339.46995	0.60739695	0.14509676	4.1861509	0.00011473
Mobile element protein	43.2987537	0.60620274	0.24396664	2.48477714	0.02959902
Oxidoreductase%2C aldo/keto reductase family	5129.24488	0.60495492	0.1000036	6.04933126	9.97E-09
Branched-chain amino acid transport system permease protein LivM (TC 3.A.1.4.1)	286.520528	0.60229412	0.13805215	4.3628016	5.51E-05
A/G-specific adenine glycosylase	519.798054	0.59989468	0.10785795	5.56189588	1.58E-07
Manganese transport protein MntH	15972.3118	0.59837598	0.09671832	6.18679048	4.43E-09
Cytochrome c-type heme lyase subunit nrfE%2Cnitrite reductase complex assembly	94.1818349	0.59810345	0.20903105	2.86131386	0.01087715
DNA-binding protein Fis	874.593917	0.5971327	0.13758018	4.34025248	6.07E-05
Rod shape-determining protein MreC	1387.97778	0.59659915	0.1033052	5.77511267	4.86E-08

Membrane protein YciC%2C linked to IspA	684.436813	0.59648962	0.11951313	4.99099668	3.11E-06
Putative membrane protein	196.569734	0.59630448	0.1432863	4.16162944	0.0001269
Permeases of the major facilitator superfamily	895.121926	0.59444495	0.13862309	4.2882102	7.56E-05
Periplasmic Murein Peptide-Binding Protein MppA	2205.56006	0.59338344	0.08266163	7.17846298	6.81E-12
Flavoprotein wrbA	15409.6952	0.59191087	0.11281322	5.24682207	8.61E-07
N-acetylmuramoyl-L-alanine amidase	7612.11492	0.58983063	0.08240123	7.15803222	7.79E-12
type 1 fimbriae anchoring protein FimD	368.8127	0.58923431	0.20299648	2.90268231	0.00968668
Inner membrane protein CreD	318.880916	0.5855283	0.14854539	3.94174673	0.00030664
Aerobic glycerol-3-phosphate dehydrogenase	2195.88558	0.58481761	0.20304906	2.8801788	0.01033151
Biofilm PGA synthesis N-glycosyltransferase PgaC	515.807151	0.58467785	0.15442398	3.78618572	0.0005458
Protein involved in stability of MscS mechanosensitive channel	7633.96886	0.58418295	0.12592317	4.63920133	1.65E-05
FIG001957: putative hydrolase	514.525574	0.58093885	0.11367114	5.1106979	1.73E-06
type 1 fimbriae major subunit FimA	495.131814	0.57793661	0.20734645	2.78729926	0.0133821
Tricarboxylate transport protein TctB	52.7968734	0.57772161	0.2273491	2.54112121	0.02566343
ID=gene:EBG00000313228	64.6788675	0.5776521	0.22259717	2.59505584	0.02232719
ID=gene:EBG00000313293	64.6788675	0.5776521	0.22259717	2.59505584	0.02232719
Uncharacterized protein YidS	184.706206	0.57302647	0.17192617	3.33297986	0.00261916
FIG00637936: hypothetical protein	187.25661	0.57288305	0.19971666	2.86847904	0.01067453
Nitrogen regulation protein NtrB	1930.2044	0.572613	0.10834716	5.28498378	7.07E-07
FIG00639456: hypothetical protein	143.419202	0.57235612	0.15618393	3.66462866	0.00084917
Gifsy-2 prophage protein	82.105829	0.57228487	0.23494291	2.4358465	0.03342292
Pantothenate:Na+ symporter (TC 2.A.21.1.1)	440.979193	0.57169888	0.17299238	3.30476331	0.00287161
Calcium/proton antiporter	2004.93528	0.57145426	0.09502787	6.01354381	1.23E-08
Paraquat-inducible protein A	881.265093	0.56881139	0.09729107	5.84649131	3.23E-08
Intracellular septation protein IspA	690.986624	0.56674836	0.09623817	5.88901867	2.53E-08
HTH-type transcriptional regulator BetI	6824.26443	0.5652352	0.09662137	5.85000171	3.17E-08
Methylated-DNA--protein-cysteine methyltransferase	614.075156	0.56520798	0.10852353	5.20816087	1.05E-06
CDP-diacylglycerol--glycerol-3-phosphate 3-phosphatidyltransferase	48.842146	0.56455939	0.24738006	2.28215403	0.04779125
Protease II	1429.26837	0.56387521	0.08568655	6.58067329	3.79E-10
Alkylated DNA repair protein AlkB	305.158698	0.56267788	0.13384991	4.20379718	0.00010714
Outer membrane pore protein E precursor	96.0298642	0.56228968	0.17885286	3.14386753	0.00477484
Endoglucanase precursor	4271.27535	0.56213499	0.13015172	4.31907447	6.62E-05
Decarboxylase family protein	14786.1962	0.56122537	0.1137167	4.93529415	4.09E-06
Lipopolysaccharide heptosyltransferase III	2523.48736	0.56080559	0.09803449	5.72049301	6.63E-08
Cytochrome c-type heme lyase subunit nrfG%2Cnitrite reductase complex assembly	62.5211457	0.55927536	0.21194078	2.63882846	0.01996887
Chaperone protein HscB	1615.27385	0.55846953	0.08263905	6.75793718	1.21E-10
Carbamoyl-phosphate synthase small chain	8396.75452	0.55718439	0.20144802	2.76589652	0.01420966
Integral membrane protein TerC	362.522576	0.55616966	0.17138494	3.24514896	0.00347322
Ethanolamine ammonia-lyase light chain	487.94634	0.5555906	0.12636748	4.39662631	4.77E-05
FIG00638170: hypothetical protein	78.0847178	0.55361609	0.21872396	2.53111769	0.0263683
Periplasmic protein related to spheroblast formation	1230.89486	0.55292013	0.16525191	3.34592272	0.00251449
Ribulosamine/erythrulosamine 3-kinase potentially involved in protein deglycation	2651.19163	0.55271003	0.11644886	4.74637578	1.01E-05
Inner membrane metabolite transport protein YhjE	1715.9981	0.55092817	0.13314336	4.13785694	0.00013984
UDP-galactose:(galactosyl) LPS alpha1%2C2-galactosyltransferase WaaW	1470.32315	0.55047759	0.15697063	3.50688261	0.00146917
Ribosomal protein S12p Asp88 (E. coli) methylthiotransferase	875.074571	0.54969482	0.08842991	6.21616378	3.69E-09
ADA regulatory protein / Methylated-DNA--protein-cysteine methyltransferase	1150.10696	0.54899813	0.11910099	4.60951793	1.89E-05
Menaquinone-specific isochorismate synthase	1240.55402	0.54755384	0.09672116	5.66115894	9.23E-08
Preprotein translocase subunit SecG (TC 3.A.5.1.1)	3377.45598	0.54703717	0.12243201	4.46808936	3.54E-05
TsaC protein (YrdC domain) required for threonylcarbamoyladenosine t(6)A37 modification in tRNA	2415.72894	0.54660005	0.10651065	5.13188144	1.56E-06
Carbon-nitrogen hydrolase	1116.52351	0.54583881	0.15395823	3.54536938	0.00128988
Putative inner membrane protein	106.77639	0.54564625	0.23975913	2.27581012	0.04842734
Polymyxin resistance protein PmrG%3B Ais protein	162.166809	0.54471808	0.17539189	3.10571981	0.00536132
Multidrug resistance protein A	267.601088	0.54459008	0.19812811	2.74867653	0.0149307
FIG00638032: hypothetical protein	124.268494	0.54400327	0.21090115	2.57942296	0.02324756
Proline/sodium symporter PutP (TC 2.A.21.2.1) @ Propionate/sodium symporter	2281.23271	0.54376397	0.14425504	3.76946246	0.0005802
Inner membrane protein YqiK	602.528919	0.54323296	0.12275034	4.42551088	4.25E-05
Putative membrane protein	529.74236	0.54302711	0.13563094	4.00371125	0.00024158
YfdE protein	100.361574	0.54289814	0.22692731	2.39238787	0.03693698
Dipeptide transport ATP-binding protein DppD (TC 3.A.1.5.2)	1767.12337	0.54275899	0.12886958	4.21169201	0.00010391
Transcriptional regulator%2C AraC family	211.110084	0.54250559	0.141264	3.84036696	0.00044746
hypothetical protein	768.152793	0.5420157	0.14247914	3.80417569	0.00051304
Mobile element protein	409.103199	0.54141625	0.14493504	3.7355788	0.00065633
CDP-diacylglycerol pyrophosphatase	138.294107	0.54112482	0.1524462	3.54961178	0.00127193
Alcohol dehydrogenase	1881.30561	0.54040174	0.11646602	4.63999479	1.64E-05
Phenylalanyl-tRNA synthetase beta chain	21441.0194	0.540293	0.07273491	7.42824909	1.13E-12
FIG00639775: hypothetical protein	231.706252	0.53999799	0.15969645	3.38140253	0.00222996
Putative inner membrane protein	202.271477	0.53995503	0.16454144	3.28157476	0.00309491
probable lipoprotein	4590.58936	0.53984	0.10255582	5.26386485	7.89E-07
Pyruvate-flavodoxin oxidoreductase	3183.80319	0.53923692	0.09580842	5.62828338	1.10E-07
L-Proline/Glycine betaine transporter ProP	34398.0136	0.53922905	0.09383643	5.74647871	5.73E-08
Tetraacyldisaccharide 4'-kinase	1283.49227	0.53846615	0.11718571	4.59498122	2.01E-05
ATP-dependent protease La	2843.04998	0.53802743	0.06838311	7.8678408	4.27E-14
Glycine cleavage system H protein	1287.57476	0.53791228	0.13639518	3.94377776	0.0003043
Cellulose synthase%2C putative	2730.16904	0.53658651	0.10427626	5.14581649	1.45E-06
Mobile element protein	109.609236	0.5351957	0.17622867	3.03693895	0.00659673
Mobile element protein	305.135327	0.53495729	0.13080628	4.08969116	0.00017015
Threonine dehydratase%2C catabolic	137.713357	0.53422928	0.15701346	3.40244258	0.00208437
AmpG permease	732.618765	0.53362957	0.0904649	5.89874719	2.40E-08
Putative amino acid permease	323.750295	0.53055956	0.15677403	3.38423109	0.00221195
Ribonucleotide reductase of class Ib (aerobic)%2Cbeta subunit	8378.6323	0.53023829	0.11759191	4.50913931	2.97E-05
FIG002082: Protein SirB2	228.725161	0.52973018	0.1633466	3.24298261	0.00349319
Putative inner membrane protein	114.602517	0.52918526	0.19259132	2.74771084	0.01496685
ID=gene:EBG00000313275	55.998954	0.52829649	0.22268951	2.37234562	0.03864494
ID=gene:EBG00000313277	55.998954	0.52829649	0.22268951	2.37234562	0.03864494
ID=gene:EBG00000313278	55.998954	0.52829649	0.22268951	2.37234562	0.03864494
ID=gene:EBG00000313281	55.998954	0.52829649	0.22268951	2.37234562	0.03864494
ID=gene:EBG00000313282	55.998954	0.52829649	0.22268951	2.37234562	0.03864494
ID=gene:EBG00000313305	55.998954	0.52829649	0.22268951	2.37234562	0.03864494
Cys regulon transcriptional activator CysB	5142.57405	0.52802129	0.08053063	6.55677563	4.42E-10
Potassium uptake protein TrkH	1596.10722	0.52689911	0.0880544	5.98379095	1.46E-08
FIG00637861: hypothetical protein	445.197269	0.52538741	0.11224545	4.68070102	1.37E-05
Phosphatidylglycerophosphatase B	753.499941	0.52509881	0.1156732	4.53950283	2.59E-05
Putative deoxyribonuclease YcfH	1254.50986	0.52449012	0.09173738	5.71729984	6.72E-08
Invasin	463.861231	0.52421167	0.11875084	4.41438276	4.45E-05
FIG00638364: hypothetical protein	162.819221	0.52359589	0.14510532	3.60838527	0.00103527
COG2879%2C Hypothetical small protein yjiX	296.371175	0.52347773	0.17722816	2.95369386	0.00835496
Multidrug transporter MdtD	235.862141	0.52318977	0.15031264	3.48067722	0.00159986
Cytochrome c-type protein NrfB precursor	91.2706396	0.5226926	0.20541587	2.54455799	0.02542474
Ribosomal large subunit pseudouridine synthase B	1373.49064	0.52255705	0.09103094	5.74043362	5.93E-08
High-affinity leucine-specific transport system%2Cperiplasmic binding protein LivK (TC 3.A.1.4.1)	460.435161	0.52095647	0.10533998	4.94547701	3.89E-06
Mg(2+) transport ATPase%2C P-type	6120.75734	0.52008895	0.11814346	4.40218131	4.67E-05
Mobile element protein	141.621711	0.51973936	0.17228288	3.01677901	0.00698242
FIG01219827: hypothetical protein	246.954162	0.51965015	0.19743153	2.63205246	0.02028921
lipid A biosynthesis lauroyl acyltransferase	3774.64369	0.51946072	0.13750235	3.77783151	0.00056232
Possible exported protein	139.441458	0.51842374	0.15612032	3.32066802	0.00272867
FIG00895798: hypothetical protein	7603.64904	0.51773466	0.12489531	4.14534903	0.00013569
Peptide transport system permease protein SapC	919.9021	0.51755161	0.12253677	4.22364347	9.89E-05

Maltose operon periplasmic protein MalM	276.72118	0.5174287	0.12270516	4.21684562	0.00010165
Paraquat-inducible protein A	588.515896	0.517082	0.09942455	5.20074781	1.09E-06
Inner membrane protein translocase component YidC%2C long form	5732.02815	0.51671323	0.10483302	4.92891695	4.21E-06
SAM-dependent methyltransferase	8625.96923	0.51545467	0.10114997	5.09594491	1.86E-06
Ferric enterobactin uptake protein FepE	8626.06808	0.51445664	0.06754249	7.61678548	2.85E-13
Putative protease ydgD	2517.90637	0.51432345	0.07799952	6.59393114	3.51E-10
Potassium channel protein	1238.14701	0.5139176	0.11643619	4.41372753	4.46E-05
Lipid A export ATP-binding/permease protein MsbA	2586.20428	0.51379505	0.0841482	6.10583538	7.19E-09
Alkaline phosphatase	2289.4492	0.51275664	0.10447567	4.90790482	4.65E-06
3-oxoacyl-[ACP] synthase	3614.12742	0.51195231	0.09011194	5.68129297	8.23E-08
Putative HTH-type transcriptional regulator ycgE	1787.62487	0.51178897	0.08707339	5.87767343	2.70E-08
Acyl-CoA dehydrogenase	635.964218	0.51105475	0.11103136	4.60279653	1.94E-05
Translation elongation factor P Lys34:lysine transferase	586.148133	0.51104942	0.11338288	4.50728911	2.99E-05
6-phospho-beta-glucosidase	408.688589	0.51074569	0.12539863	4.0729765	0.00018254
FIG00638813: hypothetical protein	369.080547	0.51036656	0.15689414	3.25293585	0.00338367
IgaA: a membrane protein that prevents overactivation of the Rcs regulatory system	2006.66484	0.5102431	0.11474709	4.44667588	3.89E-05
Xylose isomerase	412.573908	0.50837895	0.13318996	3.81694659	0.00048833
Endo-1%2C4-beta-xylanase A precursor	213.827773	0.50791048	0.17344714	2.92833005	0.00901716
Lipoprotein releasing system transmembrane protein LolE	1944.41725	0.50480185	0.11498303	4.39022937	4.89E-05
FIG004453: protein YceG like	1026.72282	0.50284684	0.11556902	4.35105224	5.79E-05
Acyl-CoA dehydrogenase	2909.28181	0.50171815	0.12314717	4.07413447	0.00018178
Invasin	77.6915494	0.50139058	0.19835358	2.52776173	0.02659559
2-methylcitrate dehydratase	152.456117	0.50109743	0.15948438	3.14198433	0.00480275
Putative inner membrane protein	508.796476	0.49815767	0.1085005	4.59129399	2.05E-05
Ethanolamine utilization polyhedral-body-like protein EutK	365.431294	0.49757271	0.14199045	3.50426876	0.00148062
cysteine synthase B	3654.98936	0.49530618	0.11429845	4.33344598	6.25E-05
Fumarate hydratase class I%2C aerobic	992.240011	0.49511084	0.13760441	3.59807379	0.00107186
Inner membrane protein YfeZ	126.996348	0.49485542	0.15531411	3.18615891	0.00417694
Putative outer membrane protein	651.23666	0.49468427	0.15807939	3.12934076	0.00498436
Hybrid sensory histidine kinase in two-component regulatory system with EvgA	1562.1744	0.49464847	0.15808201	3.12906235	0.00498589
Adenylylsulfate kinase	2515.731	0.4942433	0.13615284	3.63006241	0.0009589
Na+/H+ antiporter NhaB	2575.11234	0.49131863	0.09991921	4.91715899	4.45E-06
Phosphate transport system permease protein PstC (TC 3.A.1.7.1)	951.112197	0.49045934	0.11090976	4.42214755	4.31E-05
Inner membrane protein YfiN	1133.89017	0.48971007	0.1153431	4.2456817	9.02E-05
3-deoxy-D-manno-octulosonic-acid transferase	1007.98742	0.48859636	0.09714245	5.02968935	2.57E-06
FIG00638953: hypothetical protein	851.466849	0.48822748	0.16052522	3.04143787	0.00651034
Leucine-responsive regulatory protein%2C regulator for leucine (or lrp) regulon and high-affinity branched-chain amino acid transport system	19166.9838	0.48677864	0.11406341	4.26761425	8.23E-05
FIG003145: Surface protein	397.073211	0.4866947	0.13860592	3.51135577	0.00144764
Xanthosine permease	92.3862438	0.48665787	0.19424263	2.50541223	0.0280687
Allophanate hydrolase 2 subunit 2	12046.816	0.48606528	0.11663117	4.16754166	0.00012387
Undecaprenyl-phosphate N-acetylglucosaminyl 1-phosphate transferase	2130.47444	0.48604282	0.0987763	4.92064184	4.38E-06
ADP compounds hydrolase NudE	1168.95521	0.48468019	0.13809458	3.50976971	0.0014553
Sialic acid utilization regulator%2C RpiR family	321.460478	0.48303262	0.12543861	3.85074897	0.00043087
HTH-type transcriptional regulator prsX	452.860493	0.48290438	0.12138663	3.97823383	0.00026705
Protein SseB	2097.62039	0.48284496	0.11968292	4.03436807	0.00021371
Paraquat-inducible protein B	1815.14538	0.48253988	0.11260032	4.28542171	7.64E-05
Phage shock protein B	881.995	0.48243964	0.14429316	3.34346845	0.00253191
Inner membrane protein YihY%2C formerly thought to be RNase BN	1666.09033	0.48053283	0.08174607	5.87835962	2.69E-08
Inner membrane thiol:disulfide oxidoreductase%2CDsbB-like	361.161413	0.47935144	0.13517978	3.54602915	0.00128755
Transglycosylase%2C Slt family	107.42815	0.4793395	0.16578718	2.89129404	0.01001153
transport%3B Transport of small molecules: Cations	2878.28943	0.47768337	0.08696403	5.49288451	2.29E-07
Ribulose-5-phosphate 4-epimerase and related epimerases and aldolases	133.63633	0.4765253	0.18145415	2.62614722	0.02056151
Sensor histidine protein kinase UhpB%2Cglucose-6-phosphate specific	426.871697	0.47636077	0.13222981	3.60252176	0.00105517
Shikimate 5-dehydrogenase I alpha	2900.59098	0.47628058	0.10607311	4.49011595	3.24E-05
Protease II	240.570438	0.47531806	0.15203358	3.12640178	0.00502519
DNA primase	4019.94743	0.47482115	0.09935042	4.77925684	8.62E-06
Carbamoyl-phosphate synthase large chain	43361.5612	0.47263355	0.16729836	2.82509372	0.01206795
Alpha-ketoglutarate-dependent taurine dioxygenase	730.101446	0.47202012	0.14191221	3.32614166	0.00268081
Cysteine desulfurase	15936.398	0.47198444	0.06262293	7.53692724	5.15E-13
Phosphate transport system permease protein PstA (TC 3.A.1.7.1)	882.055143	0.47087786	0.11088347	4.24660084	8.99E-05
Nicotinamide-nucleotide adenylyltransferase%2C NadR family	185.727982	0.47004097	0.17107855	2.74751545	0.01496788
FIG00638035: hypothetical protein	1375.57139	0.46989156	0.11325792	4.14886284	0.00013374
AidA-I adhesin-like protein	347.868463	0.46887057	0.1420183	3.30148006	0.0028963
FIG006163: hypothetical protein	71.6377145	0.46591572	0.19462294	2.39394044	0.03683254
Putative heat shock protein YegD	371.521165	0.46532071	0.13285472	3.50247775	0.00148858
Outer membrane porin protein NmpC precursor	138.034699	0.46254301	0.16535595	2.79725647	0.01302506
Cystine ABC transporter%2C periplasmic cystine-binding protein FliY	10285.8307	0.46203628	0.13227536	3.49298814	0.0015362
ATP-dependent RNA helicase RhlE	2396.03327	0.4619712	0.11806804	3.91275422	0.00034073
LysR family transcriptional regulator YhjC	276.387438	0.46116376	0.13909041	3.31556825	0.00277361
Glutamate synthase [NADPH] small chain	478.429814	0.4603632	0.1114606	4.13027742	0.00014393
Arylesterase precursor	772.941877	0.46031714	0.10270812	4.48179869	3.34E-05
Glucuronide transport facilitator UidC	162.072301	0.45993733	0.16942325	2.71472379	0.01636143
PTS system%2C galactosamine-specific IID component	131.859332	0.45963057	0.1826936	2.51585476	0.02735046
Cnu protein	145.372996	0.45831344	0.166835	2.74710602	0.01497868
Putative NAGC-like transcriptional regulator	232.234188	0.45810398	0.13544212	3.38228605	0.00222469
Multiple antibiotic resistance protein MarC	230.268556	0.45772809	0.14497796	3.15722545	0.00458082
Transcription repressor of multidrug efflux pump acrAB operon%2C TetR (AcrR) family	149.326216	0.45759433	0.15001462	3.05033154	0.00634284
Maltoporin (maltose/maltodextrin high-affinity receptor%2C phage lambda receptor protein)	121.213148	0.45713854	0.17861996	2.55928034	0.02448103
D-alanyl-D-alanine carboxypeptidase	7516.45473	0.45712505	0.09208875	4.96396202	3.55E-06
Galactitol-1-phosphate 5-dehydrogenase	137.73793	0.45674539	0.17607716	2.59400702	0.02236249
Multiple antibiotic resistance protein MarR	84.2102434	0.45581845	0.19816642	2.30018009	0.04587213
Putative lipase in cluster with Phosphatidate cytidylyltransferase	231.037611	0.45544546	0.12882954	3.53525641	0.00133194
Oligopeptide transport system permease protein OppB (TC 3.A.1.5.1)	3298.5274	0.45538981	0.10144369	4.48908952	3.24E-05
Methyl-directed repair DNA adenine methylase	1201.75604	0.45524165	0.1338539	3.40103386	0.00209222
Acyl-CoA thioesterase YciA%2C involved in membrane biogenesis	201.994361	0.45474003	0.16120105	2.82094962	0.01219496
Electron transport complex protein RnfG	411.851673	0.45363093	0.12692201	3.57409202	0.00116516
type 1 fimbrae adaptor subunit FimG	109.494259	0.45334894	0.19913977	2.27653649	0.04836359
Phosphoenolpyruvate synthase	28496.6618	0.45297761	0.12971784	3.49202243	0.00154072
ABC transport system%2C permease component YbhR	5537.31707	0.45244947	0.1233878	3.66689	0.00084292
Putative TEGT family carrier/transport protein	5004.32113	0.4517851	0.08175771	5.52590188	1.91E-07
Uncharacterized protein ImpJ/VasE	98.635274	0.44941623	0.1789796	2.51099129	0.02767627
Cation transport protein chaC	3578.74871	0.448728	0.11288402	3.9751243	0.00027012
Allantoin permease	144.612173	0.44820898	0.18318926	2.44669903	0.03254169
Dipeptide transport system permease protein DppC (TC 3.A.1.5.2)	1982.67332	0.44814211	0.11397695	3.93186629	0.000318
Large-conductance mechanosensitive channel	6984.19408	0.44647495	0.09901705	4.50907165	2.97E-05
tRNA (guanine46-N7-)-methyltransferase	825.636562	0.44625189	0.09556273	4.66972753	1.44E-05
4'-phosphopantetheinyl transferase	234.508701	0.4459405	0.13646405	3.26782413	0.00323492
Preprotein translocase subunit SecE (TC 3.A.5.1.1)	3615.82799	0.4446527	0.07605547	5.84642627	3.23E-08
Phosphocarrier protein%2C nitrogen regulation associated	473.044324	0.44460846	0.13338598	3.33324744	0.00261833
Chaperone protein HscA	7869.9245	0.44439521	0.08823438	5.03653135	2.49E-06
Phage shock protein C	707.040928	0.44402707	0.13823197	3.21218803	0.00385886
Putative uncharacterized protein YrbL	2814.31172	0.44372802	0.13182979	3.36591623	0.00234762
Aminodeoxychorismate lyase	1061.92997	0.44261022	0.13882937	3.18815989	0.00415322
Probable transport protein YifK	587.816977	0.44215346	0.13660102	3.23682401	0.00356278
Ribonucleotide reductase of class Ib (aerobic)%2Calpha subunit	14947.3707	0.44132565	0.11350689	3.88809557	0.00037431
TrkA%2C Potassium channel-family protein	3892.30066	0.44127978	0.1035857	4.26004534	8.50E-05
FIG034389 (not subsystem-based): hypothetical protein	529.688396	0.44101349	0.10539863	4.18424324	0.0001156

Putative membrane protein	1365.61638	0.44098697	0.08654335	5.09556165	1.86E-06
Ethanolamine utilization polyhedral-body-like protein EutL	403.346344	0.43859349	0.15202503	2.88500832	0.01019113
Iron-sulfur cluster assembly scaffold protein IscU	4524.84253	0.43614634	0.1174385	3.71382761	0.00071016
LSU ribosomal protein L34p	249.527724	0.43582788	0.19090562	2.28294944	0.0477213
Phosphate transport ATP-binding protein PstB (TC 3.A.1.7.1)	1168.22539	0.43541131	0.12146725	3.58459846	0.00112398
Cold-shock DEAD-box protein A	21096.6983	0.43531935	0.14515236	2.99905112	0.00734205
hypothetical protein	729.216901	0.43505502	0.16229622	2.68062319	0.01793387
IS1 ORF1	95.7746149	0.43444221	0.18474704	2.35155169	0.04068419
Uncharacterized protein YfiR precursor	970.699577	0.43433397	0.13541718	3.2073771	0.00391671
Putative preQ0 transporter	232.090024	0.43350158	0.17382067	2.49395871	0.02888614
Putative isomerase	242.408002	0.43258135	0.15510691	2.78892374	0.01332935
Biofilm PGA synthesis deacetylase PgaB	259.38027	0.4325721	0.14411743	3.00152515	0.00729559
Transcriptional activator of cad operon	975.68908	0.43178513	0.10514394	4.10660975	0.00015869
Putative DMT superfamily metabolite efflux protein precursor	143.971335	0.43173154	0.1827048	2.36300059	0.03953982
D-mannonate oxidoreductase	642.855134	0.43150492	0.12337163	3.49760244	0.00151295
UDP-glucose dehydrogenase	720.225155	0.43145736	0.1215903	3.5484522	0.00127665
Free methionine-(R)-sulfoxide reductase%2C contains GAF domain	1986.43204	0.43114859	0.08477535	5.08577806	1.95E-06
Spermidine Putrescine ABC transporter permease component potC (TC_3.A.1.11.1)	480.204975	0.43061017	0.1471044	2.92724195	0.00904371
Putative NAD(P)-dependent oxidoreductase EC-YbbO	2215.66277	0.43010922	0.10265446	4.18987371	0.00011315
Transcriptional regulator%2C TetR family	148.780983	0.42952935	0.17707019	2.42575753	0.03426884
L-sorbose 1-phosphate reductase	156.938246	0.42804972	0.17132767	2.49842721	0.02855211
Histidine ABC transporter%2C histidine-binding periplasmic protein precursor HisJ (TC 3.A.1.3.1)	10862.4659	0.42559819	0.15777596	2.69748448	0.01713499
FIG146278: Maf/YceF/YhdE family protein	4413.77194	0.42515303	0.08204561	5.18191054	1.21E-06
Cyclic di-GMP binding protein precursor	12879.1545	0.42506274	0.10502944	4.04708175	0.0002028
Protease III precursor	2726.76895	0.42505543	0.07668169	5.54311531	1.74E-07
CFA/I fimbrial subunit C usher protein	3049.69958	0.42460526	0.11873081	3.576201	0.00115662
Putative iron compound-binding protein of ABC transporter family	2079.75127	0.42392593	0.14724335	2.8790838	0.01035607
Glutothione ABC transporter ATP-binding protein	11031.5032	0.42354324	0.11178652	3.78885777	0.00054037
FIG00639146: hypothetical protein	624.933986	0.42319026	0.16948028	2.4969882	0.02865438
Branched-chain amino acid transport ATP-binding protein LivF (TC 3.A.1.4.1)	350.665022	0.4223939	0.13927054	3.03290211	0.00667005
FIG00637979: hypothetical protein	834.631242	0.42219403	0.08738616	4.83136054	6.71E-06
Arabinose-proton symporter	2688.3959	0.42142959	0.08285482	5.08636167	1.94E-06
FIG00641944: hypothetical protein	370.612049	0.42111443	0.15775079	2.66949177	0.01844948
Protein yhjK	1452.45516	0.42078331	0.07904731	5.32318343	5.77E-07
Methionine ABC transporter substrate-binding protein	7461.53252	0.4206244	0.13865136	3.03368402	0.00665667
SanA protein	167.14304	0.42013883	0.16064957	2.61525035	0.02117599
L-xylulose/3-keto-L-gulonate kinase	102.918056	0.41881424	0.17131615	2.44468632	0.03270824
Bacterioferritin	3119.97351	0.41829799	0.09756242	4.28749086	7.58E-05
Hypothetical MFS-type transporter protein YcaD	3644.14215	0.41743918	0.1634691	2.55362753	0.02483303
Uncharacterized ABC transporter%2C periplasmic component YrbD	839.897096	0.41731714	0.08857573	4.71141628	1.19E-05
Phosphoenolpyruvate-dihydroxyacetone phosphotransferase operon regulatory protein DhaR	992.130833	0.41652899	0.11180106	3.72562646	0.00068126
UDP-glucose:(heptosyl) LPS alpha1%2C3-glucosyltransferase WaaG	2963.30263	0.41436726	0.11473801	3.61142089	0.00102541
Low-affinity inorganic phosphate transporter	163.481685	0.41290633	0.1763883	2.34089412	0.0418067
FIG00732392: hypothetical protein	1592.14392	0.41275359	0.16136372	2.55790827	0.02456571
Tail-specific protease precursor	9770.19092	0.4126295	0.11670479	3.53566889	0.00133078
Regulatory protein SoxS	1227.05281	0.41249446	0.14299099	2.88475839	0.01019362
Ribosomal RNA large subunit methyltransferase N	1942.55044	0.41238333	0.11480446	3.59204972	0.0010954
Ribosomal large subunit pseudouridine synthase F	333.236832	0.41225611	0.128852	3.19945458	0.00401356
Putrescine transport ATP-binding protein PotA (TC 3.A.1.11.1)	1731.82739	0.41180337	0.08533458	4.82575004	6.89E-06
LysR family transcriptional regulator YneJ	178.223805	0.41139187	0.14754431	2.78825984	0.01334959
Ferrichrome-iron receptor	4299.25444	0.41092	0.12778922	3.21560764	0.0038179
O-antigen ligase	1266.19196	0.41087744	0.12047475	3.41048588	0.00203443
Uncharacterized protein YrbK clustered with lipopolysaccharide transporters	2139.30293	0.41015459	0.09225422	4.44591697	3.90E-05
DNA topoisomerase I	7923.49552	0.40998075	0.08023796	5.10956097	1.74E-06
predicted GTPase	4367.16805	0.40973348	0.10016169	4.09072061	0.00016954
YihE protein%2C a ser/thr kinase implicated in LPS synthesis and Cpx signalling	1438.82896	0.40951757	0.08823302	4.64131898	1.63E-05
Putative enzyme	294.282544	0.40926727	0.12467662	3.28263036	0.0030853
Shikimate kinase I	4671.4001	0.40875748	0.10480851	3.90004098	0.00035828
Iron binding protein IscA for iron-sulfur cluster assembly	7073.36196	0.40733222	0.06839918	5.95522079	1.73E-08
Polymyxin resistance protein PmrL%2C sucrose-6 phosphate hydrolase	254.996202	0.40626889	0.16954182	2.39627531	0.03665008
Dienelactone hydrolase family	427.598695	0.40577723	0.15494405	2.61886292	0.02098466
Multidrug transporter MdtB	600.6299	0.40531049	0.16080372	2.52052942	0.0270425
FIG00948312: hypothetical protein	2364.01572	0.40522935	0.12801159	3.16556758	0.00446666
Transcriptional activator RfaH	254.483109	0.40493915	0.15728539	2.57455037	0.02354252
Syd protein	335.391457	0.40472928	0.11066796	3.65714958	0.00087243
Inner membrane protein YrbG%2C predicted calcium/sodium:proton antiporter	891.446962	0.4045334	0.12429619	3.25459224	0.00337011
Potassium-transporting ATPase C chain	320.490867	0.40448113	0.13290495	3.04338641	0.0064759
Kup system potassium uptake protein	1119.61426	0.40423394	0.10587992	3.81785281	0.00048691
2-keto-3-deoxy-D-arabino-heptulosonate-7-phosphate synthase I alpha	17303.289	0.40356791	0.09096597	4.4364712	4.05E-05
Phenylalanyl-tRNA synthetase alpha chain	5023.92803	0.40343808	0.09423333	4.28126762	7.77E-05
AroM protein	724.210847	0.40260035	0.13583209	2.96395617	0.00812331
Dipeptide transport system permease protein DppB (TC 3.A.1.5.2)%3B putative hemin permease	1161.46657	0.40250635	0.11215125	3.58896004	0.00110768
Dipeptide transport ATP-binding protein DppF (TC 3.A.1.5.2)	1581.36199	0.40223081	0.12590016	3.19483953	0.00406827
Uncharacterized ABC transporter%2C permease component YrbE	1318.70648	0.40178185	0.10907534	3.68352611	0.0007932
Sialic acid transporter (permease) NanT	96.97534	0.401781	0.17619529	2.28031637	0.04794448
Formate dehydrogenase O gamma subunit	1913.38323	0.40105639	0.10193947	3.93425994	0.0003156
S-adenosylmethionine:tRNA ribosyltransferase-isomerase	881.391623	0.40088479	0.09529015	4.20699075	0.00010573
Rhodanese-related sulfurtransferases	1791.86693	0.40085961	0.12386354	3.23630036	0.00356488
DNA-binding protein H-NS	65364.9062	0.40057771	0.10339434	3.87427112	0.00039435
Cation transport regulator chaB	552.94642	0.39935841	0.1594144	2.50515893	0.0280687
Molybdenum cofactor biosynthesis protein MoaA	2268.52164	0.39925241	0.10280773	3.88348646	0.00038029
BAX protein	826.895605	0.39899932	0.16388533	2.43462503	0.03352001
FIG001587: exported protein	3070.81253	0.39890276	0.07951629	5.01661707	2.74E-06
Transcriptional regulator NanR	2322.59224	0.39880336	0.08474784	4.70576424	1.22E-05
Glutathione-regulated potassium-efflux system ancillary protein KefG	722.500133	0.39879772	0.1150249	3.46705542	0.001673
Putative lipoprotein	1566.50642	0.3973723	0.10720302	3.70672693	0.00072929
DNA polymerase III subunits gamma and tau	2898.866	0.39730178	0.08340049	4.76378242	9.28E-06
Exoribonuclease II	5663.52286	0.39709137	0.09916358	4.00440751	0.0002411
hypothetical protein	151.872916	0.39681713	0.15703173	2.52698696	0.02662826
Putative major fimbrial subunit precursor	101.845794	0.39657988	0.17034531	2.32809392	0.04302447
Alkyl hydroperoxide reductase protein C	54332.6178	0.39629443	0.14724112	2.69146577	0.01742017
Lysyl-lysine 2%2C3-aminomutase	1327.93722	0.39514612	0.09497367	4.16058609	0.00012738
Multidrug translocase MdfA	845.408088	0.39157201	0.10406043	3.762929	0.00059469
Lipopolysaccharide core biosynthesis protein WaaP	1884.84451	0.39142281	0.12517597	3.12698048	0.00501832
Inner membrane protein YiaH	280.741477	0.39021	0.13408425	2.91018516	0.00948339
hypothetical protein	1521.88811	0.38948449	0.13370311	2.91305481	0.00940189
Benzoate transport protein	338.825267	0.38937456	0.12772806	3.04846531	0.00637116
5'-nucleotidase YjjG	1295.25453	0.38879342	0.10102405	3.84852346	0.00043446
Lipopolysaccharide core biosynthesis protein RfaY	1046.95803	0.38753476	0.16232826	2.38735242	0.03742941
Glycerophosphoryl diester phosphodiesterase	1046.4206	0.38530293	0.12462773	3.09163088	0.00559236
Putative oxidoreductase linked to yggC	209.898694	0.38427223	0.14963853	2.56800319	0.02394439
5'-nucleotidase	130.371937	0.38401099	0.15727641	2.44163113	0.0329392
Integration host factor alpha subunit	20508.7557	0.38392732	0.10284033	3.7332371	0.00066197
FIG017861: hypothetical protein	2008.17501	0.38392457	0.11049082	3.47471919	0.00163251
Lysine decarboxylase 2%2C constitutive	3613.36418	0.38365114	0.10721262	3.57841396	0.00114848
Gluconate permease%2C Bsu4004 homolog	297.821103	0.38362437	0.13803871	2.77910725	0.01369516
Putative membrane protein	327.569731	0.38214429	0.1144922	3.33773213	0.00257976

3'(2')%2C5'-bisphosphate nucleotidase	7543.52845	0.3819036	0.09321151	4.09717226	0.00016502
Isoaspartyl aminopeptidase	8618.1051	0.37974452	0.13176611	2.88195897	0.01027897
Ferredoxin%2C 2Fe-2S	1968.1488	0.37936116	0.11339621	3.34544836	0.00251716
Putative alanine/glycine transport protein	386.3429	0.37889663	0.10494537	3.61041781	0.00102792
Putative oxidoreductase	4582.88019	0.37664958	0.10972903	3.43254271	0.00188719
Putative membrane protein	627.359776	0.37647812	0.1051515	3.58034017	0.00114085
General secretion pathway protein E	271.239191	0.37542605	0.13872381	2.70628427	0.01675694
FIG00642307: hypothetical protein	1693.17439	0.3748276	0.11335005	3.30681445	0.00285443
3-oxoacyl-[acyl-carrier-protein] synthase%2CKASIII	6198.33677	0.37316996	0.0950192	3.92731114	0.00032272
Cardiolipin synthetase	3481.57246	0.37278942	0.12414505	3.00285378	0.00727635
Ferrochelatase%2C protoheme ferro-lyase	2696.58051	0.37133589	0.0938291	3.95757721	0.00028864
General secretion pathway protein D	440.660262	0.37057613	0.13307462	2.78472439	0.01346732
Lipoprotein spr precursor	1493.12025	0.36957206	0.13080934	2.82527268	0.01206773
Cell volume regulation protein A	2821.50938	0.36956313	0.07227809	5.11307294	1.71E-06
Glutaredoxin 3 (Grx3)	1039.09907	0.36932543	0.116293	3.17581817	0.00432343
putative transporter%2C permease protein	1080.21271	0.36886903	0.10630817	3.46980883	0.00165905
FIG00638331: hypothetical protein	1974.51637	0.36845131	0.11448117	3.21844465	0.00378503
ProQ: influences osmotic activation of compatible solute ProP	6403.36895	0.36600738	0.08247069	4.4380297	4.03E-05
Long-chain-fatty-acid--CoA ligase	951.432372	0.3638968	0.08657083	4.20345761	0.00010721
Positive transcription regulator EvgA	985.798079	0.36341698	0.11200879	3.24453994	0.00347848
Putative uncharacterized protein YaiO	274.526387	0.36243491	0.13744531	2.6369392	0.02004991
Lysine-specific permease	906.138193	0.3619114	0.119953	3.01711016	0.00698159
FIG00638475: hypothetical protein	258.670048	0.36182428	0.15105328	2.39534202	0.03672634
Penicillin-insensitive transglycosylase	1285.6296	0.35990754	0.11017832	3.26659134	0.00324344
Thiamin-phosphate pyrophosphorylase	28855.2335	0.35838998	0.09847088	3.63955309	0.00092689
UDP-glucose:(glucosyl)lipopolysaccharide alpha-1%2C3-glucosyltransferase WaaO	2194.18289	0.35831705	0.1174404	3.05105437	0.00633502
Hemolysins and related proteins containing CBS domains	939.28939	0.35772982	0.10234033	3.4954922	0.00152289
Pyruvate formate-lyase	554.962445	0.35734606	0.11915931	2.99889322	0.00734205
Allophanate hydrolase 2 subunit 1	7149.14758	0.35664985	0.12318822	2.89516201	0.00989991
Thiamine kinase	1065.75251	0.35536242	0.12812937	2.77346571	0.0139183
Putative uncharacterized protein ydbH	2376.21045	0.3548023	0.11127278	3.18858127	0.00414972
Protein YkiA	411.500473	0.35435284	0.12380135	2.86226947	0.01085624
FIG00638480: hypothetical protein	510.875908	0.35361869	0.09976466	3.54452865	0.0012931
Putative GTP-binding protein	238.739348	0.35292793	0.1294599	2.72615646	0.01584665
Thymidine kinase	474.816998	0.35290292	0.11748488	3.00381573	0.00725756
regulator of length of O-antigen component of lipopolysaccharide chains	2504.71549	0.35265256	0.09715446	3.62981322	0.00095914
FIG01068233: hypothetical protein	678.472107	0.35187236	0.14574932	2.41422989	0.03518907
Penicillin-binding protein 2 (PBP-2)	1538.71157	0.35147827	0.09017331	3.89780822	0.00036131
UPF0225 protein YchJ	493.088016	0.35139904	0.1165171	3.01585812	0.00699962
Multimodular transpeptidase-transglycosylase	1485.54039	0.35138355	0.11186763	3.14106557	0.00481493
FIG136845: Rhodanese-related sulfurtransferase	897.686492	0.34982962	0.13297787	2.63073565	0.02033716
Cytoplasmic alpha-amylase	6192.85207	0.34973579	0.0949329	3.68403152	0.00079221
Transcriptional regulator%2C TetR family	3704.84751	0.34914011	0.09476345	3.6843331	0.00079185
Transcription termination factor Rho	25911.1406	0.34889071	0.09110687	3.82946643	0.00046592
Carbon starvation protein A paralog	44721.8532	0.34746278	0.08584877	4.04738222	0.0002028
UPF0131 protein YtfP	3042.86766	0.34739855	0.12718556	2.73143073	0.0156361
FIG00639050: hypothetical protein	660.976502	0.34644007	0.14636586	2.36694592	0.03919312
23S rRNA (guanine-N-2-)-methyltransferase rlmG	423.494855	0.34478794	0.10809829	3.18957805	0.00413797
Chitobiose-specific 6-phospho-beta-glucosidase ChbF	440.061297	0.34244343	0.11103109	3.08421201	0.00571675
Thiamin biosynthesis protein ThiC	76851.3073	0.34240679	0.11867163	2.88532978	0.01018634
Copper resistance protein D	2402.10842	0.34167312	0.09125566	3.74413091	0.00063673
Cytoplasmic copper homeostasis protein CutC	1075.34878	0.34016216	0.10093103	3.3702436	0.0023147
Two-component sensor protein RcsC	1935.70122	0.33981573	0.08100039	4.19523567	0.00011098
type 1 fimbrae adaptor subunit FimF	799.6109	0.3397525	0.108461	3.13248556	0.00494595
CFA/I fimbrial minor adhesin	1054.8465	0.33969744	0.12626151	2.69042759	0.01746542
FIG004016: Uncharacterized protein YggN	1387.6143	0.33791218	0.10361311	3.26128783	0.00329794
Peptide transport system permease protein SapB	1041.567	0.33731914	0.10932734	3.08540526	0.005704
Epoxyqueuosine (oQ) reductase QueG	775.095764	0.33690589	0.12637408	2.66594139	0.01860097
tRNA(Cytosine32)-2-thiocytidine synthetase	443.43123	0.33643898	0.11517425	2.92113023	0.00919221
Polymyxin resistance protein ArnA_DH%2CUDP-glucuronic acid decarboxylase	5294.69312	0.33638093	0.1112478	3.02370869	0.00684027
Mobile element protein	190.88188	0.33609019	0.13706757	2.45200377	0.03212719
4-hydroxybenzoyl-CoA thioesterase family active site	616.427136	0.33319913	0.10522512	3.16653607	0.00445564
COG1272: Predicted membrane protein hemolysin III homolog	2932.02694	0.33315683	0.11374964	2.9288605	0.00900683
Cytochrome O ubiquinol oxidase subunit II	23176.27	0.33310561	0.09355347	3.56059072	0.00122076
NAD(P)HX epimerase / NAD(P)HX dehydratase	3784.76507	0.33262104	0.09297155	3.5776647	0.00115097
Putative exported protein	707.287277	0.33201466	0.11969349	2.77387397	0.01391003
FIG00638940: hypothetical protein	1473.06837	0.33107877	0.10242057	3.23254187	0.00360762
iron aquisition outermembrane yersiniabactin receptor (FyuA%2CPsn%2Cpesticin receptor)	130122.094	0.33077483	0.09764779	3.38742775	0.00219302
Chitobiose-specific regulator ChbR%2C AraC family	392.952516	0.33053536	0.13856511	2.38541553	0.03760955
Acyl-phosphate:glycerol-3-phosphate O-acyltransferase PlsY	367.995392	0.32983305	0.1356448	2.43159376	0.03376972
6-phosphofructokinase class II	4317.21221	0.32941433	0.09463601	3.48085599	0.00159986
Xylanase	963.331923	0.32924616	0.12808037	2.57062163	0.02378767
Tellurite resistance protein TehA	563.75847	0.32877786	0.13035722	2.52212995	0.02694605
Excinuclease ABC subunit C	6132.2442	0.32607415	0.1091261	2.98804909	0.00756003
FUSARIC ACID RESISTANCE PROTEIN FUSB / FUSARIC ACID RESISTANCE PROTEIN FUSC	407.861608	0.32508032	0.13735365	2.36673965	0.03919686
Electron transport complex protein RnfE	412.468185	0.32490276	0.1227495	2.64687638	0.0195395
tRNA uridine 5-carboxymethylaminomethyl modification enzyme GidA	4353.07502	0.32486187	0.07585705	4.28255348	7.74E-05
Glutaredoxin 2	5344.61717	0.3244624	0.12097337	2.68209766	0.01788785
Lipoprotein YcfM%2C part of a salvage pathway of unknown substrate	5824.28442	0.32353737	0.08772245	3.68819359	0.00078063
Phosphoethanolamine transferase specific for the outer Kdo residue of lipopolysaccharide	504.806238	0.32323065	0.10881741	2.97039462	0.00797665
Queuosine Biosynthesis QueE Radical SAM	572.401609	0.32317987	0.09863298	3.27659022	0.0031421
FIG000988: Predicted permease	3303.68831	0.32241752	0.08282872	3.89258101	0.00036861
Putative transport protein	349.424333	0.32199209	0.13101232	2.45772375	0.03166547
Serine transporter	282.318264	0.32143066	0.13549182	2.37232517	0.03864494
D-mannonate oxidoreductase	1028.93098	0.32096964	0.09847428	3.25942618	0.0033155
3-oxoacyl-[acyl-carrier protein] reductase	13349.813	0.31962255	0.12862082	2.48499856	0.0295949
Lipoprotein	1314.20169	0.31909959	0.09328764	3.42059869	0.00196418
DNA polymerase III delta prime subunit	1659.26073	0.31890676	0.09235806	3.45293924	0.00175831
CFA/I fimbrial auxiliary subunit	2181.09621	0.31782116	0.13153292	2.41628616	0.03502399
Iron-sulfur cluster regulator IscR	8943.60524	0.31748559	0.07639348	4.15592526	0.00012989
Cystine ABC transporter%2C permease protein	1383.8709	0.31315307	0.09498032	3.29703113	0.0029407
L-arabinose transport system permease protein (TC 3.A.1.2.2)	415.227852	0.31110074	0.12923105	2.40732196	0.03572704
Osmolarity sensory histidine kinase EnvZ	2395.70164	0.31056602	0.10179684	3.05084154	0.00633579
Trilactone hydrolase IroD	41158.605	0.31038566	0.09314425	3.33231164	0.00262376
Carbon storage regulator	3754.16296	0.30997824	0.13354714	2.32111477	0.04367217
Lysophospholipid transporter LplT	657.876499	0.30956583	0.11058146	2.79943708	0.01295818
ABC transport system%2C permease component YbhS	6043.51453	0.30907133	0.11651962	2.65252596	0.01925466
23S rRNA (Uracil-5-)-methyltransferase rumB	733.903741	0.30899487	0.10585826	2.91894895	0.00924648
Chitinase	1043.62901	0.30883494	0.10176487	3.03478937	0.00663618
Predicted chaperone lipoprotein YacC%2C potentially involved in protein secretion	627.26191	0.30846147	0.13189243	2.33873514	0.04201047
Adenine phosphoribosyltransferase	1841.04612	0.30845341	0.07928573	3.8904026	0.00037135
Ethanolamine utilization protein EutA	239.4228	0.30737702	0.133299	2.3059215	0.04522189
Putative flagellin structural protein	2713.11846	0.30716829	0.12752222	2.40874328	0.03561932
Inner membrane protein YcdZ	1145.76214	0.30708935	0.1073038	2.86186834	0.01086406
ATP-dependent helicase HrpA	7651.33324	0.30658234	0.11076089	2.76796556	0.01412941
Putative cytoplasmic protein	374.337592	0.30583642	0.13198958	2.31712547	0.04401762
Sensor protein basS/pmrB	987.284301	0.30573668	0.10301052	2.96801424	0.00803413

FIGfam014588: Predicted regulator of CFA/I fimbriae	966.174041	0.3050097	0.12685361	2.40442268	0.035961
regulator of length of O-antigen component of lipopolysaccharide chains	2419.57747	0.30486188	0.11489528	2.65338899	0.01922508
Long-chain fatty acid transport protein	537.032151	0.30458538	0.09525954	3.19742655	0.00403692
Transcriptional regulator	1528.83225	0.30451536	0.11533235	2.64032911	0.01989075
AMP nucleosidase	7567.67671	0.30359718	0.11253328	2.69784343	0.0171254
N-ethylmaleimide reductase	2137.5375	0.30223868	0.10342863	2.92219539	0.00916727
FIG143263: Glycosyl transferase / Lysophospholipid acyltransferase	4049.67301	0.30187614	0.1242491	2.42960428	0.03393952
Cell division protein DivIC (FtsB)%2C stabilizes FtsL against RasP cleavage	321.924739	0.3009649	0.12940801	2.32570537	0.04322031
FIG00613574: hypothetical protein	3211.52402	0.3009002	0.10463384	2.87576369	0.01045414
hypothetical tRNA/rRNA methyltransferase yfiF	3394.17217	0.29988975	0.11669634	2.56982988	0.0238303
Putative cytoplasmic protein	560.873977	0.29867571	0.11826563	2.52546497	0.0267308
Ribosomal small subunit pseudouridine synthase A	1049.17097	0.29859594	0.11570917	2.58057285	0.02319331
Nitrate/nitrite sensor protein	1806.25531	0.29844189	0.09572667	3.11764613	0.00516448
MFS family multidrug transport protein%2Cbicyclomycin resistance protein	964.278441	0.29749426	0.12014679	2.47608994	0.03024154
LysR family transcriptional regulator YnfL	295.435463	0.29509259	0.12987413	2.272143	0.04878527
Peptide transport periplasmic protein SapA	1856.39561	0.29384621	0.09682511	3.03481398	0.00663618
Putative efflux (PET) family inner membrane protein YccS	892.484216	0.29295896	0.11044032	2.65264504	0.01925466
ATP-dependent DNA helicase Rep	805.545419	0.29280865	0.11856685	2.46956588	0.03076904
Formyltetrahydrofolate deformylase	3613.67355	0.29216863	0.07748385	3.77070344	0.00057819
Putative transport protein	397.686897	0.29135514	0.12165555	2.39491866	0.03675159
Uncharacterized ABC transporter%2C ATP-binding protein YrbF	2101.90153	0.29094412	0.10801204	2.6936267	0.01731667
Inner membrane protein yciS	938.140055	0.29046728	0.08616179	3.37118424	0.00230831
Similarity with glutathionylspermidine synthase	368.62656	0.28832231	0.10791502	2.67175332	0.01833502
Formate dehydrogenase O alpha subunit	13603.8677	0.28666828	0.09292727	3.08486712	0.00571094
FIG065221: Holliday junction DNA helicase	5665.86871	0.28634672	0.09283977	3.08431106	0.00571675
Cell division protein FtsK	24451.7864	0.28572242	0.08466003	3.37493886	0.00227854
BigB	3206.54267	0.28532567	0.08634399	3.3045225	0.00287161
Ribonuclease I precursor	1786.83484	0.28378733	0.09370459	3.02853172	0.00675157
AmpG permease	32836.929	0.28377309	0.10778306	2.63281727	0.02026407
3-dehydroquinate synthase	6251.85124	0.27995273	0.07714499	3.62891646	0.00096179
Sulfur carrier protein adenylyltransferase ThiF	33219.022	0.27959807	0.10272659	2.72176917	0.01603342
Chromosome partition protein MukF	2585.48073	0.27922308	0.09003158	3.10139044	0.0054371
Putative membrane protein	8723.76418	0.27893389	0.08811789	3.16546271	0.00446666
tRNA pseudouridine synthase B	4751.00992	0.27860484	0.09412685	2.95988689	0.00820267
Ethanolamine ammonia-lyase heavy chain	533.886705	0.27849742	0.12204888	2.28185148	0.04779459
hypothetical protein	556.314415	0.27807763	0.09979861	2.78638766	0.01340553
Multidrug-efflux transporter%2C major facilitator superfamily (MFS) (TC 2.A.1)	844.616928	0.27363071	0.11085123	2.46844997	0.03083549
Acyl carrier protein (ACP2)	1078.02124	0.27273337	0.10441472	2.61202031	0.02136639
L-threonine transporter%2C anaerobically inducible	591.062102	0.27222451	0.11750891	2.31662873	0.04405575
DNA-cytosine methyltransferase	627.424804	0.27210191	0.1188445	2.28956263	0.04698327
Evolved beta-D-galactosidase transcriptional repressor	582.98649	0.27132547	0.10500501	2.58392883	0.02298027
2-ketobutyrate formate-lyase	862.529361	0.27064599	0.11958511	2.26320821	0.04987048
Flavoprotein MioC	1615.33546	0.26866479	0.10338538	2.59867291	0.02211539
Nitrite-sensitive transcriptional repressor NsrR	2339.71747	0.26798237	0.11012001	2.43354839	0.03360389
Heat shock (predicted periplasmic) protein YciM%2Cprecursor	2468.16918	0.26768666	0.07635586	3.5057774	0.00147427
Cystine ABC transporter%2C ATP-binding protein	1682.55839	0.26667395	0.08350947	3.19333805	0.00408697
FIG000906: Predicted Permease	3893.53459	0.26642234	0.08965724	2.97156531	0.00795081
Qercetin 2%2C3-dioxygenase	2073.12175	0.26623128	0.09344585	2.84904338	0.01127492
Mobile element protein	1534.05738	0.26599006	0.09465493	2.81010249	0.01256334
Formate dehydrogenase O beta subunit	3974.98515	0.26496413	0.10305271	2.5711516	0.02376306
Thymidylate kinase	1506.49509	0.2642448	0.07580595	3.48580555	0.00157376
Putative Diguanylate cyclase/phosphodiesterase domain 1	2327.41617	0.26408434	0.09133719	2.89131244	0.01001153
NADH-ubiquinone oxidoreductase chain K	574.803681	0.26383866	0.11602003	2.27407848	0.04858218
putative integrase	1159.17826	0.26283418	0.09033807	2.90945076	0.00950043
Oligopeptide transport ATP-binding protein OppF (TC 3.A.1.5.1)	7495.48699	0.26191065	0.09332588	2.80640971	0.01269463
Cellulose synthase operon protein C	19219.7329	0.26188971	0.09380823	2.79175622	0.01322028
tRNA (uracil(54)-C5)-methyltransferase	848.62678	0.26053374	0.08984437	2.89983371	0.00975895
Protein-export membrane protein SecD (TC 3.A.5.1.1)	9423.27585	0.26005689	0.08947162	2.90658534	0.00957725
CDP-diacylglycerol--serine O-phosphatidyltransferase	2466.24956	0.25905713	0.09359287	2.76791533	0.01412941
NAD-dependent malic enzyme	16046.4025	0.2584469	0.10476232	2.46698333	0.03093236
Hypothetical transcriptional regulator yeeY	1209.50645	0.25752747	0.08810247	2.92304477	0.00915629
Multidrug transporter MdtC	640.571825	0.25498553	0.09900891	2.57537955	0.0234978
1-acyl-sn-glycerol-3-phosphate acyltransferase	511.896914	0.25420657	0.09411087	2.70113921	0.0169878
YciO family	2406.50512	0.25340799	0.08463074	2.99427819	0.007437
GlpG protein (membrane protein of glp regulon)	665.599845	0.25306256	0.09942081	2.54536801	0.0253783
Cell division protein ZipA	7817.73979	0.25293941	0.10003744	2.52844736	0.02655671
UPF0313 protein ygiQ	1153.89883	0.2519424	0.0999055	2.52180702	0.02695763
Autolysis histidine kinase LytS	668.825893	0.25142129	0.10059674	2.49929851	0.02849579
N-acetylmuramoyl-L-alanine amidase	5040.35228	0.25138355	0.06782473	3.7063701	0.00072979
23S rRNA (guanosine-2'-O-)-methyltransferase rlmB	4999.81924	0.25106673	0.09294446	2.70125534	0.0169878
Universal stress protein E	25370.2603	0.25088383	0.10529085	2.38276946	0.03779291
YcfL protein: an outer membrane lipoprotein that is part of a salvage cluster	3246.88472	0.24931245	0.10493058	2.37597515	0.0384155
MotA/TolQ/ExbB proton channel family protein	2406.52675	0.24852341	0.08002036	3.1057522	0.00536132
Septum site-determining protein MinC	2675.24405	0.24761645	0.08354348	2.96392301	0.00812331
Gluconate utilization system Gnt-I transcriptional repressor	1030.04325	0.2457125	0.07864975	3.12413586	0.00506101
Guanylate kinase	3474.23992	0.24561212	0.07940937	3.09298673	0.00557349
Transcription antitermination protein NusG	5942.19801	0.24336431	0.09227719	2.63731826	0.02003766
ATP-dependent Clp protease ATP-binding subunit ClpX	42568.2943	0.24157378	0.06585087	3.66849788	0.00083825
hypothetical protein	12315.0211	0.23985925	0.09583354	2.5028738	0.028228
Outer membrane lipoprotein carrier protein LolA	5995.13618	0.23486557	0.10235418	2.29463592	0.04640134
D-serine/D-alanine/glycine transporter	5810.42671	0.23456065	0.08255533	2.8412538	0.01152275
FIG021952: putative membrane protein	4553.38993	0.23436916	0.08383072	2.79574303	0.01307922
Galactose operon repressor%2C GalR-LacI family of transcriptional regulators	1666.88374	0.23231814	0.09667714	2.40303076	0.03606433
Glutathione-regulated potassium-efflux system ATP-binding protein	1764.92074	0.23218247	0.09147684	2.53815561	0.02586924
Ribosome small subunit-stimulated GTPase EngC	2368.90925	0.23105138	0.08682551	2.66110013	0.01881908
FIG00641337: hypothetical protein	7032.73013	0.22988496	0.09751014	2.35754932	0.04008822
Proline-specific permease proY	1811.31676	0.22630644	0.08267212	2.73739744	0.01537946
Ribosomal-protein-S5p-alanine acetyltransferase	3241.43576	0.22569111	0.09957551	2.26653235	0.04948387
RND efflux system%2C inner membrane transporter CmeB	830.411561	0.22544292	0.09418966	2.39349958	0.03685962
YpfJ protein%2C zinc metalloprotease superfamily	989.940988	0.2244617	0.09345442	2.40183068	0.03613455
HTH-type transcriptional regulator hdfR	3815.67618	0.22427818	0.09764603	2.29684898	0.04617291
Ribosomal protein L11 methyltransferase	781.787184	0.22198229	0.08942972	2.48219822	0.02975667
Dihydroneopterin triphosphate epimerase	780.680763	0.2216539	0.09167777	2.41774977	0.0349273
Undecaprenyl-diphosphatase	807.537962	0.22163169	0.09158448	2.41996997	0.03478616
SeqA protein%2C negative modulator of initiation of replication	2691.4834	0.21949084	0.08573386	2.5601418	0.02444454
FIG00639052: hypothetical protein	6373.97238	0.21906322	0.07391813	2.96359257	0.00812745
LptA%2C protein essential for LPS transport across the periplasm	4123.19675	0.21517032	0.09267975	2.32165412	0.0436294
Chorismate mutase I	1480.17433	0.21491778	0.09356278	2.2970435	0.04617005
tRNA-i(6)A37 methylthiotransferase	2720.46546	0.21477716	0.08498374	2.52727345	0.02661957
RNA polymerase sigma-54 factor RpoN	8586.78544	0.21375728	0.06437921	3.32028423	0.00273068
Glucose-1-phosphatase	4037.07619	0.2131369	0.0738473	2.88618426	0.01016432
formate dehydrogenase formation protein FdhE	1985.70709	0.21151828	0.07733574	2.73506497	0.01548074
putative collagenase	747.98415	0.21079453	0.09084801	2.32029878	0.04374716
Outer membrane protein A precursor	542418.306	0.21052034	0.07111209	2.96040153	0.0081936
Putative ATPase component of ABC transporter with duplicated ATPase domain	3307.78432	0.21010946	0.08949261	2.34778566	0.04107897
Putative ABC iron siderophore transporter%2C fused permease and ATPase domains	45911.7726	0.20953662	0.09047834	2.31587593	0.04412391
Aerobic respiration control protein arcA	24672.6566	0.20774925	0.08892643	2.33619232	0.04221972

DinG family ATP-dependent helicase YoaA	911.886575	0.20497777	0.0880771	2.32725389	0.04306193
2-acylglycerophosphoethanolamine acyltransferase	1043.16197	0.20431399	0.08886486	2.29915391	0.04595505
RNA methyltransferase%2C TrmH family%2C group 1	793.781465	0.20386478	0.0870399	2.34219906	0.04167995
Polyribonucleotide nucleotidyltransferase	37196.0977	0.20254467	0.07353964	2.75422441	0.01469543
Cytochrome c-type biogenesis protein DsbD%2Cprotein-disulfide reductase	1705.44301	0.19451929	0.08345894	2.33071842	0.0427829
Methionyl-tRNA formyltransferase	3258.28392	0.19004075	0.07104445	2.67495565	0.01817948
Ribosome-binding factor A	2314.61026	0.18803118	0.07500396	2.50695015	0.02796753
ATP-dependent RNA helicase RhlB	7134.78451	0.18790019	0.07908619	2.37589129	0.0384155
23S rRNA (guanine-N-2-)-methyltransferase rlmL	3061.19362	0.1715518	0.0725732	2.36384506	0.03946801
Transcriptional repressor protein TyrR	6634.33207	0.16691769	0.06974584	2.39322799	0.0368697
TldD protein%2C part of TldE/TldD proteolytic complex	5085.49596	0.16213501	0.06334857	2.55941091	0.02448103
Histidyl-tRNA synthetase	5078.13556	-0.1582266	0.06989943	-2.263632	0.04983762
GTP-binding and nucleic acid-binding protein YchF	4228.3023	-0.1588549	0.06500386	-2.443776	0.03275963
YcfP protein: probably an esterase that is part of a salvage cluster	3270.19983	-0.1824076	0.0782891	-2.3299231	0.0428542
Nucleoside-diphosphate-sugar epimerases	5739.8808	-0.1857535	0.08165816	-2.2747694	0.04851607
DNA gyrase subunit A	9762.39172	-0.1951661	0.08552232	-2.2820492	0.04779125
Enoyl-[acyl-carrier-protein] reductase [NADH]	8223.17278	-0.1978668	0.08305672	-2.3823092	0.0378226
Porphobilinogen deaminase	4979.30137	-0.2000888	0.08706178	-2.2982397	0.0460453
Cysteinyl-tRNA synthetase	3208.4783	-0.2011541	0.07130917	-2.8208732	0.01219496
DNA polymerase I	12931.4197	-0.2022139	0.08427621	-2.3994184	0.03635396
Copper homeostasis protein CutF precursor / Lipoprotein NlpE involeved in surface adhesion	1775.24029	-0.2040969	0.08970247	-2.2752648	0.04847485
N6-hydroxylysine O-acetyltransferase	41772.8951	-0.2059647	0.08935738	-2.3049546	0.04531724
LSU ribosomal protein L15p (L27Ae)	27370.024	-0.2106675	0.08105638	-2.5990244	0.0221038
DNA gyrase subunit B	11482.8754	-0.2115486	0.07592106	-2.7864288	0.01340553
Mlr7403 protein	2939.54062	-0.214142	0.09368696	-2.285718	0.04743943
Ribulose-phosphate 3-epimerase	3086.16658	-0.2162756	0.08345118	-2.591642	0.02249382
Mobile element protein	2107.98704	-0.2176853	0.0764149	-2.8487279	0.01127998
HIPA PROTEIN	1489.08532	-0.2192966	0.09633129	-2.2764839	0.04836359
Acriflavine resistance protein A (AcrA)	13550.8262	-0.2235847	0.07928509	-2.8200099	0.01222124
Putative oligoketide cyclase/dehydratase or lipid transport protein YfjG	752.116063	-0.2236995	0.08860664	-2.5246363	0.02676773
3'%2C5'-cyclic-nucleotide phosphodiesterase	3820.27325	-0.22431	0.08756052	-2.5617713	0.02436626
Leucyl/phenylalanyl-tRNA--protein transferase	1048.35614	-0.2244177	0.08763286	-2.5608848	0.02441644
Chaperone-modulator protein CbpM	4088.38849	-0.2254405	0.09386292	-2.4018052	0.03613455
Thymidylate synthase	2042.9388	-0.2268282	0.09427086	-2.4061322	0.03581163
2-ketoaldonate reductase%2C broad specificity	2413.35145	-0.2279321	0.09982916	-2.2832212	0.0477087
Cyclopropane-fatty-acyl-phospholipid synthase	27305.4481	-0.227933	0.08498964	-2.6818913	0.01788964
Magnesium and cobalt efflux protein CorC	9406.67178	-0.2340384	0.1000488	-2.339242	0.04197278
Diaminohydroxyphosphoribosylaminopyrimidine deaminase	7432.10478	-0.234299	0.07741777	-3.0264236	0.00678701
Octanoate-[acyl-carrier-protein]-protein-N-octano yltransferase	729.795002	-0.2346663	0.08604753	-2.7271703	0.01582286
MotA/TolQ/ExbB proton channel family protein	11343.4539	-0.2351049	0.08399115	-2.7991627	0.01296226
RND efflux system%2C inner membrane transporter CmeB	35026.8191	-0.2366077	0.09040924	-2.6170744	0.02107375
Molybdopterin biosynthesis protein MoeA	4230.90846	-0.2379416	0.09116554	-2.6099955	0.02146688
GTPase and tRNA-U34 5-formylation enzyme TrmE	2425.86016	-0.2393377	0.10159312	-2.3558452	0.04025405
Methylenetetrahydrofolate dehydrogenase (NADP+)	5573.07315	-0.2395833	0.07552618	-3.1721889	0.00437515
Putative uncharacterized protein STY3991	1186.6334	-0.2424863	0.08505124	-2.8510615	0.0112097
Spermidine N1-acetyltransferase	4039.7468	-0.2425726	0.09770863	-2.4826121	0.02973645
S-ribosylhomocysteine lyase	9054.46835	-0.2447905	0.0869963	-2.8138038	0.01243293
Two-component response regulator CreB	1798.62026	-0.2454136	0.09165776	-2.6774992	0.01806533
Putative deoxyribonuclease YjjV	1349.1717	-0.2457686	0.09451654	-2.600271	0.0220457
Positive regulator of CheA protein activity (CheW)	11957.3033	-0.2485844	0.10567834	-2.3522734	0.04062401
Protein-L-isoaspartate O-methyltransferase	1595.89952	-0.2489084	0.094553	-2.6324751	0.02027423
Transcriptional regulator NanR	1150.74908	-0.2496248	0.08831035	-2.8266771	0.01202142
Glucose-1-phosphate thymidylyltransferase	2062.11187	-0.2500433	0.09639298	-2.5939986	0.02236249
LSU ribosomal protein L30p (L7e)	5952.56755	-0.2507323	0.09203323	-2.7243667	0.01592447
Cell division protein MraZ	4249.31744	-0.2512863	0.09565315	-2.6270577	0.02051689
Naphthoate synthase	2244.78647	-0.2524654	0.09878299	-2.555758	0.02469367
Survival protein SurA precursor (Peptidyl-prolyl cis-trans isomerase SurA)	17584.8682	-0.2532777	0.10348471	-2.4474888	0.03248596
Glutathione peroxidase	1057.81588	-0.2549809	0.09520997	-2.6780905	0.01804732
DnaJ-class molecular chaperone CbpA	14510.0637	-0.2563423	0.08785396	-2.9178231	0.00926446
Galactitol utilization operon repressor	794.072589	-0.2567867	0.10487126	-2.4485895	0.03240233
7-alpha-hydroxysteroid dehydrogenase	6626.40838	-0.2568606	0.1058306	-2.4270918	0.03415927
Conserved uncharacterized protein CreA	930.370893	-0.2578596	0.09472833	-2.7220957	0.01602595
Mobile element protein	3839.06973	-0.2579403	0.08149127	-3.1652509	0.00446719
iron-chelator utilization protein	3631.49245	-0.2582115	0.09326109	-2.7686948	0.0141106
Aspartate 1-decarboxylase	5890.3536	-0.2584598	0.08610887	-3.0015465	0.00729559
DnaJ-like protein DjlA	964.097778	-0.2586183	0.09485022	-2.7265963	0.01584211
Probable lipoprotein nlpC precursor	1573.47709	-0.2593329	0.09490146	-2.732655	0.01558628
ClpXP protease specificity-enhancing factor / Stringent starvation protein B	3735.75872	-0.2597342	0.09742897	-2.6658825	0.01860097
Exodeoxyribonuclease III	865.51227	-0.2616434	0.10833726	-2.415082	0.03512342
tolB protein precursor%2C periplasmic protein involved in the tonb-independent uptake of group A colicins	14959.4279	-0.2617802	0.08676537	-3.017105	0.00698159
D-alanine--D-alanine ligase A	2761.33557	-0.2624834	0.10438275	-2.5146247	0.02743269
2%2C3%2C4%2C5-tetrahydropyridine-2%2C6-dicarboxylate N-succinyltransferase	23452.6248	-0.2628011	0.09370922	-2.8044313	0.01276596
[Protein-PII] uridylyltransferase	12612.1414	-0.2633829	0.10132331	-2.5994308	0.02208868
Phosphoglycolate phosphatase	3498.72887	-0.2642468	0.10079707	-2.6215717	0.0208291
Signal recognition particle%2C subunit Ffh SRP54 (TC 3.A.5.1.1)	5013.15868	-0.2643335	0.06445016	-4.1013629	0.0001622
3-demethylubiquinol 3-O-methyltransferase	335.406621	-0.2648213	0.11607432	-2.2814808	0.04781965
Queuosine Biosynthesis QueC ATPase	1494.03731	-0.2649906	0.09997044	-2.65069	0.01934978
4-keto-6-deoxy-N-Acetyl-D-hexosaminyl-(Lipid carrier) aminotransferase	2336.5619	-0.2659915	0.08495694	-3.1308973	0.00496678
Stringent starvation protein A	6512.15034	-0.266125	0.0843803	-3.1538757	0.00463091
Probable UDP-N-acetyl-D-mannosaminuronic acid transferase	685.297341	-0.267661	0.10850556	-2.4667956	0.03093374
Fe-S protein%2C homolog of lactate dehydrogenase SO1521	8838.7238	-0.2700473	0.10904019	-2.4765853	0.03021415
FIG000859: hypothetical protein YebC	4030.21506	-0.2701811	0.1095923	-2.4653295	0.03104573
LSU ribosomal protein L5p (L11e)	25334.6466	-0.2703236	0.09610776	-2.8127138	0.01246846
CDP-diacylglycerol--glycerol-3-phosphate 3-phosphatidyltransferase	1894.76795	-0.2708329	0.11738357	-2.3072473	0.04508385
Galactose-1-phosphate uridylyltransferase	3945.52913	-0.2713446	0.10017779	-2.7086302	0.01665627
DNA mismatch repair endonuclease MutH	447.507031	-0.2724359	0.11122306	-2.449455	0.03234001
Topoisomerase IV subunit A	2932.73785	-0.2724889	0.08250794	-3.3025774	0.00288867
SSU ribosomal protein S9p (S16e)	14753.7436	-0.2735351	0.11751935	-2.3275745	0.04304478
rRNA small subunit methyltransferase H	10528.0633	-0.2735443	0.08529216	-3.2071446	0.00391746
Gamma-glutamyl phosphate reductase	10846.4766	-0.2737463	0.08271649	-3.3094526	0.00283129
SgrR%2C sugar-phosphate stress%2C transcriptional activator of SgrS small RNA	1901.82016	-0.274434	0.09343962	-2.9370196	0.00878797
Soluble lytic murein transglycosylase precursor	4989.32919	-0.2747965	0.08008899	-3.4311395	0.00189572
Methionine ABC transporter permease protein	3359.41903	-0.2749518	0.0883238	-3.1129985	0.00524023
Putative phosphatase	470.721451	-0.2755844	0.10598037	-2.6003344	0.0220457
Erythronate-4-phosphate dehydrogenase	2138.38161	-0.2756851	0.07468262	-3.6914218	0.00077179
Cof protein%2C HD superfamily hydrolase	1095.47883	-0.278195	0.09872418	-2.8179016	0.01228195
Outer Membrane Siderophore Receptor IroN	113235.538	-0.2782862	0.09959607	-2.7941485	0.01313687
DJ-1/YajL/PfpI superfamily%2C includes chaperone protein YajL (former ThiJ)%2C parkinsonism-associated protein DJ-1%2C peptidases PfpI%2C Hsp31	3316.68397	-0.278623	0.11419568	-2.4398733	0.03308416
Ferric enterobactin transport ATP-binding protein FepC (TC 3.A.1.14.2)	4803.80158	-0.2787332	0.09420273	-2.958865	0.00822529
UDP-N-acetylglucosamine 4-epimerase) / UDP-glucose 4-epimerase	5067.35277	-0.2794627	0.09915243	-2.8185162	0.01226505
FIG00644695: hypothetical protein	642.386353	-0.2795002	0.11989822	-2.3311453	0.04275374
Multidrug resistance protein B	723.707534	-0.2802052	0.11462622	-2.4445123	0.03270843
Outer membrane protein NlpB%2C lipoprotein component of the protein assembly complex (forms a complex with YaeT%2C YfiO%2C and YfgL)%3B Lipoprotein-34 precursor	12119.2619	-0.28064	0.09389063	-2.9890099	0.00754918
Predicted endonuclease distantly related to archaeal Holliday junction resolvase	920.420226	-0.2811945	0.12109674	-2.322065	0.04360158
Ferredoxin--NADP(+) reductase	3652.99425	-0.2816002	0.12264141	-2.2961264	0.04624015
UDP-N-acetylmuramate--alanine ligase	15910.9944	-0.2847699	0.10549683	-2.6993217	0.01705835
5-methyltetrahydrofolate--homocysteine methyltransferase	8045.49922	-0.284993	0.08467288	-3.3658121	0.00234762

DnaA regulatory inactivator Hda (Homologous to DnaA)	1229.79052	-0.2850937	0.09815583	-2.9045007	0.00963593
MFS superfamily export protein YceL	3962.09903	-0.2851055	0.11313535	-2.5200394	0.02706598
Molybdopterin biosynthesis protein MoeB	1826.41392	-0.2861193	0.09382867	-3.0493801	0.00635551
SSU ribosomal protein S5p (S2e)	25738.2035	-0.286132	0.09236893	-3.0977085	0.00549854
Glutaminyl-tRNA synthetase	9004.14013	-0.286209	0.07272482	-3.9355063	0.00031422
SSU ribosomal protein S2p (SAe)	26786.8348	-0.2864717	0.11435042	-2.5052094	0.0280687
FIG00638135: hypothetical protein	505.808889	-0.2874877	0.09556077	-3.0084279	0.00715654
D-alanine--D-alanine ligase	12137.5166	-0.2876372	0.10734792	-2.6794859	0.01798153
Copper-sensing two-component system response regulator CpxR	5541.76239	-0.2882962	0.08143052	-3.5403949	0.0013108
Aspartate-semialdehyde dehydrogenase	2517.26911	-0.2900492	0.11258426	-2.5762857	0.02344794
Pyridoxal kinase	1906.49434	-0.2905895	0.07766985	-3.7413421	0.00064267
UPF0149 exported protein YgfB	2600.21613	-0.2908241	0.10219735	-2.8457105	0.01138123
Poly(A) polymerase	7992.92476	-0.2910995	0.07818708	-3.7231156	0.00068655
D-alanyl-D-alanine carboxypeptidase	2861.94831	-0.2923351	0.08092426	-3.6124535	0.00102279
Glutamate 5-kinase	9749.521	-0.2927024	0.07383167	-3.9644561	0.00028135
LSU ribosomal protein L23p (L23Ae)	11241.6285	-0.2929001	0.11068038	-2.6463598	0.01955942
Per-activated serine protease autotransporter enterotoxin EspC	7937.26922	-0.293631	0.08231918	-3.5669817	0.00119221
LysR family transcriptional regulator YeiE	897.067785	-0.2948873	0.11001013	-2.6805469	0.01793387
Asparaginyl-tRNA synthetase	12205.1588	-0.294923	0.09028594	-3.2665443	0.00324344
NfuA Fe-S protein maturation	4062.07381	-0.2950787	0.1008744	-2.925209	0.00909792
S-formylglutathione hydrolase	923.362726	-0.2954165	0.11699	-2.5251432	0.02674221
Putative PTS system IIA component yadI	524.275763	-0.2954517	0.10021815	-2.9480859	0.00849848
Aspartokinase	5487.63262	-0.295541	0.0836593	-3.5326733	0.00134409
TldE protein%2C part of TldE/TldD proteolytic complex	7377.69003	-0.2960716	0.06963989	-4.2514651	8.81E-05
Putative sugar kinase%2C PfkB family protein	349.357801	-0.2961578	0.11340551	-2.6114937	0.02138859
COG2110%2C Macro domain%2C possibly ADP-ribose binding module	992.520362	-0.2963698	0.11628888	-2.5485652	0.02518398
Ribosomal RNA small subunit methyltransferase C	1493.83421	-0.2973576	0.10342971	-2.8749724	0.01047464
Phosphatase YidA	3860.64846	-0.2980112	0.09498444	-3.1374736	0.00487138
Universal stress protein F	2878.63643	-0.2997401	0.11928317	-2.512845	0.02755806
Hypoxanthine-guanine phosphoribosyltransferase	1853.33099	-0.2998538	0.09269861	-3.234718	0.00358247
Putative uncharacterized protein YeaK	1410.90239	-0.3000953	0.09589446	-3.1294331	0.00498436
Outer membrane protein X precursor	129907.434	-0.3002757	0.08410934	-3.5700636	0.00117992
Ribonucleotide reductase transcriptional regulator NrdR	2492.44868	-0.3003768	0.09785408	-3.0696402	0.00597838
FIG00639467: hypothetical protein	3662.21219	-0.3010392	0.12921546	-2.3297461	0.04285485
Mannonate dehydratase	488.806554	-0.3021777	0.13227773	-2.2844183	0.04758031
Undecaprenyl diphosphate synthase	4982.44908	-0.3028115	0.07288756	-4.1545021	0.00013059
Cell division protein FtsA	15467.7923	-0.3053641	0.10232829	-2.9841614	0.0076437
Protein acetyltransferase	1229.03185	-0.3062201	0.08698518	-3.5203707	0.00140413
Dephospho-CoA kinase	1779.68326	-0.3071498	0.09249459	-3.3207323	0.00272867
FIG00638276: hypothetical protein	486.81559	-0.3071961	0.1156009	-2.6573853	0.01901804
Putative ABC transporter ATP-binding protein	1716.10139	-0.3075727	0.12758095	-2.4108045	0.03547362
GTP-binding protein EngB	521.370305	-0.3081014	0.12714219	-2.4232823	0.03448691
AmpE protein	3029.55644	-0.3084382	0.08747958	-3.5258312	0.00137739
Putative oxidoreductase	911.5923	-0.3086461	0.12804306	-2.4104866	0.0354853
LSU ribosomal protein L18p (L5e)	15358.5024	-0.3099081	0.09687769	-3.1989625	0.00401794
2-octaprenyl-3-methyl-6-methoxy-1%2C4-benzoquinol hydroxylase	3322.97744	-0.3103687	0.09683559	-3.20511	0.00394038
Citrate:6-N-acetyl-6-N-hydroxy-L-lysine ligase%2Calpha subunit	76161.7677	-0.3108579	0.09304151	-3.3410668	0.00255227
tRNA dimethylallyltransferase	15699.4299	-0.3109299	0.08976904	-3.4636646	0.00169195
LSU ribosomal protein L6p (L9e)	23770.6715	-0.3110457	0.09968409	-3.1203145	0.00512099
FIG00640454: hypothetical protein	2328.8335	-0.3134882	0.08966046	-3.4963932	0.00151879
Putative Transposase	337.844785	-0.3147161	0.12058544	-2.6099012	0.02146688
tRNA pseudouridine 13 synthase	4295.60926	-0.3162966	0.09283138	-3.4072167	0.00205623
D-Galactonate repressor DgoR	762.769131	-0.3184434	0.13209154	-2.4107784	0.03547362
SSU ribosomal protein S6p	12038.946	-0.318695	0.09259872	-3.4416783	0.00182704
D-serine dehydratase	724.248794	-0.3192612	0.11947819	-2.6721298	0.01832387
2-C-methyl-D-erythritol 2%2C4-cyclodiphosphate synthase	1251.24051	-0.3194073	0.11583776	-2.7573674	0.01457029
FIG00638108: hypothetical protein	1971.54058	-0.3201184	0.11347357	-2.8210835	0.01219496
Right origin-binding protein	14211.7958	-0.3212227	0.10597592	-3.0310915	0.00670628
Multimodular transpeptidase-transglycosylase	12363.3692	-0.3219349	0.0757218	-4.2515479	8.81E-05
FIG00613320: hypothetical protein	6990.18518	-0.3229279	0.10225877	-3.1579486	0.00457225
ABC transporter%2C permease protein YnjC	451.970343	-0.3235778	0.13033504	-2.4826618	0.02973645
16S rRNA processing protein RimM	17521.3936	-0.3240351	0.12910168	-2.5099214	0.02774678
LSU ribosomal protein L4p (L1e)	23914.1228	-0.3243957	0.11610765	-2.7939217	0.01313908
UDP-N-acetylmuramoylalanine--D-glutamate ligase	15380.837	-0.3263202	0.08226197	-3.9668421	0.00027909
NADH dehydrogenase	12035.4816	-0.3270771	0.11913501	-2.7454324	0.01504737
L-threonine 3-dehydrogenase	16375.7924	-0.3275232	0.08189969	-3.9990767	0.00024579
Uncharacterized protein conserved in bacteria	1067.32585	-0.3277894	0.12444613	-2.6339867	0.02020462
Oligopeptidase A	9239.22631	-0.328267	0.10996664	-2.9851512	0.00762334
Mannitol operon repressor	1019.41396	-0.3288936	0.10666121	-3.0835348	0.00572639
FIG00896075: hypothetical protein	4879.45363	-0.3289759	0.14131811	-2.3279104	0.04302588
Putative inner membrane protein	510.600002	-0.3304113	0.10365475	-3.1876142	0.00415852
ADP-L-glycero-D-manno-heptose-6-epimerase	6822.11556	-0.3311898	0.08834784	-3.7487033	0.00062663
Murein endopeptidase	2288.03815	-0.3321995	0.08050174	-4.1266131	0.00014587
Cytochrome c heme lyase subunit CcmF	510.060864	-0.3323231	0.11075023	-3.0006535	0.00731231
Deoxyribose operon repressor%2C DeoR family	969.574837	-0.333326	0.11588371	-2.8763833	0.01043937
iron aquisition yersiniabactin synthesis enzyme (Irp1%2Cpolyketide synthetase)	516629.191	-0.3345931	0.10815299	-3.0937013	0.00556671
2-octaprenyl-6-methoxyphenol hydroxylase	4985.73971	-0.335181	0.09775375	-3.4288302	0.00190937
Anaerobic selenate reductase%2C molybdenum cofactor-containing periplasmic protein	456.996806	-0.3352906	0.1458234	-2.2992923	0.04595505
CTP synthase	15597.7853	-0.3362576	0.09633556	-3.4904828	0.00154857
DNA polymerase II	7571.50693	-0.3367476	0.08884088	-3.7904584	0.00053772
Nucleoid-associated protein NdpA	1243.88542	-0.3372719	0.10973975	-3.0733796	0.00591093
Flavodoxin 2	808.993919	-0.337485	0.10385847	-3.2494704	0.00342302
Putative membrane protein YfcA	890.593034	-0.3388928	0.1491285	-2.2724887	0.04876295
Putative inner membrane protein	320.301485	-0.3392838	0.12743938	-2.6623148	0.01876088
Mechanosensitive ion channel	1299.09713	-0.3393357	0.09726937	-3.488618	0.00155836
LSU ribosomal protein L3p (L3e)	27485.1586	-0.3405679	0.10386793	-3.278855	0.00312291
Exported zinc metalloprotease YfgC precursor	3092.94253	-0.3409362	0.08624368	-3.9531734	0.00029353
Aspartyl-tRNA synthetase	11060.8682	-0.3415125	0.09259721	-3.6881507	0.00078063
Iron(III) dicitrate transport system permease protein FecC (TC 3.A.1.14.1)	1169.7792	-0.3422226	0.12229548	-2.7983258	0.01298895
Agmatinase	1409.74021	-0.343383	0.1095432	-3.134681	0.00491205
Predicted ATP-dependent endonuclease of the OLD family%2C YbjD subgroup	6391.72709	-0.3440147	0.1071813	-3.2096521	0.00388825
Phenazine biosynthesis protein PhzF	785.181609	-0.3447095	0.10173959	-3.3881546	0.00218866
Phosphoenolpyruvate-dihydroxyacetone phosphotransferase	537.442452	-0.3460364	0.12173023	-2.8426495	0.01147864
Phosphoglycerate mutase	1423.2534	-0.3462125	0.12256724	-2.8246743	0.01207724
Acetyl esterase	199.841514	-0.346327	0.1300088	-2.6638735	0.01870285
ABC transporter%2C ATP-binding protein	10726.6171	-0.3492743	0.08996078	-3.882517	0.00038151
FKBP-type peptidyl-prolyl cis-trans isomerase FklB	1534.15011	-0.3493673	0.10425777	-3.3509951	0.00247369
6%2C7-dimethyl-8-ribityllumazine synthase	4890.99668	-0.3499203	0.08881859	-3.9397195	0.000309
Multidrug resistance protein A	557.817839	-0.3502201	0.10550415	-3.3194911	0.00273669
Mobile element protein	282.73937	-0.3506751	0.14731733	-2.3804064	0.03800083
Acyl carrier protein phosphodiesterase	1121.9741	-0.3509463	0.11850879	-2.9613526	0.00817294
RelE/StbE replicon stabilization toxin	4296.47377	-0.3510872	0.11465322	-3.0621664	0.00611887
Putative ATP-binding component of a transport system	683.217788	-0.3519237	0.1034742	-3.4010773	0.00209222
Outer membrane lipoprotein SmpA%2C a component of the essential YaeT outer-membrane protein assembly complex	3255.26155	-0.3519519	0.09236081	-3.8106192	0.00050062
Co-activator of prophage gene expression IbrA	5920.16903	-0.3520019	0.09032044	-3.8972562	0.00036185
Membrane protein involved in the export of O-antigen%2C teichoic acid lipoteichoic acids	1118.38624	-0.3531492	0.10687184	-3.3044177	0.00287161
C4-dicarboxylate transporter DcuA	7798.15498	-0.3535362	0.11037757	-3.2029715	0.00396732

3-dehydroquinate dehydratase I	1374.63581	-0.3536093	0.11759781	-3.0069379	0.00718757
Fructokinase	180.027479	-0.3536481	0.14866142	-2.3788828	0.03814055
COG1720: Uncharacterized conserved protein	1337.21246	-0.3537877	0.11759241	-3.0085926	0.00715654
Translation elongation factor Ts	26920.1914	-0.3540894	0.1145859	-3.0901657	0.00561669
Methionine aminotransferase%2C PLP-dependent	1098.22146	-0.3544095	0.1285205	-2.7576103	0.01456717
MltA-interacting protein MipA	1575.45692	-0.3548997	0.10228639	-3.4696672	0.00165905
Ribosome hibernation protein YhbH	10815.096	-0.3570036	0.09901796	-3.6054433	0.00104485
Ferric hydroxamate ABC transporter (TC 3.A.1.14.3)%2C ATP-binding protein FhuC	3790.918	-0.3570092	0.10969937	-3.2544323	0.00337011
Cob(I)alamin adenosyltransferase	1649.13659	-0.3570473	0.08480339	-4.2102946	0.00010447
ATP binding protein	3240.70801	-0.3571888	0.09317087	-3.8336962	0.00045868
TsaD/Kae1/Qri7 protein%2C required for threonylcarbamoyladenosine t(6)A37 formation in tRNA	1479.07952	-0.35838	0.14222103	-2.5198807	0.02706598
SSU ribosomal protein S8p (S15Ae)	12180.3792	-0.3591301	0.09599227	-3.7412404	0.00064267
2%2C5-diketo-D-gluconic acid reductase	2381.50158	-0.3602299	0.13687406	-2.6318346	0.02029198
Periplasmic beta-glucosidase	3202.87372	-0.3613931	0.09506941	-3.8013603	0.00051694
Deoxyguanosinetriphosphate triphosphohydrolase	4360.26534	-0.3617908	0.08753496	-4.1331008	0.00014229
Uncharacterized protein YeaC	766.333768	-0.362571	0.13763964	-2.6342046	0.02020189
Transcription termination protein NusB	9262.7556	-0.3627172	0.10709517	-3.386868	0.00219462
Uracil-DNA glycosylase%2C family 1	1335.18783	-0.3630816	0.14341815	-2.5316296	0.02634275
Putative cytoplasmic protein	1244.54475	-0.3631698	0.11129916	-3.2630053	0.00328008
Chorismate synthase	3631.18405	-0.3637167	0.07522757	-4.8348849	6.60E-06
ShiA homolog	2449.15087	-0.3647944	0.12172581	-2.9968535	0.00738712
Hypothetical transcriptional regulator ygbI	492.660288	-0.3672537	0.12482303	-2.9421947	0.00865698
putative secreted protein	3514.70726	-0.3673657	0.09338114	-3.9340459	0.00031563
Mobile element protein	13976.5067	-0.367447	0.10848023	-3.3872255	0.0021932
LSU ribosomal protein L24p (L26e)	14769.9732	-0.367686	0.10391252	-3.5384183	0.00131791
FIG001674: hypothetical protein	1105.43785	-0.3685432	0.12313314	-2.9930464	0.00746281
Malate dehydrogenase	18605.683	-0.3689096	0.11580519	-3.1856055	0.00418237
Hydrogenase maturation protease	737.261413	-0.3690573	0.12722151	-2.9009036	0.00973645
hemimethylated DNA binding protein YccV	2708.56005	-0.3690924	0.15948905	-2.314218	0.04429848
Sensor protein of zinc sigma-54-dependent two-component system	356.478196	-0.371119	0.12411596	-2.990099	0.00753091
NADP-specific glutamate dehydrogenase	17132.7782	-0.371215	0.10888447	-3.4092556	0.00204227
Cytidine deaminase	4380.01768	-0.3717562	0.08879417	-4.1867182	0.00011464
Tagatose 1%2C6-bisphosphate aldolase	171.632425	-0.3721457	0.16040149	-2.3200889	0.04375168
L-arabinose 1-dehydrogenase	2298.77628	-0.3722207	0.10992797	-3.3860417	0.00219979
ADP-heptose synthase	5275.54093	-0.3724781	0.07270188	-5.1233626	1.63E-06
Phospholipase A1 precursor	880.433637	-0.3731963	0.12779595	-2.9202515	0.00921304
FIG00641015: hypothetical protein	188.363541	-0.3742918	0.16146513	-2.3180971	0.04394402
Riboflavin kinase	6176.34327	-0.3745597	0.06648849	-5.6334512	1.07E-07
Cystathionine gamma-synthase	1875.26389	-0.3751227	0.094994	-3.9489094	0.00029857
FIG01199637: hypothetical protein	1165.89162	-0.3778173	0.10673712	-3.5396991	0.00131335
tRNA:Cm32/Um32 methyltransferase	2260.98464	-0.3785209	0.090504	-4.182367	0.00011645
N-acetylmuramoyl-L-alanine amidase	1969.59214	-0.3796869	0.09519875	-3.9883603	0.00025653
Folate-dependent protein for Fe/S cluster synthesis/repair in oxidative stress	4064.80491	-0.3803773	0.0884024	-4.3027944	7.09E-05
ABC transporter%2C ATP-binding protein YnjD	214.572537	-0.3807725	0.1583926	-2.403979	0.03598779
Uridine kinase	799.962241	-0.3819665	0.10853598	-3.519261	0.00140905
Nucleoside permease NupG	557.977816	-0.3821322	0.1030732	-3.7073868	0.00072793
L-seryl-tRNA(Sec) selenium transferase	1829.73072	-0.382314	0.09013522	-4.2415613	9.17E-05
Lipoprotein signal peptidase	5173.09957	-0.3825253	0.09807793	-3.9002183	0.00035828
D-mannonate oxidoreductase	812.167263	-0.3827309	0.09591034	-3.9905073	0.00025442
Xaa-Pro aminopeptidase	10005.5881	-0.3835397	0.08601848	-4.4588055	3.69E-05
Hydrogenase-2 operon protein hybE	681.096028	-0.3844173	0.15099739	-2.5458536	0.02535548
LSU ribosomal protein L35p	7975.67628	-0.3845645	0.13111008	-2.9331424	0.0088885
Signal transduction histidine kinase CheA	27896.8691	-0.3863103	0.11132217	-3.4702013	0.00165798
Acetate operon repressor	1323.72811	-0.3864467	0.16528149	-2.3381124	0.04204195
Thiamine-monophosphate kinase	2654.58488	-0.3877028	0.09147234	-4.23847	9.29E-05
FIG002649: ydiI hotdog fold superfamily	1691.78352	-0.3885684	0.10347524	-3.7551828	0.00061292
LSU ribosomal protein L14p (L23e)	11574.544	-0.3907354	0.10271872	-3.8039358	0.00051315
mobilization protein	6743.4465	-0.3912103	0.13405884	-2.9181982	0.00925847
Transcription accessory protein (S1 RNA-binding domain)	2943.23074	-0.3923488	0.10877898	-3.6068444	0.00103996
Catalase	21318.9789	-0.3933696	0.11530795	-3.41147	0.00202845
Putative RTX family exoprotein A gene	8498.28803	-0.3935085	0.12847007	-3.0630366	0.0061047
3-hydroxypropionate dehydrogenase	8909.57885	-0.3946542	0.0984443	-4.0089086	0.00023674
Holo-[acyl-carrier protein] synthase	1206.4135	-0.3956497	0.10248069	-3.8607238	0.00041525
Putative membrane protein	1777.66995	-0.3960425	0.08067373	-4.9091882	4.63E-06
Monofunctional biosynthetic peptidoglycan transglycosylase	1666.7005	-0.3962587	0.10199097	-3.885233	0.00037786
L-fucose operon activator	647.622922	-0.3963878	0.12593094	-3.1476606	0.00472476
Homoserine/homoserine lactone efflux protein	270.907244	-0.3966721	0.1460069	-2.7168035	0.01626746
Putative metabolite transport protein yaaU	203.856512	-0.3972223	0.13870028	-2.8638895	0.01080677
Altronate oxidoreductase	244.189336	-0.3978908	0.12366441	-3.2175041	0.00379511
Outer membrane lipoprotein pcp precursor	11535.0785	-0.3985372	0.08966087	-4.44494	3.91E-05
Antigen polymerase O6	823.71633	-0.3996196	0.16258234	-2.4579523	0.03166048
Translation initiation factor 3	12781.073	-0.3999189	0.12331158	-3.243158	0.00349319
Ferric enterobactin-binding periplasmic protein FepB (TC 3.A.1.14.2)	6039.04337	-0.4003344	0.08105444	-4.9390805	4.02E-06
Anaerobic glycerol-3-phosphate dehydrogenase subunit C	136.183287	-0.400751	0.17246921	-2.3236093	0.04344249
DcrB protein precursor	3965.93769	-0.4009647	0.10489656	-3.8224771	0.00047896
Cytochrome c heme lyase subunit CcmL / Cytochrome c heme lyase subunit CcmH	497.570788	-0.4015581	0.10284056	-3.9046671	0.00035205
DNA polymerase IV	5031.07115	-0.4016163	0.10741525	-3.7389129	0.00064816
tRNA (5-methylaminomethyl-2-thiouridylate)-methyltransferase	1846.39333	-0.4026109	0.11600622	-3.4705974	0.00165665
Protein yjbR	2235.75578	-0.402789	0.1081778	-3.7233983	0.00068629
Trehalose operon transcriptional repressor	2205.36294	-0.4031574	0.12390912	-3.253654	0.00337724
Arsenate reductase	1936.57583	-0.4033323	0.11001666	-3.666102	0.00084491
Hydrogen peroxide-inducible genes activator	8595.32211	-0.4034357	0.08816735	-4.5757949	2.19E-05
Alanyl-tRNA synthetase	20485.7708	-0.4036274	0.09257708	-4.3599068	5.57E-05
Succinate-semialdehyde dehydrogenase [NAD]	3079.83271	-0.4038876	0.14796441	-2.7296268	0.01571368
FIG00638802: hypothetical protein	230.5895	-0.4043392	0.16388393	-2.4672292	0.03092597
Exodeoxyribonuclease I	2522.34856	-0.4048029	0.13272255	-3.0499938	0.00634625
Glyoxylate carboligase	430.949682	-0.4058498	0.11236137	-3.6120046	0.00102383
Putative aldolase YdjI	190.939089	-0.4060822	0.15689523	-2.5882377	0.02270607
Anhydro-N-acetylmuramic acid kinase	1602.42558	-0.4064914	0.10097723	-4.0255746	0.00022131
C-terminal domain of CinA type S%3B Protein Implicated in DNA repair function with RecA and MutS	409.859718	-0.408887	0.11762448	-3.4762062	0.00162458
NADPH:quinone oxidoreductase 2	783.144205	-0.4096651	0.13604947	-3.0111479	0.00710092
FIG00640640: hypothetical protein	479.381592	-0.4102648	0.14603357	-2.8093872	0.01258454
Transcriptional repressor for pyruvate dehydrogenase complex	8171.37408	-0.4108438	0.13356246	-3.0760426	0.00586532
PTS system%2C trehalose-specific IIB component	140.712661	-0.4136408	0.17421119	-2.3743642	0.03855689
ABC transporter involved in cytochrome c biogenesis%2C CcmB subunit	87.9194755	-0.4137772	0.18173152	-2.2768599	0.04835929
Aspartate-semialdehyde dehydrogenase	32623.3034	-0.4143485	0.09297485	-4.4565655	3.72E-05
Phage tail length tape-measure protein 1	2231.68324	-0.4144162	0.15758077	-2.6298655	0.02036539
SSU ribosomal protein S10p (S20e)	10892.3848	-0.4144636	0.12542331	-3.304518	0.00287161
FIG001676: Ferredoxin	679.225456	-0.4145989	0.12130552	-3.4178072	0.00198312
Cytochrome c-type biogenesis protein CcmG/DsbE%2Cthiol:disulfide oxidoreductase	258.552342	-0.4154769	0.17170027	-2.41978	0.03478786
Hydroxyacylglutathione hydrolase	2552.10865	-0.4155401	0.13250944	-3.1359282	0.00489416
transposon resolvase	147.120359	-0.4169088	0.16771094	-2.4858772	0.0295362
putative methylase YhhF	656.561454	-0.4179341	0.12518202	-3.3386107	0.00257327
D-erythrose-4-phosphate dehydrogenase	8336.53288	-0.418193	0.12485334	-3.3494739	0.0024857
YgfY COG2938	775.366282	-0.418247	0.14116206	-2.9628857	0.00813694
Ferritin-like protein 2	1684.65506	-0.4185141	0.15692671	-2.6669399	0.01857108
Oligopeptide ABC transporter%2C periplasmic oligopeptide-binding protein OppA (TC 3.A.1.5.1)	247.251848	-0.4187912	0.15623063	-2.6805958	0.01793387

Dihydrodipicolinate reductase	3968.03883	-0.4191391	0.08870473	-4.7251045	1.11E-05
Translation elongation factor P	3381.4252	-0.4195608	0.11599335	-3.6171105	0.00100529
Capsular polysaccharide ABC transporter%2C permease protein KpsM	3097.90302	-0.4208926	0.13376735	-3.1464527	0.00474144
Uncharacterized HTH-type transcriptional regulator YegW	506.691841	-0.4215275	0.13340401	-3.1597815	0.00454912
Pyridoxine 5'-phosphate synthase	2858.70285	-0.4217885	0.09408088	-4.4832538	3.33E-05
Rare lipoprotein A precursor	6180.40413	-0.4218958	0.09161331	-4.6051797	1.92E-05
Respiratory nitrate reductase beta chain	688.28388	-0.4225168	0.1489905	-2.8358639	0.01170581
Protein HI1394	359.173014	-0.4228015	0.13765429	-3.0714735	0.00594529
Orotidine 5'-phosphate decarboxylase	679.689911	-0.4232405	0.12089148	-3.5009949	0.00149586
DNA recombination-dependent growth factor C	1083.11209	-0.4243829	0.08442883	-5.0265168	2.61E-06
Isochorismatase	130.655806	-0.4245157	0.16292279	-2.6056252	0.02172582
Peptidyl-prolyl cis-trans isomerase PpiA precursor	915.523596	-0.424632	0.10979047	-3.8676578	0.00040394
Cell division protein FtsL	2703.74005	-0.4248966	0.148509	-2.8610831	0.01087914
Fructose-1%2C6-bisphosphatase%2C GlpX type	566.817929	-0.4251316	0.10734431	-3.9604489	0.00028565
Adenylosuccinate synthetase	33359.9638	-0.425267	0.09404951	-4.5217357	2.81E-05
probable membrane protein b2001	266.251868	-0.4257499	0.13760076	-3.0940955	0.00556263
Selenide%2Cwater dikinase	9318.44636	-0.4285902	0.11157236	-3.8413652	0.00044664
FIG138517: Putative lipid carrier protein	2320.80274	-0.4298641	0.12543998	-3.4268511	0.00192206
virulence protein	1183.57169	-0.4303761	0.12910522	-3.3335296	0.00261736
Putative oxidoreductase	2255.8551	-0.4306883	0.11350677	-3.7943842	0.00053048
MchC protein	10701.5915	-0.4307964	0.12862472	-3.3492506	0.00248609
putative%3B ORF located using Glimmer/Genemark	880.622547	-0.4323536	0.09263707	-4.6671768	1.45E-05
Transaldolase	2498.29322	-0.4323722	0.12218682	-3.5386159	0.00131783
L%2CD-transpeptidase YcfS	313.785655	-0.4326779	0.11780575	-3.6728083	0.00082483
Transcriptional regulator%2C IclR family	889.670758	-0.4337363	0.1178982	-3.6789053	0.00080595
Serine transporter	438.401018	-0.4340718	0.11887735	-3.6514257	0.00089082
Phosphoenolpyruvate-dihydroxyacetone phosphotransferase	713.463276	-0.434077	0.14587123	-2.9757545	0.00785192
Alcohol dehydrogenase	2120.08953	-0.434155	0.11515253	-3.7702599	0.00057878
FIG00641173: hypothetical protein	438.614978	-0.4342116	0.16982787	-2.5567744	0.02463378
Transcriptional regulator KdgR%2C KDG operon repressor	1892.81092	-0.4342217	0.09889562	-4.3907069	4.89E-05
Ribonuclease E inhibitor RraA	3726.89508	-0.4347548	0.12854078	-3.3822327	0.00222469
Exodeoxyribonuclease VII small subunit	407.878977	-0.4362416	0.12213714	-3.5717356	0.00117323
RNA polymerase sigma factor FecI	7114.8091	-0.4372345	0.14630061	-2.9886036	0.00755062
Transposase YhgA	174.185206	-0.4373926	0.15880268	-2.7543151	0.01469543
FIG00643651: hypothetical protein	870.376157	-0.4381164	0.09950835	-4.40281	4.66E-05
Probable electron transfer flavoprotein-quinone oxidoreductase FixC	244.083996	-0.4382464	0.13541392	-3.2363468	0.00356488
Phosphatidylglycerophosphatase A	770.132585	-0.4382592	0.0994279	-4.4078094	4.57E-05
LSU ribosomal protein L2p (L8e)	27709.2417	-0.4384523	0.1114906	-3.9326394	0.00031723
Putative electron transport protein YsaA	293.91091	-0.4390183	0.1610343	-2.7262406	0.01584665
ATP-dependent helicase HrpB	4642.06157	-0.4403775	0.07263671	-6.0627403	9.28E-09
Periplasmic protein torT precursor	249.82834	-0.44259	0.15326058	-2.8878267	0.01011697
Glutathione reductase	7873.51501	-0.4429776	0.08904551	-4.974733	3.37E-06
LSU ribosomal protein L1p (L10Ae)	32282.6231	-0.4435261	0.141732	-3.1293291	0.00498436
Uncharacterized protein YidR	1104.72427	-0.4435487	0.14043274	-3.1584423	0.00456729
Alkaline phosphatase isozyme conversion protein precursor	1312.24622	-0.4436616	0.11311161	-3.9223347	0.00032836
Methionine repressor MetJ	669.899773	-0.4442721	0.10724378	-4.1426377	0.00013708
6-phospho-beta-glucosidase	5979.87962	-0.4443511	0.09660298	-4.5997662	1.97E-05
Nucleoprotein/polynucleotide-associated enzyme	487.831746	-0.4448987	0.14147083	-3.1448091	0.00476238
Inosine-guanosine kinase	1781.97797	-0.4449501	0.08019035	-5.5486742	1.69E-07
SSU ribosomal protein S17p (S11e)	13333.2109	-0.4454286	0.11889813	-3.7463042	0.00063171
Putative Dihydrolipoamide dehydrogenase	2659.96573	-0.445459	0.11873824	-3.7516056	0.00061988
LSU ribosomal protein L29p (L35e)	9386.66891	-0.445639	0.10325395	-4.3159513	6.70E-05
LysR family transcriptional regulator YafC	1368.68732	-0.4456513	0.13021934	-3.4223129	0.00195314
ATPase involved in DNA repair	6174.65932	-0.4461166	0.1280704	-3.4833701	0.00158669
Phospholipase/carboxylesterase family protein	1059.00684	-0.4462097	0.0903726	-4.9374445	4.05E-06
FIG01200175: hypothetical protein	8088.53157	-0.4464005	0.07511592	-5.9428216	1.86E-08
4'-phosphopantetheinyl transferase	3372.33038	-0.4471931	0.19124315	-2.3383482	0.04203471
Tryptophanyl-tRNA synthetase	5571.00511	-0.4480937	0.08844626	-5.0662815	2.14E-06
1-phosphofructokinase	3673.10449	-0.448706	0.1665807	-2.6936255	0.01731667
Selenocysteine-specific translation elongation factor	2962.16272	-0.449678	0.09543743	-4.7117568	1.18E-05
ABC transporter%2C periplasmic spermidine putrescine-binding protein PotD (TC 3.A.1.11.1)	3330.2767	-0.4506866	0.13381071	-3.3680908	0.00233133
Thiosulfate sulfurtransferase%2C rhodanese	4465.3399	-0.4507924	0.12193509	-3.6969867	0.00075673
Xanthine-guanine phosphoribosyltransferase	1226.43724	-0.4510288	0.1239237	-3.639568	0.00092689
UDP-3-O-[3-hydroxymyristoyl] N-acetylglucosamine deacetylase	87097.9919	-0.4514577	0.0842447	-5.358886	4.76E-07
Ureidoglycine aminohydrolase	1623.74953	-0.4519473	0.10975124	-4.1179244	0.00015123
4-hydroxy-3-methylbut-2-enyl diphosphate reductase	4465.85842	-0.4519569	0.08941053	-5.0548508	2.27E-06
CMP-Kdo synthetase%2C KpsU	3266.25406	-0.4520863	0.14360442	-3.1481364	0.00471994
YbbL ABC transporter ATP-binding protein	870.537	-0.4528619	0.15095423	-2.999995	0.00732394
DNA-damage-inducible protein D	3737.56793	-0.4539219	0.10017657	-4.5312178	2.69E-05
Response regulator of zinc sigma-54-dependent two-component system	1193.80469	-0.4543903	0.11227702	-4.0470465	0.0002028
Molybdenum ABC transporter%2C periplasmic molybdenum-binding protein ModA (TC 3.A.1.8.1)	24706.9082	-0.4545053	0.11599703	-3.9182492	0.00033358
FIG00639301: hypothetical protein	712.21457	-0.4565101	0.15238221	-2.9958225	0.0074079
Peptidyl-prolyl cis-trans isomerase PpiB	3385.7517	-0.4570914	0.108374	-4.2177219	0.00010135
FIG002958: hypothetical protein	886.161149	-0.4583026	0.11081731	-4.1356592	0.00014107
Capsular polysaccharide export system periplasmic protein KpsD	6624.18146	-0.4583811	0.14707656	-3.1166158	0.00517946
FIG00637900: hypothetical protein	6094.20834	-0.4589453	0.15212025	-3.0169903	0.00698159
LSU ribosomal protein L22p (L17e)	11035.4277	-0.4589498	0.13235728	-3.4675071	0.00167131
FIG00639460: hypothetical protein	573.992841	-0.4597091	0.15057741	-3.0529753	0.00629831
Iron(III) dicitrate transport system%2C periplasmic iron-binding protein FecB (TC 3.A.1.14.1)	4113.7456	-0.4603724	0.10773281	-4.2732793	8.04E-05
Putative DNA processing chain A	1040.27484	-0.4608282	0.09401933	-4.9014197	4.80E-06
Molybdopterin biosynthesis Mog protein%2Cmolybdochelatase	2229.74779	-0.4610389	0.08454615	-5.4531027	2.85E-07
DNA primase	227.485165	-0.4622214	0.18608035	-2.4839884	0.02965035
Glycosyl hydrolase YegX%2C family 25	550.017244	-0.4634609	0.19846498	-2.3352277	0.04230937
HtrA suppressor protein	307.596201	-0.4666578	0.16425395	-2.8410749	0.01152297
Phage tail assembly protein	488.321739	-0.4667776	0.17697946	-2.6374675	0.02003766
Enterobactin synthetase component F%2C serine activating enzyme	286044.565	-0.4674963	0.09253864	-5.0519031	2.30E-06
Mobile element protein	120.03684	-0.4698777	0.17696548	-2.655194	0.01913227
FIG00639383: hypothetical protein	283.629433	-0.4699463	0.15197736	-3.0922124	0.00558473
Putative oxidoreductase	3734.53145	-0.4703535	0.10798095	-4.3558934	5.67E-05
FIG01047911: hypothetical protein	641.470189	-0.4703724	0.11270884	-4.1733403	0.00012086
MchD protein	2243.11537	-0.4709233	0.16627164	-2.8322526	0.01182656
Protein ydjA	3368.74144	-0.4709917	0.12450755	-3.7828363	0.00055195
Methylglyoxal synthase	3268.20798	-0.4717094	0.10730838	-4.3958301	4.79E-05
Mlc%2C transcriptional repressor of MalT (the transcriptional activator of maltose regulon) and manXYZ operon	1071.92371	-0.4725245	0.13486029	-3.5038074	0.00148218
hypothetical protein	324.87586	-0.472536	0.16554602	-2.8544089	0.01109829
Transcriptional regulator%2C LacI family	2390.40486	-0.4729113	0.11069269	-4.2722907	8.07E-05
N-acetylmuramic acid 6-phosphate etherase	820.860181	-0.4735778	0.09370171	-5.0541004	2.28E-06
Methyl-accepting chemotaxis protein II (aspartate chemoreceptor protein)	23040.0907	-0.4737204	0.11666938	-4.0603662	0.00019205
2-Keto-3-deoxy-D-manno-octulosonate-8-phosphate synthase	11061.7309	-0.4737644	0.06608328	-7.1692015	7.21E-12
Mediator of hyperadherence YidE	1060.17007	-0.4740934	0.13923798	-3.404914	0.00206954
Ascorbate utilization transcriptional regulator UlaR%2C HTH-type	732.906229	-0.4746337	0.10789468	-4.3990465	4.72E-05
Transport ATP-binding protein CydC	4460.87424	-0.4746821	0.08824567	-5.3790976	4.26E-07
FIG003276: zinc-binding protein	580.661343	-0.475268	0.11472613	-4.1426308	0.00013708
Glucosamine--fructose-6-phosphate aminotransferase [isomerizing]	13851.4019	-0.4756932	0.07833372	-6.0726495	8.78E-09
Bis(5'-nucleosyl)-tetraphosphatase%2C symmetrical	3668.67905	-0.4758718	0.10542281	-4.5139356	2.91E-05
Ribonuclease Z	306.576666	-0.4774074	0.14869066	-3.2107424	0.00387592
Arabinose operon regulatory protein	2083.8588	-0.4775154	0.10451829	-4.568726	2.26E-05

Sensor protein torS	778.743386	-0.4777958	0.13290989	-3.5948856	0.0010843
S-adenosylmethionine decarboxylase proenzyme	4064.66937	-0.4785076	0.16375188	-2.9221504	0.00916727
Cytochrome c-type biogenesis protein CcmC%2Cputative heme lyase for CcmE	129.117235	-0.4785705	0.18795225	-2.5462343	0.0253403
tRNA (Guanine37-N1)-methyltransferase	18174.5618	-0.4788255	0.1358483	-3.5247076	0.00138229
Exodeoxyribonuclease V alpha chain	2450.77665	-0.4789626	0.08475536	-5.6511185	9.76E-08
Transcriptional (co)regulator CytR	1762.3114	-0.4790587	0.09393534	-5.0998771	1.82E-06
Polyferredoxin NapH (periplasmic nitrate reductase)	78.1012794	-0.4794911	0.20729466	-2.3130896	0.04441119
Riboflavin synthase eubacterial/eukaryotic	4126.78363	-0.4797094	0.1261824	-3.8017141	0.00051659
Outer membrane protein H precursor	23397.6032	-0.4798138	0.13287058	-3.6111367	0.00102581
hypothetical protein	1529.34286	-0.4802717	0.10320151	-4.6537277	1.55E-05
ABC transporter%2C periplasmic substrate-binding protein YnjB	789.444579	-0.4821	0.12070583	-3.9940075	0.0002509
Cystathionine beta-lyase	1284.32814	-0.482689	0.10870169	-4.4404919	3.98E-05
Fructose-1%2C6-bisphosphatase%2C type I	7890.05491	-0.4837766	0.07224187	-6.6966234	1.82E-10
Iron(III) dicitrate transport protein FecA	15653.4818	-0.4840072	0.12850542	-3.7664345	0.00058684
Putative transport protein	194.371061	-0.4851756	0.16357836	-2.9660131	0.00807744
Transcription-repair coupling factor	7508.85603	-0.4855394	0.11575108	-4.1946858	0.00011116
Putative transport protein YdjK%2C MFS superfamily	76.892217	-0.4872058	0.20188072	-2.4133347	0.03525899
TorCAD operon transcriptional regulatory protein TorR	609.840314	-0.4873402	0.10891167	-4.4746367	3.45E-05
FMN-dependent NADH-azoreductase	1463.87565	-0.4880808	0.09901894	-4.9291661	4.21E-06
Penicillin-binding protein AmpH	1839.90298	-0.4882716	0.07741782	-6.306967	2.13E-09
Holliday junction DNA helicase RuvB	3272.75552	-0.4894642	0.07840331	-6.2429024	3.13E-09
Periplasmic nitrate reductase precursor	358.650724	-0.4899175	0.16510398	-2.9673268	0.00804756
Capsular polysaccharide export system protein KpsC	5854.55923	-0.4900625	0.17055979	-2.8732595	0.01052583
FIG00640332: hypothetical protein	127.164392	-0.4902008	0.21150259	-2.3177059	0.04396974
ABC-type multidrug transport system%2C permease component	2952.67401	-0.4908989	0.11285851	-4.3496842	5.82E-05
Glutathione S-transferase	4511.97447	-0.4919468	0.11198041	-4.393151	4.84E-05
TIORF127 protein	354.806725	-0.4919508	0.12971643	-3.7925099	0.0005341
SSU ribosomal protein S3p (S3e)	33795.3096	-0.4930963	0.10700551	-4.6081395	1.90E-05
Threonyl-tRNA synthetase	92360.4817	-0.4951913	0.1146257	-4.3200722	6.60E-05
Formamidopyrimidine-DNA glycosylase	604.634534	-0.4972245	0.11341452	-4.3841344	5.03E-05
Xanthine and CO dehydrogenases maturation factor%2CXdhC/CoxF family / Selenium-dependent molybdenum hydroxylase system protein YqeB	409.242346	-0.4978564	0.14054556	-3.5423129	0.00130221
Iron(III) dicitrate transport system permease protein FecD (TC 3.A.1.14.1)	918.825414	-0.4986666	0.11507966	-4.3332301	6.25E-05
Uncharacterized sigma-54-dependent transcriptional regulator YgeV	823.236606	-0.5004602	0.14277622	-3.5052068	0.00147642
Glycerol dehydrogenase	17512.7792	-0.5008253	0.10285621	-4.8691792	5.60E-06
Phage DNA-packaging protein	388.082906	-0.5008703	0.17055574	-2.936696	0.00879222
FIG003671: Metal-dependent hydrolase	82.9734432	-0.501141	0.20827805	-2.4061151	0.03581163
SSU ribosomal protein S16p	2445.46368	-0.5024363	0.17085769	-2.9406712	0.00869479
Capsular polysaccharide export system protein KpsF	10152.441	-0.5027394	0.11297362	-4.4500601	3.83E-05
Dipeptidyl carboxypeptidase Dcp	3758.85244	-0.5029956	0.11359666	-4.4279084	4.21E-05
Glucokinase	2103.58563	-0.5032149	0.0775756	-6.4867677	6.94E-10
Methionine ABC transporter ATP-binding protein	6189.37643	-0.5039581	0.10661797	-4.7267649	1.10E-05
LSU ribosomal protein L16p (L10e)	18696.9938	-0.5045653	0.1032056	-4.8889336	5.09E-06
hypothetical protein	302.911211	-0.5049905	0.1911016	-2.6425235	0.01977235
Adenylosuccinate lyase	4942.58974	-0.5055886	0.13324126	-3.7945344	0.00053048
FIG002842: hypothetical protein	2474.9509	-0.5063496	0.10405014	-4.8664001	5.67E-06
Aerotaxis sensor receptor protein	1996.95781	-0.5063611	0.13077977	-3.8718608	0.00039796
LSU ribosomal protein L11p (L12e)	9784.08794	-0.5064133	0.13925685	-3.6365415	0.00093645
hypothetical protein	2060.79711	-0.5071157	0.11618968	-4.3645499	5.47E-05
Putative cytoplasmic protein	1005.76985	-0.5076572	0.16767222	-3.0276766	0.00676285
Putative ACR protein	705.979496	-0.508784	0.16583308	-3.0680486	0.00600677
Tyrosyl-tRNA synthetase	8411.55489	-0.5093193	0.08355678	-6.0954875	7.66E-09
Ribonucleotide reductase of class Ia (aerobic)%2Cbeta subunit	4606.18513	-0.5103178	0.11669791	-4.3729815	5.28E-05
HigA protein (antitoxin to HigB)	707.123985	-0.5129284	0.11306673	-4.5365101	2.62E-05
Protein yifE	1321.44679	-0.5134958	0.14602547	-3.5164812	0.0014229
FIG004064: hypothetical protein	4026.60282	-0.5139996	0.11683823	-4.3992414	4.72E-05
Phosphoglucosamine mutase	9629.23157	-0.5141156	0.11415319	-4.5037337	3.04E-05
Bacteriophage N4 adsorption protein A	2329.037	-0.5146692	0.10641129	-4.836603	6.55E-06
Outer membrane vitamin B12 receptor BtuB	8545.61909	-0.5153964	0.13383546	-3.85097	0.00043081
FIG00640016: hypothetical protein	669.879632	-0.5160682	0.1419084	-3.636629	0.00093645
Hydrogenase-2 large chain precursor	2334.17481	-0.5173788	0.13085317	-3.9538882	0.00029289
Transcriptional regulator SlyA	1835.25365	-0.5184043	0.13208207	-3.9248649	0.00032532
Acetoacetate metabolism regulatory protein AtoC	179.230368	-0.5186723	0.16666148	-3.1121308	0.00525252
Dihydropteroate synthase	1254.15607	-0.5190929	0.11581451	-4.4821054	3.34E-05
Branched-chain amino acid ABC transporter%2C amino acid-binding protein (TC 3.A.1.4.1)	1353.61729	-0.5191631	0.15316395	-3.3895908	0.00217866
2-amino-3-ketobutyrate coenzyme A ligase	13651.1423	-0.5194126	0.10630762	-4.8859397	5.16E-06
Putative transcriptional regulator of sorbose uptake and utilization genes	335.138127	-0.5215198	0.12810688	-4.0709742	0.00018381
3-hydroxydecanoyl-[ACP] dehydratase	1062.58846	-0.522801	0.13311916	-3.9273161	0.00032272
Phosphodiesterase yfcE	961.783167	-0.5235924	0.1175027	-4.456003	3.73E-05
FrmR: Negative transcriptional regulator of formaldehyde detoxification operon	505.600498	-0.5249336	0.13493694	-3.890214	0.00037135
L-fucose mutarotase	3809.67486	-0.5260364	0.09923652	-5.3008351	6.51E-07
PTS system%2C glucose-specific IIA component	19080.9816	-0.5273279	0.11358432	-4.6426116	1.62E-05
Dihydrodipicolinate synthase	9262.60804	-0.5275699	0.12558764	-4.2008107	0.00010838
Protein-N(5)-glutamine methyltransferase PrmB%2Cmethylates LSU ribosomal protein L3p	2529.54564	-0.5286671	0.08610131	-6.1400583	5.89E-09
LSU ribosomal protein L13p (L13Ae)	9392.62939	-0.5292344	0.10040222	-5.2711428	7.61E-07
Mannitol-1-phosphate 5-dehydrogenase	2658.40689	-0.5295694	0.08928631	-5.9311369	1.99E-08
DNA gyrase inhibitory protein	617.099748	-0.5297689	0.13386144	-3.9575917	0.00028864
L-lysine 6-monooxygenase [NADPH]	139483.052	-0.5311288	0.11817663	-4.494364	3.17E-05
ErpA%2C essential respiratory protein A / probable iron binding protein from the HesB_IscA_SufA family	5851.94694	-0.5323746	0.13797031	-3.858617	0.00041852
Hydroxyethylthiazole kinase	6565.41323	-0.532825	0.13949471	-3.819679	0.00048369
DUF1706 domain-containing protein	4300.91571	-0.5335436	0.10424987	-5.1179312	1.67E-06
FIG00638396: hypothetical protein	1228.21598	-0.5341966	0.17484144	-3.0553204	0.00625294
Exonuclease SbcD	667.041063	-0.5343666	0.14036254	-3.8070457	0.00050752
Hypothetical zinc-type alcohol dehydrogenase-like protein YdjJ	84.694312	-0.53511	0.18907427	-2.8301575	0.01189785
N-acetylmannosamine kinase	108.859999	-0.5359963	0.22928995	-2.3376352	0.04207637
Methionine aminopeptidase	9832.50247	-0.5363208	0.08978363	-5.9734804	1.55E-08
Maltodextrin glucosidase	1540.25758	-0.536794	0.11429439	-4.6965912	1.27E-05
Mobile element protein	303.607557	-0.5382769	0.12115187	-4.442993	3.94E-05
Excinuclease ABC subunit A	36502.1678	-0.5387039	0.10656372	-5.0552282	2.27E-06
Nitrate/nitrite sensor protein	764.767813	-0.5391063	0.13443685	-4.0101079	0.00023574
DNA repair protein RecN	13604.9287	-0.5394997	0.08645189	-6.2404617	3.18E-09
Protein ydgH precursor	5026.57596	-0.5395376	0.07850348	-6.8727857	5.66E-11
FIG00638797: hypothetical protein	2433.17171	-0.5400839	0.12287876	-4.3952583	4.79E-05
FIG00639029: hypothetical protein	357.682568	-0.5408283	0.18857838	-2.8679232	0.01068161
Chromosome segregation ATPase	7268.51676	-0.5410518	0.13628769	-3.9699243	0.00027586
Erythritol transcriptional regulator EryD	4812.48045	-0.5412235	0.08775529	-6.1677595	4.97E-09
FIG002208: Acetyltransferase	1879.76104	-0.5417021	0.12807451	-4.2295853	9.64E-05
Putative inner membrane protein	3144.22697	-0.5471028	0.12217927	-4.4778694	3.40E-05
Type I restriction-modification system%2CDNA-methyltransferase subunit M	2098.15473	-0.5472134	0.12275317	-4.4578356	3.71E-05
Inosine-uridine preferring nucleoside hydrolase	392.046626	-0.5472169	0.14189336	-3.8565366	0.00042177
Fumarate respiration transcriptional regulator DcuR	1060.32429	-0.5473051	0.11171984	-4.8989069	4.86E-06
Proteinase inhibitor I11%2C ecotin precursor	1077.57193	-0.5484334	0.08068905	-6.7968751	9.31E-11
Putative transporting ATPase	706.206567	-0.5489009	0.14912366	-3.6808437	0.00080043
Quinolinate phosphoribosyltransferase [decarboxylating]	2580.41858	-0.5498884	0.08703478	-6.3180307	1.99E-09
Transcriptional repressor of the lac operon	1355.84104	-0.5508021	0.11256139	-4.8933489	4.98E-06
entry exclusion protein 2	1490.89522	-0.5512349	0.12374818	-4.454489	3.75E-05
FIG001826: putative inner membrane protein	167.756387	-0.5521086	0.16804259	-3.2855279	0.00305568
Phage portal protein	428.141828	-0.55251	0.17701335	-3.12129	0.00510712

Ribonucleotide reductase of class Ia (aerobic)%2Calpha subunit	5551.17648	-0.5525486	0.12686074	-4.355552	5.67E-05
Head-tail preconnector protein GP5	2435.35773	-0.553364	0.18072041	-3.0619896	0.00611888
Fur regulated protein ORF x171.28	197.843376	-0.5552938	0.18798159	-2.9539798	0.00835192
entry exclusion protein 2	834.667474	-0.5568993	0.13375299	-4.1636401	0.0001259
Transcriptional activator of maltose regulon%2CMalT	5963.24059	-0.5588519	0.10937053	-5.1097121	1.74E-06
Flavohemoprotein (Hemoglobin-like protein) (Flavohemoglobin) (Nitric oxide dioxygenase)	1126.08788	-0.5618573	0.12745896	-4.4081428	4.56E-05
Ferredoxin reductase	928.931979	-0.5622011	0.10326252	-5.4443866	2.99E-07
Phosphoanhydride phosphohydrolase	810.741998	-0.5630996	0.13009986	-4.3282105	6.37E-05
Phosphomethylpyrimidine kinase	5062.35669	-0.5641394	0.14362187	-3.927949	0.00032245
SSU ribosomal protein S19p (S15e)	3507.0428	-0.566943	0.13066978	-4.3387463	6.10E-05
S-(hydroxymethyl)glutathione dehydrogenase	4508.01248	-0.5675033	0.10956961	-5.179386	1.22E-06
hypothetical protein	165.384667	-0.5675972	0.18045508	-3.1453656	0.00475621
Carbon starvation protein A	13107.6434	-0.5685966	0.11711199	-4.8551523	6.00E-06
Cys-tRNA(Pro) deacylase YbaK	1019.35765	-0.5691285	0.10473131	-5.434177	3.16E-07
Aspartokinase	4104.36463	-0.5699102	0.18423798	-3.0933372	0.00557023
Fumarate reductase subunit D	1030.50564	-0.5700543	0.11365425	-5.0156883	2.75E-06
Pyrroline-5-carboxylate reductase	3349.50432	-0.5712518	0.0964613	-5.9220835	2.09E-08
Putative permease PerM (%3D YfgO)	889.997444	-0.5727061	0.11907949	-4.8094439	7.44E-06
Nickel transport ATP-binding protein NikD (TC 3.A.1.5.3)	49.1094886	-0.574093	0.23315311	-2.4623006	0.03129418
Acetolactate synthase large subunit	5429.01021	-0.5753006	0.08689265	-6.6208193	2.95E-10
YjiD protein	223.133089	-0.5756165	0.16750022	-3.4365117	0.001861
Multidrug resistance protein D	545.239569	-0.5759044	0.12884181	-4.4698566	3.52E-05
hypothetical protein	1819.3547	-0.5779523	0.10786288	-5.3582136	4.77E-07
Methylglyoxal reductase%2C acetol producing	111.813246	-0.5781043	0.21353617	-2.7072896	0.01671498
PTS system%2C fructose-specific IIB component	5216.31723	-0.5784728	0.14207419	-4.071625	0.00018345
Dihydrodipicolinate synthase	374.982389	-0.5826521	0.10518841	-5.5391278	1.78E-07
LrgA-associated membrane protein LrgB	1978.47082	-0.5832103	0.21601419	-2.6998703	0.01703909
6-phosphofructokinase	25614.2684	-0.5835663	0.07778904	-7.5019088	6.60E-13
FIG01200701: hypothetical protein	511.264136	-0.583653	0.18009352	-3.2408327	0.00351744
PTS system%2C fructose-specific IIBC component	199.34071	-0.5838665	0.13217528	-4.4173657	4.40E-05
Holliday junction DNA helicase RuvA	2101.0414	-0.5840377	0.09557186	-6.1109793	6.98E-09
FIG00638471: hypothetical protein	1995.99233	-0.5851605	0.13200375	-4.432908	4.11E-05
Non-specific DNA-binding protein Dps / Iron-binding ferritin-like antioxidant protein / Ferroxidase	10756.2503	-0.5852385	0.12196535	-4.7983999	7.86E-06
DUF1706 domain-containing protein	7065.72547	-0.5860386	0.19566564	-2.9951021	0.00742118
Ferrous iron transport protein A	3754.79905	-0.5863397	0.13289269	-4.4121287	4.49E-05
Ribosyl nicotinamide transporter%2C PnuC-like	33778.2906	-0.5866094	0.08304701	-7.0635821	1.52E-11
D-Lactate dehydrogenase	11885.1133	-0.587441	0.09308523	-6.3107867	2.08E-09
Putative transport protein	1031.53021	-0.5900648	0.10614898	-5.5588364	1.60E-07
Cytochrome c-type biogenesis protein CcmE%2C heme chaperone	146.387475	-0.5914203	0.1655527	-3.5723992	0.0011719
Accessory protein YqeC in selenium-dependent molybdenum hydroxylase maturation	78.9338204	-0.5916393	0.20349843	-2.9073406	0.00955945
Ferredoxin reductase	1129.68803	-0.5917884	0.11846114	-4.995633	3.04E-06
Glucose-6-phosphate 1-dehydrogenase	9540.69505	-0.5940164	0.09287846	-6.3956313	1.24E-09
Putative transport system permease protein	405.132917	-0.5957536	0.12104475	-4.9217636	4.36E-06
Ferrous iron transport peroxidase EfeB	18020.561	-0.5958808	0.15673362	-3.8018701	0.00051659
Putative minor tail protein	972.191993	-0.5975803	0.17676688	-3.3806124	0.00223493
Prolyl-tRNA synthetase	13291.7549	-0.5981949	0.09459244	-6.3239185	1.93E-09
FIG00639467: hypothetical protein	377.001588	-0.599287	0.15844008	-3.7824206	0.00055246
Phosphoserine aminotransferase	21416.0286	-0.6003754	0.08359822	-7.1816767	6.66E-12
Glutathionylspermidine synthase	16954.8424	-0.6004704	0.09831551	-6.1075857	7.12E-09
Probable lipoprotein	130.279924	-0.60157	0.17038011	-3.5307523	0.00135295
3-oxoacyl-[acyl-carrier protein] reductase	2024.96428	-0.6017974	0.09363071	-6.4273507	1.01E-09
Phage minor tail protein	155.938177	-0.6035673	0.22637143	-2.6662699	0.0185986
COG0699: Predicted GTPases (dynamin-related)	755.629367	-0.6035707	0.09397075	-6.4229638	1.04E-09
RNA polymerase sigma factor RpoD	16498.6546	-0.6039855	0.07580066	-7.9680773	1.95E-14
Glucitol operon repressor	367.183334	-0.6071146	0.13184843	-4.6046405	1.93E-05
Transposase ECs0136	185.990575	-0.6075486	0.17543919	-3.4630156	0.0016949
FIG00644994: hypothetical protein	4369.86925	-0.6091182	0.107797	-5.650604	9.76E-08
FIG00642515: hypothetical protein	1488.49911	-0.6092034	0.10691899	-5.6978035	7.52E-08
2'-5' RNA ligase	771.983591	-0.6107706	0.10368865	-5.8904286	2.51E-08
Transaldolase	345.839422	-0.6108917	0.15486844	-3.9445849	0.00030352
Phage minor tail protein	120.440326	-0.6114181	0.20092867	-3.0429612	0.00648126
Antiholin-like protein LrgA	742.6495	-0.6116105	0.14648185	-4.1753333	0.00011992
Phage tail assembly	713.0358	-0.613624	0.16879448	-3.635332	0.00094018
16 kDa heat shock protein B	630.274434	-0.6140035	0.17328456	-3.5433248	0.00129812
Vitamin B12 ABC transporter%2C B12-binding component BtuF	1184.99019	-0.6154026	0.15274663	-4.028911	0.00021837
HtrA protease/chaperone protein	17694.1874	-0.6173889	0.09268019	-6.6614986	2.29E-10
Capsular polysaccharide export system protein KpsS	951.302347	-0.6176979	0.18774635	-3.2900664	0.00301064
Intergenic-region protein	239.633069	-0.619148	0.16963762	-3.6498271	0.00089574
FIG00637865: hypothetical protein	1374.40549	-0.6208781	0.20422594	-3.0401531	0.00653055
DNA polymerase III epsilon subunit	2877.67808	-0.6211801	0.09355811	-6.6395114	2.63E-10
Putative phage protein	62.0301325	-0.6212931	0.25116918	-2.473604	0.03043812
Predicted glutamine amidotransferase	4199.66859	-0.6228765	0.10150527	-6.1363962	6.01E-09
Spermidine synthase	4179.86898	-0.6239152	0.12873914	-4.8463521	6.26E-06
3-demethylubiquinone-9 3-methyltransferase	375.080056	-0.6253969	0.12977439	-4.8191085	7.11E-06
Putative membrane protein YeiH	372.552848	-0.6259942	0.12127594	-5.1617343	1.34E-06
OsmC/Ohr family protein	540.724382	-0.626139	0.13969341	-4.4822373	3.34E-05
Cell division protein FtsZ	51944.9713	-0.6269096	0.11949897	-5.2461505	8.63E-07
FIG00361523: hypothetical protein	3897.38635	-0.6270525	0.08205957	-7.6414301	2.39E-13
Phosphogluconate repressor HexR%2C RpiR family	631.812195	-0.6288285	0.12585342	-4.9964118	3.03E-06
Sigma cross-reacting protein 27A	3920.44544	-0.6302659	0.10298138	-6.1201932	6.62E-09
Transcriptional regulatory protein RtcR	305.327153	-0.6304822	0.16611312	-3.7954989	0.00052891
Ribosome recycling factor	15187.9073	-0.6311384	0.07144842	-8.833483	1.54E-17
Porphobilinogen synthase	8926.48944	-0.6312425	0.09481986	-6.6572808	2.35E-10
Z1226 protein	103.009673	-0.6321083	0.17543422	-3.6031075	0.00105354
Fructuronate transporter GntP	153.247327	-0.6321382	0.16457533	-3.8410264	0.00044691
Ribonuclease E inhibitor RraB	2897.27743	-0.6329538	0.12484646	-5.0698579	2.11E-06
COG1649 predicted glycoside hydrolase	705.072632	-0.6344519	0.13180965	-4.8133952	7.31E-06
FIG00639173: hypothetical protein	806.086854	-0.6352641	0.14563815	-4.3619344	5.52E-05
Aerobic C4-dicarboxylate transporter for fumarate%2C L-malate%2C D-malate%2C succunate%2C aspartate	653.595476	-0.6376153	0.19557856	-3.2601494	0.00330913
FKBP-type peptidyl-prolyl cis-trans isomerase SlpA	2288.79927	-0.6378566	0.10202516	-6.2519538	2.96E-09
Putative amidohydrolase	480.334776	-0.6384275	0.12117509	-5.2686361	7.70E-07
Phage capsid and scaffold	550.749376	-0.6405219	0.16630298	-3.8515357	0.00043015
Sodium:dicarboxylate symporter	19012.3499	-0.640829	0.09196619	-6.9680931	2.95E-11
UPF0246 protein YaaA	1453.58823	-0.6410915	0.11372265	-5.6373245	1.05E-07
Isoleucyl-tRNA synthetase	54149.0254	-0.6412874	0.08794885	-7.2915947	3.01E-12
Sorbitol-6-phosphate 2-dehydrogenase	111.177077	-0.6413636	0.1708843	-3.7532038	0.00061686
Ni/Fe-hydrogenase 2 B-type cytochrome subunit	562.208631	-0.6415966	0.19623619	-3.269512	0.00321771
Adenylate kinase	7079.1485	-0.6420282	0.1128741	-5.6880024	7.94E-08
Formate dehydrogenase N gamma subunit	196.312119	-0.6433647	0.16723809	-3.8469986	0.00043684
Inosine-uridine preferring nucleoside hydrolase	411.859433	-0.6468226	0.14109578	-4.5842804	2.11E-05
Sensory histidine kinase AtoS	146.994387	-0.6469391	0.18604439	-3.4773373	0.00161883
Ribulokinase	161.048649	-0.647233	0.16323841	-3.9649556	0.00028099
UDP-sugar hydrolase	7001.92043	-0.649285	0.09466918	-6.8584616	6.23E-11
Translation initiation factor SUI1-related protein	313.050622	-0.6495874	0.1631889	-3.9805859	0.00026485
Putative cytoplasmic protein	281.822693	-0.6538246	0.14008702	-4.6672749	1.45E-05
Phage tail assembly protein I	137.534383	-0.6594467	0.23179963	-2.8448999	0.01140404
Dihydrolipoamide dehydrogenase of pyruvate dehydrogenase complex	144339.799	-0.6612822	0.10957006	-6.0352453	1.08E-08

6-phosphogluconate dehydrogenase%2Cdecarboxylating	21843.3358	-0.6615538	0.10457963	-6.3258382	1.91E-09
3-oxoacyl-[ACP] reductase	2369.81713	-0.6644401	0.15406392	-4.3127561	6.79E-05
Putative Nudix hydrolase YfcD	1535.6492	-0.6654783	0.0798262	-8.3365897	1.02E-15
Modulator of drug activity B	530.32887	-0.6661292	0.13238966	-5.0315803	2.55E-06
FIG004614: Putative cytoplasmic protein	509.640288	-0.6671579	0.11035967	-6.0453048	1.02E-08
putative regulator PapX protein	1374.20067	-0.6672172	0.12040616	-5.5413877	1.76E-07
Mannose-6-phosphate isomerase	1720.02268	-0.6677546	0.1186438	-5.6282301	1.10E-07
Dihydroorotase	1457.81439	-0.6682734	0.12245064	-5.4574925	2.79E-07
Predicted dye-decolorizing peroxidase (DyP)%2CYfeX-like subgroup	1255.77172	-0.6708771	0.10791513	-6.2167101	3.68E-09
membrane protein yaiZ	319.677932	-0.6718479	0.16295911	-4.1228003	0.00014818
Phage major capsid protein	1752.70801	-0.6728946	0.17118616	-3.9307769	0.00031893
Protein export cytoplasm protein SecA ATPase RNA helicase (TC 3.A.5.1.1)	22613.7524	-0.6745093	0.09576828	-7.0431389	1.76E-11
Amidophosphoribosyltransferase	4159.94331	-0.676398	0.13589757	-4.9772631	3.33E-06
Ribosome hibernation protein YfiA	13170.6609	-0.6764397	0.13152726	-5.1429622	1.47E-06
Ribose-phosphate pyrophosphokinase	12487.8322	-0.6767974	0.09906369	-6.8319428	7.42E-11
Glutamyl-tRNA synthetase	4538.544	-0.6772251	0.09093869	-7.4470507	9.86E-13
Putative acetyltransferase	79.6554176	-0.6774106	0.24010742	-2.8212816	0.01219496
Putative transcriptional regulator of unknown carbohydrate utilization cluster%2C GntR family	118.170678	-0.6775519	0.20926484	-3.2377721	0.00355318
L-arabinose isomerase	367.004771	-0.679353	0.11817864	-5.7485265	5.68E-08
COG2005: N-terminal domain of molybdenum-binding protein	2773.54489	-0.6804642	0.13908776	-4.8923368	5.00E-06
Fumarate reductase subunit C	727.743438	-0.6809939	0.10848344	-6.2773998	2.54E-09
Type I restriction-modification system%2Cspecificity subunit S	1185.15108	-0.6828094	0.13442554	-5.0794617	2.01E-06
Respiratory nitrate reductase alpha chain	1206.90988	-0.6841978	0.17200068	-3.9778785	0.00026723
Nucleoside-specific channel-forming protein Tsx precursor	2421.4595	-0.6883158	0.13028307	-5.2832329	7.13E-07
Tagatose 1%2C6-bisphosphate aldolase	122.833584	-0.6883388	0.18634101	-3.6939738	0.0007652
Putative ribokinase	156.49024	-0.6885595	0.1557436	-4.4211097	4.33E-05
Uncharacterized GST-like protein yghU associated with glutathionylspermidine synthetase/amidase	2670.66433	-0.688909	0.11667564	-5.9044802	2.32E-08
putative lipoprotein	3407.90695	-0.6893312	0.10200342	-6.7579223	1.21E-10
Putative membrane protein	1732.7865	-0.6909647	0.14874971	-4.6451497	1.61E-05
3-dehydro-L-gulonate 2-dehydrogenase	87.8475687	-0.692432	0.21190874	-3.2675955	0.00323549
Thymidine phosphorylase	10975.2719	-0.6934548	0.08886277	-7.8036597	6.92E-14
Ferrous iron transport protein B	42525.8652	-0.6935257	0.11649218	-5.9534102	1.74E-08
Pyridoxamine 5'-phosphate oxidase	1918.26669	-0.6940127	0.08897245	-7.8003108	7.09E-14
Putative integral membrane protein	2184.48493	-0.6952693	0.11950526	-5.8178972	3.79E-08
GGDEF/EAL domain protein YhjH	2752.12066	-0.6955458	0.09740829	-7.1405199	8.83E-12
Anaerobic selenate reductase%2C molybdenum cofactor-containing periplasmic protein	458.154734	-0.6965828	0.12170999	-5.7233001	6.53E-08
Hydrogenase-2 operon protein hybA precursor	880.276404	-0.6981621	0.19873802	-3.5129769	0.00143982
Inorganic pyrophosphatase	9505.86119	-0.6997489	0.10689618	-6.5460605	4.71E-10
Acetolactate synthase small subunit	1920.15187	-0.700107	0.10369821	-6.751389	1.26E-10
iron aquisition regulator (YbtA%2CAraC-like%2Crequired for transcription of FyuA/psn%2CIrp2)	6424.89229	-0.7011212	0.19217271	-3.648391	0.00089881
Putative two-component system sensor kinase	281.18084	-0.7047412	0.12660134	-5.5666174	1.54E-07
Glutaminase	2895.5476	-0.7049992	0.22056392	-3.1963486	0.00404954
SbmA protein	2697.48515	-0.7059441	0.13321107	-5.2994403	6.55E-07
Ferredoxin reductase	758.408028	-0.7071691	0.09597141	-7.3685391	1.71E-12
Flagellar hook-associated protein FliD	17466.2107	-0.7081335	0.12873498	-5.5007078	2.20E-07
FIG01220476: hypothetical protein	710.988409	-0.7089504	0.14482772	-4.8951296	4.94E-06
Transcriptional antiterminator of lichenan operon%2C BglG family	84.2898238	-0.7090924	0.22360198	-3.1712259	0.004387
Alanine transaminase	4736.51157	-0.7126748	0.10470821	-6.8062933	8.76E-11
Multimeric flavodoxin WrbA	122.483044	-0.7140147	0.20710121	-3.4476605	0.00179064
RecA protein	37899.4307	-0.7146759	0.10872922	-6.5729883	3.98E-10
LSU ribosomal protein L19p	5252.92331	-0.7161982	0.137066	-5.2252066	9.65E-07
Phosphoribosylglycinamide formyltransferase	1370.33513	-0.7167725	0.1568705	-4.5691985	2.26E-05
Superoxide dismutase [Fe]	2884.77281	-0.7186732	0.13295301	-5.4054682	3.70E-07
D-galactarate dehydratase	300.082986	-0.7193817	0.16672494	-4.3147819	6.73E-05
FIG001196: Membrane protein YedZ	10569.4392	-0.7198679	0.16619668	-4.3314216	6.29E-05
Aspartate--ammonia ligase	6313.54664	-0.7200157	0.16114906	-4.4680105	3.54E-05
Inosine-5'-monophosphate dehydrogenase	4296.54879	-0.7208249	0.10898463	-6.6140052	3.08E-10
Exopolygalacturonate lyase	832.449183	-0.7212485	0.1543033	-4.6742259	1.41E-05
Oligopeptide ABC transporter%2C periplasmic oligopeptide-binding protein OppA (TC 3.A.1.5.1)	501.008023	-0.7215966	0.11512809	-6.267772	2.69E-09
YehQ protein	525.331651	-0.7229668	0.13766597	-5.251601	8.42E-07
Hypothetical flavoprotein YqcA (clustered with tRNA pseudouridine synthase C)	501.235472	-0.7232397	0.18927764	-3.821052	0.00048137
PTS system%2C fructose-specific IIA component	29.4332081	-0.7233484	0.3035451	-2.3830016	0.03778665
Putative transcription factor	470.839016	-0.724352	0.12845873	-5.6387917	1.04E-07
FIG138576: 3-oxoacyl-[ACP] synthase	3798.55028	-0.7278099	0.1650583	-4.4094111	4.54E-05
Aminoacyl-histidine dipeptidase (Peptidase D)	53580.5849	-0.729466	0.11021728	-6.6184361	3.00E-10
Aspartate aminotransferase	42884.9332	-0.7300578	0.08864338	-8.2358977	2.30E-15
Transcriptional regulator%2C IclR family	179.746015	-0.7302565	0.19635653	-3.7190332	0.00069671
PTS system%2C glucitol/sorbitol-specific IIB component and second of two IIC components	40.9983805	-0.7312097	0.24761779	-2.9529771	0.00836968
FIG00640497: hypothetical protein	163.350279	-0.7317069	0.18385604	-3.9797816	0.00026553
Uracil permease	371.912185	-0.7348375	0.16808457	-4.3718321	5.30E-05
probable membrane protein yjeI	1778.27197	-0.7357762	0.16448878	-4.4731086	3.47E-05
Crotonobetaine carnitine-CoA ligase	2034.15831	-0.7371352	0.09060194	-8.1359758	5.14E-15
Formate dehydrogenase N beta subunit	187.249855	-0.7372416	0.17832936	-4.1341571	0.00014175
Inhibitor of vertebrate lysozyme precursor	3179.05665	-0.7373069	0.11127323	-6.6260943	2.87E-10
FIG00637942: hypothetical protein	5105.33105	-0.7382446	0.12163648	-6.0692699	8.94E-09
Outer membrane protein F precursor	110512.768	-0.7414895	0.14557497	-5.0935234	1.88E-06
FIG001943: hypothetical protein YajQ	5513.31829	-0.7439005	0.10463626	-7.109395	1.10E-11
Homoserine O-succinyltransferase	1289.00869	-0.7465429	0.15216865	-4.9060232	4.69E-06
Phosphoheptose isomerase 1	8537.45359	-0.7485099	0.1170686	-6.3937717	1.25E-09
Uxu operon transcriptional regulator	2758.95483	-0.7491761	0.08585173	-8.7263944	3.90E-17
Succinate dehydrogenase iron-sulfur protein	1756.59016	-0.7511252	0.1020519	-7.3602276	1.82E-12
Cytochrome c551 peroxidase	203.022158	-0.7514343	0.13627018	-5.5142972	2.04E-07
D-serine permease DsdX	129.438752	-0.7516291	0.23329589	-3.2217845	0.00374351
Twin-arginine translocation protein TatE	535.801272	-0.7519424	0.22981415	-3.2719585	0.00319201
TonB-dependent receptor%3B Outer membrane receptor for ferric enterobactin and colicins B%2C D	102178.665	-0.7546478	0.12722852	-5.9314358	1.98E-08
Inner membrane transport protein YbaT	1836.10873	-0.7552866	0.20186239	-3.7415916	0.00064267
Thiamin ABC transporter%2C transmembrane component	2699.25795	-0.7553822	0.13566942	-5.5678148	1.53E-07
S-adenosylmethionine synthetase	21223.8462	-0.755624	0.12421912	-6.0829927	8.26E-09
DUF1440 domain-containing membrane protein	35902.9513	-0.7560478	0.13822643	-5.4696327	2.61E-07
Deoxyribose-phosphate aldolase	5244.64917	-0.7561967	0.09540015	-7.9265782	2.71E-14
Protein yihD	1157.22962	-0.7578382	0.15302874	-4.9522607	3.77E-06
PTS system%2C glucose-specific IIB component	17074.4119	-0.7582137	0.13659074	-5.550989	1.67E-07
Putative translation initiation inhibitor YoaB	318.637153	-0.7582152	0.15390743	-4.9264367	4.26E-06
Capsular polysaccharide ABC transporter%2CATP-binding protein KpsT	4633.73979	-0.7595521	0.1300942	-5.8384779	3.37E-08
UPF0265 protein YeeX	12917.4174	-0.759713	0.09841632	-7.7193803	1.31E-13
FIG000875: Thioredoxin domain-containing protein EC-YbbN	6097.65464	-0.7625969	0.1151509	-6.6225873	2.92E-10
hypothetical protein	89.465888	-0.7630408	0.29438239	-2.5920054	0.02248126
Uridine phosphorylase	13133.0057	-0.7665297	0.09433753	-8.1253953	5.58E-15
ATPase component STY3233 of energizing module of queuosine-regulated ECF transporter	130.771498	-0.7672177	0.22362635	-3.430802	0.00189681
Phage minor tail protein	119.039088	-0.7691993	0.25424793	-3.0253907	0.00680628
[NiFe] hydrogenase metallocenter assembly protein HybG	192.927319	-0.7703008	0.16586596	-4.644116	1.61E-05
Transcriptional regulator%2C TetR family	1003.90423	-0.7723908	0.14313589	-5.3962059	3.89E-07
Ribokinase	1564.17296	-0.7740285	0.12267893	-6.3093846	2.10E-09
Adenylate-forming enzyme	121.868583	-0.7742295	0.22790215	-3.3972014	0.00212035
Glutamate synthase [NADPH] small chain	368.848422	-0.7766201	0.16478747	-4.7128591	1.18E-05
FIG00638119: hypothetical protein	32.1716019	-0.7790345	0.34016705	-2.2901527	0.04693149
N-acetylneuraminate lyase	48.313844	-0.7799044	0.2573651	-3.0303424	0.00671903

Glucitol operon GutQ protein	1038.01513	-0.7808409	0.12431805	-6.280994	2.49E-09
Putative tail component of prophage CP-933K	290.507037	-0.7813304	0.1864365	-4.1908662	0.00011277
GMP synthase [glutamine-hydrolyzing]%2Camidotransferase subunit	6879.12571	-0.7818537	0.10700582	-7.3066469	2.69E-12
Ferrous iron-sensisng transcriptional regulator FeoC	2974.43105	-0.7845046	0.1251663	-6.2676988	2.69E-09
DNA-binding protein stpA	417.465223	-0.785216	0.14098829	-5.5693706	1.52E-07
FIG00637875: hypothetical protein	2753.68557	-0.7880233	0.12516037	-6.2961089	2.27E-09
2-dehydro-3-deoxygluconate kinase	1211.55146	-0.7887393	0.10522484	-7.4957516	6.88E-13
ElaA protein	198.719651	-0.7925041	0.15483644	-5.1183309	1.67E-06
Flagellar biosynthesis protein FliS	5038.68363	-0.7940072	0.13682267	-5.8031844	4.13E-08
Glucose-6-phosphate isomerase	19873.3771	-0.794497	0.09323376	-8.521559	2.23E-16
Putative glycosyltransferase	3441.35457	-0.7946634	0.15335898	-5.181721	1.21E-06
Crotonobetainyl-CoA:carnitine CoA-transferase	1182.52456	-0.7962161	0.10741721	-7.4123699	1.26E-12
Microcin H47 precursor (MccH47)	7139.49881	-0.7972781	0.16228159	-4.9129302	4.54E-06
Flagellar biosynthesis protein FlgN	2347.11634	-0.7992549	0.16203232	-4.9326882	4.14E-06
Phosphotransferase system%2C phosphocarrier protein HPr	4634.93856	-0.8002022	0.11725788	-6.8242941	7.78E-11
Mg(2+) transport ATPase protein C / Putative membrane transporter ATPase%2CYhiD	418.511397	-0.8005957	0.22353344	-3.5815478	0.00113639
Protein RcsF	1963.87212	-0.8013106	0.09729285	-8.2360685	2.30E-15
FIG00638561: hypothetical protein	59573.3661	-0.8035496	0.12797449	-6.2789825	2.52E-09
Succinate dehydrogenase flavoprotein subunit	6207.57121	-0.8052269	0.09637255	-8.3553554	8.77E-16
Cytosine deaminase	3002.55558	-0.8055191	0.09067083	-8.8839946	1.00E-17
Flagellar hook-associated protein FlgK	28672.4617	-0.8055729	0.12024789	-6.6992682	1.80E-10
Maltose/maltodextrin ABC transporter%2C substrate binding periplasmic protein MalE	166.191356	-0.8057041	0.17281834	-4.6621447	1.49E-05
FIG00639949: hypothetical protein	1447.79929	-0.8102318	0.10512487	-7.7073273	1.44E-13
Acetyltransferase	69.495084	-0.810283	0.2461751	-3.2914905	0.00299734
Alkylphosphonate utilization operon protein PhnA	943.806078	-0.8138981	0.10771114	-7.5563042	4.46E-13
HTH-type transcriptional regulator zntR	387.949333	-0.8144927	0.13976946	-5.8274012	3.59E-08
Ferritin-like protein 2	787.011421	-0.8151552	0.1360732	-5.9905641	1.41E-08
DNA damage-inducible gene in SOS regulon%2Cdependent on cyclic AMP and H-NS	1936.87201	-0.8191319	0.16547963	-4.9500466	3.81E-06
Pyridoxal kinase	956.314286	-0.8193114	0.12435753	-6.5883538	3.63E-10
Beta-lactamase	4972.40445	-0.8195321	0.10374715	-7.899322	3.33E-14
3-methyl-2-oxobutanoate hydroxymethyltransferase	10114.6854	-0.8209079	0.08329561	-9.8553551	1.33E-21
ATP synthase B chain	16574.3802	-0.8222572	0.10291178	-7.9899232	1.64E-14
probable lipoprotein	139.906647	-0.8230559	0.19425758	-4.2369308	9.35E-05
Phosphoenolpyruvate carboxykinase [ATP]	5381.8725	-0.8241228	0.11882772	-6.9354425	3.68E-11
Ribose operon repressor	959.914668	-0.8243733	0.22502554	-3.6634654	0.00085242
Exonuclease SbcC	3181.02415	-0.8250379	0.10317984	-7.9961159	1.56E-14
L-aspartate oxidase	28456.9246	-0.8254377	0.09277091	-8.8975924	9.00E-18
NADPH-dependent broad range aldehyde dehydrogenase YqhD	1931.13865	-0.8263203	0.12830497	-6.4402833	9.29E-10
Putative carbohydrate PTS system%2C IIA component	65.293698	-0.8263629	0.21022843	-3.9307856	0.00031893
putative oxidoreductase%2C Fe-S subunit	24.8635513	-0.8274914	0.34983505	-2.3653759	0.03932336
Pyruvate kinase	10515.5544	-0.8291813	0.09242035	-8.9718472	4.77E-18
iron aquisition yersiniabactin synthesis enzyme (Irp1%2Cpolyketide synthetase)	630816.169	-0.8299795	0.16250929	-5.1072744	1.76E-06
Phosphogluconate dehydratase	1163.40018	-0.8302091	0.12776193	-6.4980943	6.45E-10
Uptake hydrogenase small subunit precursor	2031.17911	-0.8306742	0.1883467	-4.4103465	4.53E-05
putative phage tail component	191.803016	-0.8311612	0.1827525	-4.5480156	2.49E-05
Glycosyl transferase	3667.98848	-0.8357256	0.13149656	-6.3554938	1.59E-09
ATP-dependent protease La	42001.1901	-0.839767	0.10207738	-8.2267694	2.47E-15
Dihydropyrimidine dehydrogenase [NADP+]	185.743136	-0.8417651	0.16257841	-5.1775947	1.23E-06
Head decoration protein	384.888392	-0.8443084	0.19545338	-4.3197432	6.60E-05
Methionine ABC transporter substrate-binding protein	32457.6997	-0.8446042	0.12930306	-6.5319743	5.16E-10
2'%2C3'-cyclic-nucleotide 2'-phosphodiesterase	2924.60413	-0.8483472	0.09776875	-8.6770794	5.97E-17
FIG00637878: hypothetical protein	1645.53574	-0.8483623	0.12470673	-6.8028592	8.95E-11
Hypothetical response regulatory protein yehT	208.535473	-0.8539571	0.16783362	-5.0881172	1.93E-06
Prophage Clp protease-like protein	725.079716	-0.8557881	0.18828962	-4.5450626	2.52E-05
Phosphoglycerate mutase	107207.163	-0.8564986	0.11287963	-7.5877167	3.53E-13
Phosphomannomutase	8328.49607	-0.8579443	0.11813931	-7.2621402	3.72E-12
Universal stress protein A	19263.6564	-0.8584209	0.16817389	-5.1043651	1.78E-06
Transcriptional regulator%2C ArsR family	380.904507	-0.8589934	0.25382697	-3.384169	0.00221195
Ascorbate-specific PTS system%2C EIIA component	138.002614	-0.8595443	0.1926917	-4.4607229	3.66E-05
Iron(III) dicitrate transport ATP-binding protein FecE (TC 3.A.1.14.1)	2087.66463	-0.8608431	0.13653457	-6.3049459	2.15E-09
3'-to-5' oligoribonuclease (orn)	1256.53821	-0.8651858	0.11138398	-7.7675964	9.14E-14
hypothetical protein	8120.81178	-0.8655432	0.1451592	-5.9627172	1.65E-08
isochorismate synthase	27034.0573	-0.8665131	0.11746	-7.3770914	1.62E-12
Transcription repressor	1703.60074	-0.8694254	0.11463465	-7.5843156	3.62E-13
FxsA protein	560.387633	-0.8695151	0.12402089	-7.0110378	2.19E-11
Cytochrome c-type biogenesis protein CcmD%2Cinteracts with CcmCE	19.4358601	-0.8697045	0.36489726	-2.3834228	0.03777276
Pectin degradation protein KdgF	99.7416508	-0.8748724	0.24519269	-3.5681015	0.00118796
Alkaline phosphatase like protein	163.483214	-0.8776959	0.18580769	-4.7236789	1.12E-05
Glycosyltransferase	5178.38349	-0.8801766	0.19694454	-4.4691596	3.53E-05
YdcH protein	289.915377	-0.880202	0.13862697	-6.3494282	1.65E-09
PTS system%2C fructose-specific IIB component	40.253626	-0.8802431	0.26773483	-3.2877423	0.00303367
Putative acetyltransferase	46.5444539	-0.881935	0.23696005	-3.7218723	0.00068943
COG3311: Predicted transcriptional regulator	36.4909425	-0.8835206	0.30271673	-2.9186383	0.00925055
UDP-N-acetylmuramate:L-alanyl-gamma-D-glutamyl-meso-diaminopimelate ligase	4404.78235	-0.8866047	0.09996823	-8.8688644	1.14E-17
Phage integrase	523.539123	-0.8886089	0.12802827	-6.940724	3.56E-11
ATP synthase delta chain	17395.3979	-0.8912079	0.11066927	-8.0528942	9.99E-15
Proposed lipoate regulatory protein YbeD	973.351122	-0.8931768	0.22516529	-3.9667607	0.00027909
5'-methylthioadenosine nucleosidase) / S-adenosylhomocysteine nucleosidase	11410.9673	-0.8935651	0.0897125	-9.9603193	4.78E-22
S-formylglutathione hydrolase	2611.32056	-0.8956661	0.12630262	-7.0914289	1.25E-11
Ornithine carbamoyltransferase	117.202211	-0.8973838	0.18890999	-4.7503247	9.88E-06
Di/tripeptide permease YjdL	777.234332	-0.8984885	0.12052314	-7.454904	9.31E-13
Putative sulfite oxidase subunit YedY	30933.8719	-0.8987239	0.19107568	-4.7034972	1.23E-05
Acetate kinase	12021.9028	-0.8990623	0.10108775	-8.8938799	9.28E-18
Anaerobic glycerol-3-phosphate dehydrogenase subunit B	172.216948	-0.8993051	0.21458842	-4.1908371	0.00011277
Anthranilate synthase%2C aminase component	108868.46	-0.9046772	0.1404387	-6.4417941	9.23E-10
Lysyl-tRNA synthetase (class II)	12716.5739	-0.9053224	0.10371969	-8.7285496	3.84E-17
phage lysozyme	62.4755594	-0.9078062	0.32204474	-2.8188821	0.01225767
Glycosyl transferase	3744.8085	-0.908994	0.10980411	-8.2783237	1.64E-15
Antigen 43 precursor	4813.36371	-0.9101245	0.11590456	-7.852362	4.77E-14
putative membrane protein	1311.617	-0.9138477	0.14494264	-6.3048922	2.15E-09
Glutamate-1-semialdehyde aminotransferase	18941.676	-0.9173393	0.10242379	-8.9563112	5.46E-18
Mutator mutT protein (7%2C8-dihydro-8-oxoguanine-triphosphatase)	71.3922194	-0.9191457	0.21956221	-4.1862654	0.00011473
NAD(P) transhydrogenase subunit beta	17115.9526	-0.9203687	0.10874513	-8.4635397	3.60E-16
N-acetylneuraminate lyase	74.3730135	-0.9210005	0.22883664	-4.024707	0.00022195
Transcriptional repressor protein TrpR	611.664357	-0.9229115	0.15881227	-5.8113361	3.94E-08
Putative oxidoreductase YdjL	63.2373681	-0.928804	0.26472613	-3.5085468	0.00146101
Ferrous iron transport periplasmic protein EfeO%2Ccontains peptidase-M75 domain and (frequently) cupredoxin-like domain	22287.3535	-0.9294107	0.15324762	-6.0647645	9.18E-09
FIG00640624: hypothetical protein	2200.91333	-0.9305631	0.1256903	-7.4036186	1.34E-12
Mannose-1-phosphate guanylyltransferase (GDP)	7123.98936	-0.9306196	0.15932808	-5.840901	3.33E-08
4-carboxymuconolactone decarboxylase domain/alkylhydroperoxidase AhpD family core domain protein	120.532799	-0.9323447	0.25314513	-3.6830442	0.00079412
FIG00638599: hypothetical protein	68.100731	-0.937126	0.23411996	-4.00276	0.00024219
Iron(III) dicitrate transport system permease protein FecD (TC 3.A.1.14.1)	694.935596	-0.937271	0.19895035	-4.7110796	1.19E-05
FIG00638822: hypothetical protein	184.267461	-0.9379444	0.27893665	-3.3625714	0.0023738
4-hydroxy-2-oxoglutarate aldolase	5879.85184	-0.9395892	0.10518249	-8.9329434	6.65E-18
Flagellar transcriptional activator FlhD	1412.34979	-0.9404393	0.13555009	-6.9379464	3.62E-11
Formate dehydrogenase N alpha subunit	435.946106	-0.940863	0.14293188	-6.582597	3.76E-10
Oxygen-insensitive NAD(P)H nitroreductase	6937.72836	-0.9413836	0.09473671	-9.9368406	6.02E-22

Pantoate--beta-alanine ligase	22615.0832	-0.9437318	0.078415	-12.035093	1.02E-31
ATP synthase alpha chain	62760.3827	-0.9451954	0.10330275	-9.1497606	9.61E-19
Type I restriction-modification system%2Crestriction subunit R	7497.6189	-0.9464091	0.10958592	-8.6362291	8.35E-17
Probable L-ascorbate-6-phosphate lactonase UlaG	751.546211	-0.9473057	0.12057116	-7.8568182	4.63E-14
5%2C10-methylenetetrahydrofolate reductase	1959.3245	-0.9496418	0.15784613	-6.0162499	1.21E-08
Predicted L-lactate dehydrogenase%2C hypothetical protein subunit YkgG	634.461694	-0.9533656	0.11916062	-8.0006766	1.51E-14
NAD(P) transhydrogenase alpha subunit	17242.0969	-0.9579689	0.10605671	-9.0326104	2.75E-18
Anaerobic glycerol-3-phosphate dehydrogenase subunit A	327.233189	-0.9588478	0.36014476	-2.6623956	0.01876088
Transaldolase	54249.8019	-0.9591348	0.09865058	-9.7225465	4.63E-21
unnamed protein product	701.258186	-0.9642534	0.16504691	-5.8422987	3.30E-08
ATP synthase C chain	7069.93501	-0.9701908	0.11950543	-8.1183823	5.88E-15
COG1457: Purine-cytosine permease and related proteins	555.295772	-0.9704124	0.14738736	-6.5840949	3.73E-10
Thiamin ABC transporter%2C ATPase component / Thiamine transport ATP-binding protein thiQ	645.014497	-0.9816628	0.1293732	-7.5878376	3.53E-13
Flagellar hook-associated protein FlgL	33750.2902	-0.9824188	0.13240364	-7.4198775	1.20E-12
Threonine efflux protein	181.996642	-0.9840057	0.14793478	-6.6516182	2.44E-10
Protein slyX	70.2440498	-0.9843337	0.32016074	-3.0744984	0.00589229
UDP-glucose 4-epimerase	6562.1415	-0.9843476	0.15005774	-6.5597925	4.34E-10
Formate dehydrogenase N alpha subunit	68.8116181	-0.9889401	0.21605597	-4.5772404	2.18E-05
hypothetical protein	2409.11241	-0.9890318	0.16760645	-5.9009171	2.37E-08
Putative phosphatase YfbT	5623.21238	-0.9893618	0.09829899	-10.064822	1.73E-22
iron aquisition yersiniabactin synthesis enzyme (YbtT%2Cresembles thioesterases)	39518.5996	-0.9913393	0.19152435	-5.176048	1.24E-06
ATP synthase gamma chain	30669.6641	-0.99307	0.1188988	-8.3522292	8.95E-16
Pseudouridine 5'-phosphate glycosidase	148.556396	-0.9940891	0.26220801	-3.7912234	0.00053647
predicted 4-deoxy-L-threo-5-hexosulose-uronate ketol-isomerase	1301.97823	-0.9942326	0.1442568	-6.8921025	4.95E-11
Pyruvate kinase	15123.3875	-0.9985469	0.11261051	-8.8672623	1.16E-17
Inosine-uridine preferring nucleoside hydrolase	578.988554	-0.9988781	0.15175756	-6.5820644	3.77E-10
hypothetical protein	530.900555	-1.0017897	0.18615248	-5.3815545	4.21E-07
Regulator of L-galactonate catabolism YjjM	673.23131	-1.0059044	0.15132542	-6.6472933	2.50E-10
Quinolinate synthetase	32323.3081	-1.0073013	0.08997557	-11.195276	1.26E-27
Pyridine nucleotide-disulphide oxidoreductase family protein	161.592452	-1.0091447	0.16332452	-6.1787702	4.64E-09
Uracil phosphoribosyltransferase	1387.96285	-1.019439	0.11278905	-9.0384568	2.62E-18
D-lactate dehydrogenase	42034.2148	-1.0210123	0.09959574	-10.251567	2.64E-23
Nitric oxide-dependent regulator DnrN or NorA	402.394937	-1.021076	0.12692351	-8.0448136	1.06E-14
FIG00644506: hypothetical protein	52.00571	-1.0216386	0.25518323	-4.0035492	0.00024158
hypothetical protein	193.652728	-1.0234515	0.17232085	-5.9392203	1.89E-08
Uridylate kinase	5282.86592	-1.0242671	0.08998291	-11.382907	1.59E-28
FIG00637894: hypothetical protein	1218.70268	-1.0272044	0.15654958	-6.5615277	4.29E-10
iron aquisition yersiniabactin synthesis enzyme (YbtT%2Cresembles thioesterases)	5257.52753	-1.0311181	0.13671614	-7.5420366	4.96E-13
Yersiniabactin synthetase%2C thiazolinyl reductase component Irp3	54084.7191	-1.0345386	0.18547271	-5.5778481	1.46E-07
Fructose repressor FruR%2C LacI family	7838.37512	-1.0380157	0.10776951	-9.6318126	1.09E-20
2%2C3-bisphosphoglycerate-independent phosphoglycerate mutase	6721.74224	-1.0562157	0.10199038	-10.356033	9.11E-24
Putative sugar phosphotransferase component II B	205.128768	-1.058052	0.17481796	-6.0523069	9.80E-09
16 kDa heat shock protein A	1265.54197	-1.0593331	0.14397256	-7.3578824	1.85E-12
Predicted L-lactate dehydrogenase%2C Iron-sulfur cluster-binding subunit YkgF	1627.77603	-1.0596108	0.12500407	-8.4766103	3.25E-16
Phosphoenolpyruvate-protein phosphotransferase of PTS system	41189.7354	-1.0608385	0.10768813	-9.8510257	1.38E-21
Phosphoglycerate kinase	30660.5316	-1.0657327	0.0980684	-10.867238	4.52E-26
2%2C3-dihydroxybenzoate-AMP ligase	51465.4003	-1.0662271	0.13115805	-8.1293296	5.42E-15
Predicted L-lactate dehydrogenase%2C Fe-S oxidoreductase subunit YkgE	666.395954	-1.0662612	0.13799641	-7.7267315	1.24E-13
Cytochrome d ubiquinol oxidase subunit I	76661.3109	-1.0674433	0.12773667	-8.356592	8.70E-16
Ribose ABC transport system%2C high affinity permease RbsD (TC 3.A.1.2.1)	2251.21857	-1.0706353	0.19225028	-5.568966	1.52E-07
Flagellar transcriptional activator FlhC	3519.84159	-1.0718637	0.1478916	-7.2476305	4.14E-12
PTS system%2C glucitol/sorbitol-specific IIA component	18.4469072	-1.0750805	0.42954874	-2.5028138	0.028228
Proofreading thioesterase in enterobactin biosynthesis EntH	7679.78937	-1.0769941	0.15560726	-6.921233	4.05E-11
Xanthine dehydrogenase%2C molybdenum binding subunit	395.850679	-1.0781613	0.12440131	-8.6668005	6.47E-17
FIG00644121: hypothetical protein	56.2658844	-1.0794807	0.22578073	-4.7811022	8.55E-06
Crotonobetainyl-CoA dehydrogenase	700.184882	-1.0794919	0.12973666	-8.3204534	1.16E-15
Flagellar biosynthesis protein FliT	2199.74199	-1.0807748	0.18292702	-5.9082291	2.27E-08
Phosphoribosylformylglycinamidine cyclo-ligase	1373.13846	-1.08239	0.18193891	-5.9491947	1.79E-08
Galactose/methyl galactoside ABC transport system%2C D-galactose-binding periplasmic protein MglB (TC 3.A.1.2.3)	430.6944	-1.0853611	0.23784799	-4.5632555	2.32E-05
Periplasmic binding protein	1455.4393	-1.0859404	0.23471142	-4.6267045	1.75E-05
Maltose/maltodextrin transport ATP-binding protein MalK	41.6052246	-1.0918028	0.3105574	-3.5156233	0.00142653
glycosyl transferase%2C family 2	13953.8827	-1.0919188	0.19984769	-5.4637546	2.69E-07
2%2C3-dihydro-2%2C3-dihydroxybenzoate dehydrogenase	19012.8824	-1.0949913	0.14778609	-7.4092986	1.29E-12
Galactoside O-acetyltransferase	1159.1178	-1.1018288	0.13079988	-8.4237752	5.00E-16
Ureidoglycolate hydrolase	77.3862262	-1.1071182	0.21258934	-5.2077787	1.05E-06
Nitrate/nitrite transporter	350.096686	-1.1208751	0.18316212	-6.1195794	6.63E-09
putative exported protein	285.472007	-1.1252917	0.17947532	-6.2698965	2.66E-09
ATP synthase epsilon chain	12670.01	-1.1257868	0.10985096	-10.248312	2.71E-23
[NiFe] hydrogenase metallocenter assembly protein HypF	1291.04056	-1.1333179	0.16174437	-7.0068458	2.25E-11
hypothetical protein	144.3357	-1.1339635	0.180987	-6.2654413	2.73E-09
ATP synthase beta chain	70487.0861	-1.1340289	0.11543813	-9.8236944	1.78E-21
Aspartate ammonia-lyase	4986.22374	-1.1389443	0.10749774	-10.595053	7.78E-25
Aerobactin siderophore receptor IutA @ TonB-dependent siderophore receptor	590617.871	-1.139244	0.13508231	-8.4337021	4.62E-16
Putative transcriptional regulator LYSR-type	654.346241	-1.1398044	0.10221443	-11.15111	2.07E-27
putative inner membrane protein	1686.16092	-1.1400053	0.17778812	-6.4121571	1.11E-09
Dethiobiotin synthetase	849.979715	-1.1427399	0.12363291	-9.2430076	4.12E-19
Predicted oxidoreductases (related to aryl-alcohol dehydrogenases)	481.006833	-1.1451211	0.11687651	-9.7977015	2.28E-21
2-keto-3-deoxy-D-arabino-heptulosonate-7-phosphate synthase I alpha	2659.58275	-1.1452772	0.14453173	-7.9240541	2.76E-14
Xanthine dehydrogenase%2C FAD binding subunit	105.683894	-1.1470272	0.26202087	-4.3776176	5.17E-05
Ferrichrome transport ATP-binding protein FhuC (TC 3.A.1.14.3)	791.635487	-1.1480847	0.18952558	-6.0576769	9.52E-09
[NiFe] hydrogenase metallocenter assembly protein HypE	859.389549	-1.1498063	0.16748907	-6.8649628	5.96E-11
Alcohol dehydrogenase	211264.871	-1.1522051	0.15740174	-7.3201548	2.44E-12
Transcriptional activatory protein CaiF	143.103537	-1.1537702	0.20820474	-5.5415176	1.76E-07
Universal stress protein C	1277.4882	-1.1539431	0.11746241	-9.8239349	1.78E-21
ATPase component STY3232 of energizing module of queuosine-regulated ECF transporter	152.86009	-1.1566762	0.17271598	-6.6969839	1.82E-10
Conserved protein YcjX with nucleoside triphosphate hydrolase domain	2906.369	-1.1635065	0.17664118	-6.5868359	3.66E-10
Isochorismatase	45322.5748	-1.1645007	0.16564319	-7.0301752	1.92E-11
iron aquisition 2%2C3-dihydroxybenzoate-AMP ligase	58812.1241	-1.1647376	0.18226218	-6.3904515	1.27E-09
Transcriptional activator GadE	866.062142	-1.169052	0.32105251	-3.6413109	0.00092191
ATP-dependent DNA helicase RecQ	1053.8697	-1.1693861	0.13687542	-8.5434338	1.85E-16
FIG007491: hypothetical protein YeeN	1137.04634	-1.1715037	0.16824663	-6.9630142	3.04E-11
Pseudouridine kinase	138.149252	-1.1721222	0.321169	-3.6495497	0.00089606
ID=gene:EBG00000313248	2650.77754	-1.1739386	0.48557289	-2.4176363	0.0349273
Periplasmic nitrate reductase component NapD	12.1507945	-1.1741212	0.48534459	-2.4191496	0.03481523
ATP-dependent protease HslV	2906.91559	-1.1757197	0.15697178	-7.4900071	7.18E-13
Lactaldehyde reductase	442.750967	-1.1780602	0.15026382	-7.8399461	5.25E-14
Ribose ABC transport system%2C periplasmic ribose-binding protein RbsB (TC 3.A.1.2.1)	3887.12953	-1.1796904	0.1327403	-8.8872058	9.78E-18
Ferrichrome-iron receptor	7759.56562	-1.1826812	0.30385275	-3.892284	0.00036877
FIG00638244: hypothetical protein	175.93131	-1.1830087	0.23507545	-5.0324636	2.54E-06
radical activating enzyme	71.5766343	-1.1855747	0.19746218	-6.0040599	1.30E-08
FIG00638989: hypothetical protein	34.6913486	-1.1914858	0.34599728	-3.4436276	0.00181513
2-deoxy-D-gluconate 3-dehydrogenase	168.650182	-1.1942782	0.19668873	-6.0719196	8.81E-09
LemA PROTEIN	23.6354143	-1.1978424	0.36021486	-3.3253554	0.00268666
Cytochrome d ubiquinol oxidase subunit II	705.140726	-1.2006894	0.14337745	-8.3743251	7.53E-16
DedA family inner membrane protein YdjX	45.0203657	-1.2116324	0.31975999	-3.7891935	0.00054005
L-asparaginase	639.166659	-1.2236809	0.14596282	-8.3835114	6.98E-16
FIG00638505: hypothetical protein	85.6809179	-1.2280357	0.31367181	-3.9150339	0.00033779

Glycerol-3-phosphate transporter	352.609722	-1.2291024	0.27378131	-4.4893584	3.24E-05
5-methyltetrahydropteroyltriglutamate--homocysteine methyltransferase	43458.7226	-1.2303332	0.21652854	-5.6820832	8.21E-08
FIG00639332: hypothetical protein	51.5818151	-1.2308355	0.33325333	-3.6933929	0.00076639
Putative transcriptional regulator LYSR-type	143.985093	-1.2339943	0.17582285	-7.0183954	2.08E-11
Serine hydroxymethyltransferase	21406.4358	-1.2343475	0.13584189	-9.086649	1.70E-18
Starvation sensing protein RspA	118.667064	-1.2372024	0.20913629	-5.9157709	2.17E-08
Ribose ABC transport system%2C permease protein RbsC (TC 3.A.1.2.1)	1345.98052	-1.2414093	0.15865299	-7.8246824	5.90E-14
UDP-glucose dehydrogenase	15376.6264	-1.2440693	0.22097902	-5.6298074	1.09E-07
Hydrogenase maturation factor HoxQ	551.475224	-1.2446937	0.18782042	-6.6270417	2.85E-10
Negative regulator of flagellin synthesis FlgM	4400.90498	-1.2455908	0.17643777	-7.0596608	1.56E-11
C4-dicarboxylate transporter DcuC (TC 2.A.61.1.1)	325.971088	-1.2558195	0.17514193	-7.170296	7.17E-12
Starvation sensing protein RspB	166.281223	-1.257775	0.17163185	-7.3283311	2.30E-12
Flagellar biosynthesis protein FliZ	4135.06083	-1.2619874	0.17110826	-7.3753738	1.64E-12
Asparagine synthetase [glutamine-hydrolyzing]	24221.0963	-1.2652467	0.12852523	-9.844345	1.46E-21
Phosphoribosylformylglycinamidine synthase%2Csynthetase subunit	16410.018	-1.2735741	0.17248376	-7.3837332	1.54E-12
Transthyretin-like protein precursor	13937.3026	-1.2772737	0.17087524	-7.4748907	8.03E-13
FIG00497792: hypothetical protein	20902.7046	-1.2841057	0.17426117	-7.368857	1.71E-12
Putative S-transferase	269.758337	-1.2918613	0.22512148	-5.7385075	5.99E-08
FIG00642340: hypothetical protein	147.557116	-1.2920925	0.17196356	-7.5137574	6.09E-13
FIG00639870: hypothetical protein	23.9112943	-1.2942199	0.34804452	-3.718547	0.00069754
ATP-dependent hsl protease ATP-binding subunit HslU	15966.9262	-1.2957303	0.1696432	-7.6379737	2.44E-13
Cobalt-zinc-cadmium resistance protein CzcA%3B Cation efflux system protein CusA	2118.47922	-1.2963044	0.18568822	-6.9810805	2.69E-11
Membrane protein YcjF	2101.55318	-1.2997248	0.18110924	-7.1764689	6.88E-12
Mgl repressor and galactose ultrainduction factor GalS%2C HTH-type transcriptional regulator	213.861324	-1.3015499	0.25671189	-5.0700803	2.11E-06
Colicin I receptor precursor	21280.5404	-1.3043087	0.14948114	-8.7255739	3.92E-17
putative TYPE II DNA MODIFICATION ENZYME (METHYLTRANSFERASE)	11361.7719	-1.3071092	0.15476773	-8.4456182	4.18E-16
Hemin ABC transporter%2C permease protein	349.337141	-1.3096752	0.23219285	-5.6404629	1.03E-07
Ribose ABC transport system%2C ATP-binding protein RbsA (TC 3.A.1.2.1)	3807.33002	-1.325209	0.12113883	-10.939589	2.07E-26
ID=gene:EBG00000313245	19.0759164	-1.3447316	0.55977415	-2.4022752	0.03612197
Fructose-bisphosphate aldolase class II	43862.0208	-1.3515659	0.11275433	-11.98682	1.76E-31
FIG139928: Putative protease	170.364307	-1.3536416	0.19804808	-6.8349139	7.28E-11
TonB-dependent receptor%3B Outer membrane receptor for ferrienterochelin and colicins	38712.8388	-1.3593104	0.18205909	-7.4663147	8.56E-13
Protein YbgE	5304.47916	-1.3623197	0.12771302	-10.667039	3.75E-25
Thiamin ABC transporter%2C substrate-binding component	8806.71072	-1.3669115	0.12874021	-10.617596	6.15E-25
iron aquisition yersiniabactin synthesis enzyme (Irp2)	246902.571	-1.3677015	0.14460654	-9.4580887	5.64E-20
GMP reductase	8918.44178	-1.3733495	0.10064646	-13.645283	1.40E-40
FIG00638136: hypothetical protein	33.4930278	-1.3748678	0.37568786	-3.6596014	0.00086475
Phosphate acetyltransferase	21329.2997	-1.3827116	0.11746341	-11.771424	2.06E-30
Substrate-specific component STY3230 of queuosine-regulated ECF transporter	279.580733	-1.3951248	0.19869548	-7.0214217	2.04E-11
Protease VII (Omptin) precursor	28248.8443	-1.3967664	0.22768381	-6.1346762	6.06E-09
Fe-S-cluster-containing hydrogenase components 2	356.546573	-1.4011545	0.20070582	-6.9811355	2.69E-11
FIG00639580: hypothetical protein	22.9755941	-1.4017522	0.36518715	-3.8384488	0.00045059
Enolase	71945.7575	-1.401809	0.1179138	-11.888422	5.27E-31
Transmembrane component STY3231 of energizing module of queuosine-regulated ECF transporter	180.477813	-1.402531	0.22113686	-6.3423663	1.72E-09
Predicted regulator of STY3230 transporter operon	93.1158159	-1.4077721	0.24783759	-5.6802204	8.28E-08
Triosephosphate isomerase	15016.6843	-1.4118327	0.10050839	-14.046914	5.60E-43
hypothetical protein	4323.41263	-1.4347294	0.14328611	-10.013039	2.86E-22
Ribose 5-phosphate isomerase A	3175.30358	-1.4455073	0.10351222	-13.964606	1.76E-42
FIG00639275: hypothetical protein	1045.89512	-1.4485318	0.18553381	-7.8073736	6.74E-14
FIG00638031: hypothetical protein	702.085389	-1.4488245	0.12892081	-11.238097	7.99E-28
Heat shock protein GrpE	6002.77915	-1.4513678	0.15833471	-9.1664537	8.26E-19
Pyruvate dehydrogenase E1 component	319982.434	-1.4673961	0.12056037	-12.171463	2.01E-32
Antigen 43 precursor	54.183052	-1.4693224	0.28784929	-5.104485	1.78E-06
Hydrogenase maturation factor HoxO/HyaE	196.959532	-1.4773382	0.18397656	-8.0300351	1.19E-14
Orotate phosphoribosyltransferase	216.69626	-1.508217	0.17177556	-8.7801603	2.46E-17
Zinc resistance-associated protein	392.823658	-1.5110135	0.18341365	-8.2382827	2.26E-15
Cytochrome d ubiquinol oxidase subunit II	58392.4652	-1.5159703	0.15064202	-10.063396	1.75E-22
ABC-type hemin transport system%2C ATPase component	455.343536	-1.52356	0.35898999	-4.2440181	9.08E-05
FIG00638665: hypothetical protein	230.055399	-1.5250281	0.183743	-8.2997886	1.37E-15
Phosphoribosylglycinamide formyltransferase 2	2113.65444	-1.5380769	0.14398799	-10.681981	3.25E-25
Coenzyme F420 hydrogenase maturation protease	663.161144	-1.5482387	0.15457655	-10.016	2.79E-22
Transketolase	30213.8432	-1.5622125	0.11925473	-13.099795	1.82E-37
Hydrogenase maturation protease	377.487827	-1.5649638	0.14566177	-10.74382	1.69E-25
Outer membrane porin protein NmpC precursor	5355.32072	-1.5667823	0.1691544	-9.2624393	3.46E-19
Antigen 43 precursor	4100.61348	-1.5755222	0.13717845	-11.485201	5.29E-29
Membrane transporter HdeD%2C H-NS repressed	2005.49366	-1.5923316	0.2043024	-7.7939936	7.44E-14
FIG00641652: hypothetical protein	42.5877417	-1.6014037	0.27692017	-5.7829075	4.64E-08
Lactose permease	2258.59686	-1.6045063	0.13138298	-12.212437	1.23E-32
Antigen 43 precursor	10437.7359	-1.6121418	0.17326041	-9.3047329	2.37E-19
L-carnitine/gamma-butyrobetaine antiporter	73.1551113	-1.6135152	0.21729859	-7.4253368	1.15E-12
Dihydrolipoamide acetyltransferase component of pyruvate dehydrogenase complex	236492.361	-1.6137373	0.10912573	-14.787871	1.60E-47
Cytochrome d ubiquinol oxidase subunit I	877.0189	-1.6185591	0.15297898	-10.580271	9.02E-25
hypothetical protein	125.030477	-1.6192867	0.67229484	-2.408596	0.03561932
FIG00638118: hypothetical protein	262.545524	-1.6225105	0.15971083	-10.159051	6.71E-23
Channel-forming transporter/cytolysins activator of TpsB family	268.074274	-1.632357	0.1374394	-11.876922	5.95E-31
Aspartokinase	73370.8844	-1.6443375	0.18421707	-8.9260864	7.05E-18
hypothetical protein	169.16983	-1.6520822	0.15965471	-10.347845	9.88E-24
[NiFe] hydrogenase metallocenter assembly protein HypD	731.583945	-1.6606258	0.18588298	-8.9337166	6.63E-18
RNA polymerase sigma factor for flagellar operon	12096.4154	-1.6717938	0.15422398	-10.840038	6.05E-26
Malonyl CoA-acyl carrier protein transacylase	44793.9903	-1.6878773	0.17408314	-9.6958118	5.97E-21
FIG00510943: hypothetical protein	77.6700069	-1.7022088	0.22891918	-7.4358506	1.07E-12
Microcin M imunity protein McmI	304.651409	-1.7146995	0.18533224	-9.2520304	3.80E-19
Tryptophan-specific transport protein	17687.7792	-1.733181	0.30253953	-5.7287756	6.33E-08
RND efflux system%2C inner membrane transporter CmeB	2273.02058	-1.7429131	0.20247393	-8.6080862	1.07E-16
IMP cyclohydrolase	7921.27921	-1.7540856	0.17865276	-9.8184077	1.86E-21
Channel-forming transporter/cytolysins activator of TpsB family	2762.30118	-1.7613472	0.14798891	-11.901886	4.56E-31
Alpha-aspartyl dipeptidase Peptidase E	325.801427	-1.7621268	0.15652848	-11.257547	6.49E-28
Multidrug resistance protein MdtE	931.180602	-1.7659681	0.21563453	-8.1896347	3.32E-15
Probable glutamate/gamma-aminobutyrate antiporter	11322.0329	-1.7779415	0.31954394	-5.5639969	1.56E-07
Nitrite reductase [NAD(P)H] large subunit	2705.79571	-1.7818928	0.22996391	-7.7485758	1.05E-13
Phosphoribosylamine--glycine ligase	11410.7306	-1.7969817	0.15740635	-11.416196	1.11E-28
Ni%2CFe-hydrogenase I cytochrome b subunit	487.816901	-1.815933	0.15841142	-11.463397	6.66E-29
Carbamate kinase	57.3154047	-1.8244707	0.28242995	-6.4599052	8.22E-10
Protein YjgF%2C putative endoribonuclease L-PSP	13474.6349	-1.8257228	0.17107848	-10.671844	3.58E-25
Protein YjgK%2C linked to biofilm formation	476.040231	-1.8309906	0.19758906	-9.2666594	3.34E-19
Chaperone protein DnaJ	9259.38364	-1.8678395	0.17934634	-10.414707	5.00E-24
Homoserine kinase	29302.701	-1.8692009	0.16607775	-11.254975	6.64E-28
Anthranilate synthase%2C aminase component	18852.2887	-1.8706441	0.48791499	-3.833955	0.00045855
Malonyl CoA-acyl carrier protein transacylase	25289.8642	-1.8842381	0.30745833	-6.1284341	6.29E-09
Formate dehydrogenase H	5989.87058	-1.8893878	0.15941172	-11.852252	7.93E-31
Threonine synthase	34558.3541	-1.8903526	0.17668062	-10.699264	2.71E-25
Nitrite transporter NirC	117.522632	-1.9044671	0.2940535	-6.4766009	7.38E-10
PTS system%2C mannose-specific IIC component	13820.4435	-1.9520173	0.14443646	-13.514713	7.87E-40
Oligogalacturonate lyase	250.064775	-1.9537309	0.20212081	-9.6661542	7.85E-21
Starvation lipoprotein Slp	6941.86468	-1.9559104	0.30610839	-6.3896008	1.28E-09
FIG00638501: hypothetical protein	7.87699125	-1.9637192	0.59901082	-3.27827	0.00312741
Glutamate decarboxylase	4773.04581	-1.9639079	0.33393114	-5.8811763	2.65E-08

Hypothetical protein YdjY	133.220993	-1.9686225	0.21950813	-8.9683353	4.91E-18
Arginine deiminase	76.141694	-1.9722576	0.31188071	-6.3237565	1.93E-09
Cyd operon protein YbgT	4703.58337	-1.9771357	0.17079082	-11.576358	1.86E-29
Beta-galactosidase	13545.1318	-1.9986595	0.11401105	-17.530401	1.73E-66
Pyruvate formate-lyase	358753.342	-2.0143361	0.14623426	-13.774721	2.38E-41
Putative polyketide synthase	10968.3207	-2.0155229	0.16666036	-12.093595	5.08E-32
PTS system%2C mannose-specific IIA component	18225.998	-2.0221607	0.14084893	-14.356948	7.79E-45
Transcriptional regulator of catabolic arginine decarboxylase (adiA)	1565.48873	-2.0238997	0.18577996	-10.894069	3.40E-26
Isoaspartyl dipeptidase	4577.08904	-2.0361337	0.11532086	-17.656248	2.27E-67
Lysine decarboxylase%2C inducible	13953.3001	-2.0372624	0.19122989	-10.653472	4.30E-25
Nitrite reductase [NAD(P)H] small subunit	186.4948	-2.0541624	0.27379018	-7.5026882	6.57E-13
Formate hydrogenlyase subunit 3	1240.4963	-2.0651306	0.20509894	-10.068948	1.67E-22
Butyryl-CoA dehydrogenase	23669.6178	-2.0740465	0.21449726	-9.669338	7.64E-21
[NiFe] hydrogenase nickel incorporation-associated protein HypB	764.781344	-2.0760384	0.18115748	-11.459855	6.89E-29
Cobalt/zinc/cadmium efflux RND transporter%2Cmembrane fusion protein%2C CzcB family	1999.91669	-2.0914089	0.32317241	-6.4714957	7.63E-10
Outer membrane porin protein NmpC precursor	7694.05549	-2.0968751	0.19933224	-10.519498	1.70E-24
Chaperone HdeB	11097.0554	-2.0971941	0.23496632	-8.9255096	7.07E-18
hypothetical protein	5922.36954	-2.0987739	0.20181145	-10.399677	5.83E-24
Long-chain-fatty-acid--CoA ligase	79680.9402	-2.1086951	0.20975347	-10.053207	1.93E-22
Glutamate decarboxylase	2481.54768	-2.1097305	0.32754183	-6.4411025	9.26E-10
Flagellar biosynthesis protein FlhB	1399.99261	-2.1161752	0.18879738	-11.208711	1.10E-27
Putative 3-hydroxyacyl-coa dehydrogenase	20195.1593	-2.121575	0.21292459	-9.9639735	4.65E-22
Lysine/cadaverine antiporter membrane protein CadB	4885.15575	-2.1266738	0.20692008	-10.277755	2.03E-23
iron aquisition yersiniabactin synthesis enzyme (Irp2)	28104.8183	-2.1275306	0.17703823	-12.017351	1.24E-31
Formate dehydrogenase H	2047.1166	-2.1351277	0.1681008	-12.701473	2.92E-35
[NiFe] hydrogenase nickel incorporation protein HypA	115.673416	-2.1387564	0.24496285	-8.730942	3.77E-17
[NiFe] hydrogenase metallocenter assembly protein HypC	137.543912	-2.1595467	0.24121408	-8.9528218	5.61E-18
PTS system%2C mannose-specific IID component	17565.2627	-2.1845834	0.15324631	-14.255374	3.12E-44
Cation efflux system protein CusC precursor	567.439953	-2.1872102	0.26006798	-8.410148	5.60E-16
Pyruvate formate-lyase	68667.0007	-2.1917815	0.17451078	-12.559577	1.72E-34
FIG139552: Putative protease	257.708652	-2.2196477	0.22706607	-9.7753388	2.81E-21
LSU ribosomal protein L31p @ LSU ribosomal protein L31p%2C zinc-dependent	1714.63511	-2.2201128	0.1502734	-14.773824	1.93E-47
Chaperone HdeA	10150.8337	-2.2645779	0.19724481	-11.481052	5.51E-29
Formate hydrogenlyase regulatory protein HycA	277.109119	-2.2692674	0.21762194	-10.427567	4.39E-24
Formate hydrogenlyase maturation protein hycH	481.959467	-2.2827702	0.1790553	-12.748967	1.61E-35
Phosphoribosylaminoimidazole-succinocarboxamide synthase	6500.99484	-2.2970927	0.14116536	-16.272354	2.29E-57
Formate hydrogenlyase subunit 2	396.4564	-2.3087067	0.24781379	-9.3162962	2.13E-19
Putative TonB dependent outer membrane receptor	4326.94692	-2.3151381	0.16738862	-13.830917	1.12E-41
Chaperone protein HtpG	32280.7139	-2.3167814	0.30782403	-7.5263176	5.56E-13
Chaperone protein DnaK	123871.375	-2.317084	0.20852298	-11.111888	3.17E-27
Putative fimbrial-like protein	91.4889482	-2.3243547	0.24506505	-9.4846436	4.43E-20
Arginine/agmatine antiporter	857.259125	-2.3267614	0.14432297	-16.121907	2.57E-56
Orf2	1061.7772	-2.3409036	0.2015262	-11.615878	1.18E-29
FIG00638598: hypothetical protein	390.330634	-2.3497831	0.24266414	-9.6832727	6.72E-21
Putative thiosulfate sulfurtransferase ynjE	362.685682	-2.3793692	0.20858908	-11.406969	1.23E-28
Heat shock protein 60 family chaperone GroEL	107336.349	-2.4124887	0.31640716	-7.6246337	2.69E-13
Arginine decarboxylase%2C catabolic	681.932066	-2.425432	0.15407403	-15.741991	9.46E-54
Molybdenum transport system protein ModD	1055.43129	-2.4454184	0.20545944	-11.902195	4.56E-31
Flagellar biosynthesis protein FliP	558.366051	-2.5079391	0.33449368	-7.4977174	6.80E-13
Formate hydrogenlyase subunit 7	991.54509	-2.560522	0.19099325	-13.406348	3.37E-39
3-oxoacyl-[acyl-carrier-protein] synthase	81727.0419	-2.5676042	0.31394004	-8.1786451	3.63E-15
Anthranilate synthase%2C amidotransferase component	37239.0527	-2.5798749	0.42850527	-6.0206374	1.18E-08
ID=gene:EBG00000313296	903.43708	-2.5832904	0.97504497	-2.6494064	0.0194111
ClpB protein	53476.711	-2.589505	0.22008495	-11.765934	2.18E-30
Flagellar biosynthesis protein FliQ	171.287614	-2.5938519	0.29335129	-8.8421358	1.43E-17
Heat shock protein 60 family co-chaperone GroES	6626.40999	-2.6064747	0.38202264	-6.8228278	7.84E-11
Putative inner membrane protein	8136.61086	-2.6143333	0.1988044	-13.150279	9.55E-38
Formate hydrogenlyase subunit 4	655.713412	-2.6151044	0.19806179	-13.203477	4.83E-38
Uptake hydrogenase large subunit	1242.38598	-2.6456137	0.20182938	-13.108169	1.65E-37
NAD-dependent glyceraldehyde-3-phosphate dehydrogenase	193948.443	-2.6923533	0.15873883	-16.960899	2.91E-62
Cation efflux system protein CusF precursor	623.49982	-2.7279622	0.3530247	-7.7273976	1.24E-13
Flagellar protein FlhE	517.64418	-2.7285791	0.18448661	-14.79012	1.57E-47
Outer membrane protein W precursor	2316.3842	-2.7773075	0.19751437	-14.061293	4.64E-43
Putative amidase	7885.36625	-2.8123884	0.20371392	-13.805578	1.57E-41
Formate hydrogenlyase subunit 5	1820.12224	-2.8146035	0.18282901	-15.394731	1.87E-51
Formate hydrogenlyase complex 3 iron-sulfur protein%3B Formate hydrogenlyase subunit 6%3B Ni%2CFe-hydrogenase III medium subunit	563.447067	-2.8193792	0.21155794	-13.326747	9.69E-39
Flagellar biosynthesis protein FliQ	1620.4369	-2.8211608	0.19765066	-14.27347	2.48E-44
Flagellar biosynthesis protein FlhA	4217.51137	-2.8370317	0.18257185	-15.539262	2.07E-52
Flagellar motor switch protein FliM	4014.31537	-2.9005574	0.20337047	-14.262431	2.86E-44
Uptake hydrogenase small subunit precursor	745.255356	-2.920793	0.23586248	-12.383458	1.52E-33
iron aquisition yersiniabactin synthesis enzyme (Irp2)	10795.3113	-2.9368708	0.19827226	-14.812313	1.18E-47
Aldose 1-epimerase family protein YeaD	6154.07077	-2.949278	0.16557042	-17.812831	1.66E-68
iron aquisition yersiniabactin synthesis enzyme (Irp2)	5223.38697	-2.9963933	0.23262019	-12.881054	3.09E-36
Flagellar protein FlgJ [peptidoglycan hydrolase]	7019.86299	-3.0435253	0.20476373	-14.863596	5.60E-48
Putative membrane protein	597.818237	-3.0716277	0.17852316	-17.205765	4.55E-64
FIG00638267: hypothetical protein	343.183998	-3.1449989	0.36337957	-8.6548589	7.14E-17
Flagellar biosynthesis protein FliL	1710.75312	-3.146588	0.19020061	-16.543523	2.81E-59
Flagellar motor switch protein FliN	2724.16025	-3.2216276	0.20544999	-15.680836	2.42E-53
Flagellum-specific ATP synthase FliI	5324.12006	-3.2311566	0.2168875	-14.897846	3.43E-48
FIG00637915: hypothetical protein	906.051366	-3.2514227	0.16676673	-19.496831	4.60E-82
Flagellar P-ring protein FlgI	10441.2703	-3.2642663	0.19394444	-16.830935	2.45E-61
ID=gene:EBG00000313254	76.7034261	-3.2966835	1.23129509	-2.6774114	0.01806533
Flagellar assembly protein FliH	2809.03859	-3.3279537	0.23036064	-14.446711	2.22E-45
Indole-3-glycerol phosphate synthase	32447.8571	-3.3498974	0.33245003	-10.076394	1.55E-22
Flagellar protein FliJ	1030.66515	-3.4204634	0.21911198	-15.610572	7.12E-53
Flagellar M-ring protein FliF	4928.74309	-3.4340898	0.21564995	-15.924371	5.64E-55
FIG00638357: hypothetical protein	343.366424	-3.4424004	0.38906526	-8.847874	1.37E-17
Flagellar L-ring protein FlgH	7739.87682	-3.4950108	0.21321877	-16.391666	3.35E-58
Flagellar motor switch protein FliG	6325.61724	-3.5160714	0.22703111	-15.487179	4.55E-52
Flagellar hook-basal body complex protein FliE	597.932173	-3.5525533	0.36294437	-9.7881482	2.49E-21
Flagellar hook-length control protein FliK	4523.16488	-3.5605286	0.31051868	-11.466391	6.48E-29
Flagellar basal-body P-ring formation protein FlgA	5051.67217	-3.6678276	0.31403439	-11.6797	5.89E-30
Flagellar basal-body rod protein FlgG	24111.2685	-3.7415964	0.21099888	-17.732779	6.15E-68
Lead%2C cadmium%2C zinc and mercury transporting ATPase	6699.95227	-3.788936	0.19567563	-19.363352	5.72E-81
Flagellar hook protein FlgE	65563.9508	-3.9362909	0.31071108	-12.668653	4.39E-35
Flagellar basal-body rod modification protein FlgD	24897.8125	-3.9616281	0.29750919	-13.315986	1.11E-38
Flagellar basal-body rod protein FlgF	21237.3832	-3.9817416	0.22587525	-17.628056	3.38E-67
Flagellar basal-body rod protein FlgC	12613.6773	-4.0730441	0.29931852	-13.607725	2.27E-40
Tryptophan synthase alpha chain	49533.8783	-4.1313597	0.32900015	-12.557318	1.75E-34
Tryptophan synthase beta chain	80187.7869	-4.1440796	0.32189511	-12.874006	3.35E-36
Flagellar basal-body rod protein FlgB	7082.29593	-4.1773211	0.36459985	-11.457276	7.05E-29
Aspartate carbamoyltransferase regulatory chain (PyrI)	3585.53228	-4.3553677	0.22538558	-19.324074	1.14E-80
Aspartate carbamoyltransferase	5442.73045	-4.84097	0.22032484	-21.971967	2.53E-104

Supplemental Table 1: Statistically Differentially Expressed Genes LB

Gene Description	baseMean	log2FoldChar	lfcSE	stat	padj
LSU ribosomal protein L36p	1068.02483	11.261438	0.91037108	12.3701623	9.55E-33
LSU ribosomal protein L31p @ LSU ribosomal protein L31p%2C zinc-independent	2627.12354	9.8664289	0.65568914	15.0474184	1.16E-48
Candidate zinc-binding lipoprotein ZinT	11391.4778	8.8760159	0.21518351	41.2485871	0
FIG00638682: hypothetical protein	9438.70125	6.12794083	0.12948575	47.3252141	0
membrane%3B Transport of small molecules: Cations	14259.3302	5.3308006	0.1170281	45.5514583	0
Putative metal chaperone%2C involved in Zn homeostasis%2C GTPase of COG0523 family	2849.90433	4.99273274	0.14554747	34.3031228	6.39E-255
Zinc ABC transporter%2C periplasmic-binding protein ZnuA	7924.49141	4.89243475	0.11749363	41.6399991	0
FIG01045439: hypothetical protein	3841.69524	3.95666619	0.16973718	23.3105448	1.96E-117
FIG01220641: hypothetical protein	814.061428	3.25473356	0.1791345	18.1692164	4.56E-71
Sulfite reductase [NADPH] flavoprotein alpha-component	2521.42462	2.90899088	0.19965243	14.5702751	1.31E-45
Sulfate adenylyltransferase subunit 2	4135.61485	2.8812242	0.17993749	16.012362	3.96E-55
Cell wall endopeptidase%2C family M23/M37	3074.34986	2.71829861	0.11637982	23.3571304	7.55E-118
Sulfate adenylyltransferase subunit 1	7127.60147	2.71193421	0.17159127	15.8046168	1.01E-53
Zinc ABC transporter%2C ATP-binding protein ZnuC	3693.60668	2.68946747	0.11351162	23.6933236	3.19E-121
Sulfite reductase [NADPH] hemoprotein beta-component	3569.58409	2.64705905	0.24792	10.6770692	2.27E-24
Sulfate and thiosulfate binding protein CysP	3875.75449	2.60464284	0.22651886	11.4985696	2.76E-28
Zinc ABC transporter%2C inner membrane permease protein ZnuB	1364.50995	2.43779296	0.13998662	17.4144712	2.91E-65
Protein ygiW precursor	2077.26306	2.36865842	0.21149292	11.1997054	8.06E-27
Adenylylsulfate kinase	1136.12398	2.32906311	0.19934645	11.6834943	3.51E-29
Sulfate transport system permease protein CysT	1607.44735	2.27802551	0.16612165	13.7129955	2.41E-40
Phosphoadenylyl-sulfate reductase [thioredoxin]	1317.41363	2.23828947	0.22181389	10.0908445	9.48E-22
Carbon starvation induced protein CsiD	1826.97339	2.21907796	0.4559736	4.86668083	2.05E-05
Acetyl-coenzyme A synthetase	2609.42832	2.19869052	0.61996545	3.54647267	0.00286902
Sulfate and thiosulfate import ATP-binding protein CysA	5024.1552	2.18224971	0.18348423	11.8933912	3.06E-30
Succinylornithine transaminase	538.573682	2.12527058	0.6439936	3.30014241	0.0060185
Methylthioribose-1-phosphate isomerase	8421.06708	2.11286367	0.25874052	8.16595593	2.95E-14
Succinylglutamic semialdehyde dehydrogenase	1416.10688	2.10632938	0.72683269	2.89795631	0.0175024
Enoyl-CoA hydratase	680.911748	2.10050748	0.64596624	3.25172952	0.00687019
gamma-aminobutyrate (GABA) permease	2015.04709	2.09077542	0.44466949	4.70186386	4.02E-05
Arginine N-succinyltransferase	718.445569	2.08885977	0.62402086	3.34741977	0.00527571
Cysteine synthase	19829.3464	2.0832434	0.11992132	17.3717523	5.57E-65
5-methylthioribose kinase	11008.7246	2.01166801	0.27381023	7.34694261	1.35E-11
L-fuculose phosphate aldolase	3400.36349	2.01057627	0.2410617	8.34050496	7.29E-15
Sulfate transport system permease protein CysW	2803.72598	2.00888587	0.20019312	10.0347399	1.57E-21
Uronate isomerase	1829.5085	1.98504086	0.23532986	8.4351423	3.49E-15
Isocitrate lyase	2101.78081	1.93145209	0.52570476	3.67402435	0.00189255
3-ketoacyl-CoA thiolase	564.641056	1.89568094	0.64437163	2.94190626	0.01563409
putative integral membrane protein	1125.92174	1.84205924	0.21293042	8.65099162	5.65E-16
Malate synthase	1415.52901	1.81210345	0.51177875	3.54079466	0.00291729
Gamma-aminobutyrate:alpha-ketoglutarate aminotransferase	9180.41073	1.78121994	0.36482196	4.88243622	1.93E-05
Succinylglutamate desuccinylase	687.984093	1.76089504	0.58890796	2.99010228	0.01395129
PTS system%2C fructose-specific IIB component	10.9106817	1.75181394	0.68599809	2.55367177	0.04061309
Taurine-binding periplasmic protein TauA	109.567541	1.73767354	0.28455099	6.1067211	3.69E-08
hypothetical protein	13.598651	1.73473656	0.64191572	2.70243662	0.02863213
L-2-hydroxyglutarate oxidase	3087.94084	1.70384445	0.35727949	4.76894004	3.05E-05
Altronate hydrolase	1832.03247	1.69715433	0.24404142	6.95436997	1.91E-10
Transcriptional repressor of PutA and PutP / Proline dehydrogenase	16781.5205	1.68695679	0.382636	4.40877693	0.00013277
Putative cytochrome oxidase subunit	381.960432	1.67521857	0.23528839	7.11985219	6.45E-11
Succinylarginine dihydrolase	1366.56078	1.67147575	0.54813835	3.0493684	0.01196491
Putative exported protein	5862.7837	1.66179785	0.26364036	6.30327562	1.19E-08
Succinate-semialdehyde dehydrogenase [NADP+]	8613.15485	1.64164037	0.26083286	6.2938402	1.23E-08
PTS system%2C N-acetylgalactosamine-specific IIB component	21.2179272	1.62725949	0.50022146	3.25307813	0.00684671
Starvation sensing protein RspA	3008.29388	1.59247943	0.537787	2.96117129	0.01483311
hypothetical protein	18.4582904	1.58322965	0.55219258	2.86716935	0.01888269
Osmotically inducible protein OsmY	30929.4812	1.57080791	0.25379423	6.18929711	2.32E-08
FIG00641190: hypothetical protein	200.950534	1.53487248	0.32331191	4.74734283	3.33E-05
Respiratory nitrate reductase beta chain	1960.62515	1.52686946	0.22391763	6.81888896	4.84E-10
Periplasmic protein related to spheroblast formation	730.811476	1.51145572	0.206833	7.30761392	1.76E-11
Glycerol-3-phosphate ABC transporter%2C permease protein UgpE (TC 3.A.1.1.3)	211.238016	1.48417496	0.31326441	4.73777074	3.46E-05
FIG00639131: hypothetical protein	654.114322	1.47689533	0.37704878	3.9169874	0.00082628
FIG00638909: hypothetical protein	177.305188	1.46387066	0.30825729	4.74885978	3.32E-05
Butyryl-CoA dehydrogenase	1451.08355	1.44232409	0.45421093	3.17544997	0.00858615
aconitate hydratase	7414.14958	1.43581349	0.44923422	3.19613564	0.00808582
Protein YcgL	524.581106	1.41563241	0.15038235	9.41355416	6.04E-19
FIG00638803: hypothetical protein	29.9259296	1.39542091	0.4972401	2.80633222	0.02217767
Gamma-glutamyltranspeptidase	5772.9777	1.3836001	0.21514852	6.43090693	5.58E-09
D-mannonate oxidoreductase	1434.01775	1.38315403	0.20604108	6.71300127	9.61E-10
Mobile element protein	59.9321731	1.37030281	0.34468124	3.97556543	0.00066738
Protease VII (Omptin) precursor	6350.03433	1.36871194	0.19160368	7.14345319	5.50E-11
Phosphate starvation-inducible protein PhoH%2Cpredicted ATPase	45430.2826	1.33728337	0.53461368	2.50140133	0.0458601
Protein yciF	62.2118739	1.30861682	0.39130178	3.34426492	0.0053058
CsiR%2C transcriptional repressor of CsiD	1222.37568	1.30588891	0.22025959	5.92886304	9.81E-08
Glycerol-3-phosphate ABC transporter%2C ATP-binding protein UgpC (TC 3.A.1.1.3)	1442.9002	1.28628557	0.35884433	3.58452251	0.00254337
Respiratory nitrate reductase alpha chain	4286.44943	1.2855264	0.19901657	6.45939369	4.77E-09
Branched-chain amino acid ABC transporter%2C amino acid-binding protein (TC 3.A.1.4.1)	2484.98441	1.28316339	0.12737035	10.074271	1.09E-21
Succinyl-CoA ligase [ADP-forming] alpha chain	15425.992	1.27801076	0.36027383	3.54733164	0.00286433
Malate dehydrogenase	23879.0419	1.27257876	0.39963171	3.18437883	0.00835737
Isocitrate dehydrogenase [NADP]	92812.9484	1.27207279	0.35250168	3.60869993	0.00235688
Citrate synthase (si)	26927.4065	1.26658213	0.23297992	5.43644328	1.38E-06
Aldehyde dehydrogenase A	1050.26503	1.24616084	0.27293251	4.56582038	6.86E-05
Manganese superoxide dismutase	14089.5909	1.24360044	0.1533485	8.10963536	4.60E-14
Alkyl hydroperoxide reductase protein C	30110.2755	1.23785257	0.12829842	9.64822909	6.67E-20
Glycerol-3-phosphate ABC transporter%2C permease protein UgpA (TC 3.A.1.1.3)	148.812807	1.22439118	0.24422044	5.01346733	1.06E-05
L-cystine uptake protein TcyP	2582.46568	1.22045155	0.17230883	7.08293103	8.00E-11
6-phosphofructokinase class II	3139.2464	1.21601076	0.09020778	13.4801091	5.46E-39
Putative type IV pilin protein precursor	16.3072723	1.21540512	0.49119616	2.47437834	0.04845037
Dihydrolipoamide succinyltransferase component (E2) of 2-oxoglutarate dehydrogenase complex	17381.5397	1.20882189	0.43812355	2.75908907	0.02500505
Two-component system response regulator QseB	120.160935	1.20188353	0.18208164	6.60079488	1.93E-09
Pyruvate oxidase [ubiquinone%2C cytochrome]	21620.9479	1.19698021	0.20537881	5.82815822	1.75E-07
FIG00641652: hypothetical protein	23.6424794	1.1702746	0.4452254	2.62849919	0.03407953
Malate synthase G	4557.12172	1.1702337	0.19523885	5.99385673	6.88E-08
FIG00638451: hypothetical protein	6238.79169	1.15286019	0.23680253	4.86844549	2.04E-05
Nicotinamidase family protein YcaC	4739.52382	1.14257603	0.21320602	5.35902342	2.10E-06
Sialic acid-induced transmembrane protein YjhT(NanM)%2C possible mutarotase	2698.29586	1.13719189	0.16017823	7.09954093	7.37E-11
TonB-dependent receptor%3B Outer membrane receptor for ferrienterochelin and colicins	28528.8668	1.13675754	0.17242475	6.59277486	2.02E-09
Dipeptide-binding ABC transporter%2C periplasmic substrate-binding component (TC 3.A.1.5.2)%3B Putative hemin-binding lipoprotein	6466.97565	1.11709275	0.10165851	10.9886794	7.86E-26
Protein involved in stability of MscS mechanosensitive channel	8004.49544	1.11302326	0.17653462	6.30484402	1.19E-08
Glycerol-3-phosphate ABC transporter%2C periplasmic glycerol-3-phosphate-binding protein (TC 3.A.1.1.3)	1152.07914	1.11142016	0.16367334	6.79047774	5.76E-10
Methylglyoxal reductase%2C acetol producing	8312.70589	1.10855558	0.15161297	7.31174613	1.73E-11
Indole-3-glycerol phosphate synthase	112740.503	1.10777738	0.14734473	7.51826957	3.93E-12
Catalase	4706.69961	1.10334057	0.22880372	4.82221428	2.47E-05
Respiratory nitrate reductase delta chain	812.423721	1.09499256	0.19767795	5.5392751	8.05E-07
ABC transporter%2C periplasmic spermidine putrescine-binding protein PotD (TC 3.A.1.11.1)	1252.73496	1.08401303	0.18665028	5.80772243	1.94E-07
Putative oxidoreductase	1129.13667	1.07771512	0.19479197	5.53264649	8.32E-07
Protein yceI precursor	2659.9623	1.07688035	0.11145495	9.66202308	6.01E-20
Taurine transport ATP-binding protein TauB	180.590691	1.07256834	0.20380912	5.26261201	3.37E-06
FIG001196: Membrane protein YedZ	542.266296	1.07211074	0.22659683	4.73135809	3.55E-05

PTS system%2C N-acetylgalactosamine-specific IIC component	53.1787274	1.07152697	0.34320226	3.1221443	0.00992719
Mannonate dehydratase	622.961526	1.06331919	0.23136617	4.59582834	6.24E-05
FIG00637864: hypothetical protein	1132.49973	1.05557085	0.16570838	6.37005095	8.16E-09
Anthranilate synthase%2C amidotransferase component	144220.739	1.04739877	0.20574346	5.09079988	7.59E-06
Isoaspartyl aminopeptidase	3879.35763	1.04191784	0.11050675	9.42854446	5.39E-19
Acidic protein msyB	2591.27571	1.04061024	0.22595695	4.60534733	5.98E-05
Probable zinc protease pqqL	1133.17772	1.04023419	0.20899904	4.97721985	1.24E-05
periplasmic trehalase precursor	2030.06883	1.03935543	0.19413362	5.35381484	2.15E-06
Probable glutamate/gamma-aminobutyrate antiporter	16816.198	1.03094494	0.25862492	3.98625528	0.00064616
Tryptophan synthase beta chain	244771.743	1.03055464	0.10609901	9.71314082	3.76E-20
Superoxide dismutase [Cu-Zn] precursor	3796.91154	1.02564159	0.17740813	5.78125484	2.26E-07
L-rhamnose mutarotase	33.2914673	1.02449153	0.36179296	2.83170665	0.02072316
D-mannonate oxidoreductase	892.903825	1.01928929	0.24643243	4.13618158	0.00037585
Succinyl-CoA ligase [ADP-forming] beta chain	12855.1784	1.01796288	0.39372622	2.58545873	0.03768626
UPF0337 protein yjbJ	1584.385	1.01642371	0.25874408	3.92829751	0.00079647
Alkanesulfonate utilization operon LysR-family regulator Cbl	299.513062	1.01476424	0.1950606	5.20230251	4.58E-06
Sulfur acceptor protein SufE for iron-sulfur cluster assembly	3755.51058	1.01412251	0.11534467	8.79210513	1.66E-16
Nitrate/nitrite transporter	866.604059	1.01321129	0.17354979	5.83815918	1.67E-07
Putative carboxymethylenebutenolidase	6062.21927	1.01257311	0.13060488	7.75294989	7.02E-13
4-alpha-glucanotransferase (amylomaltase)	3495.85816	1.01176922	0.21692491	4.66414499	4.70E-05
Glycerol uptake facilitator protein	197.653646	1.00780149	0.26450273	3.81017424	0.00119475
Nitric oxide reductase FlRd-NAD(+) reductase	267.156963	1.00386127	0.16056266	6.25214643	1.60E-08
Putative sulfite oxidase subunit YedY	1459.65848	1.00369287	0.24346177	4.1225893	0.00039411
Ribulosamine/erythrulosamine 3-kinase potentially involved in protein deglycation	5092.07168	1.00219822	0.12509619	8.01142081	9.70E-14
HTH-type transcriptional regulator gadW	758.832783	0.99425483	0.15790183	6.29666425	1.22E-08
Isocitrate dehydrogenase phosphatase	392.947655	0.99416872	0.23703996	4.19409766	0.00030222
6-phosphogluconolactonase	8508.0023	0.98259788	0.11772107	8.34683086	7.06E-15
Maltodextrin phosphorylase	4826.49194	0.98153302	0.224304	4.37590517	0.00015188
Cysteine desulfurase	15451.0563	0.98020811	0.11430438	8.57542051	1.07E-15
FIG094199: Fumarylacetoacetate hydrolase	3335.17641	0.97598986	0.13380111	7.29433304	1.89E-11
Glycoprotein-polysaccharide metabolism	6992.41939	0.97288552	0.11762692	8.27094312	1.28E-14
FIG00642236: hypothetical protein	203.881632	0.96632423	0.22335053	4.32649167	0.00018221
FIG00639909: hypothetical protein	22.6323356	0.96299921	0.37348576	2.57840942	0.03830027
Maltose/maltodextrin ABC transporter%2C permease protein MalF	188.434495	0.94946364	0.27299551	3.47794603	0.00350868
Lactate-responsive regulator LldR in Enterobacteria%2C GntR family	413.623487	0.94042727	0.26961409	3.48804943	0.00342024
FIG01046459: hypothetical protein	1286.44244	0.93972574	0.13948938	6.73689815	8.24E-10
Stage V sporulation protein involved in spore cortex synthesis (SpoVR)	29501.5172	0.92008662	0.18256403	5.03980222	9.47E-06
Ribosome hibernation protein YfiA	23856.7256	0.91996125	0.22345838	4.11692443	0.00040249
Glucose dehydrogenase%2C PQQ-dependent	23701.1204	0.91703701	0.24151541	3.79701247	0.00125759
Taurine transport system permease protein TauC	149.323254	0.91482717	0.19017914	4.81034456	2.57E-05
Iron-sulfur cluster assembly protein SufD	17762.6168	0.91012554	0.13363719	6.81042128	5.07E-10
Cytoplasmic protein YaiB	331.092347	0.90989047	0.20100459	4.52671495	7.94E-05
Flavohemoprotein (Hemoglobin-like protein) (Flavohemoglobin) (Nitric oxide dioxygenase)	867.788351	0.90882046	0.17771693	5.11386539	6.85E-06
Alcohol dehydrogenase	2929.18273	0.90533913	0.14055883	6.44099798	5.29E-09
Phage minor tail protein	41.2541925	0.90030071	0.33274213	2.70570099	0.02840434
Dipeptide-binding ABC transporter%2C periplasmic substrate-binding component (TC 3.A.1.5.2)	29784.0733	0.89734082	0.13489295	6.65224391	1.41E-09
D-allose ABC transporter%2C substrate-binding component	107.100611	0.89456995	0.25792337	3.4683556	0.00361928
Galactose/methyl galactoside ABC transport system%2C D-galactose-binding periplasmic protein MglB (TC 3.A.1.2.3)	203.122499	0.89278031	0.23265451	3.83736523	0.00109274
Respiratory nitrate reductase gamma chain	633.50164	0.8914331	0.16073095	5.54611987	7.79E-07
Glycerophosphoryl diester phosphodiesterase	904.644134	0.88856732	0.15211653	5.84135941	1.65E-07
Altronate oxidoreductase	297.466462	0.8882198	0.23732094	3.74269452	0.00151442
Thiol peroxidase%2C Tpx-type	22280.2369	0.88761586	0.11766703	7.54345435	3.35E-12
Phosphomethylpyrimidine kinase	5992.71818	0.88387732	0.11199159	7.89235434	2.40E-13
Phosphoenolpyruvate synthase	16287.8479	0.88260096	0.21351769	4.13361993	0.00037917
Alcohol dehydrogenase	43202.9678	0.8816172	0.2376358	3.70995108	0.00168742
Putrescine aminotransferase	21521.2335	0.87829489	0.22196851	3.95684458	0.00071738
Sulfate-binding protein Sbp	233.858749	0.87591993	0.16872793	5.19131556	4.78E-06
Propionate catabolism operon regulatory protein PrpR	106.808994	0.87552145	0.28671112	3.05367111	0.01182186
HTH-type transcriptional regulator gadX	1848.25765	0.87528026	0.17997906	4.86323378	2.08E-05
Glutothione ABC transporter ATP-binding protein	4767.6915	0.87452737	0.10855697	8.05592998	6.88E-14
Transketolase	43701.3055	0.87170514	0.20544042	4.24310439	0.00025051
NAD-dependent glyceraldehyde-3-phosphate dehydrogenase	10115.9129	0.86997551	0.21189955	4.1056033	0.00041839
Glycolate permease	182.71614	0.86919041	0.22217253	3.91223164	0.00084028
3-isopropylmalate dehydratase small subunit	1713.98864	0.86836383	0.11536298	7.52723126	3.73E-12
Putrescine transport system permease protein PotH (TC 3.A.1.11.2)	2965.87386	0.8657165	0.14381057	6.01983929	5.95E-08
Oxidoreductase%2C aldo/keto reductase family	5265.06673	0.86427689	0.07766419	11.1283834	1.72E-26
N-acetylneuraminic acid outer membrane channel protein NanC	93.643357	0.86028042	0.22028532	3.90530065	0.00085508
Putative regulator	191.715444	0.85409394	0.16539465	5.16397554	5.40E-06
Putrescine transport ATP-binding protein PotA (TC 3.A.1.11.1)	444.953844	0.84850123	0.19550597	4.34002717	0.00017317
Iron-sulfur cluster assembly protein SufB	13273.7983	0.84794535	0.15443639	5.49058002	1.04E-06
cysteine synthase B	1139.40689	0.84475631	0.16561736	5.10065063	7.27E-06
Hyphotheical protein	2484.27852	0.84048277	0.18959737	4.43298755	0.00011903
Thiamin-phosphate pyrophosphorylase	18843.3323	0.84046754	0.17465871	4.81205623	2.56E-05
3-keto-L-gulonate 6-phosphate decarboxylase homolog	77.5812469	0.83769285	0.29168218	2.87193703	0.01866508
FIG00638524: hypothetical protein	1177.88358	0.8337447	0.17763587	4.6935604	4.17E-05
Gluconokinase	182.438409	0.83364681	0.22848555	3.64857568	0.00205784
Fumarate hydratase class II	1455.43511	0.83305044	0.19159584	4.34795674	0.0001693
FIG00638143: hypothetical protein	43.3607078	0.82659785	0.30655234	2.69643301	0.02896738
Maltose/maltodextrin ABC transporter%2C substrate binding periplasmic protein MalE	303.28639	0.82443184	0.29922494	2.75522431	0.02525116
Pyruvate-flavodoxin oxidoreductase	4776.32528	0.82376569	0.16536796	4.98141059	1.24E-05
Fructose-bisphosphate aldolase class I	19385.5004	0.82272208	0.18143151	4.53461506	7.72E-05
Serine protein kinase (prkA protein)%2C P-loop containing	94077.1439	0.81941738	0.20012329	4.09456281	0.00043684
Hydroxyethylthiazole kinase	7682.90013	0.81842538	0.11531581	7.09725204	7.40E-11
Acetolactate synthase small subunit	1122.4081	0.81784788	0.16151392	5.06363715	8.55E-06
Catalase	11209.0546	0.81248564	0.13410001	6.05880383	4.75E-08
Iron binding protein SufA for iron-sulfur cluster assembly	3615.66715	0.80848831	0.12975768	6.23075507	1.81E-08
Putrescine transport ATP-binding protein PotG (TC 3.A.1.11.2)	5837.77425	0.80656064	0.14077471	5.72944261	2.97E-07
Acetate permease ActP (cation/acetate symporter)	431.002767	0.80620407	0.21938043	3.67491344	0.00188927
PhnB protein%3B putative DNA binding 3-demethylubiquinone-9 3-methyltransferase domain protein	569.178925	0.80545335	0.13864461	5.8094819	1.93E-07
4-aminobutyraldehyde dehydrogenase	1389.39545	0.80243366	0.19392263	4.13790628	0.00037391
FIG005189: putative transferase clustered with tellurite resistance proteins TehA/TehB	1291.59402	0.80133008	0.17665513	4.53612695	7.69E-05
L-sorbose 1-phosphate reductase	124.796687	0.80084942	0.18964889	4.22280038	0.00026946
Lysine-arginine-ornithine-binding periplasmic protein precursor (TC 3.A.1.3.1)	129.245027	0.80047638	0.26776129	2.98951495	0.01395257
Phage tail assembly protein I	227.750098	0.79997323	0.16327103	4.89966441	1.78E-05
Probable secreted protein	14007.0446	0.79710528	0.23091646	3.45192054	0.00382375
D-mannonate oxidoreductase	1466.86946	0.79496359	0.24993407	3.1806932	0.00845367
Phosphoenolpyruvate-dihydroxyacetone phosphotransferase	1211.54945	0.7943445	0.14615542	5.43493019	1.39E-06
FIG00895798: hypothetical protein	10521.2699	0.79290542	0.15163409	5.22907082	4.02E-06
Stationary phase inducible protein CsiE	2151.05488	0.79270199	0.18823518	4.21123187	0.00028087
Glycosyl hydrolase YegX%2C family 25	518.341071	0.79254802	0.14233869	5.56804365	7.04E-07
FIG00639204: hypothetical protein	236.91727	0.79250499	0.23246856	3.40908466	0.00437033
Outer membrane porin protein NmpC precursor	5289.27893	0.78878965	0.26434625	2.98392605	0.01411669
Iron-sulfur cluster assembly ATPase protein SufC	6659.93927	0.7844367	0.11962331	6.55755701	2.53E-09
DNA-binding protein HU-alpha	13622.339	0.78215984	0.12869575	6.07758865	4.39E-08
Acetate kinase	147.084476	0.7820686	0.23239191	3.36530043	0.00501695
Transcriptional regulator CsgD for 2nd curli operon	467.244	0.78144677	0.15406263	5.07226673	8.21E-06
Curli production assembly/transport component CsgE	142.429019	0.77968201	0.22172971	3.51636235	0.00316457
Phosphate starvation-inducible protein PsiF	1147.87985	0.77777964	0.16385079	4.74687756	3.33E-05
L-xylulose 5-phosphate 3-epimerase	146.684965	0.77539107	0.22503883	3.44558785	0.0038909

Tagatose 1%2C6-bisphosphate aldolase	63.2406859	0.77398074	0.27464489	2.81811452	0.02147275
Cardiolipin synthetase	2168.00634	0.7735766	0.18036289	4.28900095	0.00021082
Putative single stranded DNA-binding protein of prophage	169.795166	0.77256091	0.2323359	3.32518958	0.00560304
biofilm regulator BssR	17910.2682	0.7706759	0.22608613	3.4087713	0.00437033
Molybdenum cofactor biosynthesis protein MoaB	1994.98435	0.7695463	0.12771421	6.02553407	5.79E-08
Biofilm PGA synthesis auxiliary protein PgaD	143.506329	0.76739605	0.19221577	3.99236778	0.00063241
UPF0098 protein ybhB	2108.80675	0.76543799	0.12510222	6.11850025	3.48E-08
Sialic acid-induced transmembrane protein YjhT(NanM)%2C possible mutarotase	445.185264	0.76515651	0.12628489	6.05897138	4.75E-08
Xylanase	97.2514816	0.76462591	0.22983936	3.32678397	0.00557888
Thiamin biosynthesis protein ThiC	40529.5064	0.76411076	0.14417558	5.29986252	2.79E-06
Alcohol dehydrogenase	2508.45095	0.7633305	0.16197869	4.71253644	3.84E-05
Phosphonates transport ATP-binding protein PhnL	50.3196858	0.76215965	0.30240699	2.520311	0.04397162
Glutamate decarboxylase	1708.17142	0.76135837	0.24519186	3.10515345	0.01032641
Putrescine ABC transporter putrescine-binding protein PotF (TC 3.A.1.11.2)	13422.0189	0.76068958	0.12801303	5.9422826	9.19E-08
Inner membrane protein YhaH	2573.81677	0.75501445	0.16496993	4.57667917	6.65E-05
Tryptophan synthase alpha chain	165388.489	0.7530484	0.10166899	7.40686386	8.88E-12
Dethiobiotin synthetase	1213.68314	0.7510686	0.16264588	4.61781501	5.69E-05
Acetyl-CoA:acetoacetyl-CoA transferase%2C alpha subunit	139.036908	0.7495795	0.21046191	3.56159224	0.00273982
Glutamate Aspartate periplasmic binding protein precursor GltI (TC 3.A.1.3.4)	6369.26903	0.74888732	0.12872579	5.81769468	1.85E-07
Molybdenum cofactor biosynthesis protein MoaC	2129.33079	0.74858086	0.13251657	5.64896027	4.60E-07
Allantoinase	344.1296	0.74702585	0.17199957	4.34318448	0.00017162
Glutamate decarboxylase	7149.63316	0.74519393	0.25299503	2.9454884	0.01549827
Heat shock protein 60 family chaperone GroEL	22657.5515	0.73543568	0.17201156	4.27550139	0.00022236
Alpha%2Calpha-trehalose-phosphate synthase [UDP-forming]	9085.1909	0.73446713	0.16812577	4.36855763	0.00015622
Osmoprotectant ABC transporter permease protein YehY	959.828471	0.73253809	0.17378246	4.21525917	0.00027725
Proofreading thioesterase in enterobactin biosynthesis EntH	2942.17046	0.73102098	0.10479243	6.9758947	1.68E-10
Acetolactate synthase large subunit	806.927712	0.73023818	0.14626124	4.99269784	1.17E-05
Enoyl-CoA hydratase	1099.52339	0.72975733	0.22391238	3.25912002	0.00674717
Uncharacterized membrane protein YqjD	7399.80432	0.72796945	0.15943744	4.56586259	6.86E-05
Melibiose operon regulatory protein	716.81908	0.7268617	0.19587093	3.71092174	0.00168702
Ethanolamine utilization polyhedral-body-like protein EutM	77.6982565	0.72358229	0.2845779	2.54265102	0.04167153
type 1 fimbriae regulatory protein FimB	308.281979	0.71745842	0.19828375	3.61834205	0.00228625
Glucarate dehydratase	1791.34538	0.71617579	0.11593835	6.17721222	2.44E-08
NADP-dependent malic enzyme	13429.5972	0.71564969	0.13698675	5.22422575	4.11E-06
Oligopeptide transport system permease protein OppB (TC 3.A.1.5.1)	1807.41183	0.71547504	0.11029017	6.48720611	4.00E-09
Hyphotheical protein	392.749599	0.71134105	0.13687877	5.19686906	4.66E-06
Nicotinamidase/isochorismatase family protein	447.774196	0.70944478	0.14395065	4.92838896	1.57E-05
ATP-dependent Clp protease ATP-binding subunit ClpA	63453.5856	0.70940447	0.12986444	5.46265369	1.22E-06
FIG00638953: hypothetical protein	2785.04607	0.7080274	0.18660656	3.79422559	0.00126939
Thiazole biosynthesis protein ThiG	25737.6669	0.70715376	0.13982995	5.05724097	8.77E-06
ABC transporter protein IroC	224808.545	0.70429716	0.1481884	4.75271467	3.27E-05
Flavoprotein wrbA	19382.2963	0.70190911	0.14471329	4.850343	2.19E-05
Isochorismatase	15758.9493	0.70034133	0.1524098	4.59512002	6.25E-05
FIG00640525: hypothetical protein	653.064105	0.69818502	0.14026953	4.97745333	1.24E-05
N-acetylgalactosamine 6-sulfate sulfatase (GALNS)	96.2362981	0.69704914	0.20740724	3.36077538	0.00507051
RNA signal recognition particle 4.5S RNA	301.402922	0.69664674	0.21324449	3.26689213	0.00660865
Putative cytoplasmic protein	578.624513	0.69493473	0.18220743	3.81397588	0.00117874
UPF0229 protein YeaH	23052.1395	0.69426839	0.18928642	3.66781939	0.00193253
Phosphoenolpyruvate-dihydroxyacetone phosphotransferase	738.261587	0.69317993	0.14839212	4.67127193	4.55E-05
D-3-phosphoglycerate dehydrogenase	64.5163607	0.69153158	0.25461645	2.7159737	0.02763948
2-dehydro-3-deoxyglucarate aldolase	73.7924665	0.69025436	0.24603361	2.80552876	0.02217767
DNA-directed RNA polymerase beta' subunit	99333.7365	0.68776304	0.13020786	5.28203946	3.06E-06
Aldehyde dehydrogenase B	1861.59373	0.68592773	0.20261538	3.38536851	0.00470495
PTS system%2C N-acetylglucosamine-specific IIA component	6930.97073	0.68384406	0.15418447	4.43523323	0.00011813
Inner membrane protein	4010.77767	0.68219085	0.15770686	4.32568914	0.00018239
L-rhamnose isomerase	63.825031	0.68075003	0.2505717	2.71678738	0.02759709
Cytochrome B561	190.807298	0.67573954	0.15497499	4.36031353	0.00016089
2%2C3-dihydro-2%2C3-dihydroxybenzoate dehydrogenase	6728.03338	0.67570773	0.12044712	5.60999493	5.63E-07
Ethanolamine ammonia-lyase heavy chain	659.167051	0.67568615	0.14317702	4.71923598	3.74E-05
Thiazole biosynthesis protein ThiH	29297.1094	0.6753436	0.13252721	5.0958863	7.42E-06
FIG00639826: hypothetical protein	645.771609	0.67373247	0.14815686	4.54742669	7.39E-05
2-ketoaldonate reductase%2C broad specificity	2316.72855	0.67332261	0.11932832	5.64260548	4.75E-07
Osmoprotectant ABC transporter binding protein YehZ	1472.57684	0.67000152	0.13826474	4.84578733	2.22E-05
Lipoprotein Bor	68.7747028	0.66969001	0.27147722	2.46683687	0.04917958
putative protein PaaI%2C possibly involved in aromatic compounds catabolism	364.296426	0.66889405	0.18337997	3.6475851	0.00206224
Lipid A biosynthesis (KDO) 2-(lauroyl)-lipid IVA acyltransferase	893.891499	0.66766685	0.11708454	5.70243373	3.41E-07
AMP nucleosidase	8399.41559	0.66657847	0.10537327	6.32587826	1.06E-08
NAD synthetase	5248.38121	0.66258306	0.12856569	5.15365366	5.65E-06
Maltose operon periplasmic protein MalM	228.646071	0.66207359	0.20036905	3.30427082	0.0059551
NADH-ubiquinone oxidoreductase chain H	3138.79657	0.66154971	0.20116624	3.28857225	0.00622016
Outer membrane protein A precursor	358252.837	0.66122482	0.15938441	4.14861673	0.00036109
L-threonine 3-dehydrogenase	9263.85865	0.65976845	0.13692178	4.8185794	2.48E-05
Phage portal protein	157.862556	0.65906803	0.17087624	3.85699052	0.00102454
Putative Dihydrolipoamide dehydrogenase	1769.22881	0.65851698	0.15085315	4.36528491	0.00015791
Uncharacterized protein ygiV	249.951186	0.65815755	0.14333092	4.59187416	6.30E-05
Dipeptide transport ATP-binding protein DppF (TC 3.A.1.5.2)	1782.38613	0.65754576	0.14316144	4.5930368	6.29E-05
Arabinose-proton symporter	2314.72714	0.6564945	0.16157872	4.06300105	0.00048704
hypothetical protein	113.189353	0.65563708	0.22836734	2.87097569	0.01869275
Inner membrane protein YqjK	10776.3329	0.65452709	0.14974017	4.37108568	0.00015485
FIG00643425: hypothetical protein	243.262943	0.65382542	0.18680428	3.50005589	0.00332656
Threonine synthase	5553.35347	0.65047009	0.1136244	5.72473942	3.03E-07
Putative tail component of prophage CP-933K	94.6777234	0.64948543	0.2160735	3.00585416	0.01337692
Chaperone protein hchA	29340.1857	0.64933857	0.21226306	3.05912184	0.0116612
Putative cytoplasmic protein USSDB7A	64.1267368	0.64890447	0.24977929	2.59791141	0.03659691
Aconitate hydratase 2	33447.648	0.64427927	0.21205508	3.03826382	0.01227355
Anaerobic nitric oxide reductase flavorubredoxin	225.777288	0.64278697	0.18705871	3.43628454	0.00400085
Co-activator of prophage gene expression IbrA	69.7849616	0.64111356	0.24080633	2.66236173	0.03127228
Acetolactate synthase small subunit	172.312762	0.63811078	0.2054753	3.1055352	0.01032641
7-alpha-hydroxysteroid dehydrogenase	8659.94849	0.63681536	0.19650659	3.240682	0.00707661
COG1457: Purine-cytosine permease and related proteins	169.742904	0.63655669	0.17534128	3.63038695	0.0021971
Ethanolamine utilization polyhedral-body-like protein EutK	471.876755	0.63643117	0.18352517	3.46781405	0.00362106
iron aquisition yersiniabactin synthesis enzyme (Irp1%2Cpolyketide synthetase)	347173.515	0.63614699	0.15636328	4.06839124	0.00047697
HTH-type transcriptional regulator mlrA	1692.51274	0.63588096	0.15021133	4.2332424	0.00025915
FIG00639943: hypothetical protein	2188.17643	0.63526096	0.15320197	4.14655857	0.00036348
Glutaminase	2794.09487	0.63242851	0.17939333	3.52537366	0.0030727
iron aquisition yersiniabactin synthesis enzyme (Irp1%2Cpolyketide synthetase)	414634.199	0.6272315	0.15776529	3.9757256	0.00066738
Spermidine Putrescine ABC transporter permease component potC (TC_3.A.1.11.1)	219.45292	0.62441045	0.20757258	3.00815476	0.01331523
Sulfur carrier protein ThiS	4264.28729	0.623962	0.15107647	4.13010718	0.00038322
Putative cytoplasmic protein	148.277535	0.62317212	0.22353557	2.7877985	0.02322473
Protein containing PTS-regulatory domain	87.6772724	0.62215136	0.24775719	2.5111334	0.04479808
Transcription regulator [contains diacylglycerol kinase catalytic domain]	1278.81654	0.62142081	0.12127263	5.12416376	6.58E-06
Putative RTX family exoprotein A gene	2954.49915	0.62030682	0.10347588	5.9946997	6.88E-08
6-phospho-beta-glucosidase	205.867855	0.61859139	0.17217345	3.59283849	0.00248002
Putrescine transport system permease protein PotI (TC 3.A.1.11.2)	2610.03372	0.61803672	0.15414052	4.00956688	0.00059463
Antigen 43 precursor	3497.4491	0.61482404	0.16272078	3.77839907	0.00134267
UPF0410 protein YeaQ	2122.1015	0.61310601	0.19055385	3.21749472	0.00757514
Molybdenum cofactor biosynthesis protein MoaE	961.521718	0.61285698	0.12714928	4.81998011	2.48E-05
Putative membrane protein YchH	3031.91768	0.61279797	0.20806229	2.94526209	0.01549827
Putative NAGC-like transcriptional regulator	246.916671	0.61269518	0.15341082	3.99381992	0.00062989

Hypothetical protein GlcG in glycolate utilization operon	300.220031	0.60896335	0.21008993	2.89858425	0.0175024
Transaldolase	16188.035	0.60885936	0.18274894	3.33167099	0.0055205
Putative transport protein	274.40707	0.60858366	0.19564052	3.11072408	0.01021953
Di/tripeptide permease DtpB	750.782101	0.60605876	0.13884496	4.36500368	0.00015791
Glutamate synthase [NADPH] small chain	10671.5995	0.60407302	0.09473595	6.37638609	7.90E-09
Glutamate Aspartate transport system permease protein GltK (TC 3.A.1.3.4)	296.457286	0.60400713	0.18289565	3.30246848	0.00597701
Inner membrane protein YbhQ	760.395416	0.60285895	0.17146812	3.51586613	0.00316457
FIG00639237: hypothetical protein	12076.5991	0.59999232	0.21841077	2.74708206	0.02571894
Ethanolamine ammonia-lyase light chain	687.552765	0.59939453	0.16777466	3.57261655	0.0026487
Large-conductance mechanosensitive channel	18860.7233	0.59650531	0.14017812	4.25533817	0.00023899
FIG00638157: hypothetical protein	2488.76325	0.59622763	0.18152201	3.28460245	0.00627427
Quinone oxidoreductase	5215.81895	0.5942376	0.12680226	4.68633284	4.30E-05
Phage EaA protein	320.208351	0.59421561	0.14637225	4.05961914	0.00049305
Gifsy-2 prophage protein	219.684201	0.59378598	0.18501579	3.20938005	0.00777216
Succinyl-CoA ligase [ADP-forming] beta chain	231.273245	0.59327822	0.16446992	3.60721422	0.00236641
probable lipoprotein	647.274044	0.5931323	0.19924495	2.97690007	0.01430341
Glycolate utilization operon transcriptional activator GlcC	396.30146	0.59295356	0.23510212	2.5221106	0.04385612
FIG00639173: hypothetical protein	687.70674	0.5926552	0.1292724	4.58454534	6.43E-05
D-serine dehydratase	634.910152	0.5921593	0.13478308	4.39342479	0.00014192
4-hydroxybenzoyl-CoA thioesterase family active site	122.567039	0.59084727	0.22639349	2.60982446	0.03565357
Sulfur carrier protein adenylyltransferase ThiF	22582.4473	0.59069261	0.15749666	3.75050876	0.00147901
Ribosyl nicotinamide transporter%2C PnuC-like	10027.7743	0.58955126	0.08911163	6.61587339	1.76E-09
FIG00638941: hypothetical protein	12651.6724	0.58926649	0.17611203	3.34597516	0.00528819
Putative exported protein	8139.43614	0.58709746	0.17733818	3.31060949	0.00587043
HtrA suppressor protein	177.249832	0.58491223	0.16005267	3.65449843	0.00202136
NAD(P)H-flavin oxidoreductase	227.175087	0.58203606	0.19558902	2.97581155	0.01433874
Anthranilate synthase%2C aminase component	73337.3706	0.58024503	0.21675218	2.67699749	0.03018002
Antigen 43 precursor	9354.44527	0.57972251	0.16577927	3.49695415	0.00334737
hypothetical protein	214.153508	0.57609774	0.18211015	3.16345756	0.00889161
predicted 4-deoxy-L-threo-5-hexosulose-uronate ketol-isomerase	254.379844	0.57429761	0.19751251	2.90765185	0.01716291
S-ribosylhomocysteine lyase	10131.6789	0.57261467	0.16464311	3.47791462	0.00350868
Naphthoate synthase	3365.35717	0.57231665	0.18334748	3.12148628	0.00993728
Inner membrane protein YqjE	12752.8302	0.57137315	0.16321538	3.50073101	0.00332339
ATP-dependent RNA helicase	2536.19429	0.57132519	0.13986287	4.08489533	0.00045132
Trehalose-6-phosphate phosphatase	3602.18379	0.57126995	0.14282768	3.99971457	0.00061572
YcgN (Fragment)	721.160707	0.57034882	0.11932115	4.77994749	2.92E-05
orf%2C hypothetical protein	708.387261	0.57011004	0.13464883	4.23405109	0.00025887
Cell filamentation protein fic	7838.70703	0.56959408	0.1921441	2.96441107	0.01472156
Ethanolamine utilization polyhedral-body-like protein EutL	541.342713	0.56942974	0.1755914	3.24292503	0.00703956
Homoserine kinase	5656.96738	0.56832863	0.15524276	3.66090258	0.00198043
Cystine ABC transporter%2C periplasmic cystine-binding protein FliY	6685.78465	0.56387305	0.14442774	3.90418802	0.00085731
Dipeptidyl carboxypeptidase Dcp	2760.69665	0.56260255	0.14688583	3.830203	0.00111624
Chaperone protein HtpG	7315.13612	0.56241873	0.13282811	4.23418444	0.00025887
Tripeptide aminopeptidase	8457.6895	0.56221464	0.151402	3.71338979	0.00167366
Regulator of sigma D	1779.60221	0.56151506	0.14831224	3.78603329	0.00130948
Prophage CP4-57 integrase	204.23102	0.5611827	0.22261321	2.52088675	0.04393608
FIG00639422: hypothetical protein	1263.75773	0.56112109	0.15212565	3.68853707	0.00180677
Putative uncharacterized protein YrbL	2256.68392	0.55830914	0.10366351	5.38578271	1.82E-06
ABC-type polar amino acid transport system%2CATPase component	628.067099	0.55782471	0.12524166	4.45398677	0.00010888
Protein YciE	417.744068	0.5574773	0.15635979	3.56534952	0.00271421
PTS system%2C mannitol-specific IIC component	2575.23776	0.5568576	0.14445972	3.85476031	0.00103189
Non-specific DNA-binding protein Dps / Iron-binding ferritin-like antioxidant protein / Ferroxidase	37142.8478	0.5565227	0.18829234	2.95563108	0.0150665
NAD(P) transhydrogenase subunit beta	12855.7364	0.55648694	0.14325416	3.88461265	0.00092203
Inner membrane transport protein YbaT	2483.97479	0.55194977	0.19717619	2.79927197	0.02254586
Cation transport regulator chaB	846.840225	0.55167145	0.15525093	3.55341794	0.00280352
Phosphate transport system regulatory protein PhoU	1175.25509	0.55008132	0.11189836	4.91590172	1.66E-05
Osmoprotectant ABC transporter ATP-binding subunit YehX	1406.5105	0.5499187	0.19969048	2.75385533	0.02528066
Trans-aconitate 2-methyltransferase	1186.27828	0.54889601	0.18358465	2.98987967	0.01395129
type 1 fimbriae major subunit FimA	121.7245	0.54825603	0.17947406	3.05479262	0.01179134
Fosmidomycin resistance protein	463.156982	0.54568308	0.15089056	3.61641635	0.00229551
2-deoxy-D-gluconate 3-dehydrogenase	262.690787	0.54498987	0.16477672	3.30744462	0.00590432
S-(hydroxymethyl)glutathione dehydrogenase	1630.79966	0.54322416	0.16654355	3.26175446	0.0066937
Glycolate dehydrogenase	245.796655	0.54196129	0.16201215	3.34518927	0.00529566
PapI protein	253.616733	0.54062303	0.2067189	2.61525694	0.03515244
hypothetical protein	138.275742	0.53968786	0.18875497	2.85919807	0.01927579
L-lysine 6-monooxygenase [NADPH]	121955.144	0.53555433	0.11717086	4.57071242	6.76E-05
L%2CD-transpeptidase YcbB	18558.3943	0.5347674	0.13137361	4.07058466	0.00047355
3-isopropylmalate dehydratase large subunit	2406.11822	0.53346057	0.17060761	3.12682757	0.0097943
L%2CD-transpeptidase YnhG	12598.2585	0.52734978	0.11494918	4.5876775	6.37E-05
Endonuclease/Exonuclease/phosphatase family protein	1358.29077	0.52641909	0.19934687	2.64071908	0.03301929
2-hydroxy-3-oxopropionate reductase	502.952249	0.5263154	0.17130091	3.07246125	0.01123191
Branched-chain amino acid aminotransferase	7318.96531	0.52630976	0.11035257	4.76934752	3.05E-05
hypothetical protein	168.560061	0.525841	0.18870347	2.78659957	0.02328834
Putative Rz endopeptidase from lambdoid prophage DLP12	141.742949	0.52491989	0.16819464	3.1209074	0.00994472
Acyl-CoA dehydrogenases	3841.39911	0.52397982	0.17860269	2.93377337	0.01597679
Peptide methionine sulfoxide reductase MsrA	5996.22678	0.52297666	0.10060079	5.1985341	4.65E-06
Transcriptional regulator%2C AraC family	347.513809	0.52283562	0.13672075	3.82411319	0.00113561
Putative membrane protein	3408.12988	0.51792071	0.19509436	2.65471904	0.03193317
Ribosome-associated heat shock protein implicated in the recycling of the 50S subunit (S4 paralog)	459.372934	0.51563007	0.12595424	4.09378878	0.00043731
iron aquisition outermembrane yersiniabactin receptor (FyuA%2CPsn%2Cpesticin receptor)	69662.3671	0.51416579	0.16220933	3.16976707	0.00873374
NADH-ubiquinone oxidoreductase chain J	2281.65771	0.51408834	0.1814566	2.83312007	0.02066965
PTS system%2C maltose and glucose-specific IIC component	412.498214	0.51326383	0.17668303	2.90499797	0.0172553
Outer membrane protein C precursor	163663.101	0.51233789	0.1247247	4.10775005	0.00041548
Hypothetical zinc-type alcohol dehydrogenase-like protein YphC	301.000799	0.51137062	0.18094988	2.82603455	0.02105214
Head decoration protein	137.288297	0.51130425	0.20225149	2.52806173	0.04322722
Aquaporin Z	2526.09578	0.50999588	0.10704958	4.764109	3.12E-05
Glutathione-regulated potassium-efflux system protein KefC	1700.11278	0.50781774	0.16787359	3.02500071	0.01277376
Alpha-ketoglutarate-dependent taurine dioxygenase	264.189487	0.50680938	0.1800294	2.81514786	0.02162957
Cytoplasmic trehalase	9945.75286	0.5047901	0.16215035	3.11309906	0.01016219
FIG00638099: hypothetical protein	259.482296	0.5038423	0.1706076	2.95322307	0.01516838
Transcriptional regulator%2C TetR family	3244.82895	0.50335599	0.11009206	4.57213688	6.75E-05
Flagellar biosynthesis protein FliC	311257.262	0.50288088	0.14417023	3.48810475	0.00342024
Beta-1%2C3-galactosyltransferase / Beta-1%2C4-galactosyltransferase	334.072406	0.50180484	0.1667077	3.01008787	0.01324557
Uncharacterized protein ImpA	1664.11658	0.50032773	0.1430467	3.49765312	0.00334611
Aspartokinase	11558.0932	0.49794989	0.16529881	3.01242274	0.01320318
probable membrane protein b2001	342.020738	0.49785997	0.17475385	2.84892129	0.019771
FIG00638107: hypothetical protein	2979.64155	0.49640386	0.15984687	3.10549638	0.01032641
Putative cytoplasmic protein %2Cprobably associated with Glutathione-regulated potassium-efflux	299.174013	0.49481508	0.19456114	2.54323694	0.0416365
Capsular polysaccharide export system protein KpsF	4564.32415	0.49461033	0.15321556	3.22819901	0.00735431
Putative GTP-binding protein YdgA	21788.8197	0.49326705	0.10813924	4.5614067	6.96E-05
NADH-ubiquinone oxidoreductase chain G	12733.4583	0.49305318	0.19179846	2.57068375	0.03906552
ABC transporter%2C periplasmic substrate-binding protein YnjB	868.103724	0.49002637	0.12771581	3.83684985	0.00109289
FIG00638146: hypothetical protein	155.013998	0.48982311	0.19733178	2.48223122	0.04770039
Xanthine/uracil/thiamine/ascorbate permease family protein	306.881131	0.48961788	0.15047042	3.25391447	0.00684469
Thiosulfate sulfurtransferase%2C rhodanese	3638.44582	0.48868937	0.10368029	4.71342809	3.83E-05
Membrane alanine aminopeptidase N	23618.152	0.48796502	0.10415142	4.68514989	4.31E-05
Glycosyltransferase IroB	246440.751	0.48471768	0.12636326	3.83590673	0.00109289
Enterobactin synthetase component F%2C serine activating enzyme	130373.027	0.48303419	0.1262646	3.8255711	0.00113179
Periplasmic protein YqjC	6552.71935	0.48279155	0.12620639	3.82541288	0.00113179

S-formylglutathione hydrolase	338.468416	0.48147078	0.16690667	2.88467066	0.01803275
Thioredoxin 2	360.077826	0.47858561	0.14246753	3.359261	0.00508375
NADH-ubiquinone oxidoreductase chain I	2229.19108	0.4784997	0.16263934	2.94209083	0.01563409
Inner membrane protein YhjD	1414.12741	0.47803245	0.1169434	4.08772486	0.00044686
Protein of unknown function DUF541	2420.95526	0.47713611	0.10404914	4.58568069	6.41E-05
FIG00637886: hypothetical protein	238.100762	0.47704179	0.18568994	2.56902334	0.0392199
Selenoprotein O and cysteine-containing homologs	6337.53539	0.47473569	0.12671254	3.74655645	0.00149421
Protein ydhR precursor	1951.32425	0.47426838	0.14974082	3.16726192	0.00878715
Glutamine ABC transporter%2C periplasmic glutamine-binding protein (TC 3.A.1.3.2)	11223.3292	0.47394658	0.09888089	4.7931061	2.77E-05
Glycine dehydrogenase [decarboxylating] (glycine cleavage system P protein)	1761.2075	0.47293644	0.14339849	3.2980573	0.00605505
Sensory histidine kinase QseC	223.159323	0.4718179	0.14034206	3.36191367	0.00507051
Regulatory protein SoxS	414.581892	0.47176936	0.18512914	2.54832577	0.04113715
Cytoplasmic protein YaiE	2973.66561	0.47161282	0.15044233	3.13484123	0.0096108
Glutathione S-transferase%2C omega	1296.59413	0.47135816	0.1202638	3.91936862	0.00081983
Glutamate Aspartate periplasmic binding protein precursor GltI (TC 3.A.1.3.4)	1076.19628	0.47135568	0.14997028	3.14299387	0.00943057
18K peptidoglycan-associated outer membrane lipoprotein%3B Peptidoglycan-associated lipoprotein precursor%3B Outer membrane protein P6%3B OmpA/MotB precursor	17544.7665	0.47040944	0.12871806	3.65457231	0.00202136
Tellurite resistance protein TehB	1002.28318	0.46952026	0.13694146	3.42862027	0.00409313
LysR family transcriptional regulator YdcI	637.279224	0.46937754	0.18192381	2.58007752	0.03814822
Acetolactate synthase large subunit	3538.4636	0.46757254	0.14875407	3.14325885	0.00943057
Glucokinase%2C ROK family	654.117656	0.46255874	0.17276156	2.67744015	0.03016721
L-fuculokinase	787.178188	0.46203977	0.15011622	3.0778804	0.01108138
Cell division protein BolA	10384.5324	0.46104555	0.15386315	2.99646516	0.01371394
Molybdenum cofactor biosynthesis protein MoaD	396.298329	0.46102421	0.16768024	2.74942488	0.02558363
FIG004088: inner membrane protein YebE	735.467402	0.46077435	0.155293	2.96712893	0.01463907
Uridine phosphorylase	16627.7282	0.4607062	0.15134865	3.044006	0.01206915
Phosphoenolpyruvate-dihydroxyacetone phosphotransferase operon regulatory protein DhaR	586.630625	0.46038235	0.17372565	2.65005398	0.03229127
L-proline glycine betaine ABC transport system permease protein ProV (TC 3.A.1.12.1)	2307.82192	0.45968072	0.13327962	3.44899481	0.00384793
probable ribonuclease inhibitor YPO3690	408.587287	0.45791123	0.18064542	2.53486209	0.04246769
Xylose ABC transporter%2C permease protein XylH	181.717554	0.45693732	0.17064995	2.67762933	0.03016721
probable haloacid dehalogenase-like hydrolase STY3852	1111.23176	0.45594455	0.10501712	4.34162113	0.00017238
Cyclic di-GMP binding protein precursor	12291.5867	0.45584262	0.11462254	3.9769022	0.00066738
Universal stress protein A	26023.6266	0.4540446	0.12809082	3.5447084	0.00288362
Starvation lipoprotein Slp paralog	2174.00246	0.4539405	0.16162977	2.80852028	0.02205826
Phosphate transport ATP-binding protein PstB (TC 3.A.1.7.1)	654.781198	0.45351911	0.17118376	2.6493116	0.03233369
Phage tail length tape-measure protein 1	780.521689	0.45345718	0.12968089	3.49671562	0.00334737
Lysine decarboxylase 2%2C constitutive	3746.41009	0.4525391	0.16819129	2.6906214	0.02936973
Outer membrane stress sensor protease DegQ%2Cserine protease	2757.1608	0.45234288	0.0985757	4.58878705	6.36E-05
Outer membrane lipoprotein Blc	3965.65692	0.45012183	0.14298413	3.1480544	0.00931517
Two-component system response regulator OmpR	3048.85162	0.44985873	0.09709549	4.63315801	5.35E-05
Low-specificity L-threonine aldolase	4846.48671	0.44926259	0.15172151	2.96110011	0.01483311
Glutamate Aspartate transport system permease protein GltJ (TC 3.A.1.3.4)	288.548348	0.44874026	0.15030901	2.98545144	0.01408456
3'(2')%2C5'-bisphosphate nucleotidase	9070.43849	0.44778913	0.14981921	2.98886327	0.01396698
FIG00639383: hypothetical protein	179.929717	0.44698108	0.17903555	2.49660512	0.04625805
Cytidine deaminase	4469.08318	0.44600117	0.15305559	2.91398152	0.01685468
Glutathione-regulated potassium-efflux system protein KefB	1437.39156	0.44412694	0.11950492	3.71639058	0.00165691
VgrG protein	196.046952	0.44395451	0.17353671	2.55827434	0.04021462
Phenylalanyl-tRNA synthetase beta chain	13384.9145	0.443913	0.12360326	3.59143434	0.00248395
probable lipoprotein	4137.76408	0.44374111	0.09489502	4.67612667	4.48E-05
Dienelactone hydrolase family	822.38914	0.44274861	0.13567862	3.26321571	0.00666817
D-alanyl-D-alanine carboxypeptidase	5564.83805	0.43939776	0.09668491	4.54463633	7.45E-05
Glutamate transport membrane-spanning protein	2368.50961	0.43907876	0.11634342	3.77398873	0.001359
Universal stress protein D	6040.65595	0.43699321	0.10195606	4.28609336	0.00021304
Oligopeptidase A	10779.4145	0.43458992	0.1111636	3.90946259	0.0008458
Peptide transport system permease protein SapC	926.86015	0.4336782	0.13770025	3.14943648	0.00928282
Ribonucleotide reductase of class Ib (aerobic)%2Cbeta subunit	8609.17305	0.4326745	0.12877019	3.36005171	0.00507651
Ribonuclease E	13430.6596	0.43228378	0.09510128	4.54550965	7.44E-05
Probable glutathione S-transferase	617.085454	0.4322009	0.14936591	2.89357122	0.0176399
Aerobactin siderophore receptor IutA @ TonB-dependent siderophore receptor	665164.934	0.43120166	0.1683854	2.56080187	0.03995693
L-arabinose transport system permease protein (TC 3.A.1.2.2)	359.974328	0.43086393	0.15373086	2.80271599	0.0223282
Nucleoside permease NupC	2938.61785	0.4301027	0.09627847	4.46727805	0.00010292
PsiE protein	1544.81619	0.42996738	0.11722793	3.66778949	0.00193253
Yersiniabactin synthetase%2C thiazolinyl reductase component Irp3	35839.0353	0.42991023	0.14455587	2.9740074	0.01439217
C4-type zinc finger protein%2C DksA/TraR family	7371.65299	0.42897702	0.10661075	4.0237687	0.00056575
2-amino-3-ketobutyrate coenzyme A ligase	7183.10621	0.42893257	0.11772001	3.64366735	0.00209031
L-proline glycine betaine ABC transport system permease protein ProW (TC 3.A.1.12.1)	1843.97681	0.42884492	0.15809109	2.71264444	0.02789295
Peptidase B	4552.42961	0.42813092	0.10013769	4.27542226	0.00022236
Phage tail fiber protein	306.360958	0.42710636	0.14587582	2.92787636	0.01620303
Folate-dependent protein for Fe/S cluster synthesis/repair in oxidative stress	3815.71373	0.42706289	0.10004543	4.26868971	0.00022684
FIG00638355: hypothetical protein	4655.16972	0.42589696	0.16089086	2.64711716	0.03251552
PTS system%2C galactitol-specific IIC component	213.487821	0.42515039	0.17251417	2.46443746	0.0494197
Carbonic anhydrase	3236.36728	0.42509801	0.17166179	2.47636946	0.04825825
Alcohol dehydrogenase	3092.61915	0.42277227	0.16356835	2.5846827	0.03773893
Dipeptide transport system permease protein DppC (TC 3.A.1.5.2)	1014.67431	0.42197229	0.12508773	3.37341068	0.00488561
Prophage Clp protease-like protein	312.878011	0.42105212	0.13457559	3.12874062	0.0097577
Putative membrane protein	11272.6171	0.42048718	0.12394317	3.39258057	0.00460298
Respiratory nitrate reductase gamma chain	233.859953	0.41754332	0.16826609	2.48144663	0.04772931
Hexuronate utilization operon transcriptional repressor ExuR	4572.29855	0.41743689	0.10356422	4.03070565	0.00055172
UDP-glucose:(glucosyl)lipopolysaccharide alpha-1%2C3-glucosyltransferase WaaO	2769.57271	0.41677682	0.1443162	2.88794193	0.01786744
Biotin synthesis protein BioC	2194.65148	0.4165623	0.13175445	3.16165651	0.00892433
Outer membrane lipoprotein pcp precursor	12891.5605	0.41612304	0.11552087	3.60214605	0.00240898
Ethanolamine utilization protein EutA	279.495054	0.416054	0.15524178	2.68003876	0.03004178
Anthranilate synthase%2C aminase component	50901.3109	0.41480055	0.12533127	3.3096333	0.00588276
FIG137360: hypothetical protein	1521.9782	0.41470397	0.10019774	4.13885555	0.00037325
Glutaredoxin 2	12587.0185	0.4137491	0.16441965	2.51642128	0.04434992
FIG00948312: hypothetical protein	1774.685	0.41365311	0.13856364	2.98529333	0.01408456
Endonuclease IV	953.783859	0.41224416	0.10247608	4.02283297	0.00056677
Uncharacterized PLP-dependent aminotransferase YfdZ	3449.0628	0.41213974	0.11013119	3.74226186	0.00151442
Puative phophotriesterase	823.065484	0.40906708	0.14114571	2.89818983	0.0175024
Dihydroxy-acid dehydratase	9855.09434	0.40846168	0.09886447	4.13153149	0.00038174
Potassium efflux system KefA protein / Small-conductance mechanosensitive channel	1077.89609	0.40819844	0.13349995	3.05766723	0.01169233
Aldo-keto reductase	260.124184	0.40637456	0.14612906	2.7809292	0.02358531
N-ethylmaleimide reductase	1636.29542	0.40495325	0.10792269	3.75225294	0.00147147
UDP-N-acetylmuramoylalanyl-D-glutamyl-2%2C6-diaminopimelate--D-alanyl-D-alanine ligase	10800.3694	0.40452152	0.0949396	4.26083042	0.00023378
Thioredoxin reductase	6796.12639	0.40291308	0.11159923	3.610357	0.00234583
Alkaline phosphatase	736.890396	0.40274912	0.11305343	3.56246714	0.00273519
Dihydroneopterin triphosphate epimerase	902.131327	0.40184087	0.11342307	3.54284956	0.00289933
Glucose-1-phosphatase	4436.28898	0.40175039	0.13822653	2.9064637	0.01721033
ABC transporter%2C periplasmic spermidine putrescine-binding protein PotD (TC 3.A.1.11.1)	2702.71523	0.39981986	0.10664896	3.74893368	0.00148284
Phage major capsid protein	648.21835	0.39941175	0.15162372	2.63422994	0.03356846
Head-tail preconnector protein GP5	786.0946	0.39928028	0.1167954	3.41863025	0.00423379
6-phospho-beta-glucosidase	5235.91818	0.39753043	0.08589689	4.62799549	5.45E-05
FIG00638703: hypothetical protein	1828.02557	0.39721129	0.08693328	4.569151	6.79E-05
Inner membrane protein YphA	3594.56151	0.3958387	0.14824192	2.67022108	0.03074089
Predicted exported alpha-N-acetylgalactosaminidase	2691.27425	0.39531982	0.12458656	3.17305359	0.00864639
Putative exported protein	3584.81863	0.393911	0.11278275	3.49265287	0.00338809
hypothetical tRNA/rRNA methyltransferase yfiF	2383.61581	0.39230849	0.1115737	3.51613759	0.00316457
L-aspartate oxidase	7405.48237	0.39190232	0.13333331	2.93926778	0.01573454
putative transport	773.502472	0.39131462	0.13127742	2.98082195	0.01418917
FIG00637900: hypothetical protein	1313.64197	0.39130772	0.10143619	3.85767346	0.0010237

Biotin synthase	2133.73021	0.38858956	0.11932925	3.25644849	0.00680193
Ferrichrome-iron receptor	3111.27662	0.38780617	0.09913365	3.91195305	0.00084028
UDP-glucose:(glucosyl)lipopolysaccharide alpha-1%2C2-glucosyltransferase	2749.06834	0.38477126	0.13339712	2.88440462	0.01803275
Succinyl-CoA synthetase%2C alpha subunit-related enzymes	5298.82325	0.38375607	0.13711188	2.79885363	0.02255319
Leucine-responsive regulatory protein%2C regulator for leucine (or lrp) regulon and high-affinity branched-chain amino acid transport system	7235.38262	0.38285025	0.15261578	2.5085889	0.04508502
Cytosine deaminase	322.012506	0.38206705	0.14058403	2.71771293	0.02754545
NAD(P)HX epimerase / NAD(P)HX dehydratase	3585.65554	0.37623199	0.13332349	2.8219483	0.02128032
Copper-sensing two-component system response regulator CpxR	4985.9851	0.37555163	0.11480108	3.27132484	0.00651465
COG0699: Predicted GTPases (dynamin-related)	679.071126	0.37271223	0.14917144	2.49854943	0.04611764
Dipeptide-binding ABC transporter%2C periplasmic substrate-binding component (TC 3.A.1.5.2)%3B Putative hemin-binding lipoprotein	3670.86038	0.37026986	0.10408501	3.55737942	0.00277502
Ribonucleotide reductase of class Ib (aerobic)%2Calpha subunit	15397.4363	0.36943584	0.13745411	2.68770317	0.02950264
Multidrug transporter MdtC	880.082375	0.368964	0.13829472	2.66795441	0.03087782
Transcriptional regulator%2C GntR family	906.82304	0.36828146	0.12975154	2.83835915	0.02035637
Oxidoreductase (putative)	595.396297	0.36805313	0.14148807	2.60130143	0.03629968
UDP-sugar hydrolase	3991.03917	0.36580567	0.12246384	2.98705037	0.01403464
TonB-dependent hemin %2C ferrichrome receptor	121631.308	0.36553449	0.11120473	3.28704096	0.00624558
Universal stress protein F	2490.19556	0.36492493	0.14621878	2.49574595	0.04633254
NADPH-dependent broad range aldehyde dehydrogenase YqhD	1419.77451	0.36420265	0.11797633	3.08708238	0.01079464
Cell division inhibitor	2148.20243	0.3636589	0.11709754	3.10560665	0.01032641
Protein ImpG/VasA	1241.88724	0.36249076	0.11720062	3.09290826	0.0106603
FIG00639264: hypothetical protein	2005.25057	0.36195491	0.0943573	3.83600336	0.00109289
Endoglucanase precursor	5034.57107	0.36103035	0.08363046	4.31697185	0.00018874
Proline/sodium symporter PutP (TC 2.A.21.2.1) @ Propionate/sodium symporter	1914.07318	0.3598731	0.11820008	3.04460952	0.01205871
33 kDa chaperonin (Heat shock protein 33) (HSP33)	1544.42034	0.35741661	0.10889286	3.28227783	0.00630913
Aerobic respiration control protein arcA	10836.2333	0.35623853	0.12475574	2.85548808	0.01946347
Exodeoxyribonuclease V alpha chain	2842.43835	0.35320853	0.11108885	3.1795137	0.0084774
Integration host factor beta subunit	4694.0861	0.35299561	0.13518992	2.61110893	0.03555079
Biosynthetic Aromatic amino acid aminotransferase alpha	2781.73315	0.35206131	0.11869542	2.96609014	0.01467284
Putative minor tail protein	327.139611	0.35114234	0.14087404	2.49259792	0.04659378
Lipopolysaccharide core biosynthesis protein WaaP	2275.31164	0.35042292	0.12122858	2.89059664	0.01776499
Phosphoenolpyruvate-dihydroxyacetone phosphotransferase	1567.74285	0.34994495	0.11312266	3.09350006	0.0106603
Glucose-1-phosphate adenylyltransferase	25115.2158	0.3479794	0.13049773	2.66655511	0.03093982
COG2005: N-terminal domain of molybdenum-binding protein	3908.44762	0.3477448	0.13613091	2.55448815	0.04058619
Type I secretion outer membrane protein%2C TolC precursor	11306.2219	0.34756723	0.10153302	3.423194	0.00416952
NAD(P) transhydrogenase alpha subunit	9983.75347	0.34754371	0.10811033	3.21471315	0.00763905
CFA/I fimbrial major subunit	2407.7359	0.34645523	0.13597794	2.54787828	0.04115546
Mannitol-1-phosphate 5-dehydrogenase	2552.45772	0.34146442	0.12396417	2.75454118	0.0252591
Zinc transporter ZupT	2740.84224	0.33930692	0.10313454	3.28994453	0.00619835
NAD-dependent malic enzyme	13031.7786	0.33810208	0.08515919	3.97023599	0.00068106
Uncharacterized protein YhjG	3117.75351	0.33808595	0.11640396	2.90441975	0.01726931
Threonine dehydratase biosynthetic	6341.42057	0.33799374	0.10957404	3.08461511	0.01085899
Sensory histidine kinase BaeS	707.163136	0.33628665	0.12140923	2.76986067	0.0243086
Cation transport protein chaC	4194.57001	0.33564548	0.10694029	3.13862513	0.00953671
Putative inner membrane protein	363.400653	0.33561198	0.13617661	2.46453466	0.0494197
Ferredoxin--NADP(+) reductase	2537.41444	0.33302898	0.10436049	3.19114052	0.00818502
Peptide transport system ATP-binding protein SapF	1106.25809	0.33267857	0.11166415	2.97927823	0.01423914
Aldo-keto reductase	2872.89569	0.33190288	0.12727619	2.60773744	0.03577849
Universal stress protein G	18960.6928	0.33175761	0.09336322	3.55340778	0.00280352
Glycerol kinase	1468.29804	0.33040174	0.0972417	3.39773728	0.00453705
Phosphoribosyl-AMP cyclohydrolase	3322.8656	0.3303115	0.1104111	2.99165129	0.01390124
Alpha-2-macroglobulin	16164.3005	0.32985934	0.08907713	3.70307532	0.00172148
Allophanate hydrolase 2 subunit 1	4079.49244	0.32895991	0.09381375	3.50652107	0.00325706
Putative oxidoreductase	4170.36608	0.32832661	0.13244065	2.47904712	0.04797442
putative capsid protein of prophage	771.565706	0.32717073	0.10646022	3.07317365	0.01121822
Cell division inhibitor	1986.58028	0.32587843	0.09430967	3.45540833	0.00378035
Putative deoxyribonuclease YjjV	984.326996	0.32146025	0.10438154	3.07966555	0.01102811
Zinc transporter ZitB	952.625088	0.32055822	0.11727415	2.73340908	0.02648668
Protein export cytoplasm chaperone protein (SecB%2Cmaintains protein to be exported in unfolded state)	3674.792	0.31734556	0.07784051	4.07686899	0.00046196
Trilactone hydrolase IroD	29549.542	0.31716285	0.10013712	3.16728564	0.00878715
Putative ABC transporter ATP-binding protein	1598.17271	0.31517834	0.11722781	2.68859692	0.02946959
Sodium/glutamate symport protein	2345.70563	0.31329563	0.11020234	2.84291279	0.02008769
FIG00905232: hypothetical protein	900.43022	0.31266784	0.10536059	2.96759766	0.01463248
Kup system potassium uptake protein	961.484909	0.31254692	0.10725831	2.9139647	0.01685468
DJ-1/YajL/PfpI superfamily%2C includes chaperone protein YajL (former ThiJ)%2C parkinsonism-associated protein DJ-1%2C peptidases PfpI%2C Hsp31	2438.93029	0.31149794	0.10710281	2.90840126	0.01713965
Carbon starvation protein A	7856.40953	0.3094549	0.11781351	2.62665035	0.03415153
Outer membrane protein H precursor	10553.0962	0.30734058	0.08910318	3.44926614	0.00384793
Pyruvate formate-lyase	683.739988	0.30723985	0.12267077	2.50458893	0.04556067
Anthranilate phosphoribosyltransferase like	6667.04225	0.3036475	0.08713299	3.48487425	0.00344506
Phosphate starvation-inducible ATPase PhoH with RNA binding motif	8740.90495	0.30000817	0.09982325	3.00539384	0.01337692
Uncharacterized ABC transporter%2C auxiliary component YrbC	3379.93046	0.29446597	0.10026536	2.9368664	0.01584019
FIG00639949: hypothetical protein	1599.03357	0.29362095	0.11892419	2.46897578	0.04895289
Metal-dependent hydrolase YbeY%2C involved in rRNA and/or ribosome maturation and assembly	4451.46932	0.29225368	0.10688769	2.73421271	0.02646603
Phage tail fiber protein	992.66438	0.29127641	0.11215913	2.59699254	0.0366319
Allophanate hydrolase 2 subunit 2	6627.57208	0.28830577	0.08662246	3.32830266	0.00555634
ADP-heptose synthase	4859.45148	0.28772567	0.09679243	2.97260502	0.01441139
Glutamate synthase [NADPH] large chain	24998.6417	0.28757755	0.10779795	2.66774615	0.03087782
Citrate:6-N-acetyl-6-N-hydroxy-L-lysine ligase%2Calpha subunit	72421.5159	0.28664685	0.10465681	2.7389222	0.02614407
Protein ydcF	765.871304	0.2852409	0.09913885	2.8771858	0.01839453
N6-hydroxylysine O-acetyltransferase	43587.9085	0.28430422	0.10266663	2.76919803	0.02433483
Acetylornithine deacetylase	2533.1881	0.28312461	0.09032283	3.13458535	0.0096108
FIG00732392: hypothetical protein	1492.05078	0.2825882	0.09884845	2.85880253	0.01928054
Uncharacterized protein conserved in bacteria	1292.21976	0.27802089	0.11057486	2.51432284	0.04450032
PTS IIA-like nitrogen-regulatory protein PtsN	2511.7562	0.27743452	0.09326874	2.97457141	0.01438127
Decarboxylase family protein	15099.976	0.27565263	0.10451827	2.63736318	0.03328917
Cob(I)alamin adenosyltransferase	1085.38936	0.27511589	0.11040345	2.49191386	0.04664581
Cys regulon transcriptional activator CysB	2530.00864	0.27471799	0.10177934	2.69915293	0.02883683
Deoxyguanosinetriphosphate triphosphohydrolase	1442.61976	0.27336662	0.08954871	3.05271423	0.01184595
Tail fiber assembly protein	963.584289	0.27308884	0.10663569	2.56095152	0.03995693
Virulence factor MviM	3475.76851	0.27028623	0.10395652	2.59999293	0.036407
FIG00639467: hypothetical protein	3653.81843	0.26529695	0.10386727	2.55419195	0.04058657
Putative oxidoreductase	1432.78258	0.2619519	0.0963745	2.71806238	0.02754183
Phosphodiesterase yfcE	1199.33021	0.2599605	0.10043569	2.58832783	0.03743778
Uncharacterized protein yhaV	1875.02658	0.25676618	0.10357356	2.47907086	0.04797442
D-cysteine desulfhydrase	4139.45158	0.25301974	0.10257203	2.46675185	0.04917958
Hemin transport protein HmuS	50669.0538	0.24337105	0.09057358	2.68699828	0.02950264
Universal stress protein E	17839.5434	0.23786677	0.09413102	2.5269754	0.04332514
Lipoprotein YcfM%2C part of a salvage pathway of unknown substrate	3274.75402	0.23022664	0.07217226	3.18996019	0.00820806
ATP-dependent Clp protease proteolytic subunit	9227.03233	0.22221429	0.07328421	3.03222609	0.01250752
Protein of unknown function YceH	3515.21561	0.20567957	0.08355639	2.46156611	0.04963642
Peptide deformylase	3566.81895	-0.2111542	0.08182493	-2.5805612	0.0381273
Arabinose 5-phosphate isomerase	2427.98721	-0.2276115	0.08920756	-2.5514823	0.04080059
GTP-binding protein EngA	3024.16832	-0.2416301	0.09757351	-2.4763903	0.04825825
Replicative DNA helicase	2044.61918	-0.2421289	0.09672377	-2.5033029	0.04568912
Protein-L-isoaspartate O-methyltransferase	1318.07511	-0.24288	0.09727394	-2.496866	0.04625805
UDP-3-O-[3-hydroxymyristoyl] glucosamine N-acyltransferase	12864.3395	-0.2430837	0.09871284	-2.4625334	0.04960408
Phosphatidate cytidylyltransferase	1962.73065	-0.2469068	0.09340516	-2.6433963	0.03281709
Per-activated serine protease autotransporter enterotoxin EspC	3169.43608	-0.2480679	0.07069484	-3.5089961	0.00323201
Translation elongation factor LepA	3942.01437	-0.248187	0.09940811	-2.4966477	0.04625805
3-polyprenyl-4-hydroxybenzoate carboxy-lyase	4379.80761	-0.2502879	0.10084327	-2.4819494	0.04770039

Lipoprotein releasing system transmembrane protein LolE	2192.38781	-0.257445	0.08586433	-2.9982769	0.01364778
Diaminohydroxyphosphoribosylaminopyrimidine deaminase	3562.97959	-0.2580045	0.09622391	-2.6812934	0.02995634
Nucleoid-associated protein NdpA	714.135391	-0.264039	0.10623985	-2.4853102	0.04736679
Arginine ABC transporter%2C permease protein ArtQ	2497.49701	-0.2651823	0.09659758	-2.7452269	0.02572801
3-oxoacyl-[acyl-carrier-protein] synthase%2C KASII	15282.951	-0.2682497	0.096377	-2.7833374	0.02345596
Lipopolysaccharide ABC transporter%2C ATP-binding protein LptB	3082.22687	-0.2686429	0.098337	-2.7318601	0.02656202
TrkA%2C Potassium channel-family protein	2280.23628	-0.2779617	0.10948081	-2.5389075	0.04208492
Putative capsular polysaccharide transport protein YegH	2525.49731	-0.2789117	0.09245528	-3.0167204	0.01304676
ABC transporter multidrug efflux pump%2C fused ATP-binding domains	17611.7659	-0.2792161	0.10347583	-2.6983699	0.02885196
Cobalt-zinc-cadmium resistance protein	1776.93698	-0.2799805	0.10550166	-2.6538014	0.03199149
Erythritol transcriptional regulator EryD	2067.57423	-0.2802643	0.11149135	-2.5137763	0.04450032
1-deoxy-D-xylulose 5-phosphate reductoisomerase	1812.74813	-0.2806275	0.11390826	-2.4636277	0.04949217
Single-stranded-DNA-specific exonuclease RecJ	2834.02488	-0.2810256	0.09415222	-2.9848006	0.01409181
Ferric enterobactin transport ATP-binding protein FepC (TC 3.A.1.14.2)	1915.33755	-0.2821456	0.10000401	-2.821343	0.02129958
Histidyl-tRNA synthetase	5334.70146	-0.2835926	0.09037905	-3.1378139	0.0095513
Methionyl-tRNA formyltransferase	3972.07394	-0.2843364	0.08539891	-3.3295085	0.00554789
Octaprenyl diphosphate synthase	2348.6002	-0.2870371	0.09358401	-3.0671596	0.01139318
ATP-dependent helicase HrpB	2086.75936	-0.2871325	0.09804765	-2.9284995	0.01618754
Tail-specific protease precursor	8358.14633	-0.2902913	0.10144596	-2.8615362	0.01917256
Siroheme synthase / Precorrin-2 oxidase	975.472677	-0.2917903	0.10704008	-2.7259909	0.02701386
Macrolide export ATP-binding/permease protein MacB	1925.70797	-0.2935998	0.09965183	-2.9462555	0.01549783
Dihydrofolate synthase	983.611077	-0.2936667	0.09905847	-2.9645791	0.01472156
Cysteinyl-tRNA synthetase	1715.77138	-0.294965	0.0981369	-3.0056486	0.01337692
mobilization protein	4242.62165	-0.2953154	0.08404466	-3.5137907	0.00318433
N-acetylglucosamine-1-phosphate uridyltransferase	3549.80828	-0.295592	0.09093808	-3.2504749	0.00689147
Dephospho-CoA kinase	941.119644	-0.2970105	0.10620849	-2.7964855	0.02263168
N-acetylmuramoyl-L-alanine amidase	1190.0491	-0.2978283	0.11120811	-2.6781165	0.03016721
CTP synthase	17574.5916	-0.2981852	0.10589449	-2.8158704	0.02160213
Two-component sensor protein RcsC	1723.52738	-0.3014	0.09230918	-3.2651138	0.00663296
Cell division protein FtsA	6860.22694	-0.3053249	0.1134169	-2.692058	0.02929673
Enoyl-[acyl-carrier-protein] reductase [NADH]	4344.69328	-0.3082947	0.10895872	-2.8294632	0.02084842
1-deoxy-D-xylulose 5-phosphate synthase	1570.01366	-0.3099144	0.0989958	-3.1305813	0.00972707
23S rRNA (guanosine-2'-O-)-methyltransferase rlmB	3013.06721	-0.3116581	0.11201139	-2.7823783	0.0235028
Inorganic pyrophosphatase	2973.35257	-0.3124914	0.12046561	-2.5940299	0.03691736
Thiamine-monophosphate kinase	1153.54481	-0.3134073	0.10741504	-2.9177224	0.01668768
FIG001881: hydrolase of alkaline phosphatase superfamily	1844.82455	-0.3148389	0.09736724	-3.2335204	0.00723751
ATP synthase B chain	5984.05388	-0.3153332	0.09879611	-3.1917574	0.00817797
UDP-glucose dehydrogenase	2006.83238	-0.3160118	0.11456515	-2.758359	0.02503714
Methyl-directed repair DNA adenine methylase	1159.29132	-0.3164483	0.10801189	-2.9297541	0.0161562
Succinate dehydrogenase flavoprotein subunit	493.962692	-0.3165248	0.12549756	-2.5221593	0.04385612
tRNA pseudouridine synthase A	1887.35024	-0.3182781	0.10985941	-2.89714	0.01751201
GMP synthase [glutamine-hydrolyzing]%2Camidotransferase subunit	4251.53701	-0.3185239	0.10125276	-3.1458295	0.0093746
Guanylate kinase	3532.48379	-0.3187425	0.10686058	-2.9827885	0.01413833
Negative regulator of allantoin and glyoxylate utilization operons	5414.91526	-0.3195016	0.10635268	-3.0041704	0.01341591
Transcriptional regulator%2C GntR family domain / Aspartate aminotransferase	1069.25631	-0.3196477	0.10176708	-3.1409738	0.00948406
Lysophospholipase L2	1558.98966	-0.3207828	0.11800492	-2.7183848	0.02754048
LSU ribosomal protein L36p	2508.84459	-0.3230265	0.12275265	-2.6315236	0.03380732
Sensor kinase CitA%2C DpiB	716.647677	-0.3240607	0.10431153	-3.1066623	0.01032358
TsaD/Kae1/Qri7 protein%2C required for threonylcarbamoyladenosine t(6)A37 formation in tRNA	995.519641	-0.324418	0.12116605	-2.6774661	0.03016721
DNA polymerase III beta subunit	2093.65743	-0.325939	0.09705694	-3.3582247	0.00509554
putative alpha helix protein	700.533476	-0.3260937	0.13196105	-2.4711359	0.04873584
Uncharacterized acetyltransferase YjgM	464.036068	-0.3275551	0.13307418	-2.4614475	0.04963642
GTP pyrophosphokinase	6226.87523	-0.3297944	0.0925191	-3.5646091	0.00271741
tRNA (5-methoxyuridine) 34 synthase	1784.42246	-0.330685	0.12987973	-2.5460865	0.0413327
LPS-assembly lipoprotein RlpB precursor (Rare lipoprotein B)	3613.32102	-0.3317455	0.10845564	-3.0588126	0.0116612
FIG021862: membrane protein%2C exporter	1349.93672	-0.3317532	0.11057342	-3.0002979	0.0135726
regulator of length of O-antigen component of lipopolysaccharide chains	1974.88607	-0.331989	0.09952729	-3.3356582	0.00545733
Heat shock (predicted periplasmic) protein YciM%2Cprecursor	2453.73308	-0.3332624	0.12055882	-2.7643135	0.02466967
Radical SAM family protein HutW%2C similar to coproporphyrinogen III oxidase%2Coxygen-independent%2Cassociated with heme uptake	8274.20449	-0.3343851	0.12416641	-2.6930396	0.02923722
UDP-N-acetylglucosamine 1-carboxyvinyltransferase	1607.16978	-0.3347149	0.10842187	-3.0871534	0.01079464
(3R)-hydroxymyristoyl-[acyl carrier protein] dehydratase	3441.52865	-0.3356062	0.11891495	-2.8222372	0.02128032
DNA-directed RNA polymerase alpha subunit	37339.433	-0.3358465	0.10878654	-3.087206	0.01079464
Arginine ABC transporter%2C permease protein ArtM	1287.17123	-0.3365325	0.09775022	-3.4427794	0.00391972
S-adenosylmethionine decarboxylase proenzyme	1687.38694	-0.3384595	0.11348633	-2.9823812	0.01414171
Putative DNA processing chain A	850.495311	-0.3407231	0.12771917	-2.6677524	0.03087782
Undecaprenyl-phosphate N-acetylglucosaminyl 1-phosphate transferase	1516.98029	-0.3407243	0.1191146	-2.8604743	0.01921761
UDP-N-acetylmuramate:L-alanyl-gamma-D-glutamyl-meso-diaminopimelate ligase	1961.35616	-0.34166	0.12599527	-2.7116891	0.02792199
Proline-specific permease proY	1097.36704	-0.34263	0.10721414	-3.195754	0.00808617
Putative ATPase component of ABC transporter with duplicated ATPase domain	2054.96573	-0.3426927	0.11357783	-3.01725	0.01303867
Transport ATP-binding protein CydC	2549.14784	-0.3427741	0.11087338	-3.0915817	0.01068611
FIG00638108: hypothetical protein	1220.14244	-0.343541	0.13188475	-2.6048577	0.03601823
Biotin carboxyl carrier protein of acetyl-CoA carboxylase	2298.80722	-0.343594	0.10623835	-3.2341807	0.00723023
DedA protein	657.649401	-0.3440668	0.11093046	-3.1016443	0.01041246
DNA recombination and repair protein RecO	1610.16568	-0.3449583	0.11091707	-3.1100561	0.01023031
Phosphatase YidA	2895.52267	-0.3455264	0.11539306	-2.9943432	0.01379441
Periplasmic esterase IroE	4491.76123	-0.3459116	0.13367753	-2.5876574	0.03747863
FIG143263: Glycosyl transferase / Lysophospholipid acyltransferase	2119.98047	-0.3461335	0.11769586	-2.940915	0.01566764
DNA polymerase III subunits gamma and tau	1875.44398	-0.3464444	0.09914935	-3.4941671	0.0033742
Transcriptional regulator%2C TetR family	685.441197	-0.3478279	0.13505947	-2.5753682	0.03860586
Transcription repressor	477.120408	-0.3497357	0.13973272	-2.5028907	0.04570494
Crotonobetaine carnitine-CoA ligase	550.951222	-0.3505397	0.13156697	-2.6643439	0.03111625
RNA:NAD 2'-phosphotransferase	440.136398	-0.3515168	0.12147115	-2.8938297	0.0176399
Transcriptional regulator%2C LacI family	1841.09868	-0.3527982	0.12190559	-2.8940285	0.0176399
Purine nucleotide synthesis repressor	843.07134	-0.3533557	0.13845406	-2.5521514	0.04075651
ATP-dependent DNA helicase RecQ	738.45156	-0.3540788	0.12655776	-2.7977646	0.02260744
Hemolysins and related proteins containing CBS domains	931.87352	-0.3543157	0.12078057	-2.933549	0.01597679
Ribokinase	827.496151	-0.3546802	0.13830533	-2.5644723	0.03967031
Mlr7403 protein	3121.13906	-0.3572431	0.12076143	-2.9582552	0.01495475
Putative ATP-binding component of a transport system	757.873836	-0.3576655	0.13303138	-2.6885804	0.02946959
Lysine-specific permease	1830.19816	-0.3578647	0.11471623	-3.1195651	0.00997801
Glutamate synthase [NADPH] small chain	240.127758	-0.3589645	0.14516324	-2.4728334	0.04854742
4-hydroxythreonine-4-phosphate dehydrogenase	2825.78284	-0.3602042	0.10606843	-3.3959606	0.00455988
Mn-dependent transcriptional regulator MntR	434.927621	-0.3607868	0.14351333	-2.5139601	0.04450032
Putative permease	885.858594	-0.3613553	0.13001299	-2.7793782	0.02365284
Ribosome recycling factor	6223.04501	-0.3628595	0.13807481	-2.627992	0.0340946
tRNA (uridine-5-oxyacetic acid methyl ester) 34 synthase	688.693032	-0.3631142	0.13824546	-2.6265905	0.03415153
Chaperone protein HscA	5334.96913	-0.3644543	0.13907563	-2.6205478	0.03464174
GGDEF/EAL domain protein YhjH	1287.79487	-0.3648129	0.14046484	-2.5971831	0.0366319
Cell volume regulation protein A	2456.37162	-0.3649466	0.10428882	-3.4993839	0.0033297
Preprotein translocase subunit YajC (TC 3.A.5.1.1)	1223.28176	-0.3696381	0.12962233	-2.8516544	0.01964087
Putative membrane protein	356.281465	-0.3709061	0.12245673	-3.0288751	0.01263273
Putative transport protein	367.824863	-0.3714695	0.11815893	-3.1438121	0.00942769
Methionine aminopeptidase	2909.26327	-0.371499	0.14529757	-2.5568151	0.04034968
DedA family inner membrane protein YghB	1238.41505	-0.3715263	0.13242619	-2.805535	0.02217767
Flagellar hook-associated protein FlgK	13092.2058	-0.3718597	0.15034618	-2.4733565	0.04854742
Translation elongation factor P	1785.40411	-0.3739858	0.14253572	-2.6238038	0.03440217
Intramembrane protease RasP/YluC%2C implicated in cell division based on FtsL cleavage	5497.80479	-0.3763198	0.09030667	-4.1671314	0.00033795
Helicase PriA essential for oriC/DnaA-independent DNA replication	985.557407	-0.3766045	0.15313669	-2.45927	0.04989904
Putative membrane protein YeiH	435.300601	-0.3791093	0.14950698	-2.5357297	0.04239793

Transport ATP-binding protein CydD	1519.19925	-0.3795236	0.13805272	-2.749121	0.02558363
Formyltetrahydrofolate deformylase	2021.73119	-0.380133	0.14377577	-2.6439293	0.03279438
Flagellar motor rotation protein MotB	6405.18855	-0.3821519	0.10107438	-3.7808977	0.00133428
Mobile element protein	307.368263	-0.3836188	0.15344175	-2.5000943	0.0459545
hypothetical protein	754.064763	-0.3843729	0.13843959	-2.7764668	0.02384287
Phosphoheptose isomerase	1144.80799	-0.3872882	0.11434229	-3.3870948	0.00468228
mannose-specific adhesin FimH	8981.21702	-0.3908745	0.14983756	-2.6086548	0.03574463
DnaJ-like protein DjlA	508.761287	-0.3923599	0.14538876	-2.6986946	0.02885018
Malonyl CoA-acyl carrier protein transacylase	100135.788	-0.3936235	0.13045031	-3.0174209	0.01303867
Putative 3-hydroxyacyl-coa dehydrogenase	35263.207	-0.3943816	0.15969332	-2.4696188	0.04890396
Superoxide dismutase [Fe]	1003.89989	-0.3960929	0.14994765	-2.6415412	0.03296828
Ferric hydroxamate ABC transporter (TC 3.A.1.14.3)%2C permease component FhuB	1994.63147	-0.3960954	0.11899272	-3.3287366	0.00555548
Alanine transaminase	2222.7736	-0.3997793	0.1606035	-2.4892315	0.04696122
Hypothetical response regulatory protein ypdB	929.566183	-0.4002716	0.12959487	-3.0886376	0.01077637
Topoisomerase IV subunit B	2373.23924	-0.4003911	0.09726079	-4.1166751	0.00040249
Rod shape-determining protein RodA	849.049179	-0.4006775	0.13085797	-3.0619264	0.01156736
COG1272: Predicted membrane protein hemolysin III homolog	7051.30042	-0.4007365	0.12493708	-3.2075066	0.00781289
Ferric enterobactin transport system permease protein fepD	1786.77911	-0.4007924	0.14287427	-2.8052104	0.02217767
UPF0141 membrane protein YijP possibly required for phosphoethanolamine modification of lipopolysaccharide	2137.15611	-0.4012299	0.14871935	-2.6978994	0.02886641
Hydrolase%2C alpha/beta fold family functionally coupled to Phosphoribulokinase	415.270284	-0.4032717	0.14139922	-2.8520077	0.01963862
Probable sugar efflux transporter	1227.68775	-0.4033131	0.14006342	-2.8795038	0.01827833
YehQ protein	318.686561	-0.4037377	0.13561332	-2.9771242	0.01430341
ATP synthase delta chain	5544.83326	-0.4044741	0.12139491	-3.3318871	0.0055205
Type I restriction-modification system%2CDNA-methyltransferase subunit M	767.116031	-0.4054401	0.14553508	-2.7858581	0.02331919
Putative phosphatase	212.091335	-0.4058738	0.14690384	-2.7628538	0.024742
Similarity with glutathionylspermidine synthase	260.555657	-0.406741	0.13446722	-3.0248335	0.01277376
Hydrogenase-2 operon protein hybE	366.169071	-0.4072135	0.1168934	-3.4836311	0.00345577
Outer membrane protein YfgL%2C lipoprotein component of the protein assembly complex (forms a complex with YaeT%2C YfiO%2C and NlpB)	4136.41525	-0.4072954	0.10813442	-3.7665661	0.00139223
hypothetical protein	12726.1034	-0.4077892	0.10991564	-3.71002	0.00168742
TolA protein	1474.78108	-0.4092828	0.11607408	-3.5260487	0.0030698
Dihydroneopterin triphosphate pyrophosphohydrolase type 2 (nudB)	995.7997	-0.4095975	0.12629278	-3.243238	0.00703956
COG0613%2C Predicted metal-dependent phosphoesterases (PHP family)	582.563028	-0.4100787	0.15725092	-2.6077987	0.03577849
DNA polymerase III delta subunit	878.748332	-0.4104935	0.12989059	-3.1603021	0.00895469
Apolipoprotein N-acyltransferase	2055.91596	-0.4135042	0.11189085	-3.6956034	0.00176036
3-oxoacyl-(acyl carrier protein) synthase	164.918045	-0.4148123	0.15247314	-2.72056	0.0273853
Putative cytoplasmic protein	4255.31823	-0.4151049	0.16008097	-2.5930936	0.03698627
Molybdenum transport ATP-binding protein ModC (TC 3.A.1.8.1)	3586.1961	-0.4157076	0.13984379	-2.9726571	0.01441139
Methyl-accepting chemotaxis protein II (aspartate chemoreceptor protein)	29396.2755	-0.4162999	0.1356709	-3.0684536	0.01136269
FIG00639460: hypothetical protein	359.855596	-0.4164634	0.13972056	-2.9806882	0.01418917
FIG00638276: hypothetical protein	277.6754	-0.417977	0.14946538	-2.7964806	0.02263168
Cell division protein DivIC (FtsB)%2C stabilizes FtsL against RasP cleavage	317.03434	-0.4180968	0.15280028	-2.7362304	0.02633427
LSU ribosomal protein L17p	8056.39579	-0.4199558	0.11684942	-3.5939918	0.00247319
Phospholipase A1 precursor	581.22611	-0.4200017	0.13120763	-3.2010465	0.00796968
Dihydropteroate synthase	806.966311	-0.4216991	0.11740797	-3.5917419	0.00248395
Ribosomal large subunit pseudouridine synthase C	268.233042	-0.4229132	0.17035563	-2.4825312	0.04769938
Ribosomal large subunit pseudouridine synthase B	1120.0686	-0.4231648	0.12265589	-3.4500164	0.003845
YaaH protein	375.521196	-0.4239101	0.14868954	-2.8509745	0.01966334
SSU ribosomal protein S11p (S14e)	20266.9191	-0.4252203	0.11477833	-3.7047089	0.00171348
Uridine kinase	449.283873	-0.4255326	0.16899208	-2.5180621	0.04418029
D-glycero-D-manno-heptose 1%2C7-bisphosphate phosphatase	1440.26285	-0.428029	0.13302927	-3.2175552	0.00757514
[NiFe] hydrogenase metallocenter assembly protein HypD	343.314087	-0.4280918	0.17162187	-2.4943893	0.04641833
Putative inner membrane protein	196.903281	-0.4289131	0.1573872	-2.7252095	0.02705274
Nicotinate-nucleotide adenylyltransferase	676.220514	-0.4292999	0.14658719	-2.9286316	0.01618754
tRNA-specific 2-thiouridylase MnmA	1476.59262	-0.431787	0.14580417	-2.9614175	0.01483311
Lipid-A-disaccharide synthase	2286.72711	-0.4331732	0.14974429	-2.8927526	0.01766789
putative TYPE II DNA MODIFICATION ENZYME (METHYLTRANSFERASE)	1507.05642	-0.4339041	0.16344651	-2.6547163	0.03193317
Ribonuclease III	1470.58572	-0.4348039	0.12221899	-3.5575803	0.00277502
Ferrichrome transport ATP-binding protein FhuC (TC 3.A.1.14.3)	277.444223	-0.4359112	0.16341387	-2.6675287	0.03087782
Putative efflux (PET) family inner membrane protein YccS	674.722981	-0.4359477	0.10129699	-4.3036591	0.00019861
Hydrogenase-2 large chain precursor	1361.61788	-0.4360521	0.11159645	-3.907401	0.0008511
Putative heat shock protein YegD	469.972639	-0.4373164	0.12449734	-3.5126564	0.00319288
Nucleoside 5-triphosphatase RdgB (dHAPTP%2CdITP%2CXTP-specific)	422.615341	-0.4381554	0.12614134	-3.4735275	0.00356109
FIG00638687: hypothetical protein	307.28466	-0.4395677	0.17579645	-2.5004354	0.04594777
Vitamin B12 ABC transporter%2C B12-binding component BtuF	459.970678	-0.4405705	0.16910738	-2.6052706	0.03600597
Chromosomal replication initiator protein DnaA	4084.17936	-0.4409597	0.11414855	-3.8630338	0.00100346
Uncharacterized HTH-type transcriptional regulator YegW	380.716534	-0.4410182	0.14194554	-3.1069536	0.01032358
dTDP-glucose 4%2C6-dehydratase	1372.03878	-0.4412796	0.13187172	-3.3462794	0.00528819
Endonuclease VIII	863.660536	-0.4417581	0.13420524	-3.2916604	0.00616908
Uracil phosphoribosyltransferase	458.004326	-0.4422945	0.1788634	-2.4728062	0.04854742
Vitamin B12 ABC transporter%2C permease component BtuC	554.816702	-0.443149	0.13487834	-3.2855458	0.00627029
FIG004798: Putative cytoplasmic protein	410.346304	-0.4451963	0.12025919	-3.7019734	0.00172283
Diaminopimelate epimerase	2567.83745	-0.4452794	0.13419359	-3.3181866	0.0057374
Ribonucleotide reductase of class III (anaerobic)%2C activating protein	363.747098	-0.4457979	0.12469535	-3.5750964	0.00263242
RND efflux system%2C inner membrane transporter CmeB	408.571266	-0.4464405	0.1229968	-3.6296918	0.00219926
SSU rRNA (adenine(1518)-N(6)/adenine(1519)-N(6))-dimethyltransferase	2047.95125	-0.4464644	0.11042648	-4.0430917	0.00052565
Putative sulfate permease	557.365636	-0.44675	0.16044678	-2.7844126	0.02340084
ATP synthase A chain	3011.55297	-0.4485787	0.11256098	-3.9852058	0.00064765
Hydrogenase maturation protease	519.260871	-0.4495743	0.13962434	-3.2198853	0.0075611
NADH ubiquinone oxidoreductase chain A	1555.08101	-0.4502829	0.13893184	-3.2410344	0.00707661
Lysyl-tRNA synthetase (class II)	5966.69267	-0.4508126	0.10680495	-4.2208964	0.00027107
LSU ribosomal protein L21p	1394.96265	-0.450934	0.17585882	-2.564182	0.03967031
Flagellar biosynthesis protein FliS	3802.9257	-0.451132	0.12969826	-3.4783196	0.00350868
Fumarate reductase subunit C	430.714439	-0.4515985	0.15332161	-2.9454324	0.01549827
PspA/IM30 family protein	150.93653	-0.4551089	0.1830589	-2.4861338	0.04729548
Methyl-accepting chemotaxis protein I (serine chemoreceptor protein)	3714.31641	-0.4554469	0.10204597	-4.463154	0.00010462
Leucyl/phenylalanyl-tRNA--protein transferase	585.468572	-0.455872	0.1154437	-3.9488682	0.00073862
Putative cytoplasmic protein	413.677847	-0.4559921	0.13287272	-3.4317964	0.00405153
Protein of unknown function DUF484	1671.31847	-0.4565862	0.15022571	-3.0393345	0.01224394
YbbM seven transmembrane helix protein	419.180632	-0.4566252	0.17015393	-2.6836005	0.02977725
Uridylate kinase	1536.81814	-0.4571935	0.10624041	-4.303386	0.00019861
LSU ribosomal protein L18p (L5e)	9004.4284	-0.4579565	0.14925377	-3.0683081	0.01136269
1%2C4-dihydroxy-2-naphthoate octaprenyltransferase	323.160065	-0.458248	0.15816519	-2.8972746	0.01751201
Putative transport protein	776.253435	-0.458371	0.11735322	-3.9059089	0.00085465
Methyl-accepting chemotaxis protein IV (dipeptide chemoreceptor protein)	553.025431	-0.4590013	0.15859844	-2.8941096	0.0176399
Recombination protein RecR	1195.66453	-0.4599073	0.11938862	-3.852187	0.00103872
Adenine phosphoribosyltransferase	828.578794	-0.4599502	0.16110675	-2.8549409	0.01947758
hypothetical protein	5799.38316	-0.4608228	0.11674223	-3.9473531	0.00074177
23S rRNA (guanine-N-2-)-methyltransferase rlmG	360.988522	-0.463621	0.15956878	-2.9054618	0.01724762
Cytochrome d ubiquinol oxidase subunit I	28280.908	-0.4640963	0.18606833	-2.4942251	0.04641833
SSU ribosomal protein S13p (S18e)	11408.743	-0.4642153	0.1067431	-4.3489026	0.00016903
Guanosine-5'-triphosphate%2C3'-diphosphate pyrophosphatase	3962.42171	-0.4646213	0.13006731	-3.5721608	0.00264893
Probable lipoprotein nlpC precursor	1467.74209	-0.4648026	0.16871517	-2.7549544	0.02525116
3-oxoacyl-[acyl-carrier-protein] synthase%2CKASIII	4179.59095	-0.464852	0.11550271	-4.0245985	0.00056499
Cell division protein MraZ	1694.3965	-0.4648724	0.14073803	-3.3031046	0.00597169
Membrane protein YciC%2C linked to IspA	622.406768	-0.4654952	0.14138642	-3.2923615	0.00616214
SSU ribosomal protein S4p (S9e)	19848.4206	-0.4692468	0.10264251	-4.5716618	6.75E-05
HTH-type transcriptional regulator prsX	301.890568	-0.469942	0.14992355	-3.1345445	0.0096108
UDP-N-acetylglucosamine 2-epimerase	1657.99154	-0.4715598	0.10442329	-4.5158493	8.31E-05
3-hydroxydecanoyl-[acyl-carrier-protein] dehydratase	1456.975	-0.471824	0.13044954	-3.6169081	0.00229504

DNA mismatch repair endonuclease MutH	361.697096	-0.4727367	0.13544111	-3.4903485	0.00340679
Heat shock protein 60 family co-chaperone GroES	969.240892	-0.4743537	0.15224373	-3.1157517	0.01008344
Ribose operon repressor	629.248933	-0.474354	0.13805424	-3.4359969	0.00400085
Mobile element protein	156.149743	-0.4752622	0.17660806	-2.691056	0.02935816
FIG00639031: hypothetical protein	3162.10769	-0.4777307	0.08313782	-5.74625	2.72E-07
Undecaprenyl diphosphate synthase	1970.65321	-0.479649	0.10728718	-4.4707018	0.00010158
FIG002708: Protein SirB1	1055.62885	-0.4809197	0.15694443	-3.0642677	0.01149053
Putative transporting ATPase	432.635908	-0.4810618	0.14944489	-3.2189911	0.00756505
Biopolymer transport protein ExbD/TolR	5631.01149	-0.4816113	0.1155803	-4.1668977	0.00033795
Beta-lactamase	5206.86529	-0.4819089	0.13000914	-3.7067309	0.0017029
Glutamate racemase	1131.62133	-0.4823543	0.15548086	-3.1023386	0.01040048
Ferric hydroxamate ABC transporter (TC 3.A.1.14.3)%2C ATP-binding protein FhuC	1990.65658	-0.4823589	0.12516819	-3.8536857	0.0010344
Phenylalanyl-tRNA synthetase alpha chain	1900.91679	-0.4846682	0.14636207	-3.3114332	0.00586133
Mobile element protein	547.912043	-0.4848566	0.15683604	-3.0914871	0.01068611
6%2C7-dimethyl-8-ribityllumazine synthase	1962.59834	-0.4855632	0.09209149	-5.2726173	3.21E-06
Transcription termination protein NusB	2905.59854	-0.4869585	0.10105263	-4.81886	2.48E-05
Hemin ABC transporter%2C permease protein	1838.94768	-0.4877754	0.14014063	-3.480614	0.00348955
Pyridoxamine 5'-phosphate oxidase	995.643504	-0.4882235	0.17857658	-2.733973	0.02646603
Channel-forming transporter/cytolysins activator of TpsB family	694.178143	-0.4883224	0.15692987	-3.1117237	0.01019731
Putative transcriptional regulator	945.700155	-0.4887564	0.17682167	-2.7641203	0.02466967
Putative exported protein	1130.01363	-0.4890033	0.14316635	-3.4156302	0.00427436
Formate dehydrogenase O alpha subunit	1711.33501	-0.4906526	0.15674338	-3.1302923	0.00972707
DNA polymerase III alpha subunit	4809.90492	-0.4912938	0.11061892	-4.4413179	0.00011517
Chaperone protein HscB	1119.73263	-0.49141	0.16291493	-3.0163595	0.0130476
Acetylglutamate kinase	1840.57883	-0.4923377	0.13298124	-3.7023093	0.00172283
Protein YjgK%2C linked to biofilm formation	266.696471	-0.4932333	0.19027853	-2.5921647	0.03705452
Sensor protein torS	314.160686	-0.4948813	0.1537231	-3.2193036	0.00756505
Orotidine 5'-phosphate decarboxylase	357.87162	-0.4962682	0.13011141	-3.8141789	0.00117874
Tetraacyldisaccharide 4'-kinase	1275.86159	-0.4965055	0.11936359	-4.159606	0.0003458
Cytochrome c-type protein NapC	106.285985	-0.4965846	0.19072026	-2.6037326	0.03610547
Protein-export membrane protein SecF (TC 3.A.5.1.1)	3432.96216	-0.4978607	0.13330848	-3.7346514	0.0015581
Chemotaxis response regulator protein-glutamate methylesterase CheB	6258.90078	-0.499649	0.10882887	-4.5911439	6.31E-05
Adenylosuccinate synthetase	19221.6383	-0.4997691	0.13438084	-3.7190505	0.00164551
GTP-binding protein TypA/BipA	12716.5113	-0.4998341	0.10546113	-4.7395098	3.44E-05
Potassium efflux system KefA protein / Small-conductance mechanosensitive channel	2118.29753	-0.5003827	0.09899556	-5.0545978	8.85E-06
tRNA S(4)U 4-thiouridine synthase (former ThiI) / Rhodanese-like domain required for thiamine synthesis	694.427601	-0.50107	0.16174797	-3.0978444	0.01053432
Outer membrane vitamin B12 receptor BtuB	2791.90452	-0.5012433	0.11736544	-4.2707914	0.00022587
NADH-ubiquinone oxidoreductase chain B	2683.91032	-0.5021179	0.12540659	-4.0039196	0.00060747
Nitrate/nitrite sensor protein	558.149483	-0.5028273	0.1188766	-4.2298261	0.00026195
FIG01046632: hypothetical protein	182.685045	-0.5039169	0.18833101	-2.6756978	0.03027018
SSU ribosomal protein S15p (S13e)	2419.33737	-0.5042601	0.12848905	-3.9245378	0.0008057
DNA polymerase I	151.24013	-0.5068701	0.2011683	-2.519632	0.04402011
Transcription termination protein NusA	10468.444	-0.5077132	0.11767734	-4.3144517	0.0001904
Succinate dehydrogenase iron-sulfur protein	1018.95186	-0.50803	0.18498317	-2.7463579	0.02572724
Selenophosphate-dependent tRNA 2-selenouridine synthase	190.476827	-0.5092666	0.19296324	-2.6391899	0.03313944
UPF0125 protein yfjF	339.318558	-0.5102458	0.15043406	-3.3918234	0.00460896
DcrB protein precursor	2233.75397	-0.5112556	0.12810392	-3.9909446	0.00063486
Peptide chain release factor 2%3B programmed frameshift-containing	2810.13894	-0.5125746	0.15647166	-3.2758305	0.00642023
Twin-arginine translocation protein TatC	1280.1823	-0.5126379	0.13757371	-3.7262779	0.00160489
FIG00637874: hypothetical protein	233.505893	-0.5126396	0.2001654	-2.5610801	0.03995693
N-acetyl-gamma-glutamyl-phosphate reductase	2087.14565	-0.5142657	0.13952828	-3.6857454	0.00181706
SSU ribosomal protein S9p (S16e)	11014.3927	-0.5153609	0.12720088	-4.0515512	0.00050924
Ferrous iron transport protein B	20662.0601	-0.5165664	0.14808874	-3.4882217	0.00342024
Membrane-bound lytic murein transglycosylase A precursor	735.1036	-0.5168196	0.12637644	-4.089525	0.00044441
Ribosomal RNA large subunit methyltransferase F	346.705877	-0.5177215	0.16833723	-3.0755021	0.01115706
Spermidine Putrescine ABC transporter permease component PotB (TC 3.A.1.11.1)	511.701549	-0.5180375	0.17847706	-2.9025438	0.01733718
Acetylornithine aminotransferase	760.159676	-0.5190834	0.13301404	-3.9024708	0.00086169
Transglycosylase%2C Slt family	135.144978	-0.5196679	0.19819823	-2.6219603	0.03452855
MotA/TolQ/ExbB proton channel family protein	7973.28486	-0.51997	0.17942197	-2.8980288	0.0175024
Inner membrane protein	582.159296	-0.5206613	0.15946289	-3.265094	0.00663296
Uridine kinase family protein YggC homolog	345.736817	-0.5209683	0.16590914	-3.140082	0.00950118
FIG01219785: hypothetical protein	269.104079	-0.5216467	0.15665095	-3.3299938	0.00554604
S-adenosylmethionine:tRNA ribosyltransferase-isomerase	382.790245	-0.5231144	0.18098401	-2.8903898	0.01776499
Malonyl CoA-acyl carrier protein transacylase	34460.3559	-0.5242059	0.15526851	-3.3761247	0.0048447
Transcriptional regulator%2C ArsR family	352.94627	-0.5246724	0.19524186	-2.6872948	0.02950264
tRNA dihydrouridine synthase A	338.941955	-0.5254654	0.13657842	-3.8473532	0.00105529
Biotin carboxylase of acetyl-CoA carboxylase	3873.51692	-0.5277994	0.11348645	-4.6507704	4.98E-05
Rod shape-determining protein MreC	1012.4145	-0.5279664	0.17733653	-2.9772003	0.01430341
Transmembrane component STY3231 of energizing module of queuosine-regulated ECF transporter	97.684255	-0.5281452	0.20827401	-2.5358189	0.04239793
Tyrosine recombinase XerC	2146.35478	-0.5285944	0.19582519	-2.6993175	0.02883683
Butyryl-CoA dehydrogenase	36441.9106	-0.5287443	0.16429304	-3.2182999	0.00757348
C4-dicarboxylate transporter DcuC (TC 2.A.61.1.1)	151.677654	-0.5293828	0.18870759	-2.8053072	0.02217767
LSU ribosomal protein L6p (L9e)	15017.5692	-0.5313982	0.14429804	-3.682643	0.0018361
Putative HTH-type transcriptional regulator ypdC	406.659181	-0.5319167	0.14091367	-3.7747698	0.00135728
CDP-diacylglycerol--glycerol-3-phosphate 3-phosphatidyltransferase	2385.76425	-0.5321028	0.16237902	-3.2769188	0.00640651
Putative TonB dependent outer membrane receptor	989.055104	-0.5322383	0.14539519	-3.6606318	0.00198043
Putative metalloprotease yggG	144.108077	-0.5351779	0.18321578	-2.9210253	0.01654632
FIG00638699: hypothetical protein	201.743801	-0.535244	0.1617846	-3.3083743	0.00590078
FIG00639050: hypothetical protein	571.063377	-0.5356045	0.18446018	-2.903632	0.01729491
2-haloalkanoic acid dehalogenase	1093.7659	-0.5358225	0.15558201	-3.4439875	0.00390812
CFA/I fimbrial subunit C usher protein	1107.0402	-0.5364447	0.19835695	-2.7044411	0.02848609
Electron transport complex protein RnfG	395.807552	-0.5367518	0.19542051	-2.7466502	0.02572724
Transcriptional regulatory protein CitB%2C DpiA	291.767715	-0.5372467	0.18600715	-2.8883124	0.01786456
hypothetical protein	801.873444	-0.5373461	0.15903861	-3.3787147	0.00480627
Phosphate acetyltransferase	6588.46961	-0.5383344	0.12940634	-4.1600309	0.0003458
Septum formation protein Maf	1045.66316	-0.5392276	0.12307374	-4.3813371	0.00014939
regulator of length of O-antigen component of lipopolysaccharide chains	2406.58608	-0.5450381	0.18790458	-2.9006109	0.01742647
Phosphogluconate repressor HexR%2C RpiR family	466.925624	-0.5456673	0.17574916	-3.1048072	0.01032641
ABC-type hemin transport system%2C ATPase component	1198.03772	-0.5497861	0.14020733	-3.9212365	0.00081516
Protein At2g37660%2C chloroplast precursor	135.856251	-0.5499352	0.17892893	-3.0734839	0.01121822
16S rRNA (cytosine(967)-C(5))-methyltransferase	1150.43471	-0.5513534	0.1417573	-3.8894184	0.00090576
Pantothenate:Na+ symporter (TC 2.A.21.1.1)	419.347513	-0.5524205	0.143522	-3.8490304	0.00105014
Oligopeptide transport system permease protein OppC (TC 3.A.1.5.1)	1950.87834	-0.5528346	0.14994792	-3.6868445	0.00181242
FIG00642550: hypothetical protein	377.727543	-0.5541761	0.16430596	-3.3728303	0.00488882
Transporter%2C putative	997.065378	-0.5553204	0.14500126	-3.8297624	0.00111624
Fructose repressor FruR%2C LacI family	1546.25357	-0.5555112	0.14695216	-3.7802183	0.00133541
Proposed lipoate regulatory protein YbeD	542.850581	-0.5556769	0.17075694	-3.2541981	0.00684469
Pyruvate formate-lyase	158521.716	-0.5564265	0.20996308	-2.6501161	0.03229127
TonB-dependent receptor%3B Outer membrane receptor for ferric enterobactin and colicins B%2C D	26153.7044	-0.556752	0.1484766	-3.7497629	0.00148068
Cytochrome c551 peroxidase	92.3215826	-0.5577706	0.21226142	-2.6277531	0.0340946
2-keto-3-deoxy-D-arabino-heptulosonate-7-phosphate synthase I alpha	2469.18362	-0.5593376	0.1965726	-2.8454503	0.01994811
Endonuclease III	274.156569	-0.5604811	0.16171569	-3.4658425	0.00364217
Flagellar biosynthesis protein FliT	1685.66561	-0.5633984	0.18713021	-3.0107293	0.01323243
Cytochrome O ubiquinol oxidase subunit I	18674.4641	-0.5651159	0.1737092	-3.2532297	0.00684671
putative inner membrane protein	1539.26018	-0.5659602	0.1974052	-2.8669975	0.01888269
Periplasmic thiol:disulfide oxidoreductase DsbB%2Crequired for DsbA reoxidation	452.472166	-0.5663934	0.17718153	-3.1966843	0.00808081
Electron transport complex protein RnfD	443.335308	-0.5667036	0.17440761	-3.2493054	0.00691075
Iron(III) dicitrate transport system%2C periplasmic iron-binding protein FecB (TC 3.A.1.14.1)	2087.56051	-0.5698713	0.11764534	-4.8439764	2.23E-05
Methyl-accepting chemotaxis protein	11274.8531	-0.5706603	0.20884351	-2.732478	0.02653693

Flagellar hook-associated protein FlgL	13966.17	-0.5709941	0.13365441	-4.2721682	0.00022505
Transcriptional regulator for fatty acid degradation FadR%2C GntR family	953.20265	-0.5734443	0.1193018	-4.8066694	2.61E-05
Xylanase	547.531655	-0.5751289	0.13234024	-4.3458354	0.00017048
Putative two-component system response regulator YedW	184.390784	-0.5753404	0.20893798	-2.7536421	0.02528066
Hemin uptake protein HemP	330.917688	-0.5759298	0.17550039	-3.2816439	0.00631479
Rhodanese-related sulfurtransferases	1943.60315	-0.5809144	0.19471232	-2.9834495	0.01412324
Protein yihD	822.307619	-0.5810055	0.20509583	-2.832849	0.02066965
DnaA regulatory inactivator Hda (Homologous to DnaA)	1189.77062	-0.5821046	0.12830507	-4.536879	7.68E-05
Flagellar hook-basal body complex protein FliE	77.3849327	-0.5823976	0.23655862	-2.461959	0.04963642
Electron transport complex protein RnfC	1394.13816	-0.5857621	0.13769046	-4.254195	0.00023901
lipid A biosynthesis lauroyl acyltransferase	3388.67392	-0.5880925	0.12238461	-4.8052815	2.61E-05
Chemotaxis protein methyltransferase CheR	7460.05849	-0.5882758	0.14945893	-3.9360364	0.00077441
Putative amidase	11082.2711	-0.5907957	0.15656695	-3.7734385	0.00135946
MotA/TolQ/ExbB proton channel family protein	931.011323	-0.593401	0.19183783	-3.0932432	0.0106603
rRNA small subunit 7-methylguanosine (m7G) methyltransferase GidB	265.709324	-0.5983906	0.18061715	-3.313033	0.00583603
Putative thiosulfate sulfurtransferase ynjE	112.202213	-0.5987801	0.19339416	-3.0961645	0.01058158
tRNA (guanosine(18)-2'-O)-methyltransferase	570.259342	-0.5992025	0.13001865	-4.6085887	5.91E-05
LSU ribosomal protein L13p (L13Ae)	7106.46879	-0.5992031	0.129383	-4.6312354	5.39E-05
Nicotinamide-nucleotide adenylyltransferase%2C NadR family	121.627822	-0.5998813	0.17442936	-3.4391072	0.00396733
NADH dehydrogenase	8056.77471	-0.6001208	0.12755997	-4.7046174	3.98E-05
Putative membrane protein YfcA	521.263424	-0.6009675	0.14101843	-4.2616236	0.00023354
Radical SAM family enzyme%2C similar to coproporphyrinogen III oxidase%2Coxygen-independent%2Cclustered with nucleoside-triphosphatase RdgB	578.909509	-0.6023417	0.13165336	-4.5752095	6.68E-05
Ribose-phosphate pyrophosphokinase	4169.78225	-0.6067156	0.09820109	-6.1782982	2.44E-08
Capsular polysaccharide export system protein KpsS	406.806889	-0.6080348	0.21179742	-2.8708317	0.01869275
Flagellar motor rotation protein MotA	6588.39991	-0.6080978	0.15829481	-3.8415523	0.00107845
Pyridoxal kinase	716.701991	-0.6087251	0.13116552	-4.6408931	5.19E-05
Predicted N-ribosylNicotinamide CRP-like regulator	125.389248	-0.6115316	0.22756389	-2.6872965	0.02950264
Protein-export membrane protein SecD (TC 3.A.5.1.1)	5136.19535	-0.6138082	0.12001747	-5.1143235	6.85E-06
16S rRNA processing protein RimM	10176.7337	-0.6147445	0.12204913	-5.0368611	9.58E-06
LSU ribosomal protein L28p	3203.98423	-0.6155653	0.13280531	-4.6350955	5.32E-05
Antiholin-like protein LrgA	251.011027	-0.6160694	0.15644635	-3.9378955	0.00077002
Putative Diguanylate cyclase/phosphodiesterase domain 1	3210.01731	-0.6168721	0.1155086	-5.3404862	2.27E-06
Dipeptide transport system permease protein DppB (TC 3.A.1.5.2)	628.318738	-0.6169999	0.18239864	-3.3827002	0.00474397
Carbamoyl-phosphate synthase small chain	815.036749	-0.6172297	0.20007295	-3.0850232	0.01085687
FIG00638676: hypothetical protein	481.750213	-0.6180887	0.22789458	-2.7121694	0.02790724
Formate dehydrogenase H	261.468475	-0.6201919	0.23837854	-2.6017103	0.03628772
Cell division trigger factor	7752.5804	-0.620767	0.12470118	-4.9780362	1.24E-05
Acetate kinase	4674.12895	-0.6216035	0.14993981	-4.1456867	0.00036401
RNA polymerase sigma factor FecI	4674.94521	-0.6230556	0.15668194	-3.9765632	0.00066738
Putative membrane protein	246.982285	-0.6241304	0.13768055	-4.5331779	7.75E-05
Penicillin-binding protein 2 (PBP-2)	935.818573	-0.6264196	0.15525786	-4.034705	0.00054359
LSU ribosomal protein L34p	274.702499	-0.6291344	0.19078555	-3.2975996	0.00605662
2-amino-4-hydroxy-6-hydroxymethyldihydropteridine pyrophosphokinase	207.724558	-0.6293355	0.20179195	-3.1187343	0.00999404
GTP-binding protein Obg	2231.98927	-0.6298523	0.14589056	-4.3172928	0.00018874
Ribose ABC transport system%2C high affinity permease RbsD (TC 3.A.1.2.1)	1357.5779	-0.631597	0.24951978	-2.5312503	0.04287184
FIG00638308: hypothetical protein	1904.10203	-0.632356	0.1533404	-4.1238705	0.00039283
Arginine ABC transporter%2C periplasmic arginine-binding protein ArtJ	925.792316	-0.6348849	0.19010209	-3.3397051	0.00538604
FIG00638396: hypothetical protein	1887.57128	-0.6351438	0.17686501	-3.5911219	0.00248395
Protein YkiA	171.512217	-0.6372198	0.19718561	-3.2315736	0.00727749
Ornithine carbamoyltransferase	1797.07289	-0.6381385	0.14686935	-4.34494	0.00017072
Deoxyribonuclease TatD	501.713515	-0.6390546	0.17331801	-3.6871792	0.00181242
ATP-dependent DNA helicase RecG	1406.12534	-0.641655	0.12679827	-5.0604397	8.66E-06
tRNA (Guanine37-N1)-methyltransferase	10920.569	-0.6438212	0.12474361	-5.161156	5.45E-06
FIG00613574: hypothetical protein	2227.58254	-0.6440949	0.11832777	-5.4433114	1.35E-06
Transcription elongation factor GreA	504.84441	-0.6457996	0.19022694	-3.3948906	0.00457102
Holliday junction resolvase / Crossover junction endodeoxyribonuclease rusA	87.7136731	-0.6471992	0.2313676	-2.7972768	0.02261968
Signal peptidase I	2060.32481	-0.6490217	0.13098064	-4.9550964	1.38E-05
Probable acyltransferase yihG	58.7316048	-0.6501904	0.25790659	-2.5210308	0.04393608
iron aquisition yersiniabactin synthesis enzyme (Irp2)	40439.8936	-0.6505885	0.16402667	-3.9663578	0.00069079
Methionine repressor MetJ	730.40687	-0.6516779	0.15979903	-4.0781093	0.00046053
Transposase ECs0136	102.820277	-0.6519462	0.20854341	-3.1261896	0.00980359
Ferrichrome-iron receptor	752.47826	-0.656616	0.24313116	-2.7006659	0.02875862
Iron(III) dicitrate transmembrane sensor protein FecR	10308.2193	-0.6587652	0.12947976	-5.0877847	7.68E-06
Ribonuclease HII	753.84824	-0.6590683	0.11091867	-5.9419057	9.19E-08
Uracil permease	157.3716	-0.6592649	0.20119082	-3.2768143	0.00640651
AmpG permease	300.213715	-0.6601814	0.21844133	-3.0222368	0.01286929
Membrane-bound lytic murein transglycosylase D precursor	2364.25065	-0.6626053	0.14802395	-4.4763381	9.92E-05
Inosine-guanosine kinase	945.745678	-0.6639907	0.17017508	-3.9018091	0.00086233
Flagellar biosynthesis protein FlhA	925.576248	-0.6643163	0.13689354	-4.8527949	2.17E-05
tRNA-guanine transglycosylase	1108.73629	-0.6657512	0.13309527	-5.0020651	1.12E-05
YjcB protein	770.081553	-0.6676545	0.22098348	-3.0212868	0.01289515
FxsA protein	367.575725	-0.6720807	0.19362	-3.4711324	0.00358753
Type I restriction-modification system%2Cspecificity subunit S	361.448884	-0.6729412	0.1410373	-4.7713706	3.04E-05
Iron(III) dicitrate transport protein FecA	6196.17479	-0.6790644	0.10734977	-6.3257176	1.06E-08
Putative S-transferase	106.129579	-0.6791765	0.2067679	-3.284729	0.00627427
Spermidine Putrescine ABC transporter permease component potC (TC_3.A.1.11.1)	378.604011	-0.6818442	0.20024129	-3.405113	0.00442277
tRNA:Cm32/Um32 methyltransferase	1750.013	-0.6842178	0.12900928	-5.3036322	2.75E-06
Ferric hydroxamate ABC transporter (TC 3.A.1.14.3)%2C periplasmic substrate binding protein FhuD	1481.15143	-0.6848927	0.11286336	-6.0683349	4.61E-08
tRNA(Cytosine32)-2-thiocytidine synthetase	325.403142	-0.6875602	0.15278389	-4.500214	8.92E-05
Rod shape-determining protein MreD	400.157075	-0.6878692	0.13690338	-5.0244866	1.01E-05
FIG003671: Metal-dependent hydrolase	50.8489424	-0.6897048	0.26726515	-2.5806014	0.0381273
MFS superfamily export protein YceL	1185.63428	-0.6899476	0.21272762	-3.243338	0.00703956
Putative F1C and S fimbrial switch Regulatory protein	695.691731	-0.6904829	0.21036623	-3.2822896	0.00630913
Ferrous iron-sensisng transcriptional regulator FeoC	2347.48794	-0.6906907	0.17256903	-4.0024024	0.00061007
LSU ribosomal protein L9p	7309.13822	-0.6914604	0.14091008	-4.9071041	1.72E-05
FIG00643651: hypothetical protein	500.186742	-0.6927138	0.19478013	-3.5563882	0.00278096
FIG004454: RNA binding protein	319.887521	-0.6942754	0.21675179	-3.2030895	0.00792353
SSU ribosomal protein S16p	1583.63638	-0.6942937	0.13795336	-5.0328145	9.74E-06
LSU ribosomal protein L7/L12 (P1/P2)	7189.81315	-0.6950179	0.1399871	-4.964871	1.32E-05
Di/tripeptide permease YbgH	1120.99953	-0.6966691	0.17945618	-3.8821129	0.00092972
FIG00638928: hypothetical protein	298.630858	-0.6981196	0.17781314	-3.9261417	0.00080199
FIG00639097: hypothetical protein	40.8008187	-0.7028861	0.28170185	-2.495142	0.04637377
LSU ribosomal protein L22p (L17e)	5798.05079	-0.7104902	0.18509382	-3.8385409	0.00108964
Crotonobetainyl-CoA dehydrogenase	204.188998	-0.7160645	0.19623815	-3.6489566	0.00205784
Multidrug resistance protein D	229.409142	-0.7203779	0.18323672	-3.9314058	0.00078786
Transcriptional repressor for pyruvate dehydrogenase complex	2420.05459	-0.7246215	0.15956963	-4.5410991	7.55E-05
Iron(III) dicitrate transport system permease protein FecD (TC 3.A.1.14.1)	311.066747	-0.7249015	0.15224849	-4.7613054	3.15E-05
Dethiobiotin synthetase	602.869413	-0.725663	0.21626374	-3.3554537	0.0051395
ID=gene:EBG00000313244	70.8350168	-0.725848	0.29316561	-2.4758975	0.04828337
Ribosomal protein S12p Asp88 (E. coli) methylthiotransferase	575.182883	-0.7296295	0.17737234	-4.1135472	0.00040704
SSU ribosomal protein S7p (S5e)	6659.0418	-0.7299363	0.12279407	-5.9443931	9.18E-08
Lipoprotein nlpI precursor	28890.9861	-0.730719	0.13122613	-5.5683953	7.04E-07
FIG00638745: hypothetical protein	97.3492211	-0.7326361	0.2038304	-3.5943417	0.00247319
DNA recombination and repair protein RecF	480.588226	-0.7327044	0.18232141	-4.0187512	0.00057543
Putative polyketide synthase	12346.8957	-0.7382148	0.15840418	-4.6603244	4.77E-05
Putative membrane protein	74.2474815	-0.7419864	0.25426984	-2.9181063	0.01668456
hypothetical protein	103.3483	-0.7451306	0.28418767	-2.6219666	0.03452855
Cold shock protein CspE	20839.2492	-0.7534916	0.26730482	-2.8188477	0.02144479
Alcohol dehydrogenase	339377.314	-0.7568211	0.20727592	-3.6512736	0.00204338

Serine transporter	165.408819	-0.7595483	0.212594	-3.5727645	0.0026487
Adenylate kinase	1506.15856	-0.761586	0.17335946	-4.3931032	0.00014192
Putative vimentin	8629.64874	-0.7630312	0.16896854	-4.5158179	8.31E-05
Mg-chelatase subunit ChlD	52.4019351	-0.7631503	0.28381633	-2.688888	0.02946959
LSU ribosomal protein L33p @ LSU ribosomal protein L33p%2C zinc-independent	2289.36561	-0.7668672	0.13922867	-5.5079692	9.51E-07
Formate efflux transporter (TC 2.A.44 family)	6793.22688	-0.7686879	0.17754028	-4.3296533	0.00018009
Cold shock protein CspG	668.951754	-0.7731291	0.27010067	-2.8623738	0.01914116
chaperone FimC	4003.03114	-0.7770408	0.18726205	-4.1494834	0.00036058
FIG00638753: hypothetical protein	186.163003	-0.7791891	0.18701356	-4.1664849	0.00033795
Flagellar biosynthesis protein FliZ	2627.83053	-0.7812604	0.22084508	-3.5375947	0.00294812
FIG00637977: hypothetical protein	54.1013557	-0.7813842	0.25996537	-3.0057242	0.01337692
hypothetical protein	1490.33042	-0.7820024	0.21082236	-3.7092955	0.00168877
Ni/Fe-hydrogenase 2 B-type cytochrome subunit	162.139041	-0.7835281	0.19696674	-3.9779718	0.00066625
Xanthine-guanine phosphoribosyltransferase	420.373316	-0.784724	0.21100015	-3.7190683	0.00164551
Multimodular transpeptidase-transglycosylase	1102.0615	-0.7851494	0.15381523	-5.1044971	7.16E-06
Iron(III) dicitrate transport ATP-binding protein FecE (TC 3.A.1.14.1)	506.579689	-0.7869178	0.14164736	-5.5554712	7.47E-07
Succinate dehydrogenase flavoprotein subunit	3438.81666	-0.7870019	0.16160631	-4.8698713	2.03E-05
Putative inner membrane protein	9765.06262	-0.7886912	0.15726943	-5.0149047	1.06E-05
Inner membrane protein YccF	87.8181866	-0.7931317	0.27603955	-2.8732538	0.01860624
FIG00637875: hypothetical protein	1036.30947	-0.7951098	0.13953417	-5.698316	3.47E-07
LSU ribosomal protein L1p (L10Ae)	13816.5504	-0.7953975	0.14895736	-5.3397662	2.27E-06
tRNA dihydrouridine synthase B	2403.73298	-0.7968735	0.17168847	-4.6413921	5.19E-05
Mobile element protein	260.278214	-0.7989789	0.26869378	-2.9735667	0.01439731
SSU ribosomal protein S6p	5694.04185	-0.8038775	0.14465511	-5.5572008	7.44E-07
LSU ribosomal protein L19p	2715.93582	-0.8134661	0.17191662	-4.7317475	3.55E-05
Phosphate:acyl-ACP acyltransferase PlsX	2135.21453	-0.8171172	0.12198816	-6.6983323	1.05E-09
SSU ribosomal protein S12p (S23e)	4859.21461	-0.8220996	0.1410455	-5.8286125	1.75E-07
Cytochrome O ubiquinol oxidase subunit II	8174.7509	-0.8253167	0.14660413	-5.6295599	5.06E-07
FIG00639065: hypothetical protein	39.4776029	-0.8266911	0.32199648	-2.5673917	0.03937157
Transposase YhgA	71.9915992	-0.8280245	0.23744765	-3.4871875	0.00342402
Iron(III) dicitrate transport system permease protein FecC (TC 3.A.1.14.1)	409.710578	-0.8291408	0.11975131	-6.9238559	2.34E-10
ID=gene:EBG00000313283	35.9648682	-0.8299305	0.32994529	-2.5153579	0.04441064
ID=gene:EBG00000313290	35.9648682	-0.8299305	0.32994529	-2.5153579	0.04441064
Probable microcin H47 secretion/processing ATP-binding protein mchF	1897.19603	-0.8308193	0.1400337	-5.9329957	9.63E-08
FIG00639275: hypothetical protein	356.487003	-0.8308392	0.16727413	-4.9669317	1.31E-05
UPF0313 protein ygiQ	853.764384	-0.8346244	0.19053106	-4.3805163	0.00014953
LSU ribosomal protein L10p (P0)	9807.32752	-0.8356813	0.14575396	-5.7335064	2.92E-07
Colicin I receptor precursor	8828.14569	-0.8456034	0.19804088	-4.2698428	0.00022625
ID=gene:EBG00000313275	48.5788764	-0.8474508	0.27823935	-3.045762	0.01202632
ID=gene:EBG00000313277	48.5788764	-0.8474508	0.27823935	-3.045762	0.01202632
ID=gene:EBG00000313278	48.5788764	-0.8474508	0.27823935	-3.045762	0.01202632
ID=gene:EBG00000313281	48.5788764	-0.8474508	0.27823935	-3.045762	0.01202632
ID=gene:EBG00000313282	48.5788764	-0.8474508	0.27823935	-3.045762	0.01202632
ID=gene:EBG00000313305	48.5788764	-0.8474508	0.27823935	-3.045762	0.01202632
Arginine/ornithine antiporter ArcD	253.725426	-0.8475075	0.15838114	-5.3510635	2.17E-06
Translation initiation factor 1	1399.23179	-0.8485328	0.17497622	-4.8494176	2.19E-05
Serine transporter	370.853602	-0.8566234	0.16878352	-5.0752788	8.12E-06
SSU ribosomal protein S14p (S29e) @ SSU ribosomal protein S14p (S29e)%2C zinc-independent	12694.6835	-0.8607791	0.12371149	-6.9579564	1.89E-10
FIG00639812: hypothetical protein	34.03305	-0.8657513	0.34792685	-2.488314	0.0470445
Inner membrane protein translocase component YidC%2C long form	4376.35652	-0.8726664	0.18486502	-4.72056	3.73E-05
Oligopeptide transport system permease protein OppB (TC 3.A.1.5.1)	1949.61076	-0.8742428	0.17555897	-4.9797675	1.24E-05
MchC protein	2724.36984	-0.8777576	0.16974602	-5.1710056	5.23E-06
LSU ribosomal protein L2p (L8e)	15822.2664	-0.8812465	0.17012792	-5.1799054	5.03E-06
Putative protease	856.675488	-0.8828043	0.15446061	-5.7154004	3.18E-07
4-hydroxybenzoyl-CoA thioesterase family active site	311.52654	-0.8847746	0.23803706	-3.7169615	0.00165617
LSU ribosomal protein L5p (L11e)	16545.5443	-0.8925039	0.1347744	-6.6222065	1.71E-09
Ribonuclease P protein component	923.659149	-0.8984335	0.21705259	-4.1392435	0.00037325
Flagellar biosynthesis protein FlhB	437.932851	-0.8989436	0.18630073	-4.8252284	2.44E-05
Hypothetical protein yqgB	54.4410204	-0.9062861	0.31471173	-2.8797341	0.01827833
hypothetical protein	166.841032	-0.9097051	0.25742866	-3.5338145	0.0029858
SSU ribosomal protein S19p (S15e)	1941.75408	-0.9097771	0.18001943	-5.0537715	8.85E-06
Permease of the drug/metabolite transporter (DMT) superfamily	891.750038	-0.9136549	0.22217121	-4.1123911	0.00040815
LSU ribosomal protein L23p (L23Ae)	6314.60346	-0.9138743	0.17608874	-5.1898506	4.80E-06
Glutamyl-tRNA reductase	1716.31879	-0.9193003	0.14865779	-6.1840036	2.38E-08
Glucans biosynthesis protein C	89.3560546	-0.9203646	0.26364886	-3.4908728	0.00340542
ID=gene:EBG00000313286	130.832878	-0.928616	0.3084297	-3.0107866	0.01323243
ID=gene:EBG00000313289	130.832878	-0.928616	0.3084297	-3.0107866	0.01323243
FIG00642441: hypothetical protein	126.095384	-0.9332146	0.18910087	-4.9350096	1.52E-05
ID=gene:EBG00000313274	47.1612848	-0.9417136	0.34312762	-2.7444995	0.02572801
ID=gene:EBG00000313276	47.1612848	-0.9417136	0.34312762	-2.7444995	0.02572801
ID=gene:EBG00000313306	47.1612848	-0.9417136	0.34312762	-2.7444995	0.02572801
ID=gene:EBG00000313308	47.1612848	-0.9417136	0.34312762	-2.7444995	0.02572801
ID=gene:EBG00000313311	47.1612848	-0.9417136	0.34312762	-2.7444995	0.02572801
Flagellar motor switch protein FliN	278.499722	-0.9437199	0.26011769	-3.6280495	0.00220952
Putative inner membrane protein YjeT (clustered with HflC)	68.2726868	-0.9468962	0.23582941	-4.0151746	0.00058296
SSU ribosomal protein S8p (S15Ae)	6952.40928	-0.9480547	0.13047253	-7.2663165	2.29E-11
hypothetical protein	63.9334328	-0.952447	0.25265714	-3.7697212	0.00137731
Inactive homolog of metal-dependent proteases%2Cputative molecular chaperone	300.01309	-0.9556303	0.22268842	-4.2913339	0.00020916
SSU ribosomal protein S18p @ SSU ribosomal protein S18p%2C zinc-independent	4196.40735	-0.9612083	0.13159303	-7.3044011	1.78E-11
hypothetical protein	59.0841954	-0.9743514	0.28360338	-3.4356128	0.00400085
Putative secretion permease	676.7642	-0.9769247	0.16378944	-5.9645157	8.18E-08
Flagellar motor switch protein FliM	428.354608	-0.9812725	0.21498114	-4.5644585	6.88E-05
FIG00638351: hypothetical protein	231.834055	-0.9976626	0.20842487	-4.7866772	2.85E-05
Peptide chain release factor 1	1137.37889	-1.002938	0.12674225	-7.9132099	2.07E-13
RNA polymerase sigma factor for flagellar operon	9275.76089	-1.0049511	0.18800155	-5.3454402	2.22E-06
Flagellar biosynthesis protein FliQ	203.143854	-1.0126807	0.24820536	-4.0800112	0.00045781
FIG00641578: hypothetical protein	1095.18172	-1.0130774	0.37209515	-2.7226299	0.02723957
Hybrid sensory histidine kinase in two-component regulatory system with EvgA	1416.36106	-1.0179169	0.1769862	-5.7513914	2.66E-07
LSU ribosomal protein L24p (L26e)	9238.05816	-1.0189529	0.12174709	-8.3694228	5.97E-15
Flagellar biosynthesis protein FliL	229.678194	-1.0277344	0.29473935	-3.4869262	0.00342402
LSU ribosomal protein L14p (L23e)	7100.06713	-1.0300291	0.15446356	-6.668428	1.27E-09
Putative transcriptional regulator LYSR-type	66.8604371	-1.0375367	0.27843297	-3.726343	0.00160489
Mobile element protein	198.095139	-1.0405555	0.21742207	-4.7858778	2.85E-05
Flagellar basal-body rod modification protein FlgD	1456.38122	-1.0413767	0.27580738	-3.7757389	0.00135455
Ornithine carbamoyltransferase	104.83429	-1.0434434	0.3300103	-3.1618511	0.00892433
ID=gene:EBG00000313297	43.3192899	-1.0462181	0.31199637	-3.3533021	0.00517223
Putative fimbrial-like protein	31.9381756	-1.048183	0.40718722	-2.5742041	0.0387031
Flagellar protein FlgJ [peptidoglycan hydrolase]	516.178169	-1.0566755	0.25882621	-4.0825675	0.00045382
Flagellar basal-body P-ring formation protein FlgA	418.213254	-1.0585113	0.26786432	-3.9516696	0.00073155
LSU ribosomal protein L4p (L1e)	14053.1302	-1.0732312	0.1689898	-6.3508641	9.16E-09
Flagellar hook protein FlgE	3234.93459	-1.0804059	0.25543247	-4.2297125	0.00026195
LSU ribosomal protein L3p (L3e)	16017.8849	-1.0846584	0.14998406	-7.2318244	2.92E-11
Flagellar basal-body rod protein FlgG	1152.20791	-1.0891292	0.26894727	-4.049601	0.00051237
Flagellar motor switch protein FliG	657.294734	-1.0962131	0.23458924	-4.6729046	4.53E-05
Fumarate hydratase class I%2C anaerobic	274.383422	-1.0971524	0.19619368	-5.5921904	6.20E-07
FIG00637865: hypothetical protein	768.794502	-1.1018401	0.20264882	-5.4371899	1.38E-06
hypothetical protein	77.7115028	-1.1064554	0.25694541	-4.3061887	0.00019714
FIG139552: Putative protease	59.5981328	-1.1096529	0.26558931	-4.1780783	0.00032352
Putative exported protein	89.0434493	-1.1162708	0.28553523	-3.9093977	0.0008458

ID=gene:EBG00000313300	34.2567863	-1.1232303	0.33954968	-3.308	0.00590078
LSU ribosomal protein L31p @ LSU ribosomal protein L31p%2C zinc-dependent	1228.26454	-1.1365431	0.18736387	-6.065967	4.65E-08
Di/tripeptide permease DtpA	3846.66891	-1.1380404	0.14539726	-7.8271106	3.97E-13
Outer membrane protein W precursor	424.516582	-1.1433658	0.25466427	-4.4896986	9.35E-05
Flagellar protein FliJ	87.6135071	-1.1467892	0.34701984	-3.3046791	0.00595464
Putrescine importer	761.255253	-1.1467994	0.24843347	-4.6161226	5.72E-05
ATP-dependent RNA helicase RhlE	1374.56084	-1.1475905	0.14931738	-7.685579	1.15E-12
Hydrogenase-2 operon protein hybA precursor	212.171858	-1.15025	0.1631008	-7.0523875	9.84E-11
FIG139928: Putative protease	71.9385186	-1.1507496	0.25251971	-4.5570682	7.08E-05
DNA-binding protein Fis	659.728129	-1.1530279	0.18739192	-6.1530291	2.83E-08
Flagellar basal-body rod protein FlgF	1045.642	-1.1597956	0.27518272	-4.2146381	0.00027734
Periplasmic binding protein	386.489621	-1.1712581	0.1537669	-7.6171015	1.93E-12
SSU ribosomal protein S21p	406.609056	-1.1792313	0.24339363	-4.8449554	2.23E-05
Arginine deiminase	98.0274479	-1.1877162	0.27126695	-4.3784035	0.00015057
Flagellar P-ring protein FlgI	744.596026	-1.1877786	0.24116135	-4.9252445	1.59E-05
Flagellum-specific ATP synthase FliI	534.243086	-1.1911626	0.28613463	-4.1629448	0.00034241
Flagellar basal-body rod protein FlgC	712.104017	-1.1963352	0.37458417	-3.1937687	0.00813159
ID=gene:EBG00000313288	64.0815478	-1.2054349	0.38538032	-3.1279099	0.00977025
Molybdenum transport system protein ModD	176.300695	-1.2099629	0.21482376	-5.6323515	5.01E-07
Flagellar protein FlhE	130.129162	-1.2133162	0.23314493	-5.2041284	4.56E-06
Flagellar basal-body rod protein FlgB	481.631213	-1.228433	0.28359146	-4.3316994	0.0001789
Flagellar L-ring protein FlgH	485.48977	-1.2357403	0.29046689	-4.2543241	0.00023901
Cold-shock DEAD-box protein A	22647.6572	-1.2368604	0.10746188	-11.50976	2.54E-28
Flagellar assembly protein FliH	303.562829	-1.2390429	0.26808076	-4.6219017	5.60E-05
LSU ribosomal protein L11p (L12e)	4015.01682	-1.2497356	0.16725303	-7.4721253	5.50E-12
Uptake hydrogenase small subunit precursor	587.458305	-1.2527273	0.13494505	-9.2832402	2.01E-18
FIG00638267: hypothetical protein	42.5372276	-1.2586241	0.29721126	-4.2347792	0.00025887
4'-phosphopantetheinyl transferase	1161.89135	-1.2586377	0.16279821	-7.7312752	8.18E-13
Diaminopimelate decarboxylase	6472.01914	-1.2673289	0.26075653	-4.8602002	2.10E-05
Flagellar biosynthesis protein FliP	88.3318728	-1.2794513	0.24977935	-5.122326	6.61E-06
SSU ribosomal protein S20p	1230.55228	-1.2880937	0.20449539	-6.298889	1.21E-08
MchD protein	525.726398	-1.2926724	0.1754473	-7.3678674	1.17E-11
SSU ribosomal protein S10p (S20e)	6299.56762	-1.298552	0.16088602	-8.0712546	6.19E-14
Protein-N(5)-glutamine methyltransferase PrmC%2Cmethylates polypeptide chain release factors RF1 and RF2	527.212162	-1.3096719	0.14574549	-8.9860196	3.06E-17
Putative exported protein	988.445577	-1.317259	0.42000411	-3.1363003	0.00958887
FIG002082: Protein SirB2	122.610051	-1.3185397	0.23755097	-5.5505547	7.64E-07
Hypothetical MFS-type transporter protein YcaD	1178.62952	-1.3403891	0.37041616	-3.6186032	0.00228625
Hemin ABC transporter%2C permease protein	142.024875	-1.3538261	0.2143956	-6.3146169	1.13E-08
Membrane protein with DUF350 domain	21.5135249	-1.3589607	0.37733942	-3.6014279	0.00241159
Flagellar M-ring protein FliF	458.724116	-1.3603312	0.2786814	-4.8813132	1.93E-05
ID=gene:EBG00000313299	53.1112194	-1.3901312	0.37562037	-3.7008939	0.00172711
Putative outer membrane protein	268.136693	-1.4216495	0.23463969	-6.0588618	4.75E-08
C4-dicarboxylate transporter DcuB	170.574644	-1.4331912	0.23439288	-6.1144829	3.54E-08
ID=gene:EBG00000313298	43.2066235	-1.4820631	0.30299965	-4.891303	1.85E-05
Cobalt-zinc-cadmium resistance protein CzcA%3B Cation efflux system protein CusA	973.233768	-1.4825367	0.36304077	-4.0836644	0.0004527
Orf2	204.742121	-1.5093893	0.23435949	-6.4404874	5.29E-09
ABC-type hemin transport system%2C ATPase component	161.937159	-1.5186477	0.1914517	-7.9322757	1.81E-13
ID=gene:EBG00000313230	15.764247	-1.561168	0.49899132	-3.1286477	0.0097577
Cold shock protein CspA	14785.1522	-1.5884546	0.17937344	-8.8555734	9.68E-17
hypothetical protein	44.467851	-1.5890812	0.27591017	-5.7594152	2.55E-07
ID=gene:EBG00000313231	31.5216564	-1.6174331	0.45933209	-3.5212718	0.00311563
hypothetical protein	93.1084095	-1.6177744	0.34573522	-4.6792294	4.43E-05
Inositol-1-monophosphatase	1023.44228	-1.6456319	0.26529372	-6.2030563	2.15E-08
FIG00637915: hypothetical protein	107.221215	-1.6587678	0.32634116	-5.0829254	7.84E-06
hypothetical protein	249.155953	-1.6707535	0.6009494	-2.7801901	0.0236164
Ferrichrome-iron receptor	1823.59572	-1.7110682	0.16778739	-10.197836	3.28E-22
Carbamate kinase	57.0106501	-1.7301014	0.33421484	-5.1766147	5.10E-06
Lead%2C cadmium%2C zinc and mercury transporting ATPase	2026.16705	-1.7735686	0.17297129	-10.253543	1.92E-22
Pyruvate formate-lyase	43339.8671	-1.7868553	0.2519247	-7.0928148	7.54E-11
ID=gene:EBG00000313307	25.4326063	-1.8946258	0.56371717	-3.3609511	0.00507051
ID=gene:EBG00000313312	25.4326063	-1.8946258	0.56371717	-3.3609511	0.00507051
ID=gene:EBG00000313225	33.9133917	-2.1102381	0.48694555	-4.3336223	0.00017781
FIG00638707: hypothetical protein	86.0994746	-2.5729017	0.3141226	-8.1907564	2.45E-14
Cobalt/zinc/cadmium efflux RND transporter%2Cmembrane fusion protein%2C CzcB family	607.183489	-3.1720634	0.7745105	-4.0955718	0.00043593
Cation efflux system protein CusC precursor	238.119615	-3.7191147	0.92758112	-4.0094765	0.00059463
ID=gene:EBG00000313254	55.1263514	-4.157494	1.46105968	-2.8455333	0.01994811
Cation efflux system protein CusF precursor	112.910026	-4.589993	1.01386839	-4.5272079	7.94E-05
ID=gene:EBG00000313296	506.024089	-4.6713897	0.95792248	-4.8765843	1.97E-05
